# Role of epigenetics in the etiology of hypospadias through penile foreskin DNA methylation alterations

**DOI:** 10.1038/s41598-023-27763-5

**Published:** 2023-01-11

**Authors:** Martin Kaefer, Richard Rink, Rosalia Misseri, Paul Winchester, Cathy Proctor, Millissia Ben Maamar, Daniel Beck, Eric Nilsson, Michael K. Skinner

**Affiliations:** 1 Department of Pediatric Urology, Riley Hospital for Children, Indiana University School of Medicine, Indiana University, Indianapolis, IN 46202-5201 USA; 2 Department of Pediatrics, St. Franciscan Hospital, School of Medicine, Indiana University, Indianapolis, IN 46202-5201 USA; 3 Center for Reproductive Biology, School of Biological Sciences, Washington State University, Pullman, WA 99164-4236 USA

**Keywords:** Hypospadias, DNA methylation, Organogenesis

## Abstract

Abnormal penile foreskin development in hypospadias is the most frequent genital malformation in male children, which has increased dramatically in recent decades. A number of environmental factors have been shown to be associated with hypospadias development. The current study investigated the role of epigenetics in the etiology of hypospadias and compared mild (distal), moderate (mid shaft), and severe (proximal) hypospadias. Penile foreskin samples were collected from hypospadias and non-hypospadias individuals to identify alterations in DNA methylation associated with hypospadias. Dramatic numbers of differential DNA methylation regions (DMRs) were observed in the mild hypospadias, with reduced numbers in moderate and low numbers in severe hypospadias. Atresia (cell loss) of the principal foreskin fibroblast is suspected to be a component of the disease etiology. A genome-wide (> 95%) epigenetic analysis was used and the genomic features of the DMRs identified. The DMR associated genes identified a number of novel hypospadias associated genes and pathways, as well as genes and networks known to be involved in hypospadias etiology. Observations demonstrate altered DNA methylation sites in penile foreskin is a component of hypospadias etiology. In addition, a potential role of environmental epigenetics and epigenetic inheritance in hypospadias disease etiology is suggested.

## Introduction

Hypospadias involves abnormal penile and urethral development in newborn males. This is one of the most frequent genital malformations in male children, which has increased dramatically in past decades^1–3^. The prevalence of hypospadias in the U.S. is approximately 6.8 per 1000 male births, which has increased from 6.1 per 1000 in 1997, for an 11.5% increase over 15 years^4^. This supports a potential environmental impact and role of epigenetic inheritance in hypospadias disease etiology^3,5,6^. The severity of hypospadias is classified as mild (distal penile opening near the tip of the penis) (50% of cases), moderate (mid shaft penile opening midway up the penis) (30%), and severe (proximal penile opening at or below the scrotum) (20%). Clinical factors associated with increased risk of hypospadias include: having an affected family member, maternal hypertension and preeclampsia, small for dates at birth, prematurity, and multiple gestation^7^. Although urethral formation occurs between the 8th and 14th week of pregnancy, the developmental origins or etiology of hypospadias is largely unknown^1–3^. The assumption is genetic mutations in critical genes will be the cause of the disease^7–10^. However, genome-wide association studies (GWAS) suggest less than 10% of the hypospadias patients have correlated genetic mutations^11^. Heritability estimates for hypospadias range between 57 and 77%^12–14^. Observations suggest a potential role of epigenetic inheritance^15^ of hypospadias and environmental impacts are now thought to be critical^8^. Since environmental factors such as toxicants or pharmaceuticals (diethylstilbestrol, DES) cannot directly alter DNA sequence, environmental epigenetics is thought to be the primary molecular mechanism involved^6^. Abnormal androgen production or action in later fetal development is associated with hypospadias^1^. It has been postulated that changes in concentrations of sex hormones during the crucial period of penile fetal development (weeks 8–14) through androgenesis and environmental exposures (i.e., endocrine disruptors) are major factors. Environmental factors such as diet and chemical exposures of the mother and fetus are also felt to likely be involved in the etiology of hypospadias^5,15–17^, and are known to act through epigenetics^6^.

Penile development is dependent on normal fibroblast maturation and eventual foreskin formation^1^. Abnormal foreskin development is one of the primary factors in hypospadias. This penile tissue is androgen responsive and thus would be vulnerable to endocrine disruptor exposures (i.e., anti-androgenic fungicide vinclozolin) throughout development. Male sexual differentiation regulates testosterone production in the testis and actions on the genital target tissues through the androgen receptor^1,2^, which includes foreskin development in male penile development. In addition to androgen action on the androgen receptor, Hox genes are also involved in male fibroblast development^9,10^. Environmental exposures and factors have also been shown to have dramatic impacts on the penile developmental process, independent of classic genetic mutations^5,15–17^. A number of reviews and literature have suggested a potential role for environmental epigenetics in the etiology of hypospadias^2,8,18,19^, but few direct experimental studies have been performed.

Epigenetics is defined as “molecular factors and processes around DNA that regulate genome activity, independent of DNA sequence, and are mitotically stable”^6,20^. The known molecular factors and processes involved are DNA methylation, histone modifications, chromatin structure, non-coding RNA, and RNA methylation^6^. All these factors can regulate gene expression directly, independent of DNA sequence. In contrast to DNA sequence that generally cannot be modified directly by environmental exposures, epigenetic mechanisms have evolved to respond to environmental factors to regulate gene expression and phenotypic variation^6^. Epigenetics operates in all areas of biology from cell and developmental biology to evolutionary biology^20,21^. Epigenetics is the precursor to any gene expression event and integrates with genetics to regulate all biological processes^6,20,21^, including reproductive development in the male and penile formation. Since epigenetics mediates environmental impacts on biology, epigenetic alterations that are associated with the etiology of hypospadias were investigated.

An additional element to environmental impacts on epigenetic regulation of biology^6^, is that when the epigenetic alterations become programmed into the germline (sperm or egg) they can be transmitted to the embryo after fertilization to influence the health of the offspring and subsequent generations. This phenomenon is termed environmentally induced epigenetic transgenerational inheritance and is a non-genetic form of inheritance that can be transmitted for hundreds of generations^6,22^. The phenomenon has been shown in all organisms from plants to humans^6,20^. The transgenerational occurrence of hypospadias in humans following diethylstilbestrol (DES) exposure has been suggested to be the result of epigenetic transgenerational inheritance^2,18,19,23^. Although no direct epigenetic analyses have been reported, the DES-induced transmission and inheritance of hypospadias has been observed^8,24–26^. Therefore, the increase in hypospadias frequency in the population over the past decades through environmental impacts may not simply be direct fetal exposure, but due in part to epigenetic transgenerational inheritance from ancestral exposures to environmental contaminants^8^. Other studies have linked severity of hypospadias with maternal exposure to environmental toxicants during pregnancy^27,28^.

The current study was designed to collect foreskin fibroblasts from mild (distal), moderate (mid shaft), and severe (proximal) hypospadias male patients and compare with foreskin from non-disease control population patients. The epigenetics (i.e., DNA methylation) in the foreskin cell populations were assessed and compared to identify differential DNA methylation regions (DMRs) associated with hypospadias disease etiology. The current study utilized a genome-wide DNA methylation analyses that potentially assesses greater than 95% of the genome^29^. An initial study on hypospadias previously published used an array based analysis of CpG islands that involves less than 1% of the genome, but did show differences in DNA methylation between control and hypospadias patient tissues^2^. A number of studies have demonstrated in environmentally exposed populations the presence of increased hypospadias frequency and suggested epigenetic effects, but generally did not directly assess epigenetic alterations^5,15–18,30–32^. The most common exposure correlated with hypospadias occurrence was diethylstilbestrol (DES). Various endocrine disruptors including 17α estradiol, progesterone, anti-androgenic pesticide vinclozolin, and the antihistamine loratadine at physiological levels cause hypospadias in animal models^33–37^. More recent studies have assessed epigenetic alterations at specific genomic sites^2,19,23^, but not at a genome-wide level. Therefore, the current study reports genome-wide alterations in DNA methylation in foreskin tissues from mild, moderate, and severe hypospadias patients to provide new insights into the etiology of hypospadias.

## Results

The objectives of the current study were to identify the DNA methylation alterations in the foreskin of newborn male children with and without hypospadias, and to compare epigenetic alterations between mild (distal), moderate (mid shaft), and severe (proximal) cases of hypospadias. The foreskin sample collections were found histologically to be greater than 90% pure fibroblast populations in the control and hypospadias tissue collected. Vascular tissue (< 5%) was the primary contaminant. Samples consisted of foreskin tissue collected during a Hypospadias surgical repair (cases) or a circumcision procedure (controls). Samples were processed and stored at − 20 °C following surgery. Prior to sample collection, approvals to conduct the study were obtained from Indiana University (IU) Institutional Review Board (IRB # 1503167555). The legal guardian of a study participant was consented, and then samples collected from Riley Hospital for Children IU Health and Franciscan Health, Indianapolis IN. The severity was categorized as mild (distal), moderate (mid shaft), and severe (proximal) based on the urethral meatus location at the time of the procedure, as identified by the Urologist. Hypospadias samples and circumcision foreskin tissues were analyzed to assess epigenetic alterations in each group specifically for comparison. The samples were obtained from non-Hispanic white Caucasian males less than 36 months of age between 2016 and 2018 with the male karyotype being confirmed with the sequence data, Supplemental Table S1. The control (non-disease) group (n = 15) had a mean ± SD age 13 ± 5.8 months, the mild hypospadias (n = 17) had a mean age 11 ± 4.2 months, the moderate hypospadias (n = 13) had a mean age 10 ± 3.8 months, and the severe hypospadias (n = 6) had a mean age 10 ± 3.2 months, Supplemental Table S1. There was no statistical difference in the mean age between the disease and control groups (p < 0.292). The sample identification, hospital, age, sex, and disease category are presented, Supplemental Table S1. The frozen samples were shipped on ice to Washington State University and stored at − 80 °C until DNA preparation and analysis.

**Figure 1:**
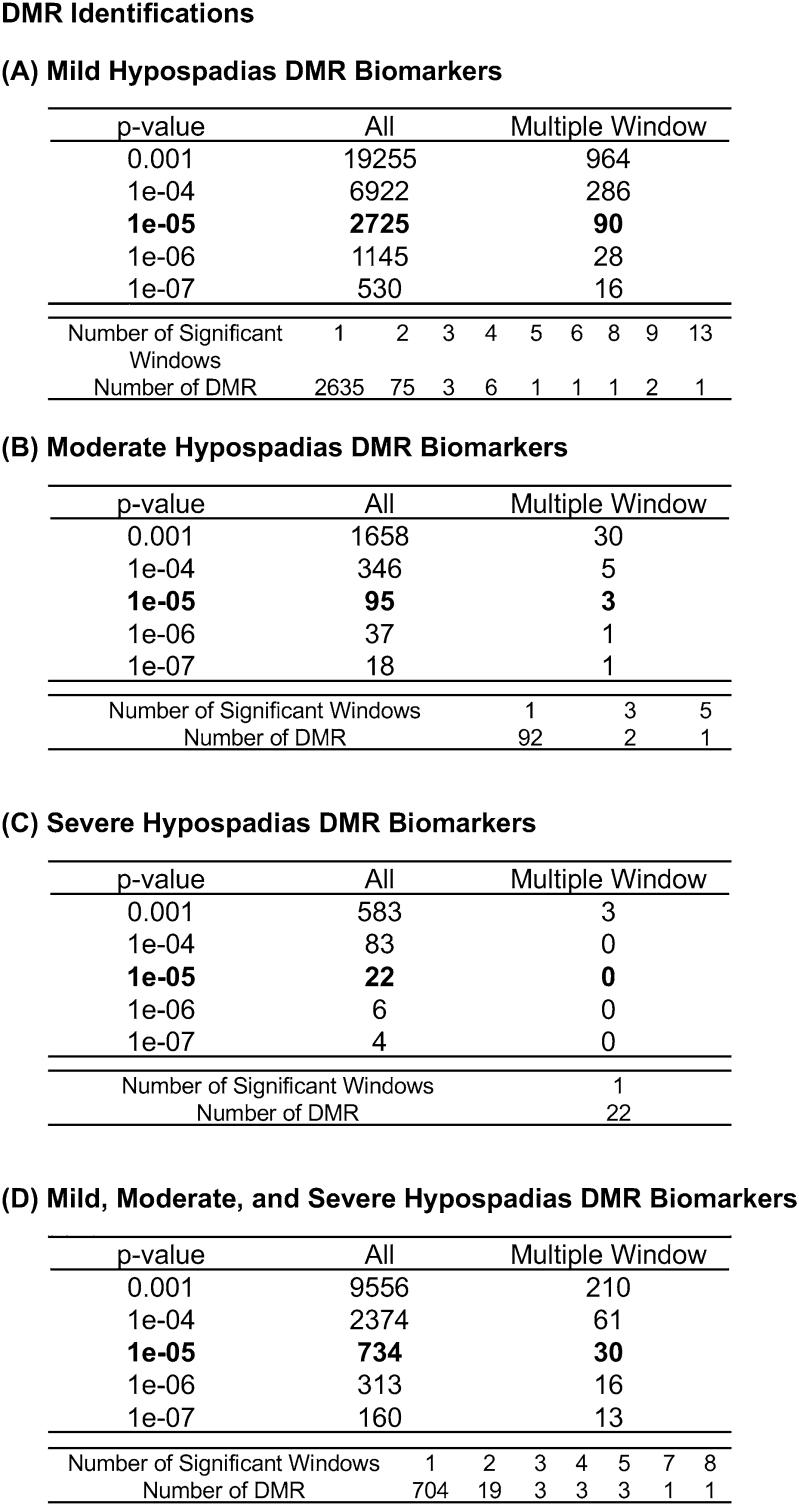
Hypospadias DMR. DMR identification and numbers. The number of DMRs found using different p value cutoff thresholds. The All Window column shows all DMRs. The Multiple Window column shows the number of DMRs containing at least two significant windows (1000 bp each). The number of DMRs with the number of significant windows (1000 bp per window) at a p value threshold of p < 1e−05 for DMR is presented. (**A**) Mild (distal) hypospadias DMRs. (**B**) Moderate (mid shaft) hypospadias DMRs. (**C**) Severe (proximal) hypospadias DMRs. (**D**) Combined mild, moderate, and severe hypospadias DMRs.

**Figure 2:**
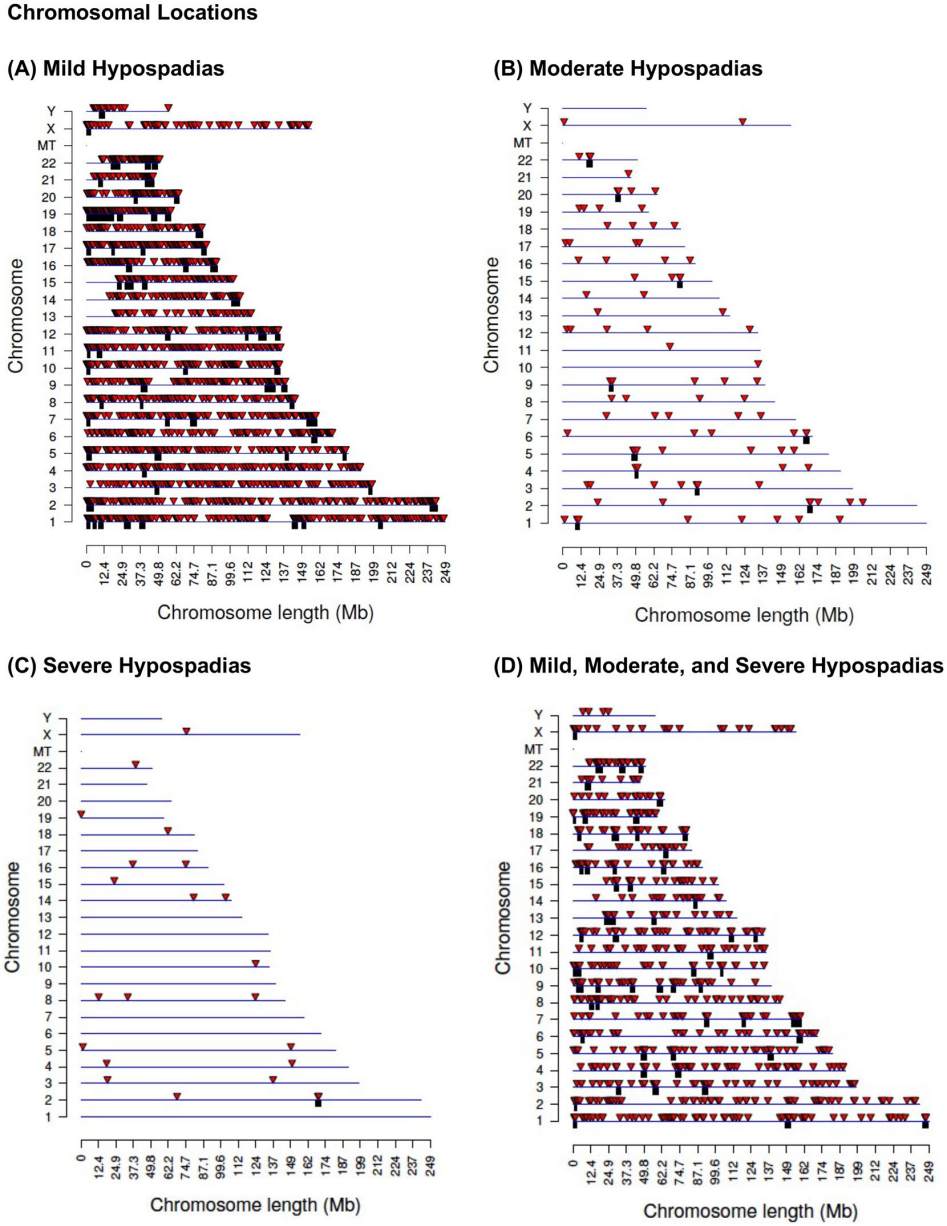
DMR chromosomal locations. The DMR locations on the individual chromosomes is represented with a red arrowhead and a cluster of DMRs with a black box. All DMRs containing at least one significant window at a p value threshold of 1e−05 for DMR are shown. (**A**) Mild (distal) hypospadias. (**B**) Moderate (mid shaft) hypospadias. (**C**) Severe (proximal) hypospadias. (**D**) Combined mild, moderate, and severe hypospadias.

DNA was isolated from small < 5 mm fragments of minced foreskin samples and DNA isolated and analyzed with a methylated DNA immunoprecipitation (MeDIP) procedure to obtain methylated DNA for subsequent sequencing (Seq) for an MeDIP-Seq protocol^38^, as described in the Methods. This procedure can provide a genome-wide analysis of greater than 95% of the genome^29^. Differential DNA methylation regions (DMRs) were identified by comparing the control and hypospadias case samples from each individual newborn male child in the separate groups. The DMRs were identified for each group and presented in Fig. 1. The control group was compared with each of the mild (distal), moderate (mid shaft), and severe (proximal) hypospadias groups to identify the DMRs. The DMRs at various p value statistical thresholds are presented, and p < 1e−05 (i.e., p < 0.00001) was used for all subsequent analyses. The number of DMR with adjacent DMR 1 kb windows are shown at a significance level of p < 1e−05. The majority of DMR for each group involved a single 1 kb window, with some DMRs with two adjacent 1 kb windows or more, Fig. 1. The mild hypospadias had 2725 DMRs at p < 1e−05 edgeR and a false discovery rate (FDR) p value less than 0.01, Fig. 1A. The moderate hypospadias had 95 DMRs at p < 1e−05 edgeR and a false discovery rate (FDR) p value less than 0.1, Fig. 1B. The severe hypospadias had 22 DMRs at p < 1e−05 edgeR and a false discovery rate (FDR) p value less than 0.1 for 15 DMR (Fig. 1C). A combination analysis of all mild, moderate, and severe hypospadias had 734 DMRs at p < 1e−05 edgeR and FDR p value less than 0.05 (Fig. 1D). The lists of DMRs for each group and genomic features are presented in Supplemental Table S2 for mild, Supplemental Table S3 for moderate, Supplemental Table S4 for severe, and Supplemental Table S5 for all hypospadias. These tables present for each group the DMR name, chromosomal locations, start position, DMR length in bp, log-fold change (for positive increased DNA methylation or for negative decreased DNA methylation) for each DMR, gene associations and functional categories for the associated genes, Supplemental Tables S2–S5. Approximately half of the DMRs had an increase in DNA methylation and the rest a decrease in DNA methylation. The chromosomal locations of the DMRs (red arrowheads) for each group are presented in Fig. 2. The DMRs are present on most chromosomes, with groups of DMRs throughout the genome. The black boxes indicate clusters of DMRs at particular regions (Fig. 2). No obvious common regions are observed between the groups. The genomic features of the DMRs were investigated. The CpG density of the DMRs for each group is predominantly between 1 and 3 CpG/100 bp, Supplemental Figure S1. These regions with low CpG density are considered CpG deserts^39^, which represents the majority (> 90%) of the genome^29^. The sizes of the DMRs for each group are predominantly 1 or 2 kb (Supplemental Figure S1).

**Figure 3:**
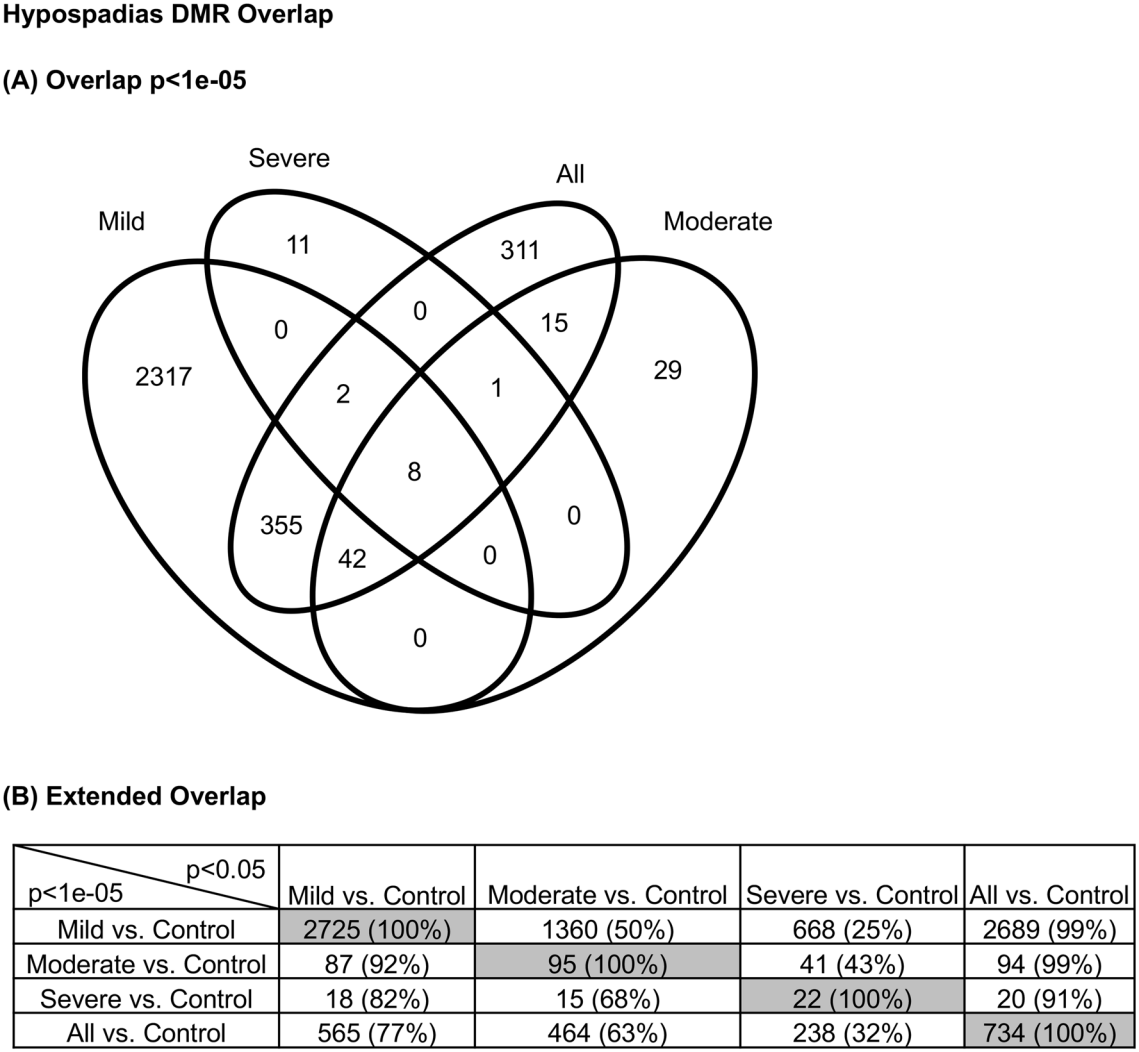
Hypospadias group DMR overlap. (**A**) Venn diagram. (**B**) Extended overlap p < 1e−05 compared to p < 0.05 comparison. The number of DMRs overlapped and percentage presented.

**Figure 4:**
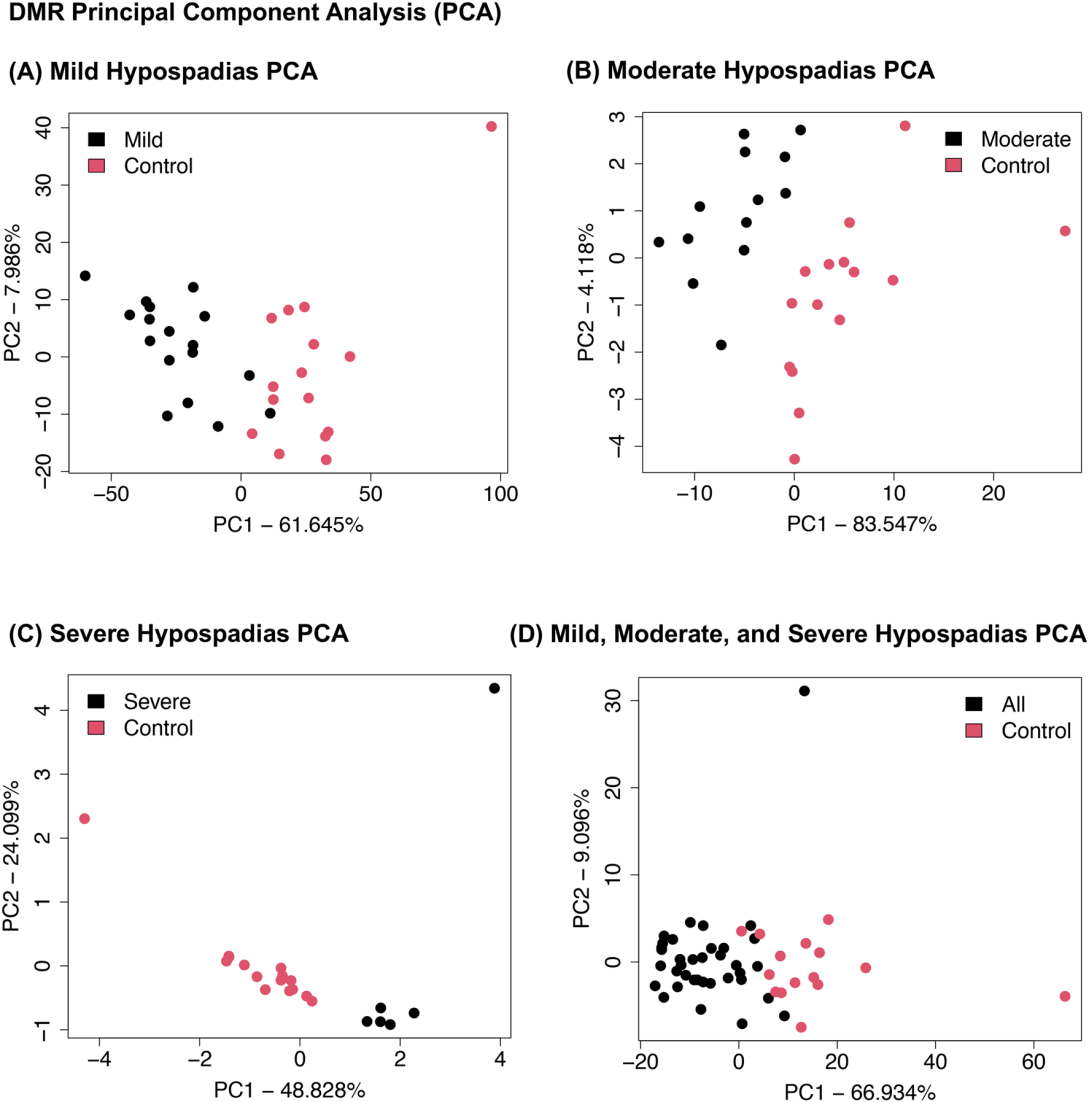
DMR principal component analysis (PCA). The first two principal components used. The underlying data is the RPKM read depth for DMR genomic windows. (**A**) Mild (distal) hypospadias, (**B**) Moderate hypospadias (moderate), (**C**) Severe (proximal) hypospadias, and (**D**) Combined mild, moderate, and severe hypospadias.

An overlap of the DMRs for each group demonstrated the majority of DMRs were distinct for each group except the All group, at p < 1e−05 (i.e., p < 0.00001) (Fig. 3A). As expected, the All hypospadias group had good overlap with the mild (distal) hypospadias and other groups (Fig. 3A). Further analysis of potential overlap used an extended overlap analysis with a comparison of the p < 1e−05 DMRs with the other groups at p < 0.05 threshold. The extended overlap demonstrated a much higher overlap with the All hypospadias group having greater than 90% overlap with the other groups and the mild hypospadias having 92% with moderate, 82% with severe and 77% with all (Fig. 3B). The severe (proximal) hypospadias had the lowest level of overlap with 25% with mild and 32% with all hypospadias. Therefore, a high DMR overlap between the hypospadias stages was observed with a reduced statistical threshold. A principal component analysis (PCA) of the DMR sets within each group demonstrated for all comparisons a good separation between the hypospadias and control group DMRs (Fig. 4). The DMR PCA demonstrate the control (non-disease) samples are distinct from the hypospadias samples at DMR sites.

**Figure 5:**
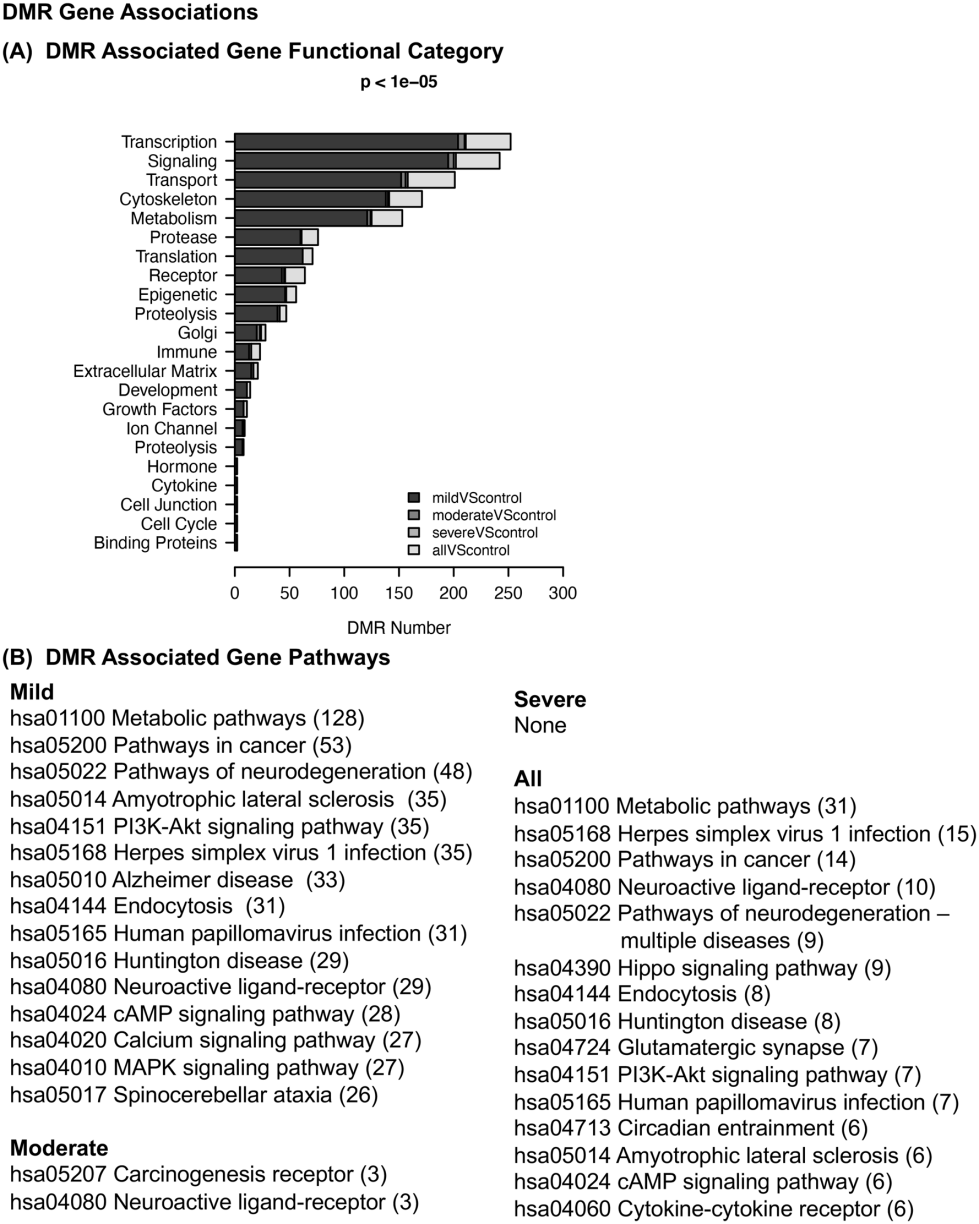
DMR gene associations. (**A**) the DMR associated gene functional categories are presented for mild, moderate, severe and combined all groups as indicated by the inset. The gene category and DMR number listed. (**B**) DMR associated gene pathways (KEGG) with KEGG identifier and number of pathway genes in brackets.

**Figure 6:**
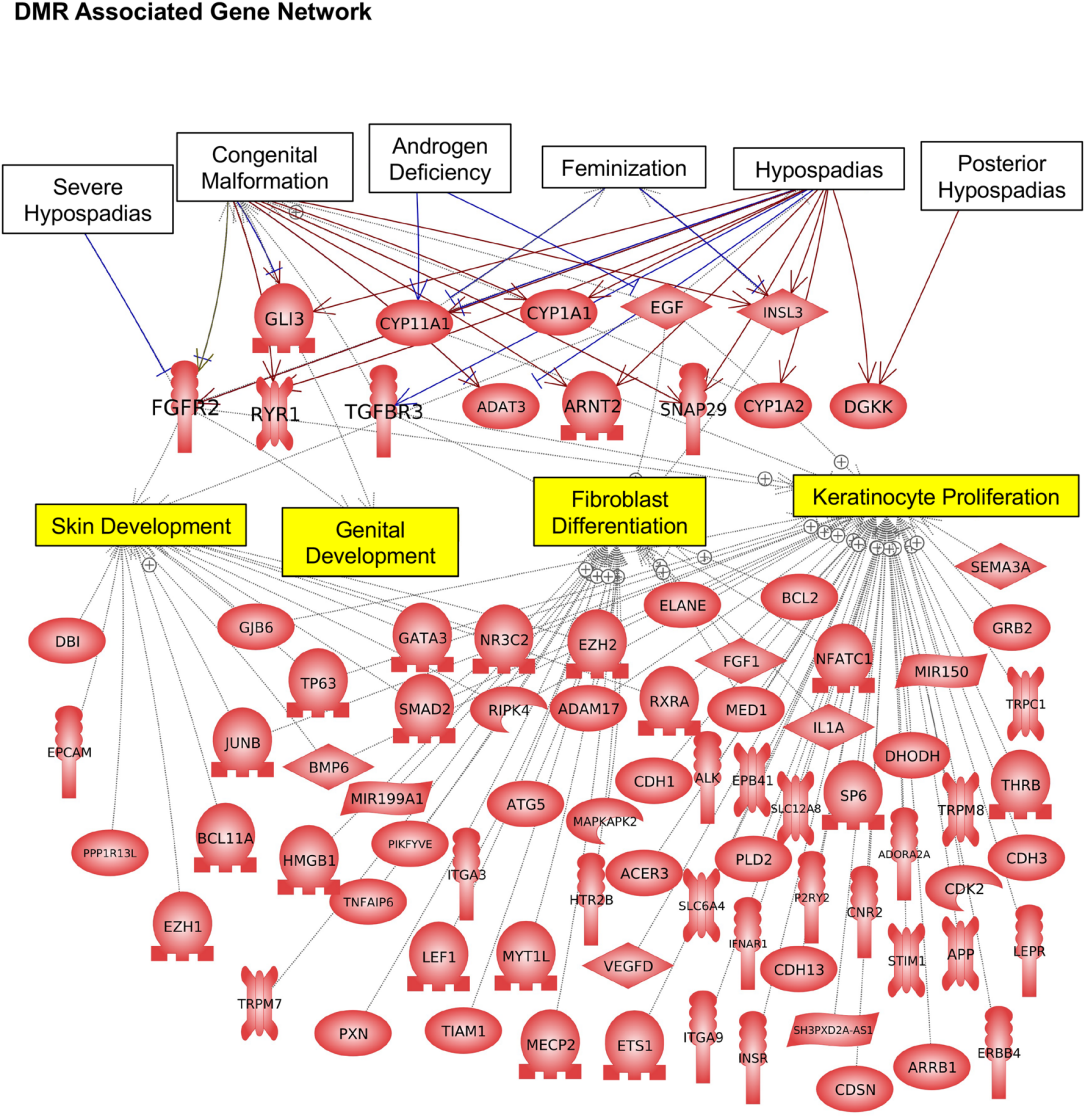
DMR associated gene networks. The hypospadias disease categories (white box) and related categories presented are correlated with DMR associated genes. The disease associated cellular processes (yellow box) are correlated with the known hypospadias associated genes, as well as DMR associated genes correlated to the cellular processes, but not known to previously be associated with hypospadias (lower set genes). Gene symbols and correlated names are presented in Supplemental Table S6.

The final analysis investigated the DMR associated genes for each of the hypospadias groups. The DMRs within 10 kb of a gene to include proximal and distal promoter regions were identified. The DMR gene association does not confirm a regulatory site for gene expression, and many distal DMRs not identified as associated with genes may also influence gene expression through ncRNA mediated mechanisms. The DMR associated genes listed in Supplemental Tables S2–S5 were analyzed for gene functional category (Fig. 5A). The transcription, signaling, and transport categories were prominent in each comparison group. The DMR associated gene groups were analyzed for pathway associations with ≥ 3 genes in the pathway for each comparison group (Fig. 5B). The mild hypospadias had the highest number of pathway associations, due to having the highest number of DMRs, followed by the all combined hypospadias, with metabolism, pathways in cancer, and neurodegeneration pathways being predominant. The moderate and severe hypospadias had too few DMR and associated genes for this analysis. The DMR associated genes in the mild hypospadias group were further analyzed with a gene network analysis with a focus on genes previously shown to be involved in hypospadias (Fig. 6). The moderate and severe hypospadias groups had too few genes for this analysis, but both did show links with CYP11A1 and CYP1A1 involved in steroidogenesis of androgen. The identified DMR associated genes were correlated with major diseases, including hypospadias, and cellular processes, using the Pathway Studio (Elsevier Inc.) analysis described in the Methods. Hypospadias, severe hypospadias, posterior hypospadias, congenital malformation, androgen deficiency, and feminization were diseases over-represented in the mild group of DMR associated genes. Skin development, genital development, fibroblast differentiation, and keratinocyte proliferation are cellular processes that had correlations with the hypospadias-disease-connected DMR associated genes (Fig. 6). Previously identified hypospadias associated genes are present, including GLI3, CYP11A1, CYP1A1, EGF, TGFBR3, FGFR2, RYR1, INSL3, ADAT3, ARNT2, SNAP29, CYPIA2, DGKK^9,10^. In addition to the previous known hypospadias associated disease categories, a large number of hypospadias DMR associated genes identified were correlated with the cellular processes presented (Fig. 6). The DMR associated genes and names in Fig. 6 are presented in Supplemental Table S6. These DMR associated genes have not been directly associated to hypospadias previously, but require consideration due to the current study observations.

## Discussion

Hypospadias is the most frequent malformation of the penis and the second most common birth defect in male newborns after cryptorchidism^40^, which has increased in frequency over the past decades by 11.5%^4^. This corresponds with an increase in all chronic disease, which now impacts over 80% of the human population worldwide^41,42^. As with many diseases, including hypospadias, environmental exposures are thought to be the primary source for this increase in chronic disease^41^. Although genetics and genetic mutations have been speculated to be the primary molecular basis for hypospadias^9,10^, as with most other disease, environmental factors seldom directly induce genetic mutations, so fall short of explaining the molecular basis of disease etiology. Hypospadias has been shown to be impacted by a number of environmental toxicants and factors, such as DES, that increase the frequency of the disease in the human population^5,15–17,25,26,30–32^. Factors such as DES do not have the capacity to impact DNA sequence (i.e., genetic mutations), but have been shown to promote epigenetic alterations^43,44^. Previous reports have speculated on the role of epigenetics in hypospadias, but few studies^2^ have directly assessed epigenetic alterations in tissues related to hypospadias. As with many pathologies, various degrees of hypospadias [i.e., mild (distal), moderate (mid shaft), severe (proximal)] reflect the magnitude of abnormal or disrupted development of the tissue. Little molecular information has been reported on the etiology of these different stages of the disease. Clinical treatments of the various severities of hypospadias are different, and the abnormal tissue development distinct^45,46^. The current study was designed to investigate the role of environmental epigenetics in hypospadias etiology, and to compare the mild (distal), moderate (mid shaft), and severe (proximal) hypospadias with regards to epigenetic alterations.

Abnormal penile development can result in hypospadias and involves the androgen responsive development of fibroblasts and foreskin^47^. Therefore, hypospadias etiology involves molecular alterations in the fibroblast of the foreskin to, in part, generate the hypospadias. Although genetic mutations have been shown to be involved, genome-wide association studies (GWAS) suggest less than ten percent of hypospadias patients have associated genetic mutations^11^. Transcriptome analyses of non-diseased and hypospadias foreskin have been reported and support the role of altered gene expression having a role in hypospadias^48,49^. The question is what regulates the altered transcriptome during the development of hypospadias. Epigenetics is now known to be the primary regulator of gene expression events, so is the precursor molecular process that promotes the gene transcription. The current study identified DNA methylation alterations between hypospadias and non-disease penile fibroblast and foreskin samples (Fig. 1). Interestingly, the mild (distal) hypospadias cases had the highest number of DMRs, followed by fewer DMR in the moderate hypospadias penile fibroblast, and a dramatically reduced number of DMRs in the severe (proximal) hypospadias. This contrasts with the intuitive expectation that the severe hypospadias would have the most dramatic impacts. In considering the current observations, several potential explanations can be made. The first is that the foreskin in cases of mild hypospadias contain cells that have been impacted by the developmental etiology of disease, so those cells are still present. In contrast, with the severe hypospadias those affected fibroblast populations are removed (i.e., agenesis), and those that remain are similar to the mature fibroblasts in the non-disease foreskin. Therefore, a transient transmission of the fibroblasts leads to a dramatic alteration in the number of DMRs and transcriptome for the mild hypospadias group, but the progression to severe removes this cell population, and the remaining fibroblasts appear similar to the surrounding shaft fibroblasts. The moderate (mid shaft) hypospadias cells are intermediate with some of the epigenetic altered fibroblasts remaining. This hypothesis, developed from the current study observations, is supported by the overlap of the DMRs between the mild, moderate, and severe hypospadias tissue. This suggests a loss (atresia) of foreskin fibroblasts with large numbers of DMRs in the severe (proximal) hypospadias compared to the mild hypospadias. An alternate interpretation is that the sample size for the severe hypospadias was less, so a lower statistical significance was observed. However, the combined mild, moderate, and severe hypospadias foreskin provided a significant number of DMRs that showed a very high percentage overlap in DMRs between the individual stages (Fig. 4). Therefore, the suggestion that severe (proximal) hypospadias may have a reduced number of the affected cell populations is a viable hypothesis proposed for further investigations.

The genomic features of the foreskin hypospadias fibroblasts were investigated. The chromosomal locations of the DMRs were throughout the genome with no major location over-represented. The DMRs were in low CpG density deserts^39^ and 1 or 2 kb in size. There was generally a good separation of principal components in the PCA analysis between the hypospadias and non-disease DMRs. This demonstrates the hypospadias DMR genomic features are distinct from those in the non-disease samples. The final analysis investigated the DMR gene associations and the disease relationships (Figs. 5, 6). Although the DMRs cannot be definitively correlated with gene expression, the genomic locations of the DMRs can be associated with genes in a manner similar to GWAS genetic mutations. The DMRs within 10 kb of a gene were associated with a gene to include proximal and distal promoter gene considerations. The DMR-associated gene categories were predominant in transcription and signaling, which are larger gene families in the genome and involved in molecular control of genome activity and cellular function. The associated gene pathways suggest metabolism and signaling. A number of the DMR-associated genes also demonstrate correlations with hypospadias disease and cellular processes previously identified (Fig. 6). Therefore, the experimental design of the current study and epigenetic analysis was partially validated through the GWAS and transcriptome identification of previously known hypospadias associated genes and processes. Novel observations identify a large number (i.e., 66) of new DMR-associated genes and three non-coding RNA potentially associated in hypospadias, which included DBI, EPCAM, PPP1R13L, EZH1, BCL11A, JUNB, GJB6, TP63, BMP6, HMGB1, TRPM7, GATA3, SMAD2, MIR199A1, PIKFYVE, TNFAIP6, PXN, NR3C2, RIPK4, ATG5, ITGA3, LEF1, TIAM1, MYT1L, EZH2, ADAM17, MAPKAPK2, HTR2B, VEGFD, MECP2, ETS1, ACER3, CDH1, RXRA, ELANE, ALK, SLC6A4, ITGA9, INSR, IFNAR1, PLD2, EPB41, MED1, FGF1, BCL2, NFATC1, IL1A, SLC12A8, P2RY2, CDH13, SH3PXD2A-AS1, CDSN, CNR2, SP6, SEMA3A, GRB2, MIR150, TRPC1, DHODH, TRPM8, THRB, ADORA2A, CDK2, CDH3, STIM1, APP, LEPR, ARRB1, ERBB4 (Fig. 6). The gene symbols and correlated gene names are presented in Supplemental Table S6. Novel hypospadias associated pathways and cellular processes were also identified (Figs. 5, 6). Therefore, in addition to the insights into the presence and role of epigenetics in hypospadias etiology, a large number of potential novel associated genes and cellular processes are also provided and need to be considered in hypospadias development.

The current study demonstrates a role for epigenetics in hypospadias etiology. Direct environmental exposures, such as DES, have been shown to alter epigenetics and critical gene expression events to generate the etiology of disease, both in the individual exposed and in subsequent generations^6^. Epigenetics has evolved to be responsive to the environment and allow phenotypic variation and adaptation to facilitate natural selection and evolution^21^. When environmental exposures are at critical developmental periods, they can reprogram the normal cellular differentiation to promote a susceptibility for disease later in life, such as hypospadias in the male fetus and newborn. In addition, direct exposure impacts on epigenetics in the germline (sperm or egg) can transmit epigenetic and transcriptome alterations to the subsequent generation embryonic stem cell that then alters the epigenome and transcriptome of all derived somatic cells in the individual^6^. This results in increased disease susceptibility later in life, or in the case of hypospadias, fetal penile development and newborn hypospadias. This generational phenomenon is termed “environmentally induced epigenetic transgenerational inheritance” and is a non-genetic form of inheritance that impacts the offspring phenotypes and health for generations to come^6^. In plants, worms, and flies, the phenomenon has been transmitted hundreds of generations, and in mammals minimally five generations^6^. This environmentally induced epigenetic transgenerational inheritance phenomenon now needs to be incorporated into studies of disease etiology and provides an explanation for the dramatic increase in chronic disease incidence in the human population^6^. Although epigenetic changes linked to aging have been observed^50,51^, this is not relevant to the current study as fetal development and early life periods like hypospadias will not have such aging impacts. In considering the etiology of hypospadias, the direct and transgenerational actions of the environment on hypospadias is required. Previous DES studies have suggested the potential epigenetic transgenerational inheritance of hypospadias^25,26^. Therefore, the parental and ancestral impacts on hypospadias etiology need to be investigated. Recently, transgenerational epigenetics of parental or ancestral exposures have been shown to be involved in parental transmitted autism and male infertility etiology^52,53^. Similar studies with hypospadias are required in the future and will provide further insights into environmentally induced hypospadias etiology.

Combined observations identify a better understanding of the etiology of hypospadias on a molecular level so as to integrate environmental impacts and clarify the severity of hypospadias progression. The following components of hypospadias etiology need to be considered: (1) Environmental factors are critical in the induction of hypospadias and will act through epigenetic alterations in the penile tissues during development; (2) although genetic factors will be important, environmental factors generally cannot directly alter DNA sequence and require a precursor epigenetic modification to influence the transcriptome; (3) the differential DNA methylation regions (DMRs) that develop alter gene expression events that associate with hypospadias etiology; (4) DMR associated genes and cellular processes previously associated with hypospadias etiology were identified, as well as an expanded number of potential novel DMR associated genes were identified that need to be integrated into hypospadias etiology; and (5) The progression of hypospadias from mild (distal), moderate (mid shaft), and severe (proximal) appears to involve a reduction in detected DNA methylation alterations that suggest the more severe forms may have lost (i.e., agenesis) the foreskin fibroblast and epithelial cells involved in the mild hypospadias stage. More thorough epigenetic analysis of hypospadias at various developmental stages, in response to known environmental exposures, and generational impacts will allow further advances in understanding the etiology of hypospadias.

## Methods

### Clinical sample collection and analysis

Indiana University (IU) Health Hospitals Riley Hospital for Children and Franciscan Health, Indianapolis, Indiana, USA provided samples for the current study. Informed consent and HIPAA authorization were obtained from the legal guardians of all minor age participants prior to the clinical sample collection. The study protocol was approved by the Indiana University Institutional Review Board (IRB) #1503167555. All research was performed in accordance with relevant guidelines and regulations. Following IRB approval foreskin samples were prospectively collected from 51 non-Hispanic white males less than 20 months of age who were undergoing a surgical repair for hypospadias (cases) or simple circumcision (controls). Of the 51 participants, 36 (70.6%) were cases and 15 (29.4%) were controls. Controls were mean age matched to cases with no statistical difference. The mean ± SD age for cases was 11 ± 3.8 months and for controls 13 ± 5.8 months was not significant (p < 0.068). Hypospadias cases were categorized by levels of severity by the Urologist in accordance with the medical and surgical procedure codes: n = 17 (47.2%) mild (distal), n = 13 (36.1%) moderate (mid shaft), n = 6 (16.7%) severe (proximal). The demographic data for these subjects is presented in Supplemental S1. Foreskin tissues were after collection stored at − 20 °C until shipment and then stored at − 80 °C.

### DNA preparation

Frozen human foreskin tissue samples were stored at − 80 °C and thawed for analysis. Genomic DNA from foreskin tissue samples was prepared as follows: the foreskin tissue was suspended in 750 μl of cell lysis solution and 3.5 µl of Proteinase K (20 mg/ml). This suspension was incubated at 55 °C for 3 h, then vortexed and centrifuged briefly. The lysis solution was then transferred to a new 1.5 µl microcentrifuge tube. The microcentrifuge tube with the foreskin tissue was centrifuged again to retain any remaining solution which was combined with the transferred lysis solution. The foreskin tissue had 300 µl of protein precipitation solution (Promega, A795A, Madison, WI) added to the lysis solution. The sample was incubated on ice for 15 min, then centrifuged at 4 °C for 30 min. The supernatant was transferred to a fresh 2 ml microcentrifuge tube and 1000 µl ice cold isopropanol was added along with 2 µl glycoblue. This suspension was mixed thoroughly and incubated at − 20 °C overnight. The suspension was then centrifuged at 4 °C for 20 min, the supernatant was discarded, and the pellet was washed with 75% ethanol, then air-dried and resuspended in 100 μl H_2_O. DNA concentration was measured using the Nanodrop (Thermo Fisher, Waltham, MA).

### Methylated DNA immunoprecipitation (MeDIP)

Methylated DNA Immunoprecipitation (MeDIP) with genomic DNA was performed as follows: individual DNA samples (2–4 μg of total DNA) were diluted to 130 μl with 1× Tris–EDTA (TE, 10 mM Tris, 1 mM EDTA) and sonicated with the Covaris M220 using the 300 bp setting. Fragment size was verified on a 2% E-gel agarose gel. The sonicated DNA was transferred from the Covaris tube to a 1.7 ml microfuge tube, and the volume was measured. The sonicated DNA was then diluted with TE buffer (10 mM Tris HCl, pH7.5; 1 mM EDTA) to 400 μl, heat-denatured for 10 min at 95 °C, then immediately cooled on ice for 10 min. Then 100 μl of 5× IP buffer and 5 μg of antibody (monoclonal mouse anti 5-methyl cytidine; Diagenode #C15200006) were added to the denatured sonicated DNA. The DNA-antibody mixture was incubated overnight on a rotator at 4 °C. The following day magnetic beads (Dynabeads M-280 Sheep anti-Mouse IgG; 11201D) were pre-washed as follows: the beads were resuspended in the vial, then the appropriate volume (50 μl per sample) was transferred to a microfuge tube. The same volume of Washing Buffer (at least 1 ml 1× PBS with 0.1% BSA and 2 mM EDTA) was added and the bead sample was resuspended. The tube was then placed into a magnetic rack for 1–2 min and the supernatant was discarded. The tube was removed from the magnetic rack and the beads were washed once. The washed beads were resuspended in the same volume of 1X IP buffer (50 mM sodium phosphate ph 7.0, 700 mM NaCl, 0.25% Triton X-100) as the initial volume of beads. 50 μl of beads were added to the 500 μl of DNA-antibody mixture from the overnight incubation, then incubated for 2 h on a rotator at 4 °C. After the incubation, the bead-antibody-DNA complex was washed three times with 1X IP buffer as follows: the tube was placed into a magnetic rack for 1–2 min and the supernatant was discarded, then the magnetic bead antibody pellet was washed with 1X IP buffer 3 times. The washed bead antibody DNA pellet was then resuspended in 250 μl digestion buffer with 3.5 μl Proteinase K (20 mg/ml). The sample was incubated for 2–3 h on a rotator at 55 °C, then 250 μl of buffered phenol–chloroform-isoamylalcohol solution was added to the sample, and the tube was vortexed for 30 s and then centrifuged at 14,000×*g* for 5 min at room temperature. The aqueous supernatant was carefully removed and transferred to a fresh microfuge tube. Then 250 μl chloroform were added to the supernatant from the previous step, vortexed for 30 s and centrifuged at 14,000×*g* for 5 min at room temperature. The aqueous supernatant was removed and transferred to a fresh microfuge tube. To the supernatant 2 μl of glycoblue (20 mg/ml), 20 μl of 5 M NaCl and 500 μl ethanol were added and mixed well, then precipitated in − 20 °C freezer for 1 h to overnight. The precipitate was centrifuged at 14,000×*g* for 20 min at 4 °C and the supernatant was removed, while not disturbing the pellet. The pellet was washed with 500 μl cold 70% ethanol in − 20 °C freezer for 15 min then centrifuged again at 14,000×*g* for 5 min at 4 °C and the supernatant was discarded. The tube was spun again briefly to collect residual ethanol to the bottom of the tube and as much liquid as possible was removed with gel loading tip. The pellet was air-dried at room temperature until it looked dry (about 5 min) then resuspended in 20 μl H_2_O or TE buffer. DNA concentration was measured in Qubit (Life Technologies) with ssDNA kit (Molecular Probes Q10212).

### MeDIP-Seq analysis

The MeDIP DNA samples (50 ng of each) were used to create libraries for next generation sequencing (NGS) using the NEBNext Ultra RNA Library Prep Kit for Illumina (San Diego, CA) starting at step 1.4 of the manufacturer’s protocol to generate double stranded DNA. After this step the manufacturer’s protocol was followed. Each sample received a separate index primer. NGS was performed at WSU Spokane Genomics Core using the Illumina HiSeq 2500 with a PE50 application, with a read size of approximately 50 bp and approximately 10–32 million reads per sample, and 10–11 sample libraries each were run in one lane.

### Molecular bioinformatics and statistics

Basic read quality was verified using information produced by the FastQC program^54^. Reads were filtered and trimmed to remove low quality base pairs using Trimmomatic^55^. The reads for each sample were mapped to the GRCh38 human genome using Bowtie2^56^ with default parameter options. The mapped read files were then converted to sorted BAM files using SAMtools^57^. To identify DMR, the reference genome was broken into 1000 bp windows. Duplicate reads were considered PCR artifacts and removed. The MEDIPS R package^58^ was used to calculate differential coverage between control and exposure sample groups using TMM normalization. Only genomic windows with an average read depth across all samples of at least 10 reads were analyzed for differential coverage. The edgeR p value (the exact p value for differential expression based on the negative binomial mode)^59^ was used to determine the relative difference between the two groups for each genomic window. FDR adjusted p values were also calculated. Windows with an edgeR p value less than 10^–5^ (i.e., p < 1e−05 or p < 0.00001) were considered DMRs. The DMR edges were extended until no genomic window with an edgeR p value less than 0.1 remained within 1000 bp of the DMR. CpG density and other information was then calculated for the DMR based on the reference genome. DMR were annotated using the NCBI provided annotations. The genes that overlapped with DMR were then input into the KEGG pathway search^60,61^ to identify associated pathways. The DMR associated genes were then sorted into functional groups by reducing Panther^62^ protein classifications into more general categories. All MeDIP-Seq genomic data obtained in the current study have been deposited in the NCBI public GEO database (GEO #: GSE200681). Lists of DMR associated genes were analyzed for functional relationships using the KEGG database (https://www.genome.jp/kegg/pathway.html) and Pathway Studio software (version 12.2.1.2: database of functional relationships and pathways of mammalian proteins; Elsevier).

### Ethics approval and consent to participate

Indiana University (IU) Health Hospitals Riley Hospital for Children and Franciscan Health, Indianapolis, Indiana, USA provided samples for the current study. Informed consent and HIPAA authorization were obtained from the legal guardians of all minor age participants and approved prior to the clinical sample collection. The study protocol was approved by the Indiana University Institutional Review Board (IRB) #1503167555. All research was performed in accordance with relevant guidelines and regulations.

## Supplementary Information

Supplementary Information.

## Data Availability

All molecular data have been deposited into the public database at NCBI (GEO # GSE200681), and R code computational tools are available at GitHub (https://github.com/skinnerlab/MeDIP-seq) and www.skinner.wsu.edu.

## Abbreviations

DMRs: Differential DNA methylation regions
DES: Diethylstilbestrol
MeDIP: Methylated DNA immunoprecipitation
NGS: Next generation sequencing
FDR: False discovery rate
PCA: Principal component analysis
Supplemental Table S6: Gene symbols and names
