# Supplementary material for: Role of epigenetics in the etiology of hypospadias through penile foreskin DNA methylation alterations
Source: Sci Rep. 2023 Jan 11;13:555. doi: 10.1038/s41598-023-27763-5 (PMC9834259; doi:10.1038/s41598-023-27763-5)
Supplement: Supplementary file 1 — Supplementary Information. [file 41598_2023_27763_MOESM1_ESM.pdf]

Role of Epigenetics in the Etiology of Hypospadias through Penile Foreskin DNA Methylation Alterations

Martin Kaefer, Richard Rink, Rosalia Misseri, Paul Winchester, Cathy Proctor, Millissia Ben Maamar, Daniel Beck, Eric Nilsson, and Michael K. Skinner

SUPPLEMENTAL MATERIAL

Supplemental Figure and Table Legends

Supplemental Figure S1. DMR genomic features. The number of DMRs at different CpG densities and length (kb). All DMRs at a p-value threshold of 1e-05 are shown. **(A)** Mild (distal) hypospadias DMR CpG density. **(B)** Mild hypospadias DMR length. **(C)** Moderate (mid shaft) hypospadias DMR CpG density. **(D)** Moderate hypospadias DMR length. **(E)** Severe (proximal) hypospadias DMR CpG density. **(F)** Severe hypospadias DMR length. **(G)** Mild, moderate, and severe hypospadias DMR CpG density. **(H)** Mild, moderate, and severe hypospadias DMR length.

Supplemental Table S1. Clinical sample information. Sample information (identification, collection site, age days and months, date collected, case, gender, and hypospadias severity.

Supplemental Table S2. DMR table for mild (distal) hypospadias versus control p<1e-05. DMR name, chromosome number, start nucleotide site, length (bp), p-value, maximum log fold change (LFC), CpG number and density, gene annotation, and gene category.

Supplemental Table S3. DMR table for moderate (mid shaft) hypospadias versus control p<1e-05. DMR name, chromosome number, start nucleotide site, length (bp), p-value, maximum log fold change (LFC), CpG number and density, gene annotation, and gene category.

Supplemental Table S4. DMR table for severe (proximal) hypospadias versus control p<1e-05. DMR name, chromosome number, start nucleotide site, length (bp), p-value, maximum log fold change (LFC), CpG number and density, gene annotation, and gene category.

Supplemental Table S5. DMR table for combined mild, moderate, and severe hypospadias versus control p<1e-05. DMR name, chromosome number, start nucleotide site, length (bp), p-value, maximum log fold change (LFC), CpG number and density, gene annotation, and gene category.

Supplemental Table S6. DMR associated gene symbols and names. The genes identified in Figure 6 and Discussion section are all listed with the correlated names.

Supplemental Figure S1

DMR Genomic Features

(A) Mild Hypospadias CpG Density

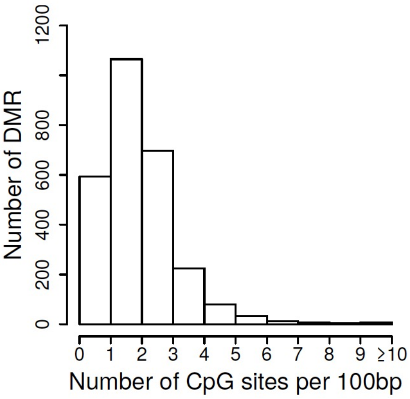

(B) Mild Hypospadias DMR Length

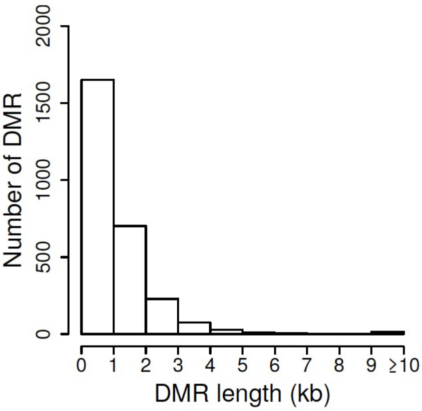

(C) Moderate Hypospadias CpG Density

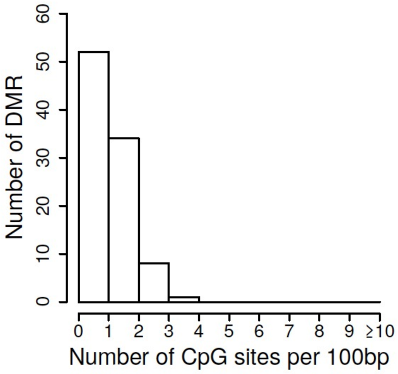

(D) Moderate Hypospadias DMR Length

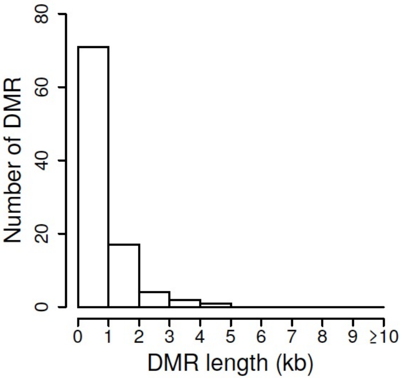

(E) Severe Hypospadias CpG Density

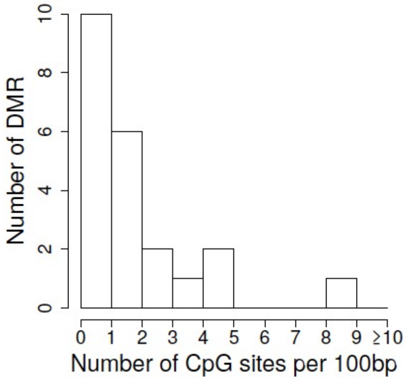

(F) Severe Hypospadias DMR Length

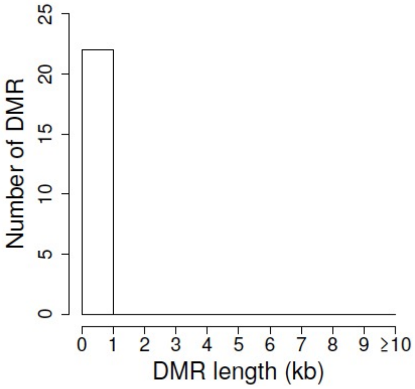

(G) All Hypospadias CpG Density

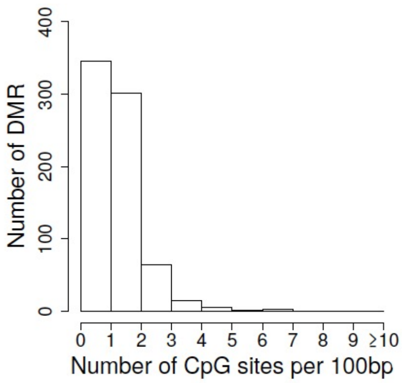

(H) All Hypospadias DMR Length

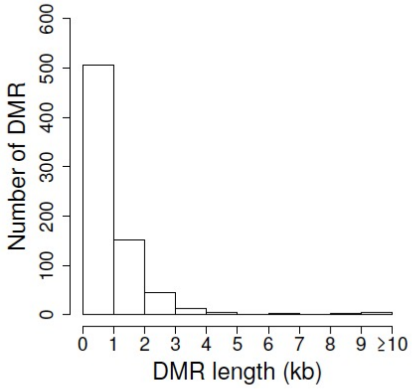

Supplemental Table S1
Clinical Sample Information

Case Sample ID	Sample ID	CASE Collection Site	CASE Age at Collection (days)	CASE Age at Collection (months)	CASE Race	CASE Gender	CASE Group	CASE Hypospadias Severity
HYPO IURIL FO 001	**HYM1**	Riley	262	8.7	Caucasian	male	Case	mild
HYPO IURIL FO 007	**HYM2**	Riley	307	10.2	Caucasian	male	Case	mild
HYPO IURIL FO 011	**HYM3**	Riley	269	9.0	Caucasian	male	Case	mild
HYPO IURIL FO 014	**HYM4**	Riley	257	8.6	Caucasian	male	Case	mild
HYPO IURIL FO 022	**HYM5**	Riley	395	13.2	Caucasian	male	Case	mild
HYPO IURIL FO 025	**HYM6**	Riley	462	15.4	Caucasian	male	Case	mild
HYPO IURIL FO 042	**HYM7**	Riley	189	6.3	Caucasian	male	Case	mild
HYPO IURIL FO 048	**HYM8**	Riley	244	8.1	Caucasian	male	Case	mild
HYPO IURIL FO 053	**HYM9**	Riley	236	7.9	Caucasian	male	Case	mild
HYPO IURIL FO 074	**HYM10**	Riley	450	15.0	Caucasian	male	Case	mild
HYPO IURIL FO 078	**HYM11**	Riley	701	23.4	Caucasian	male	Case	mild
HYPO IURIL FO 079	**HYM12**	Riley	224	7.5	Caucasian	male	Case	mild
HYPO IURIL FO 080	**HYM13**	Riley	217	7.2	Caucasian	male	Case	mild
HYPO IURIL FO 082	**HYM14**	Riley	383	12.8	Caucasian	male	Case	mild
HYPO SSFHS FO 002	**HYM15**	St. Francis	370	12.3	Caucasian	male	Case	mild
HYPO SSFHS FO 003	**HYM16**	St. Francis	367	12.2	Caucasian	male	Case	mild
HYPO SSFHS FO 004	**HYM17**	St. Francis	318	10.6	Caucasian	male	Case	mild
HYPO IURIL FO 002	**HYS18**	Riley	206	6.9	Caucasian	male	Case	moderate
HYPO IURIL FO 005	**HYS19**	Riley	219	7.3	Caucasian	male	Case	moderate
HYPO IURIL FO 008	**HYS20**	Riley	209	7.0	Caucasian	male	Case	moderate
HYPO IURIL FO 032	**HYS23**	Riley	449	15.0	Caucasian	male	Case	moderate
HYPO IURIL FO 034	**HYS24**	Riley	338	11.3	Caucasian	male	Case	moderate
HYPO IURIL FO 040	**HYS25**	Riley	600	20.0	Caucasian	male	Case	moderate
HYPO IURIL FO 044	**HYS26**	Riley	329	11.0	Caucasian	male	Case	moderate
HYPO IURIL FO 047	**HYS27**	Riley	253	8.4	Caucasian	male	Case	moderate
HYPO IURIL FO 052	**HYS29**	Riley	210	7.0	Caucasian	male	Case	moderate
HYPO IURIL FO 073	**HYS30**	Riley	317	10.6	Caucasian	male	Case	moderate
HYPO IURIL FO 081	**HYS31**	Riley	241	8.0	Caucasian	male	Case	moderate
HYPO IURIL FO 085	**HYS33**	Riley	346	11.5	Caucasian	male	Case	moderate
HYPO SSFHS FO 09	**HYS36**	St. Francis	290	9.7	Caucasian	male	Case	moderate
HYPO IURIL FO 009	**HYS21**	Riley	468	15.6	Caucasian	male	Case	severe
HYPO IURIL FO 010	**HYS22**	Riley	188	6.3	Caucasian	male	Case	severe
HYPO IURIL FO 051	**HYS28**	Riley	299	10.0	Caucasian	male	Case	severe
HYPO IURIL FO 084	**HYS32**	Riley	221	7.4	Caucasian	male	Case	severe
HYPO SSFHS FO 013 (prev 060)	**HYS35**	St. Francis	291	9.7	Caucasian	male	Case	severe
HYPO SSFHS FO 05	**HYS34**	St. Francis	283	9.4	Caucasian	male	Case	severe

CONTROL Sample ID	Sample ID	CONTROL Collection Site	CONTROL Age at Collection (days)	CONTROL Age at Collection (months)	CONTROL Race	CONTROL Gender	CASE Group	CASE Hypospadias Severity
HYPO SSFHS FO 07	**HYC49**	St. Francis	288	9.6	Caucasian	male	Control	none
HYPO IURIL FO 020	**HYC39**	Riley	321	10.7	Caucasian	male	Control	none
HYPO IURIL FO 050	**HYC43**	Riley	321	10.7	Caucasian	male	Control	none
HYPO IURIL FO 039	**HYC41**	Riley	283	9.4	Caucasian	male	Control	none
HYPO IURIL FO 049	**HYC42**	Riley	468	15.6	Caucasian	male	Control	none
HYPO SSFHS FO 06	**HYC48**	St. Francis	655	21.8	Caucasian	male	Control	none
HYPO IURIL FO 004	**HYC37**	Riley	207	6.9	Caucasian	male	Control	none
HYPO IURIL FO 028	**HYC40**	Riley	260	8.7	Caucasian	male	Control	none
HYPO IURIL FO 019	**HYC38**	Riley	260	8.7	Caucasian	male	Control	none
HYPO IURIL FO 083	**HYC45**	Riley	491	16.4	Caucasian	male	Control	none
HYPO IURIL FO 077	**HYC44**	Riley	877	29.2	Caucasian	male	Control	none
HYPO SSFHS FO 011	**HYC47**	St. Francis	386	12.9	Caucasian	male	Control	none
HYPO SSFHS FO 010	**HYC46**	St. Francis	386	12.9	Caucasian	male	Control	none
HYPO SSFHS FO 012	**HYC50**	St. Francis	368	12.3	Caucasian	male	Control	none
HYPO SSFHS FO 014	**HYC51**	St. Francis	339	11.3	Caucasian	male	Control	none

Supplemental Table S1. Clinical sample information. Sample information (identification, collection site, age days and months, date collected, case, gender, and hypospadias severity.

Supplemental Table S2
DMR Table Mild versus Control p<1e-05

DMR Name	Chr	Start	Length	# Sig Win	minP	maxLFC	CpG #	CpG Density	Gene Annotation	Gene Category
DMR1:788001	1	788001	4000	1	6.77E-08	-0.4130552	78	1.95	LOC100288069;LINC01409	
DMR1:820001	1	820001	4000	2	4.59E-11	0.8573156	37	0.925	FAM87B;LINC00115;LINC01128	
DMR1:875001	1	875001	2000	1	2.50E-07	-0.7297566	43	2.15	LOC107984850;FAM41C;TUBB8P11	
DMR1:1362001	1	1362001	6000	1	1.23E-06	-0.582795	129	2.15	MXRA8;AURKAIP1	
DMR1:1720001	1	1720001	2000	1	1.32E-09	-0.6682232	49	2.45	CDK11A;SLC35E2A	Signaling;Transport
DMR1:1783001	1	1783001	2000	1	9.36E-06	-0.6853059	46	2.3	NADK;GNB1	Signaling;Signaling
DMR1:2217001	1	2217001	1000	1	6.48E-06	-0.5196744	29	2.9	FAAP20;LOC105378593	
DMR1:2896001	1	2896001	1000	1	4.64E-08	0.6786443	18	1.8		
DMR1:3692001	1	3692001	3000	1	9.78E-06	-0.6085598	76	2.533333333	TP73	Transcription
DMR1:3929001	1	3929001	2000	1	8.26E-06	-0.3546132	23	1.15	LINC01346	
DMR1:4302001	1	4302001	2000	1	6.98E-06	-0.4819832	38	1.9		
DMR1:4780001	1	4780001	1000	1	7.25E-06	-0.5327415	21	2.1	AJAP1	
DMR1:4939001	1	4939001	1000	1	1.12E-06	0.7579953	9	0.9		
DMR1:5480001	1	5480001	2000	1	9.70E-06	0.6609307	48	2.4	LOC107984911;LOC105376686	
DMR1:5883001	1	5883001	3000	1	2.97E-06	-0.4366861	99	3.3	NPHP4	
DMR1:6012001	1	6012001	1000	1	3.64E-06	-0.6013789	15	1.5	KCNAB2	
DMR1:6651001	1	6651001	1000	1	9.81E-08	0.7985146	20	2	DNAJC11	Transcription
DMR1:6703001	1	6703001	1000	1	8.06E-06	-0.5472783	27	2.7	DNAJC11	Transcription
DMR1:9122001	1	9122001	2000	1	9.36E-08	-1.6059539	22	1.1	GPR157	Signaling
DMR1:9477001	1	9477001	3000	1	5.62E-07	0.7773504	52	1.733333333		
DMR1:9642001	1	9642001	2000	1	5.29E-06	-0.5775419	33	1.65	PIK3CD;PIK3CD-AS1	Signaling
DMR1:9856001	1	9856001	2000	1	6.73E-06	-0.7320641	48	2.4	CTNNBIP1;LOC105376717	
DMR1:10035001	1	10035001	1000	1	4.70E-06	-0.3699255	52	5.2	UBE4B	Proteolysis
DMR1:10199001	1	10199001	2000	1	5.67E-07	-0.6703284	33	1.65	KIF1B	Cytoskeleton
DMR1:10577001	1	10577001	1000	1	9.03E-07	-0.7456126	14	1.4	PEX14	Transport
DMR1:10715001	1	10715001	2000	1	1.56E-06	-0.4669767	48	2.4	CASZ1	Transcription
DMR1:11373001	1	11373001	2000	1	2.43E-07	-0.489997	18	0.9	LOC105376739;LOC105376740	
DMR1:11826001	1	11826001	3000	1	3.21E-07	0.731116	75	2.5	CLCN6	Transport
DMR1:11834001	1	11834001	3000	1	3.96E-07	0.7464084	79	2.633333333	CLCN6;NPPA-AS1;NPPA	Transport
DMR1:14417001	1	14417001	2000	1	2.37E-07	-0.8633298	25	1.25	KAZN;KAZN-AS1	
DMR1:15381001	1	15381001	2000	1	1.81E-06	0.6524264	49	2.45	FHAD1;LOC101927441	
DMR1:15457001	1	15457001	2000	1	7.93E-08	-0.7165132	24	1.2	CTRC;CELA2A;LOC105376767	Protease
DMR1:16323001	1	16323001	1000	1	1.72E-07	-0.6218969	20	2	FBXO42	
DMR1:16870001	1	16870001	1000	1	6.10E-06	0.655432	21	2.1	TRV-CAC11-2;TRG-CCC1-2;LOC112267871;TRE-TTC3-1;RNU1-5P;TRN-GTT13-1	
DMR1:17412001	1	17412001	1000	1	4.18E-08	1.1205713	17	1.7	RCC2;RCC2-AS1	
DMR1:17450001	1	17450001	1000	1	2.58E-06	-0.698476	14	1.4		
DMR1:17484001	1	17484001	1000	1	1.18E-06	-0.6583338	21	2.1		
DMR1:20512001	1	20512001	1000	1	4.45E-06	-0.8451593	13	1.3	MUL1	
DMR1:20626001	1	20626001	1000	1	7.24E-06	-0.7269188	2	0.2	CDA;PINK1;MIR6084	Metabolism;Signaling
DMR1:21191001	1	21191001	1000	1	1.70E-06	-0.6021961	26	2.6		
DMR1:22576001	1	22576001	5000	1	2.83E-06	0.8832491	134	2.68	EPHA8	Receptor
DMR1:23677001	1	23677001	1000	1	1.84E-06	-0.8919592	14	1.4	LOC729856	
DMR1:23721001	1	23721001	2000	1	1.85E-06	-0.7056119	36	1.8		
DMR1:23910001	1	23910001	1000	1	7.68E-06	-0.5658299	14	1.4	CNR2;BTBD6P1;LOC107984929	Signaling
DMR1:25608001	1	25608001	1000	1	3.63E-10	-0.9167793	26	2.6	MAN1C1	Golgi
DMR1:26925001	1	26925001	1000	1	3.48E-06	-0.5918507	31	3.1	NUDC	Cytoskeleton
DMR1:27107001	1	27107001	2000	1	9.79E-07	-0.5064965	47	2.35	SLC9A1	Transport
DMR1:27160001	1	27160001	2000	1	9.32E-08	-0.6316421	53	2.65	SLC9A1;LOC102723760	Transport
DMR1:27187001	1	27187001	3000	1	9.95E-06	-0.6226966	58	1.933333333	RPL18AP5	
DMR1:27757001	1	27757001	1000	1	4.27E-07	-0.851594	18	1.8	FAM76A	
DMR1:27959001	1	27959001	1000	1	1.36E-06	-0.56006	71	7.1	SMPDL3B;XKR8	Signaling
DMR1:28295001	1	28295001	1000	1	3.30E-07	-0.6286917	26	2.6		
DMR1:28379001	1	28379001	4000	1	3.16E-06	-0.6114148	102	2.55	PHACTR4	Signaling
DMR1:28533001	1	28533001	1000	1	3.51E-06	-0.5057147	34	3.4	RCC1;PRDX3P2	
DMR1:28678001	1	28678001	1000	1	4.08E-06	-0.5323588	30	3	GMEB1	
DMR1:29067001	1	29067001	1000	1	1.24E-07	-0.6368667	27	2.7	EPB41	
DMR1:29132001	1	29132001	2000	1	2.01E-06	-0.4807419	41	2.05	TMEM200B	
DMR1:29187001	1	29187001	1000	1	4.99E-06	-0.6978736	25	2.5	SRSF4;MECR	Translation;Metabolism
DMR1:29536001	1	29536001	1000	1	1.92E-06	-0.9637771	7	0.7		
DMR1:31194001	1	31194001	4000	1	3.60E-06	-0.4544855	87	2.175	NKAIN1	
DMR1:32738001	1	32738001	2000	1	7.95E-07	-0.6790889	59	2.95	KIAA1522	

DMR1:32865001	1	32865001	2000	1	1.38E-07	-0.6477234	24	1.2	S100PBP;FNDC5;LOC105378631	
DMR1:35161001	1	35161001	1000	1	2.32E-06	-1.0339092	20	2		
DMR1:36191001	1	36191001	1000	1	8.13E-08	-0.6528079	20	2	RN7SL131P	
DMR1:37801001	1	37801001	1000	1	4.78E-09	-0.7641486	22	2.2	MANEAL;YRDC;C1orf122;MTF1	Transcription
DMR1:37861001	1	37861001	3000	1	1.87E-06	-0.4153095	64	2.133333333	MTF1;INPP5B	Transcription;Signaling
DMR1:37950001	1	37950001	1000	1	9.76E-07	-0.6710676	25	2.5	INPP5B;LOC105378651;SF3A3	Signaling;Translation
DMR1:38562001	1	38562001	1000	1	7.62E-06	-0.7294085	9	0.9		
DMR1:38775001	1	38775001	1000	1	1.90E-06	0.7113981	16	1.6		
DMR1:38970001	1	38970001	2000	1	5.14E-06	-0.6139955	42	2.1		
DMR1:38983001	1	38983001	3000	2	1.58E-07	-0.6436254	60	2	AKIRIN1	
DMR1:40022001	1	40022001	1000	1	2.30E-06	-0.5787969	33	3.3	LOC105378669	
DMR1:40185001	1	40185001	1000	1	6.42E-09	-0.8800793	21	2.1	RLF;RNU6-1237P	Transcription
DMR1:40456001	1	40456001	1000	1	9.98E-09	0.8518258	9	0.9	ZFP69B	Transcription
DMR1:41328001	1	41328001	2000	1	4.92E-06	0.8221739	17	0.85		
DMR1:41526001	1	41526001	4000	2	2.10E-09	-0.5508453	47	1.175	HIVEP3	
DMR1:42786001	1	42786001	2000	1	8.06E-06	-0.5018362	38	1.9	C1orf50;TMEM269-DT;TMEM269;LOC107984946	
DMR1:43851001	1	43851001	1000	1	8.01E-06	0.7309698	24	2.4	ST3GAL3;SHMT1P1	Transport
DMR1:44547001	1	44547001	2000	1	8.21E-09	0.4949184	26	1.3	RNF220;MIR5584	
DMR1:44742001	1	44742001	1000	1	3.10E-07	-0.7871357	21	2.1	KIF2C	Cytoskeleton
DMR1:45811001	1	45811001	1000	1	3.94E-07	-0.5405099	46	4.6	MAST2	Signaling
DMR1:48547001	1	48547001	2000	1	3.67E-07	0.6685656	17	0.85	AGBL4	Protease
DMR1:52797001	1	52797001	1000	1	2.78E-07	-0.9095082	17	1.7	ZYG11B;RNU6-969P	
DMR1:53030001	1	53030001	1000	1	2.35E-08	-0.9048279	13	1.3	SCP2	Transport
DMR1:54119001	1	54119001	3000	1	6.67E-09	-0.4598972	86	2.866666667	TCEANC2	Transcription
DMR1:54491001	1	54491001	2000	1	1.16E-06	-0.5621181	41	2.05		
DMR1:54550001	1	54550001	3000	1	7.35E-06	-0.6883699	37	1.233333333	ACOT11	Metabolism
DMR1:54909001	1	54909001	2000	1	1.79E-06	0.6115232	25	1.25		
DMR1:57207001	1	57207001	1000	1	2.79E-06	-0.6004718	28	2.8	DAB1	Cytoskeleton
DMR1:61925001	1	61925001	1000	1	3.78E-07	-0.6609481	17	1.7	PATJ	
DMR1:62302001	1	62302001	1000	1	9.00E-06	0.7022344	10	1	KANK4;RNU6-371P	Cytoskeleton
DMR1:62620001	1	62620001	1000	1	1.10E-06	-0.6070627	21	2.1	DOCK7	Transcription
DMR1:62783001	1	62783001	1000	1	4.75E-06	-0.5865481	47	4.7	ATG4C	Protease
DMR1:63422001	1	63422001	1000	1	6.76E-06	-0.8805903	7	0.7	ALG6	Golgi
DMR1:65530001	1	65530001	1000	1	1.09E-06	0.7953243	8	0.8	LEPR	Receptor
DMR1:70167001	1	70167001	1000	1	5.96E-06	-0.7442033	8	0.8	LRRC40	Cytoskeleton
DMR1:70480001	1	70480001	1000	1	3.71E-06	-0.726426	28	2.8		
DMR1:74500001	1	74500001	1000	1	9.31E-06	-0.570573	19	1.9	FPGT-TNNI3K;TNNI3K;LRRC53	
DMR1:74740001	1	74740001	3000	1	3.08E-06	0.7254702	45	1.5	CRYZ;TYW3	Metabolism;Epigenetic
DMR1:84939001	1	84939001	2000	1	9.48E-06	0.718078	34	1.7	MCOLN2	Transport
DMR1:88457001	1	88457001	1000	1	1.09E-06	-0.4941981	7	0.7	LOC105378839	
DMR1:90686001	1	90686001	1000	1	1.83E-06	-0.6240863	23	2.3		
DMR1:91873001	1	91873001	1000	1	2.16E-06	-0.8993481	10	1	TGFBR3	Receptor
DMR1:92121001	1	92121001	2000	1	9.94E-07	-0.7432738	23	1.15	BTBD8;GAPDHP46;PRKAR1AP1	
DMR1:92302001	1	92302001	1000	1	2.89E-06	-0.6520538	27	2.7	GLMN;RPAP2	Proteolysis
DMR1:94960001	1	94960001	2000	1	5.86E-06	-0.7950802	21	1.05	CNN3-DT	
DMR1:98215001	1	98215001	1000	1	9.97E-06	-0.4407812	18	1.8	LINC01776	
DMR1:99306001	1	99306001	1000	1	1.14E-06	0.7706012	27	2.7	PLPPR4	Signaling
DMR1:102225001	1	102225001	1000	1	1.91E-06	0.6391313	8	0.8		
DMR1:104977001	1	104977001	1000	1	2.18E-06	-0.8829162	10	1		
DMR1:105013001	1	105013001	1000	1	1.47E-07	-1.0447351	10	1	LOC105378880	
DMR1:105707001	1	105707001	1000	1	2.08E-09	-0.9358129	9	0.9	SEPTIN2P1	
DMR1:106483001	1	106483001	1000	1	8.05E-06	0.774615	6	0.6	LOC105378887	
DMR1:106803001	1	106803001	3000	1	3.72E-06	0.5010205	39	1.3	MTCO3P14;MTATP6P14;MTCO2P14;MTCO1P14	
DMR1:107012001	1	107012001	2000	1	6.10E-06	-0.6254179	37	1.85		
DMR1:109004001	1	109004001	2000	1	1.31E-06	-0.5852991	45	2.25	WDR47	
DMR1:109054001	1	109054001	2000	1	9.24E-06	-0.5414973	54	2.7	RANP5;TAF13	Transcription
DMR1:109087001	1	109087001	2000	1	6.08E-06	-0.7400489	45	2.25	TMEM167B-DT;TMEM167B;NDUFB3P1	
DMR1:110956001	1	110956001	1000	1	2.55E-08	0.6835196	6	0.6	LRIF1	
DMR1:111740001	1	111740001	2000	2	7.21E-12	-0.9845512	78	3.9	INKA2;INKA2-AS1;LOC101928718	
DMR1:112214001	1	112214001	1000	1	2.30E-06	0.7196884	8	0.8		
DMR1:112425001	1	112425001	1000	1	3.68E-06	-0.8007383	18	1.8	CTTNBP2NL	
DMR1:114098001	1	114098001	1000	1	9.84E-06	0.5904207	10	1	SYT6	Transport
DMR1:114205001	1	114205001	1000	1	7.44E-07	-0.6884116	10	1	LOC107985443	
DMR1:115641001	1	115641001	1000	1	6.49E-07	-0.6149139	46	4.6	VANGL1	
DMR1:115900001	1	115900001	1000	1	6.98E-06	-0.7084427	15	1.5		

DMR1:120310001	1	120310001	1000	1	1.00E-05	-0.5818533	32	3.2	LOC100996723	
DMR1:121396001	1	121396001	2000	1	1.75E-07	-0.8028891	166	8.3	SRGAP2C;SRGAP2-AS1;LOC107985107;LINC02798	Signaling
DMR1:122462001	1	122462001	1000	1	5.19E-07	-0.5142102	20	2		
DMR1:122496001	1	122496001	1000	1	5.44E-06	-0.5676477	21	2.1		
DMR1:122720001	1	122720001	1000	1	7.64E-06	-1.3585514	24	2.4		
DMR1:123362001	1	123362001	1000	1	2.90E-06	-1.0051942	21	2.1		
DMR1:124044001	1	124044001	3000	1	1.43E-06	-1.134609	57	1.9		
DMR1:124250001	1	124250001	1000	1	7.91E-06	-0.9769802	20	2		
DMR1:125085001	1	125085001	3000	1	2.92E-06	-0.6033661	327	10.9		
DMR1:125166001	1	125166001	6000	1	8.17E-07	-0.5240823	236	3.933333333		
DMR1:143497001	1	143497001	2000	2	3.52E-10	-0.8620085	224	11.2	NKAIN1P1;LOC101927452;LINC02799	
DMR1:143696001	1	143696001	1000	1	1.28E-07	-0.9966603	20	2	TRQ-CTG4-2;RNVU1-17	
DMR1:143920001	1	143920001	1000	1	3.86E-06	-0.6364698	25	2.5	LOC105371209;RPL22P5	
DMR1:143966001	1	143966001	2000	1	7.70E-06	-0.6712978	28	1.4	FAM72C;SRGAP2D	
DMR1:144322001	1	144322001	2000	2	2.89E-10	-1.090874	173	8.65	CH17-125A10.2;LOC105371196	
DMR1:144440001	1	144440001	1000	1	1.58E-06	-0.7051428	22	2.2	NBPF15;PFN1P6	
DMR1:144642001	1	144642001	2000	2	4.09E-08	-0.85842	200	10	LINC01632;LOC105371214;LOC105371215	
DMR1:144845001	1	144845001	3000	1	2.16E-08	-0.6263075	53	1.766666667	LOC105371217	
DMR1:145252001	1	145252001	2000	1	6.28E-06	-0.810855	9	0.45	PPIAL4D	Transcription
DMR1:146157001	1	146157001	3000	1	9.13E-06	0.6021301	47	1.566666667	NOTCH2NLA	
DMR1:146252001	1	146252001	1000	1	1.47E-06	-0.5873308	34	3.4		
DMR1:146808001	1	146808001	2000	1	4.36E-09	0.7957034	21	1.05	HYDIN2	
DMR1:147293001	1	147293001	1000	1	8.90E-06	0.8058247	16	1.6	CHD1L	
DMR1:148155001	1	148155001	1000	1	4.91E-06	-0.7582473	6	0.6	NBPF11;ABHD17AP1;LOC105371227;LINC02805	
DMR1:148520001	1	148520001	1000	1	8.74E-06	-0.6832805	7	0.7	TRN-GTT2-1	
DMR1:149321001	1	149321001	2000	1	7.35E-06	-0.6258372	66	3.3		
DMR1:149825001	1	149825001	1000	1	1.66E-07	-0.6775687	22	2.2	H4C14	
DMR1:150246001	1	150246001	3000	1	3.95E-09	-0.7882686	65	2.166666667	ANP32E;RNU2-17P;CA14	Epigenetic
DMR1:150480001	1	150480001	3000	1	6.31E-09	-0.5816525	63	2.1	RPRD2;TARS2;MIR6878	Signaling;Translation
DMR1:150486001	1	150486001	1000	1	3.09E-06	-0.6447183	29	2.9	RPRD2;TARS2;MIR6878	Signaling;Translation
DMR1:150583001	1	150583001	3000	2	4.08E-08	-0.6779094	73	2.433333333	ADAMTSL4-AS1;MCL1;LOC107985203	
DMR1:151251001	1	151251001	1000	1	2.65E-06	-0.7171751	19	1.9	PIP5K1A;PSMD4	Signaling;Protease
DMR1:151321001	1	151321001	1000	1	3.05E-06	-0.7817479	13	1.3	PI4KB	Signaling
DMR1:151497001	1	151497001	1000	1	1.15E-08	-0.7376456	32	3.2		
DMR1:153316001	1	153316001	1000	1	2.27E-06	0.8196115	8	0.8	PGLYRP3	
DMR1:153987001	1	153987001	3000	1	3.07E-06	-0.4286099	139	4.633333333	JTB;RAB13;RPS27;NUP210L;RPL34P5	Translation;Transport
DMR1:154189001	1	154189001	1000	1	1.53E-08	-0.6673729	20	2	TPM3;MIR190B;C1orf189	Cytoskeleton
DMR1:154634001	1	154634001	1000	1	7.05E-07	-0.6476199	22	2.2	ADAR	Metabolism
DMR1:154714001	1	154714001	1000	1	9.38E-06	-0.4393997	6	0.6	KCNN3	Transport
DMR1:154754001	1	154754001	1000	1	1.12E-06	0.5827178	18	1.8	KCNN3	Transport
DMR1:155992001	1	155992001	2000	1	3.66E-06	-0.3997333	44	2.2	ARHGEF2;ARHGEF2-AS2;ARHGEF2-AS1;LOC107985208	
DMR1:157592001	1	157592001	1000	1	1.91E-06	-0.9529406	4	0.4	FCRL4	Immune
DMR1:161725001	1	161725001	1000	1	9.22E-09	-0.7253335	30	3	FCRLB;RN7SL466P	Immune
DMR1:166878001	1	166878001	1000	1	1.63E-10	0.7776063	24	2.4	TADA1;DUTP6	
DMR1:169026001	1	169026001	1000	1	7.05E-06	-0.7779113	18	1.8	LINC00970	
DMR1:171687001	1	171687001	1000	1	1.04E-06	-0.7778769	12	1.2	RPL4P3	
DMR1:172290001	1	172290001	2000	1	8.44E-08	0.7264141	14	0.7	DNM3	Transport
DMR1:174863001	1	174863001	1000	1	7.81E-06	-0.5298797	25	2.5	RABGAP1L	Signaling
DMR1:175843001	1	175843001	1000	1	3.24E-06	0.5659879	8	0.8		
DMR1:177351001	1	177351001	2000	1	1.63E-06	-0.5100378	14	0.7	LINC01645	
DMR1:177598001	1	177598001	2000	1	3.82E-06	-0.7063309	32	1.6		
DMR1:179239001	1	179239001	3000	1	2.66E-06	-0.6157041	52	1.733333333	ABL2	
DMR1:182586001	1	182586001	1000	1	7.65E-07	0.6998463	20	2	RNASEL	
DMR1:184110001	1	184110001	2000	1	9.88E-06	-0.5236307	33	1.65	LOC102724830	
DMR1:187851001	1	187851001	1000	1	1.49E-06	0.8619744	13	1.3		
DMR1:189425001	1	189425001	1000	1	1.35E-06	-0.8126658	4	0.4	LOC105371657	
DMR1:189562001	1	189562001	1000	1	2.10E-21	-3.1174749	0	0	LOC105371657	
DMR1:190787001	1	190787001	1000	1	5.47E-07	0.6779403	7	0.7	LINC01720	
DMR1:197419001	1	197419001	1000	1	1.66E-07	-0.7870552	17	1.7	CRB1	Cytoskeleton
DMR1:200487001	1	200487001	1000	1	6.15E-07	-0.6083467	20	2		
DMR1:200653001	1	200653001	1000	1	5.70E-07	0.4903677	13	1.3	DDX59	
DMR1:201684001	1	201684001	1000	1	1.39E-06	-0.6448649	19	1.9	NAV1;IPO9-AS1	
DMR1:202905001	1	202905001	2000	1	5.77E-06	-0.5962143	39	1.95	KLHL12;HNRNPA1P59	Cytoskeleton
DMR1:202927001	1	202927001	3000	1	2.12E-07	-0.7599619	136	4.533333333	KLHL12	Cytoskeleton

DMR1:203526001	1	203526001	2000	1	8.26E-07	-0.4644113	21	1.05		
DMR1:203596001	1	203596001	1000	1	1.06E-08	-0.6493948	27	2.7		
DMR1:204270001	1	204270001	1000	1	1.64E-06	0.5923421	10	1	PLEKHA6	
DMR1:204315001	1	204315001	2000	1	9.32E-06	0.7338159	37	1.85	PLEKHA6	
DMR1:204472001	1	204472001	1000	1	6.76E-06	-0.5209533	28	2.8	PIK3C2B	Signaling
DMR1:204523001	1	204523001	1000	1	4.29E-06	-0.6177461	26	2.6	MDM4;LOC100291628	Epigenetic
DMR1:205117001	1	205117001	1000	1	5.75E-06	-0.7290055	17	1.7	RBBP5	
DMR1:206612001	1	206612001	2000	1	6.45E-06	-0.8107227	80	4	EIF2D	Translation
DMR1:208044001	1	208044001	2000	1	9.30E-06	0.5889473	45	2.25	PLXNA2	
DMR1:208288001	1	208288001	1000	1	3.17E-08	0.7248202	8	0.8	LOC105372889	
DMR1:212765001	1	212765001	3000	1	2.90E-06	0.6987251	25	0.833333333	NSL1	
DMR1:213383001	1	213383001	1000	1	8.00E-06	0.8657541	13	1.3	RPS6KC1	Signaling
DMR1:213566001	1	213566001	1000	1	8.96E-07	-0.7805615	1	0.1	LOC105372912	
DMR1:214755001	1	214755001	1000	1	3.91E-06	-0.6390958	21	2.1		
DMR1:216625001	1	216625001	2000	1	8.81E-06	0.561813	12	0.6	ESRRG	
DMR1:218091001	1	218091001	1000	1	5.69E-06	0.5678855	13	1.3		
DMR1:219922001	1	219922001	1000	1	8.20E-06	-0.521502	12	1.2	SLC30A10;LOC107985281	
DMR1:221332001	1	221332001	1000	1	1.12E-07	0.7197536	11	1.1	LOC105372932;LINC02817	
DMR1:221372001	1	221372001	1000	1	3.28E-08	1.2503155	13	1.3	LOC105372932	
DMR1:221948001	1	221948001	3000	1	1.49E-08	0.699405	23	0.766666667	LINC02257	
DMR1:222461001	1	222461001	1000	1	3.26E-09	-0.880167	8	0.8	TRT-TGT2-1;CICP13;LOC728417	
DMR1:223652001	1	223652001	1000	1	7.59E-07	0.8554162	17	1.7	CAPN8;SNRPEP10	Protease
DMR1:224465001	1	224465001	1000	1	9.93E-07	-0.6428898	21	2.1	CNIH3	Transport
DMR1:226013001	1	226013001	2000	1	1.08E-07	0.7781699	21	1.05		
DMR1:226085001	1	226085001	1000	1	3.25E-07	-0.5854708	24	2.4	LINC01703	
DMR1:228663001	1	228663001	1000	1	1.02E-09	-0.8450471	21	2.1	RHOU	Signaling
DMR1:230469001	1	230469001	1000	1	3.11E-07	-0.6727861	14	1.4		
DMR1:232760001	1	232760001	2000	1	3.01E-06	0.6285952	10	0.5	LOC107983960	
DMR1:233595001	1	233595001	1000	1	4.90E-06	-0.8553726	20	2		
DMR1:234948001	1	234948001	1000	1	4.91E-06	-0.7198252	20	2	LOC105373211;LOC101927851	
DMR1:236069001	1	236069001	1000	1	2.25E-06	-0.4541683	41	4.1	NID1	Receptor
DMR1:236890001	1	236890001	2000	1	1.98E-06	0.8240005	45	2.25	MTR	
DMR1:238403001	1	238403001	1000	1	3.10E-07	0.7814607	9	0.9	LOC105373220	
DMR1:238641001	1	238641001	1000	1	1.71E-09	-1.1376065	3	0.3		
DMR1:242297001	1	242297001	2000	1	7.34E-06	0.7629349	37	1.85	PLD5	Metabolism
DMR1:242327001	1	242327001	1000	1	7.84E-06	-0.8823809	7	0.7	PLD5	Metabolism
DMR1:243048001	1	243048001	2000	1	2.09E-06	-0.7382916	31	1.55	SEPTIN14P21;CICP21;LOC105373249;LOC102724236;LINC01347	
DMR1:245357001	1	245357001	1000	1	2.97E-07	0.7284551	12	1.2	KIF26B	Cytoskeleton
DMR1:246413001	1	246413001	1000	1	1.29E-07	1.0056099	12	1.2	SMYD3	Epigenetic
DMR1:246519001	1	246519001	3000	1	1.55E-06	0.8136924	84	2.8	LINC01743	
DMR1:246636001	1	246636001	4000	1	2.60E-08	0.7143305	88	2.2	CNST;LOC100887078;LOC107985100	
DMR1:246792001	1	246792001	1000	1	4.80E-06	0.6148976	34	3.4	KIF28P;LINC01341	Cytoskeleton
DMR1:246922001	1	246922001	1000	1	6.75E-11	-0.8984442	24	2.4	AHCTF1	Cytoskeleton
DMR1:247757001	1	247757001	2000	1	1.60E-06	0.7260695	24	1.2	OR14A2;OR1C1	Signaling;Receptor
DMR2:427001	2	427001	1000	1	6.96E-06	0.7948686	73	7.3		
DMR2:494001	2	494001	3000	1	1.04E-06	0.6722209	127	4.233333333	LINC01874;LOC100996637	
DMR2:628001	2	628001	2000	1	1.81E-06	0.6885116	51	2.55		
DMR2:878001	2	878001	1000	1	1.62E-06	-0.4531905	23	2.3	LINC01115	
DMR2:1165001	2	1165001	2000	1	1.86E-08	1.0807659	37	1.85	SNTG2	
DMR2:1422001	2	1422001	4000	1	4.32E-07	0.6803661	90	2.25	TPO	Metabolism
DMR2:1457001	2	1457001	5000	2	2.82E-09	0.8742404	79	1.58	TPO	Metabolism
DMR2:1558001	2	1558001	1000	1	6.87E-06	-0.4779001	32	3.2	LOC102723730	
DMR2:1746001	2	1746001	1000	1	7.95E-06	0.8141076	8	0.8	PXDN	Metabolism
DMR2:2054001	2	2054001	1000	1	8.94E-08	0.7274486	7	0.7	MYT1L	Transcription
DMR2:2693001	2	2693001	3000	1	1.87E-07	0.7994089	72	2.4		
DMR2:2906001	2	2906001	1000	1	6.50E-07	-0.9077619	14	1.4	LINC01250	
DMR2:3007001	2	3007001	1000	1	1.90E-06	0.7379657	18	1.8	LINC01250	
DMR2:3312001	2	3312001	1000	1	4.71E-09	0.9485996	18	1.8	EIPR1	
DMR2:3527001	2	3527001	3000	1	2.85E-06	-0.4422915	99	3.3	ADI1;RNASEH1	Metabolism;Translation
DMR2:4067001	2	4067001	1000	1	3.56E-07	0.7923999	12	1.2		
DMR2:4503001	2	4503001	1000	1	6.68E-06	1.0896902	13	1.3		
DMR2:4864001	2	4864001	1000	1	1.05E-06	0.9050802	15	1.5		
DMR2:6965001	2	6965001	1000	1	3.56E-06	0.5857355	20	2	RNF144A	Proteolysis
DMR2:8361001	2	8361001	1000	1	6.08E-06	0.5560569	16	1.6		
DMR2:9149001	2	9149001	2000	1	4.39E-06	0.9594141	45	2.25	LOC105373417	
DMR2:9550001	2	9550001	1000	1	1.47E-06	-0.7850071	13	1.3	ADAM17	Protease
DMR2:10696001	2	10696001	4000	1	5.65E-07	-0.5789665	115	2.875	NOL10;RN7SL832P	

DMR2:11419001	2	11419001	1000	1	5.32E-06	-0.7757836	15	1.5	LOC105373429	
DMR2:11874001	2	11874001	2000	1	1.46E-06	-0.7213376	27	1.35	LOC105373430	
DMR2:12583001	2	12583001	1000	1	7.67E-06	0.7969233	10	1	MIR3681HG	
DMR2:12934001	2	12934001	1000	1	9.23E-06	0.5760597	8	0.8		
DMR2:16641001	2	16641001	2000	1	1.05E-06	-0.6075877	19	0.95	CYRIA	
DMR2:18875001	2	18875001	1000	1	4.34E-06	-0.5333991	38	3.8	LOC105373456	
DMR2:20442001	2	20442001	1000	1	3.61E-08	-0.6541741	44	4.4	RHOB	Signaling
DMR2:23831001	2	23831001	1000	1	2.28E-06	0.6526658	4	0.4	ATAD2B	Epigenetic
DMR2:24042001	2	24042001	1000	1	9.86E-07	-0.4648409	34	3.4	WDCP;FKBP1B;RNU6-370P	Transcription
DMR2:24751001	2	24751001	1000	1	4.92E-09	-0.686103	21	2.1	NCOA1	Epigenetic
DMR2:25004001	2	25004001	2000	1	7.56E-06	-0.8350832	48	2.4	DNAJC27-AS1	
DMR2:25863001	2	25863001	3000	1	2.59E-09	-0.7757758	71	2.366666667	ASXL2	
DMR2:27446001	2	27446001	1000	1	7.02E-07	-0.6313963	28	2.8	NRBP1;KRTCAP3;IFT172	Signaling;Development
DMR2:28880001	2	28880001	2000	1	5.12E-06	-0.4907913	44	2.2	TRMT61B	Epigenetic
DMR2:29907001	2	29907001	1000	1	4.79E-07	0.6460639	10	1	ALK	Receptor
DMR2:32050001	2	32050001	3000	1	1.28E-06	-0.5177586	77	2.566666667		
DMR2:32110001	2	32110001	2000	1	9.47E-07	-0.9958924	25	1.25	SPAST	Cytoskeleton
DMR2:32149001	2	32149001	2000	1	3.96E-06	-0.7693334	27	1.35	SPAST	Cytoskeleton
DMR2:32866001	2	32866001	1000	1	1.96E-09	-0.7297337	2	0.2	LINC00486	
DMR2:33725001	2	33725001	1000	1	8.83E-06	-0.7081371	18	1.8	LINC01317;MYADML	
DMR2:33990001	2	33990001	2000	1	4.16E-06	-0.9142924	21	1.05	LINC01317	
DMR2:35832001	2	35832001	1000	1	6.10E-07	0.7506133	5	0.5		
DMR2:37034001	2	37034001	3000	1	4.57E-07	-0.6232144	53	1.766666667	HEATR5B	
DMR2:38844001	2	38844001	1000	1	7.16E-07	-0.7422146	27	2.7	DHX57;LOC105374470	Transcription
DMR2:38937001	2	38937001	1000	1	8.65E-06	-0.6521302	16	1.6	ARHGEF33;LOC105374471;RN7SL96P	Transcription
DMR2:41382001	2	41382001	3000	1	9.51E-07	0.5924171	32	1.066666667		
DMR2:42930001	2	42930001	1000	1	5.49E-06	-0.7842493	16	1.6	LOC105374567;LOC105374568	
DMR2:43431001	2	43431001	1000	1	1.47E-06	-0.4455307	35	3.5	THADA	Cytoskeleton
DMR2:44406001	2	44406001	1000	1	4.90E-06	0.8672192	9	0.9	CAMKMT	Golgi
DMR2:46283001	2	46283001	1000	1	3.85E-07	0.769266	8	0.8	LOC101926974	
DMR2:47383001	2	47383001	1000	1	9.44E-07	-0.6254894	26	2.6	EPCAM;MIR559	
DMR2:47537001	2	47537001	3000	1	3.42E-07	0.6401614	47	1.566666667	MSH2;KCNK12;MSH2-OT1	Transcription;Transport
DMR2:47788001	2	47788001	1000	1	4.33E-10	-0.8680137	21	2.1	MSH6;RPL36AP15	Transcription
DMR2:50399001	2	50399001	1000	1	5.36E-07	-0.851038	6	0.6	NRXN1	
DMR2:52570001	2	52570001	1000	1	4.32E-07	0.6453	23	2.3	CRTC1P1	
DMR2:60441001	2	60441001	1000	1	6.65E-06	0.7811003	11	1.1	BCL11A	Transcription
DMR2:68449001	2	68449001	1000	1	3.40E-08	-1.0521309	10	1	WDR4P2;FBXO48	
DMR2:68451001	2	68451001	1000	1	2.51E-47	-4.9374401	0	0	WDR4P2;FBXO48	
DMR2:70100001	2	70100001	1000	1	1.79E-07	-0.6526189	27	2.7	MRPL36P1	
DMR2:71617001	2	71617001	2000	1	6.46E-06	-0.3922627	22	1.1	DYSF	Transport
DMR2:73137001	2	73137001	1000	1	1.16E-06	-0.5115336	38	3.8		
DMR2:73748001	2	73748001	1000	1	7.98E-06	-0.6270771	8	0.8		
DMR2:74468001	2	74468001	2000	1	4.97E-06	-0.5776359	39	1.95	INO80B-WBP1;WBP1;MOGS;MRPL53;CCDC142	Metabolism;Translation
DMR2:74868001	2	74868001	1000	1	6.43E-07	0.7051928	16	1.6	HK2	Signaling
DMR2:76430001	2	76430001	1000	1	2.64E-08	0.8043098	11	1.1		
DMR2:78760001	2	78760001	5000	1	9.18E-09	0.986603	54	1.08		
DMR2:84445001	2	84445001	2000	1	5.19E-06	0.5092792	15	0.75	SUCLG1	Metabolism
DMR2:85365001	2	85365001	2000	1	8.57E-06	-0.656477	29	1.45	ELMOD3;RN7SL113P	Cytoskeleton
DMR2:88021001	2	88021001	1000	1	1.58E-06	-0.8744131	25	2.5	LOC105374852;RNU2-63P;KRCC1	
DMR2:89654001	2	89654001	2000	1	1.05E-06	-0.6796777	16	0.8	IGK	
DMR2:90311001	2	90311001	1000	1	3.61E-08	-0.5831392	20	2	LOC101926946;IGKV1OR2-118	
DMR2:92270001	2	92270001	1000	1	2.13E-08	-0.7713438	14	1.4		
DMR2:93921001	2	93921001	1000	1	3.48E-06	-0.7396541	13	1.3		
DMR2:94196001	2	94196001	1000	1	1.69E-06	0.6670274	14	1.4	BMS1P23	
DMR2:96188001	2	96188001	1000	1	2.25E-09	-0.7155744	19	1.9	STARD7	
DMR2:96804001	2	96804001	1000	1	4.71E-08	-0.7208382	25	2.5	CNNM4;MIR3127;CNNM3-DT;CNNM3	
DMR2:96957001	2	96957001	2000	1	5.33E-06	-0.644125	43	2.15	FAM178B;RNA5SP101	
DMR2:97737001	2	97737001	2000	1	8.90E-07	0.4766484	49	2.45	ZAP70	
DMR2:98058001	2	98058001	2000	1	2.23E-08	0.6460375	33	1.65	LOC105373501	
DMR2:98582001	2	98582001	1000	1	7.25E-10	0.6991041	17	1.7	INPP4A	
DMR2:100765001	2	100765001	2000	1	9.37E-06	1.0697101	16	0.8		
DMR2:101449001	2	101449001	1000	1	1.50E-08	0.6346238	7	0.7	RFX8	Transcription
DMR2:102502001	2	102502001	1000	1	6.02E-07	0.7790148	19	1.9	SLC9A4	Transport
DMR2:103361001	2	103361001	2000	2	8.08E-07	-0.7015929	42	2.1		
DMR2:105314001	2	105314001	1000	1	2.51E-06	-0.6023572	21	2.1	TGFBRAP1	
DMR2:105688001	2	105688001	2000	1	7.01E-06	-0.695232	37	1.85		

DMR2:107605001	2	107605001	1000	1	8.57E-07	-1.0158199	1	0.1		
DMR2:108509001	2	108509001	1000	1	3.95E-06	-0.8082155	18	1.8	GCC2;GCC2-AS1	Cytoskeleton
DMR2:108811001	2	108811001	1000	1	2.47E-06	-0.5553767	4	0.4	CCDC138	
DMR2:112776001	2	112776001	1000	1	6.85E-07	-0.7942322	23	2.3	IL1A	Cytokine
DMR2:113667001	2	113667001	1000	1	7.97E-07	-0.7155783	27	2.7	SNRPA1P1;ACRP1	
DMR2:115067001	2	115067001	1000	1	6.00E-07	-0.6100259	38	3.8	DPP10;LOC105373575	Protease
DMR2:121236001	2	121236001	1000	1	9.80E-06	0.6695152	25	2.5	TFCP2L1	Transcription
DMR2:121898001	2	121898001	1000	1	3.54E-08	-0.8670539	13	1.3	LOC105373592	
DMR2:122840001	2	122840001	1000	1	2.19E-06	-0.7188034	16	1.6		
DMR2:127246001	2	127246001	3000	1	1.48E-06	0.679478	62	2.066666667	WBP11P2;ERCC3	Epigenetic
DMR2:136345001	2	136345001	1000	1	8.21E-15	-1.77037	8	0.8		
DMR2:137487001	2	137487001	1000	1	1.03E-07	-0.5876592	39	3.9	THSD7B;LOC105373634	Cytoskeleton
DMR2:141126001	2	141126001	1000	1	6.65E-06	0.7506397	9	0.9	LRP1B	
DMR2:142677001	2	142677001	1000	1	6.75E-06	-0.7226411	26	2.6	LOC107985823	
DMR2:143092001	2	143092001	1000	1	4.95E-10	0.8054388	18	1.8	ARHGAP15;MTCYBP11;MTND6P11;MTND5P24;MTND4P22;MTND3P9;MTCO3P5;MTATP6P5;MTCO2P5	Signaling
DMR2:147568001	2	147568001	1000	1	4.14E-06	-0.4994708	5	0.5		
DMR2:148446001	2	148446001	1000	1	2.99E-07	-0.8931678	4	0.4	MBD5	
DMR2:148834001	2	148834001	3000	1	6.17E-06	0.5003797	23	0.766666667	RNU2-9P	
DMR2:149538001	2	149538001	1000	1	6.61E-08	0.7602282	13	1.3		
DMR2:151376001	2	151376001	1000	1	3.74E-08	-0.9381463	9	0.9	LOC101929319;TNFAIP6;MIR4773-2;MIR4773-1;RN7SL124P	
DMR2:152130001	2	152130001	1000	1	2.99E-06	-0.6753619	20	2	STAM2;RPL30P2	Cytoskeleton
DMR2:152707001	2	152707001	1000	1	3.53E-09	0.7676704	14	1.4	PRPF40A;ARL6IP6	
DMR2:155263001	2	155263001	1000	1	2.67E-06	0.5654285	20	2	MTCO1P45;LOC105373698;MTND2P20;ATP5F1AP2	
DMR2:155331001	2	155331001	1000	1	3.74E-06	-0.6166784	25	2.5	RNU6-546P	
DMR2:157020001	2	157020001	1000	1	9.29E-07	-0.7854319	9	0.9	LOC105373710;LOC105373709	
DMR2:157742001	2	157742001	1000	1	6.71E-06	0.4631066	19	1.9	ACVR1	Signaling
DMR2:159754001	2	159754001	2000	1	6.31E-06	0.6425774	10	0.5	MARCHF7	Proteolysis
DMR2:164374001	2	164374001	1000	1	2.64E-06	-0.6941582	12	1.2		
DMR2:167545001	2	167545001	1000	1	1.44E-06	-0.6563793	16	1.6	B3GALT1	Golgi
DMR2:168058001	2	168058001	1000	1	2.26E-06	0.4430423	5	0.5	STK39	
DMR2:168240001	2	168240001	1000	1	1.15E-06	0.8981989	4	0.4	STK39;PHF5GP;LOC107985959	
DMR2:168728001	2	168728001	1000	1	6.04E-06	-0.7989107	19	1.9	CERS6	
DMR2:168975001	2	168975001	1000	1	1.32E-47	-4.9064872	0	0	ABCB11	Transport
DMR2:172857001	2	172857001	1000	1	3.41E-06	-0.6703915	16	1.6	RAPGEF4	Transcription
DMR2:173294001	2	173294001	1000	1	6.52E-06	-0.7113837	24	2.4	LOC105373743;RPS2P18	
DMR2:173784001	2	173784001	1000	1	2.04E-12	1.0298004	12	1.2		
DMR2:178654001	2	178654001	2000	1	1.54E-07	0.8486801	19	0.95	TTN	
DMR2:178699001	2	178699001	1000	1	2.75E-11	-0.641074	40	4	TTN	
DMR2:181275001	2	181275001	1000	1	5.03E-06	-0.6826473	5	0.5	LINC01934	
DMR2:182385001	2	182385001	1000	1	1.86E-08	-0.8581252	7	0.7	PDE1A	Signaling
DMR2:183385001	2	183385001	1000	1	4.73E-06	-0.7027229	27	2.7		
DMR2:185036001	2	185036001	1000	1	4.74E-07	-0.7744875	20	2		
DMR2:185552001	2	185552001	1000	1	1.34E-06	-0.6492448	6	0.6	ELF2P4	
DMR2:186191001	2	186191001	1000	1	5.82E-06	-0.5739269	6	0.6		
DMR2:193908001	2	193908001	1000	1	4.06E-08	-0.7293737	24	2.4		
DMR2:194135001	2	194135001	2000	1	6.61E-07	0.6646447	26	1.3	GLULP6	
DMR2:196603001	2	196603001	1000	1	5.27E-09	-0.7929603	11	1.1	HECW2	Proteolysis
DMR2:197027001	2	197027001	2000	1	5.18E-06	0.7383692	18	0.9	ANKRD44;RPL4P7	Cytoskeleton
DMR2:200530001	2	200530001	2000	1	2.80E-06	0.6559366	6	0.3	SGO2	Cell Cycle
DMR2:201097001	2	201097001	3000	1	1.56E-07	-0.6198736	82	2.733333333	RPL17P10;LOC105373836	
DMR2:202212001	2	202212001	2000	1	7.40E-06	-0.6044992	37	1.85	KIAA2012;SUMO1	
DMR2:204549001	2	204549001	1000	1	7.77E-09	1.0086978	7	0.7	PARD3B	
DMR2:205380001	2	205380001	1000	1	3.85E-44	-4.1695348	2	0.2	PARD3B	
DMR2:206071001	2	206071001	1000	1	3.36E-06	-0.5944438	9	0.9	INO80D	
DMR2:206156001	2	206156001	1000	1	8.89E-08	-0.7891951	13	1.3	NDUFS1;EEF1B2;SNORD51;SNORA41	Metabolism;Translation
DMR2:206172001	2	206172001	3000	2	2.18E-08	-0.7947329	89	2.966666667	EEF1B2;SNORA41;CMKLR2	Translation
DMR2:206179001	2	206179001	1000	1	8.62E-06	-0.6052212	19	1.9	CMKLR2	
DMR2:207813001	2	207813001	1000	1	8.60E-06	-0.4881614	20	2	PLEKHM3	
DMR2:208094001	2	208094001	1000	1	1.48E-07	-0.6893627	37	3.7		
DMR2:211778001	2	211778001	2000	1	1.61E-06	0.6625571	36	1.8	ERBB4;MTND2P23;MTCO1P46	Receptor
DMR2:215296001	2	215296001	2000	1	3.57E-07	-0.6249832	50	2.5		
DMR2:216010001	2	216010001	1000	1	1.33E-06	-0.606195	33	3.3	MREG	
DMR2:216024001	2	216024001	2000	1	1.50E-07	-0.6381193	23	1.15	MREG;PECR	Metabolism
DMR2:216966001	2	216966001	1000	1	3.42E-09	-0.7392767	12	1.2	LOC101928278	

DMR2:220806001	2	220806001	1000	1	1.79E-06	0.8005651	7	0.7	LOC105373896;LOC107985989;LOC107985988	
DMR2:221381001	2	221381001	1000	1	2.38E-15	-1.4765378	20	2		
DMR2:221794001	2	221794001	2000	1	9.14E-06	-0.5823839	13	0.65		
DMR2:222619001	2	222619001	1000	1	2.07E-06	-0.6974098	15	1.5	FARSB	Translation
DMR2:224677001	2	224677001	1000	1	2.31E-08	-0.6567587	36	3.6	LOC105373910	
DMR2:227399001	2	227399001	2000	1	5.46E-06	-0.594418	27	1.35		
DMR2:228341001	2	228341001	1000	1	4.22E-06	0.8501972	6	0.6		
DMR2:228797001	2	228797001	1000	1	8.14E-06	0.6886932	11	1.1		
DMR2:228895001	2	228895001	4000	1	4.66E-06	0.607566	30	0.75		
DMR2:231337001	2	231337001	1000	1	1.52E-06	-0.6244374	25	2.5	ARMC9	Cytoskeleton
DMR2:231628001	2	231628001	3000	1	9.74E-06	-0.6563617	72	2.4		
DMR2:231834001	2	231834001	1000	1	4.11E-06	0.9105096	17	1.7		
DMR2:231848001	2	231848001	1000	1	3.59E-07	1.0082596	12	1.2		
DMR2:232604001	2	232604001	2000	1	1.07E-07	-0.6719073	68	3.4	LOC105373929;EFHD1	Signaling
DMR2:233788001	2	233788001	1000	1	3.82E-09	-0.9149298	23	2.3	MROH2A	
DMR2:233907001	2	233907001	2000	1	2.34E-07	0.7185702	13	0.65	TRPM8	Transport
DMR2:234514001	2	234514001	2000	1	7.62E-08	-0.5119554	28	1.4		
DMR2:235302001	2	235302001	2000	1	7.38E-07	-0.5346163	54	2.7		
DMR2:235461001	2	235461001	1000	1	5.83E-08	0.6765192	16	1.6		
DMR2:235765001	2	235765001	1000	1	2.46E-06	-0.4769417	47	4.7	AGAP1;LOC105373942	
DMR2:235841001	2	235841001	1000	1	2.13E-06	0.5338412	23	2.3	AGAP1	
DMR2:237445001	2	237445001	3000	1	7.03E-06	0.6484971	68	2.266666667		
DMR2:238236001	2	238236001	1000	1	1.19E-07	0.8595735	16	1.6	LINC02610;TARDBPP3;HES6;PER2	Transcription;Transcription
DMR2:238252001	2	238252001	1000	1	5.61E-06	0.5650319	31	3.1	PER2	Transcription
DMR2:238901001	2	238901001	4000	1	7.51E-06	-0.4569608	35	0.875	TWIST2	Transcription
DMR2:238907001	2	238907001	3000	1	7.54E-06	-0.3510039	79	2.633333333	TWIST2;LINC01940	Transcription
DMR2:239386001	2	239386001	1000	1	5.46E-06	0.5794735	28	2.8	HDAC4	
DMR2:239641001	2	239641001	3000	1	8.78E-06	-0.4359174	38	1.266666667		
DMR2:240042001	2	240042001	2000	1	4.04E-10	-1.0327047	75	3.75	OR6B3	Signaling
DMR2:240923001	2	240923001	2000	2	1.06E-07	-0.8523041	58	2.9	CROCC2;LOC112268440	Epigenetic
DMR2:240984001	2	240984001	2000	1	1.29E-09	-0.5831159	21	1.05	CROCC2	Epigenetic
DMR2:241570001	2	241570001	2000	2	4.26E-06	-0.5972912	86	4.3	BOK;LOC105373975	
DMR2:241618001	2	241618001	3000	1	1.52E-06	-0.4562664	61	2.033333333	THAP4	
DMR2:241694001	2	241694001	1000	1	5.77E-06	-0.6162751	27	2.7	DTYMK;ING5	Signaling;Epigenetic
DMR2:241861001	2	241861001	2000	1	5.75E-08	-0.4391968	25	1.25	PDCD1;LOC105373977;RTP5	
DMR2:241896001	2	241896001	1000	1	1.53E-06	-0.6807573	17	1.7	LINC01237;FAM240C	
DMR2:241996001	2	241996001	2000	1	4.14E-06	0.7146807	56	2.8	LINC01237;LOC285097	
DMR2:242054001	2	242054001	3000	1	5.87E-06	0.8499569	82	2.733333333	LINC01237;LINC01880	
DMR2:242121001	2	242121001	1000	1	1.14E-07	-0.5950723	11	1.1	LINC01881;CICP10;SEPTIN14P2	
DMR3:2705001	3	2705001	1000	1	3.19E-08	-1.126035	13	1.3	CNTN4	Cytoskeleton
DMR3:9621001	3	9621001	2000	1	1.61E-07	-0.720546	38	1.9		
DMR3:9882001	3	9882001	2000	1	1.52E-06	-0.4674073	67	3.35	CIDEC;JAGN1	
DMR3:13702001	3	13702001	8000	2	1.75E-07	0.7120734	70	0.875	LINC00620	
DMR3:15945001	3	15945001	3000	1	1.78E-06	-0.70546	40	1.333333333		
DMR3:18826001	3	18826001	2000	1	5.33E-46	-4.6402807	18	0.9		
DMR3:20817001	3	20817001	5000	1	1.03E-06	-0.7916429	47	0.94		
DMR3:22697001	3	22697001	1000	1	3.87E-07	-0.5772357	21	2.1		
DMR3:23906001	3	23906001	1000	1	4.39E-09	-0.7332443	11	1.1	NKIRAS1;RPL15	Translation
DMR3:24254001	3	24254001	1000	1	2.18E-09	-0.9156358	10	1	THRB	Transcription
DMR3:27636001	3	27636001	1000	1	8.94E-07	-0.6397785	10	1	LOC643634	
DMR3:31965001	3	31965001	1000	1	6.80E-12	-1.5055168	11	1.1	OSBPL10	
DMR3:32114001	3	32114001	2000	1	9.80E-10	0.8974083	33	1.65	GPD1L	Metabolism
DMR3:32515001	3	32515001	1000	1	1.16E-07	-0.8704575	24	2.4	MIR548AY;RPL31P18;DYNC1LI1	Cytoskeleton
DMR3:32540001	3	32540001	1000	1	4.74E-06	-0.8652184	14	1.4	DYNC1LI1	Cytoskeleton
DMR3:33361001	3	33361001	1000	1	7.82E-06	-1.0522314	12	1.2	FBXL2	
DMR3:37629001	3	37629001	1000	1	5.11E-06	0.8733062	19	1.9	ITGA9	Extracellular Matrix
DMR3:40713001	3	40713001	2000	1	2.70E-06	0.825577	21	1.05	LOC105377043	
DMR3:41414001	3	41414001	2000	1	8.36E-06	0.713325	23	1.15	ULK4	Signaling
DMR3:44525001	3	44525001	1000	1	1.96E-06	0.9500578	13	1.3	EI24P3;LOC100419748	
DMR3:45444001	3	45444001	1000	1	4.05E-06	-0.7175648	30	3	LARS2	Translation
DMR3:47382001	3	47382001	1000	1	6.68E-06	-0.5293134	32	3.2	PTPN23	Transport
DMR3:47789001	3	47789001	3000	1	4.46E-07	-0.5821373	104	3.466666667	SMARCC1	Epigenetic
DMR3:48486001	3	48486001	1000	1	9.59E-09	-0.9253186	10	1	SHISA5	Cytoskeleton
DMR3:48808001	3	48808001	1000	1	1.85E-06	-0.51449	35	3.5	PRKAR2A	Signaling
DMR3:48939001	3	48939001	1000	1	1.49E-06	-0.6308861	18	1.8	ARIH2	Proteolysis
DMR3:49291001	3	49291001	1000	1	2.31E-06	-0.634151	23	2.3	USP4	Protease
DMR3:49348001	3	49348001	1000	1	4.71E-06	-0.5788798	29	2.9	USP4;GPX1	Protease;Metabolism

DMR3:49431001	3	49431001	2000	1	4.41E-06	-0.5028087	38	1.9	AMT;NICN1	Epigenetic
DMR3:49920001	3	49920001	4000	1	4.21E-06	-0.6894561	86	2.15	LOC102724438;MON1A	
DMR3:51850001	3	51850001	2000	1	8.26E-06	0.5441171	30	1.5	IQCF2	
DMR3:53341001	3	53341001	2000	1	2.58E-06	0.7771539	23	1.15	DCP1A;SNORD38C;LOC105377094	Translation
DMR3:55236001	3	55236001	1000	1	9.01E-06	0.7150693	10	1	LOC105377097	
DMR3:56255001	3	56255001	1000	1	7.05E-06	0.7854033	9	0.9	ERC2	Transport
DMR3:57610001	3	57610001	1000	1	4.21E-06	0.7645962	11	1.1	PDE12;ARF4-AS1;RNU6ATAC26P	Translation
DMR3:57885001	3	57885001	1000	1	4.07E-07	-0.4940691	30	3	SLMAP	Cytoskeleton
DMR3:58374001	3	58374001	1000	1	6.65E-10	-0.7054208	13	1.3	PXK	
DMR3:58386001	3	58386001	1000	1	7.20E-08	-1.1609487	6	0.6	PXK	
DMR3:59051001	3	59051001	1000	1	2.36E-06	0.5771575	40	4	CFAP20DC;LOC105377110	
DMR3:64802001	3	64802001	2000	1	1.46E-06	0.5400363	23	1.15	ADAMTS9-AS2	
DMR3:70267001	3	70267001	1000	1	1.13E-07	-0.6811223	22	2.2	MDFIC2	
DMR3:70612001	3	70612001	3000	1	1.40E-06	0.5742373	42	1.4		
DMR3:71435001	3	71435001	2000	1	4.26E-12	-0.9396866	45	2.25	FOXP1	
DMR3:72133001	3	72133001	1000	1	1.61E-06	0.7541584	11	1.1		
DMR3:76012001	3	76012001	3000	1	5.91E-06	0.4956177	65	2.166666667	ROBO2	
DMR3:77289001	3	77289001	8000	1	9.73E-06	0.5756465	171	2.1375	ROBO2	
DMR3:77433001	3	77433001	1000	1	5.81E-06	0.7226882	9	0.9	ROBO2	
DMR3:80224001	3	80224001	1000	1	2.65E-06	0.815114	8	0.8	HNRNPA3P8	
DMR3:80603001	3	80603001	1000	1	7.26E-06	-0.5189496	30	3	LOC105377177	
DMR3:84188001	3	84188001	3000	1	3.15E-10	0.8461603	26	0.866666667		
DMR3:85693001	3	85693001	1000	1	2.86E-06	-0.5207595	29	2.9	CADM2	
DMR3:88176001	3	88176001	1000	1	3.11E-06	-0.92777	13	1.3		
DMR3:89875001	3	89875001	1000	1	7.84E-08	0.9456176	13	1.3		
DMR3:90204001	3	90204001	2000	1	1.41E-09	-0.844624	39	1.95	PROS2P	
DMR3:91260001	3	91260001	3000	1	3.23E-06	-0.63295	41	1.366666667		
DMR3:91542001	3	91542001	12000	8	4.95E-14	-0.6651684	183	1.525		
DMR3:92042001	3	92042001	1000	1	9.03E-06	-0.8184669	17	1.7		
DMR3:93470001	3	93470001	1000	1	2.01E-12	-1.0663141	8	0.8		
DMR3:93705001	3	93705001	9000	9	4.48E-15	-0.6656631	153	1.7		
DMR3:96374001	3	96374001	2000	1	5.98E-06	0.7108397	25	1.25		
DMR3:97186001	3	97186001	1000	1	2.96E-06	1.1365426	9	0.9	EPHA6	Receptor
DMR3:97988001	3	97988001	2000	2	3.12E-06	-0.5485564	17	0.85	GABRR3	Ion Channel
DMR3:100224001	3	100224001	2000	1	1.60E-07	-0.6524886	57	2.85	DUSP12P1;VTI1BP1	
DMR3:107391001	3	107391001	1000	1	2.42E-06	0.8439503	6	0.6	LOC101929579	
DMR3:108005001	3	108005001	1000	1	2.92E-06	-0.5776987	22	2.2		
DMR3:109339001	3	109339001	3000	1	1.53E-06	-0.6691834	80	2.666666667	DPPA4	
DMR3:112524001	3	112524001	1000	1	1.10E-06	0.8779993	10	1	OR7E100P;ATG3	Proteolysis
DMR3:113080001	3	113080001	1000	1	7.91E-06	-0.6815966	7	0.7	LOC107986114	
DMR3:113666001	3	113666001	1000	1	5.72E-09	-0.8046617	23	2.3	USF3	
DMR3:116031001	3	116031001	1000	1	1.43E-07	-1.0033118	3	0.3	LSAMP	Immune
DMR3:118673001	3	118673001	1000	1	1.60E-06	-0.6253168	25	2.5	LOC105374060;LOC107983969	
DMR3:121919001	3	121919001	1000	1	2.46E-06	0.7742004	21	2.1	SLC15A2	Transport
DMR3:122452001	3	122452001	1000	1	6.26E-09	-0.6543369	14	1.4	WDR5B-DT;KPNA1	Transport
DMR3:125093001	3	125093001	1000	1	3.10E-06	0.8390958	4	0.4	SLC12A8	Transport
DMR3:126035001	3	126035001	1000	1	9.40E-06	0.5927744	16	1.6	SLC41A3	Transport
DMR3:128669001	3	128669001	3000	1	2.41E-06	-0.5563068	56	1.866666667	POU5F1P6	
DMR3:128673001	3	128673001	3000	1	3.36E-07	-0.6592367	56	1.866666667	POU5F1P6	
DMR3:128716001	3	128716001	1000	1	1.82E-06	-0.606258	15	1.5	RAB7A	
DMR3:129124001	3	129124001	1000	1	3.54E-06	-0.5573598	34	3.4	RAB43;ISY1-RAB43;ISY1	Translation
DMR3:129136001	3	129136001	2000	1	9.73E-06	-0.5104555	51	2.55	ISY1-RAB43;ISY1	Translation
DMR3:129164001	3	129164001	1000	1	4.53E-06	-0.6303164	18	1.8	ISY1-RAB43;ISY1;CNBP	Translation;Metabolism
DMR3:130747001	3	130747001	1000	1	1.04E-11	-1.0492504	51	5.1	PIK3R4;LOC107986023	Signaling
DMR3:132361001	3	132361001	1000	1	1.68E-06	0.6939904	6	0.6	ACP3	Signaling
DMR3:133145001	3	133145001	2000	1	5.18E-06	0.8528137	12	0.6	TMEM108	
DMR3:136376001	3	136376001	2000	1	6.93E-06	-0.7875833	34	1.7	STAG1	Epigenetic
DMR3:136769001	3	136769001	1000	1	7.54E-07	-0.7277639	15	1.5		
DMR3:136867001	3	136867001	1000	1	5.85E-06	-0.5912928	10	1	NCK1-DT;NCK1	Cytoskeleton
DMR3:138847001	3	138847001	2000	1	5.17E-06	-0.5666929	71	3.55		
DMR3:139801001	3	139801001	1000	1	1.23E-07	1.017869	7	0.7		
DMR3:141034001	3	141034001	1000	1	1.85E-06	-0.7904984	12	1.2		
DMR3:142790001	3	142790001	1000	1	7.66E-06	0.761231	10	1	TRPC1	Transport
DMR3:146214001	3	146214001	1000	1	9.08E-07	-0.7660171	25	2.5	PLSCR4	Transport
DMR3:146337001	3	146337001	1000	1	7.09E-06	-0.6436987	13	1.3	LOC100419967	
DMR3:147159001	3	147159001	1000	1	5.78E-06	-0.5852277	21	2.1		
DMR3:154210001	3	154210001	1000	1	8.23E-06	-0.8615631	17	1.7	ARHGEF26	Transcription
DMR3:156053001	3	156053001	1000	1	6.15E-06	0.7844747	22	2.2	ALG1L15P	

DMR3:156541001	3	156541001	1000	1	2.49E-06	-0.7416017	9	0.9	KCNAB1;SSR3	
DMR3:158341001	3	158341001	2000	1	4.20E-06	1.0393469	21	1.05	RSRC1;LOC100422604	
DMR3:158571001	3	158571001	2000	1	6.21E-09	-0.5982831	81	4.05	LOC100996447;MLF1	Signaling
DMR3:160087001	3	160087001	1000	1	4.53E-06	0.6141763	13	1.3	IL12A-AS1	
DMR3:160095001	3	160095001	2000	1	5.06E-06	0.6533073	15	0.75	IL12A-AS1;BRD7P2	
DMR3:169831001	3	169831001	1000	1	1.90E-08	-0.6654574	22	2.2	LRRIQ4;LRRC31	Cytoskeleton
DMR3:169916001	3	169916001	1000	1	2.97E-07	0.6705372	16	1.6	SAMD7	Epigenetic
DMR3:170376001	3	170376001	2000	1	5.60E-06	-0.5859268	31	1.55	SKIL	
DMR3:170404001	3	170404001	1000	1	2.29E-06	-0.7307418	14	1.4	SKIL	
DMR3:171101001	3	171101001	1000	1	9.41E-06	0.538839	14	1.4	TNIK;LOC105374216;MIR569	Signaling
DMR3:171125001	3	171125001	2000	1	2.51E-06	0.7235677	21	1.05	TNIK;LOC105374216	Signaling
DMR3:172009001	3	172009001	1000	1	8.57E-06	0.6598807	11	1.1		
DMR3:174564001	3	174564001	1000	1	8.67E-09	0.8229764	6	0.6	NAALADL2	Protease
DMR3:174885001	3	174885001	1000	1	8.60E-07	-0.6464099	7	0.7	NAALADL2;LOC107986027	Protease
DMR3:176946001	3	176946001	1000	1	2.22E-06	0.6707394	15	1.5		
DMR3:177217001	3	177217001	1000	1	8.05E-06	-0.6935723	15	1.5	LOC107986053	
DMR3:177369001	3	177369001	1000	1	5.87E-08	-0.6441805	21	2.1		
DMR3:177501001	3	177501001	1000	1	5.09E-06	-1.0041934	12	1.2	LINC00578;RN7SKP52	
DMR3:179212001	3	179212001	1000	1	4.55E-06	-0.7611619	22	2.2	PIK3CA	Signaling
DMR3:181897001	3	181897001	1000	1	9.41E-08	-0.6074667	46	4.6		
DMR3:182988001	3	182988001	1000	1	4.22E-06	-0.5803972	26	2.6	DCUN1D1;LOC105374246	
DMR3:184210001	3	184210001	1000	1	3.28E-11	-0.745206	16	1.6		
DMR3:186555001	3	186555001	1000	1	4.71E-06	0.5480265	16	1.6	TBCCD1	
DMR3:189841001	3	189841001	2000	1	9.04E-06	1.0376044	25	1.25	TP63	Transcription
DMR3:191006001	3	191006001	2000	1	5.75E-06	-0.8597795	10	0.5		
DMR3:191016001	3	191016001	1000	1	7.56E-07	0.8399362	11	1.1		
DMR3:191316001	3	191316001	1000	1	6.31E-06	0.6982152	26	2.6	UTS2B	
DMR3:192072001	3	192072001	1000	1	3.17E-06	0.5140496	13	1.3		
DMR3:193417001	3	193417001	2000	1	1.07E-07	-0.6403365	42	2.1	ATP13A4	
DMR3:193889001	3	193889001	1000	1	8.81E-06	-0.7938	12	1.2	LOC105374287;LOC105374286	
DMR3:193999001	3	193999001	1000	1	6.93E-07	0.7429308	14	1.4	LINC02026;DPPA2P3;LINC02028	
DMR3:194324001	3	194324001	4000	1	1.23E-07	-0.5417791	69	1.725		
DMR3:195982001	3	195982001	1000	1	8.34E-06	0.5991301	28	2.8	SDHAP1	
DMR3:196161001	3	196161001	1000	1	1.14E-06	-0.8230313	15	1.5	LINC00885	
DMR3:196694001	3	196694001	2000	1	6.86E-06	-0.5174411	23	1.15		
DMR3:197034001	3	197034001	2000	1	1.15E-08	1.0122303	17	0.85	MELTF;DLG1	Transport;Cytoskeleton
DMR3:197300001	3	197300001	2000	1	4.66E-06	-0.6096042	34	1.7	DLG1;MIR4797;DLG1-AS1	Cytoskeleton
DMR3:197759001	3	197759001	3000	1	1.25E-06	0.4828514	29	0.966666667	RUBCN;FYTTD1	
DMR3:197806001	3	197806001	1000	1	1.45E-06	-1.0052817	13	1.3	LRCH3	
DMR3:197910001	3	197910001	2000	1	2.78E-06	-0.8294309	25	1.25	IQCG	Cytoskeleton
DMR4:10001	4	10001	1000	1	6.66E-06	-0.4926437	120	12		
DMR4:926001	4	926001	2000	1	1.79E-06	-0.5923215	110	5.5	GAK;TMEM175	Transport;Transport
DMR4:1042001	4	1042001	1000	1	4.56E-08	-0.6184093	24	2.4	LOC105374343	
DMR4:1651001	4	1651001	2000	1	1.38E-06	-0.4589415	51	2.55	FAM53A	
DMR4:2253001	4	2253001	1000	1	5.54E-06	0.7560433	28	2.8	MXD4;MIR4800	Transcription
DMR4:2524001	4	2524001	2000	1	6.62E-06	-0.5281725	55	2.75	RNF4;FAM193A	
DMR4:4038001	4	4038001	3000	1	3.79E-07	-0.8691217	38	1.266666667	LOC101928217	
DMR4:4470001	4	4470001	1000	1	4.33E-06	0.9246811	11	1.1	STX18;STX18-IT1	Transcription
DMR4:6299001	4	6299001	1000	1	8.12E-08	-0.6176038	26	2.6	WFS1;LOC107986257	
DMR4:6953001	4	6953001	1000	1	7.41E-06	-0.5138456	44	4.4	TBC1D14	Signaling
DMR4:7129001	4	7129001	3000	1	5.99E-07	-0.4701101	64	2.133333333		
DMR4:7229001	4	7229001	2000	1	5.48E-07	0.6253334	30	1.5	SORCS2	Transport
DMR4:9745001	4	9745001	2000	1	2.11E-06	0.6593329	34	1.7	LOC100420821;OR7E35P	
DMR4:15670001	4	15670001	1000	1	1.32E-06	-1.0156088	8	0.8	FBXL5;FAM200B	
DMR4:17461001	4	17461001	1000	1	6.10E-06	-0.7413936	13	1.3		
DMR4:21996001	4	21996001	1000	1	5.44E-06	0.745309	12	1.2		
DMR4:25752001	4	25752001	1000	1	1.52E-07	-0.6664294	17	1.7	SEL1L3	
DMR4:26283001	4	26283001	1000	1	8.67E-06	-0.802975	26	2.6	RBPJ;LOC105374542	Transcription
DMR4:26384001	4	26384001	1000	1	1.81E-08	-1.2129787	7	0.7	RBPJ	Transcription
DMR4:28906001	4	28906001	1000	1	1.21E-06	0.8589187	6	0.6		
DMR4:29455001	4	29455001	1000	1	3.55E-06	-0.8624318	15	1.5	LOC100533708	
DMR4:29898001	4	29898001	1000	1	9.73E-07	0.8348542	6	0.6		
DMR4:30499001	4	30499001	1000	1	1.57E-08	1.176533	4	0.4		
DMR4:33251001	4	33251001	1000	1	3.43E-08	0.640748	33	3.3		
DMR4:38135001	4	38135001	3000	1	1.85E-06	0.7664365	62	2.066666667	TBC1D1	Signaling
DMR4:39628001	4	39628001	1000	1	2.72E-07	-0.759066	27	2.7	SMIM14;RNU7-11P	
DMR4:39795001	4	39795001	1000	1	1.29E-06	-0.7774046	13	1.3		
DMR4:39807001	4	39807001	3000	1	1.21E-06	-0.5024734	68	2.266666667		
DMR4:39811001	4	39811001	4000	1	3.61E-07	-0.4830879	43	1.075	PDS5A	Epigenetic

DMR4:39984001	4	39984001	2000	1	3.53E-07	-0.8177322	54	2.7	PDS5A;PABPC1P1	Epigenetic
DMR4:40593001	4	40593001	1000	1	8.80E-06	-0.5145387	43	4.3	RBM47	Metabolism
DMR4:40890001	4	40890001	1000	1	1.03E-06	0.5247069	24	2.4	APBB2	
DMR4:42256001	4	42256001	2000	1	6.47E-07	-0.7537934	47	2.35		
DMR4:42893001	4	42893001	1000	1	2.16E-06	0.7355032	12	1.2	RN7SKP82;GRXCR1	Metabolism
DMR4:47306001	4	47306001	1000	1	7.43E-06	-0.6622573	3	0.3	GABRB1	Ion Channel
DMR4:48702001	4	48702001	1000	1	1.68E-06	-0.557823	23	2.3	FRYL	Cytoskeleton
DMR4:49601001	4	49601001	1000	1	8.61E-07	-0.5539822	1	0.1		
DMR4:49709001	4	49709001	3000	3	3.02E-21	-1.0965085	11	0.366666667		
DMR4:51107001	4	51107001	1000	1	2.77E-20	-1.1001505	7	0.7		
DMR4:51561001	4	51561001	1000	1	3.71E-06	0.7271246	10	1		
DMR4:52667001	4	52667001	1000	1	2.61E-07	0.7046809	9	0.9	USP46;USP46-DT	Protease
DMR4:55067001	4	55067001	1000	1	4.80E-06	-0.7094521	7	0.7	RN7SL424P	
DMR4:58291001	4	58291001	5000	1	5.44E-06	0.4932087	40	0.8		
DMR4:60703001	4	60703001	1000	1	5.74E-07	-0.7881465	29	2.9		
DMR4:62809001	4	62809001	1000	1	1.18E-06	-0.9044958	30	3	EXOC5P1	
DMR4:62911001	4	62911001	1000	1	1.20E-06	0.7311677	8	0.8		
DMR4:64365001	4	64365001	1000	1	1.28E-06	0.971092	7	0.7	TECRL	Metabolism
DMR4:64607001	4	64607001	2000	1	1.12E-06	0.5560372	31	1.55	MTCO3P27;MTCYBP16;MTND6P16;MTND5P13;MTCO3P28	
DMR4:65751001	4	65751001	1000	1	7.47E-06	-0.7216537	5	0.5		
DMR4:66657001	4	66657001	1000	1	4.07E-08	-0.5376613	25	2.5		
DMR4:72548001	4	72548001	1000	1	2.05E-06	0.9233553	15	1.5	ADAMTS3	Protease
DMR4:73543001	4	73543001	1000	1	1.76E-09	-1.5587119	17	1.7	LOC107986287	
DMR4:75627001	4	75627001	3000	1	5.46E-06	-0.6372171	64	2.133333333	CDKL2	Signaling
DMR4:76598001	4	76598001	2000	1	1.85E-07	0.670039	27	1.35	SHROOM3	Cytoskeleton
DMR4:77115001	4	77115001	1000	1	1.63E-06	-0.8271014	8	0.8	LOC339966	
DMR4:78535001	4	78535001	1000	1	1.08E-06	0.6554399	11	1.1	FRAS1	
DMR4:80400001	4	80400001	1000	1	4.01E-06	0.8518597	7	0.7	CFAP299	Development
DMR4:81594001	4	81594001	1000	1	2.52E-06	-0.6794902	4	0.4		
DMR4:81762001	4	81762001	1000	1	1.29E-06	0.6475292	9	0.9		
DMR4:83130001	4	83130001	1000	1	8.45E-06	-0.4564692	32	3.2		
DMR4:83312001	4	83312001	1000	1	5.67E-07	-0.5773284	35	3.5	HPSE;LOC105377313	Metabolism
DMR4:85255001	4	85255001	1000	1	4.90E-07	-0.8483622	10	1	LOC100526736	
DMR4:87308001	4	87308001	1000	1	3.03E-06	-0.5773017	9	0.9	MIR5705;HSD17B13	
DMR4:87614001	4	87614001	2000	2	1.96E-07	1.2428054	27	1.35	DSPP	
DMR4:92388001	4	92388001	1000	1	3.07E-08	1.1678303	11	1.1	GRID2	Receptor
DMR4:93984001	4	93984001	1000	1	3.28E-06	-0.6989411	6	0.6		
DMR4:94340001	4	94340001	1000	1	2.10E-06	-0.4342945	41	4.1	HPGDS	Transport
DMR4:99014001	4	99014001	3000	1	7.60E-08	0.9569408	44	1.466666667	METAP1;ABT1P1	Protease
DMR4:100645001	4	100645001	1000	1	2.86E-10	-1.3968127	2	0.2		
DMR4:102179001	4	102179001	1000	1	5.52E-06	0.7241327	8	0.8		
DMR4:104453001	4	104453001	1000	1	2.73E-06	-0.5699652	9	0.9		
DMR4:108066001	4	108066001	1000	1	7.21E-08	0.9251455	15	1.5	LEF1	Transcription
DMR4:109926001	4	109926001	1000	1	1.99E-06	-0.8559054	26	2.6	EGF	Receptor
DMR4:110233001	4	110233001	1000	1	4.23E-06	0.515324	11	1.1		
DMR4:111731001	4	111731001	1000	1	8.87E-06	-0.7475914	5	0.5		
DMR4:112665001	4	112665001	1000	1	1.45E-06	-0.716884	21	2.1	LARP7	Metabolism
DMR4:114640001	4	114640001	1000	1	7.85E-06	-0.6325816	26	2.6	UGT8	
DMR4:114845001	4	114845001	1000	1	3.19E-07	0.7725911	13	1.3	NDST4	Transport
DMR4:116330001	4	116330001	1000	1	3.65E-06	-0.8770676	30	3		
DMR4:116980001	4	116980001	1000	1	2.37E-07	0.6440341	17	1.7		
DMR4:120892001	4	120892001	1000	1	2.67E-06	0.7955501	10	1	PRDM5;SETP12	Transcription
DMR4:124651001	4	124651001	1000	1	8.97E-06	0.4813155	30	3		
DMR4:128706001	4	128706001	2000	1	5.75E-09	-0.7429226	56	2.8		
DMR4:128784001	4	128784001	1000	1	4.08E-11	-0.7641468	34	3.4		
DMR4:130010001	4	130010001	2000	1	4.49E-09	-1.254595	21	1.05		
DMR4:134078001	4	134078001	2000	1	3.06E-06	-0.7203284	34	1.7	PABPC4L	
DMR4:136374001	4	136374001	1000	1	3.46E-08	-0.9140132	15	1.5		
DMR4:137223001	4	137223001	1000	1	7.64E-08	0.6185274	16	1.6		
DMR4:139165001	4	139165001	2000	1	2.34E-06	-0.6883266	26	1.3	ELF2	Transcription
DMR4:139783001	4	139783001	2000	1	5.28E-07	0.7463348	40	2	MAML3;RN7SKP253	Transcription
DMR4:139843001	4	139843001	1000	1	2.45E-06	0.745194	12	1.2	MAML3	Transcription
DMR4:140287001	4	140287001	2000	1	7.23E-08	0.8620504	9	0.45	SCOC;SCOC-AS1	
DMR4:141115001	4	141115001	1000	1	2.25E-06	0.9925063	5	0.5	RNF150	
DMR4:141580001	4	141580001	1000	1	5.37E-06	-0.8495613	3	0.3	LINC02276	
DMR4:143271001	4	143271001	3000	2	2.12E-07	-0.9479347	29	0.966666667		
DMR4:143686001	4	143686001	1000	1	2.69E-06	0.5726923	10	1	FREM3	
DMR4:147531001	4	147531001	2000	1	2.93E-06	-0.7399817	33	1.65	EDNRA	

DMR4:148322001	4	148322001	2000	1	4.24E-06	0.5911889	49	2.45	NR3C2	
DMR4:149557001	4	149557001	1000	1	8.93E-09	0.7708592	10	1	IQCM	
DMR4:150489001	4	150489001	1000	1	3.63E-39	-3.472153	3	0.3	LRBA	
DMR4:151075001	4	151075001	1000	1	8.96E-07	-0.7741677	19	1.9		
DMR4:151353001	4	151353001	2000	1	2.61E-06	0.7373767	31	1.55		
DMR4:154370001	4	154370001	2000	1	1.21E-07	0.8627043	14	0.7	DCHS2;LOC100419960	Cytoskeleton
DMR4:157275001	4	157275001	1000	1	2.15E-09	1.1008433	8	0.8	GRIA2	Receptor
DMR4:159355001	4	159355001	3000	1	7.78E-06	0.6768816	44	1.466666667	RAPGEF2	Transcription
DMR4:160832001	4	160832001	1000	1	3.47E-08	-0.6966268	25	2.5		
DMR4:161877001	4	161877001	1000	1	4.33E-06	-0.5616918	33	3.3	FSTL5	Protease; Proteolysis
DMR4:168155001	4	168155001	1000	1	9.74E-19	-2.1028232	4	0.4	ANXA10	Signaling
DMR4:168181001	4	168181001	2000	1	2.24E-06	-0.5652889	62	3.1	ANXA10;LOC105377524	Signaling
DMR4:176476001	4	176476001	1000	1	7.20E-06	0.7533874	16	1.6		
DMR4:180566001	4	180566001	2000	1	5.78E-08	-0.4572788	14	0.7	NDUFB5P1	
DMR4:182792001	4	182792001	1000	1	8.81E-13	0.8797694	23	2.3	TENM3	
DMR4:182810001	4	182810001	1000	1	3.13E-07	-0.4331547	5	0.5	TENM3	
DMR4:183857001	4	183857001	1000	1	3.59E-06	0.6554024	12	1.2	STOX2	
DMR4:184965001	4	184965001	3000	1	9.76E-06	-0.5062821	189	6.3		
DMR4:186054001	4	186054001	4000	2	4.14E-09	-0.78751	107	2.675		
DMR4:186684001	4	186684001	1000	1	4.45E-07	0.9686371	19	1.9	FAT1	Cytoskeleton
DMR4:188508001	4	188508001	1000	1	8.48E-07	0.6137977	7	0.7	LINC01060	
DMR4:189048001	4	189048001	1000	1	2.11E-07	-0.7391657	26	2.6	LOC105377611;LOC105377612	
DMR4:189552001	4	189552001	2000	1	7.69E-07	0.778833	57	2.85	LOC105377615	
DMR4:189645001	4	189645001	2000	2	1.67E-06	-0.5142464	155	7.75		
DMR4:189677001	4	189677001	1000	1	1.14E-06	0.5486429	15	1.5		
DMR4:189805001	4	189805001	1000	1	1.35E-06	0.6271455	22	2.2	FRG1-DT;LOC105377619	
DMR5:344001	5	344001	3000	2	1.20E-12	-0.9015777	117	3.9	PDCD6-AHRR;AHRR	Transcription
DMR5:495001	5	495001	1000	1	2.21E-06	-0.7571434	108	10.8	SLC9A3	Transport
DMR5:507001	5	507001	1000	1	4.23E-06	-0.5618055	55	5.5	SLC9A3	Transport
DMR5:1098001	5	1098001	1000	1	4.07E-07	-0.7040424	13	1.3	SLC12A7	Transport
DMR5:1191001	5	1191001	4000	1	3.15E-06	-0.4435151	104	2.6	SLC6A19	Transport
DMR5:1616001	5	1616001	1000	1	2.72E-06	0.6171301	11	1.1	LOC728613;LOC100132773;LOC112267946	
DMR5:1866001	5	1866001	2000	1	9.57E-08	0.757994	62	3.1	LINC02116;IRX4	Development
DMR5:2489001	5	2489001	3000	1	7.80E-06	0.6201903	71	2.366666667		
DMR5:3001001	5	3001001	3000	1	6.17E-07	0.6790058	51	1.7		
DMR5:3078001	5	3078001	1000	1	1.28E-06	0.6820814	24	2.4		
DMR5:3483001	5	3483001	2000	1	9.86E-07	0.7296504	14	0.7	LINC01019	
DMR5:3508001	5	3508001	1000	1	2.68E-08	-0.9597969	6	0.6	LINC01019;LINC01017	
DMR5:4207001	5	4207001	1000	1	2.90E-06	0.6334617	32	3.2		
DMR5:4973001	5	4973001	2000	1	7.16E-07	0.5182349	12	0.6	LOC105374630	
DMR5:6446001	5	6446001	3000	2	6.61E-07	-0.5520203	106	3.533333333	UBE2QL1;LOC105374639	Proteolysis
DMR5:6728001	5	6728001	1000	1	1.85E-07	0.7283283	18	1.8	TENT4A	Translation
DMR5:7126001	5	7126001	1000	1	4.54E-06	0.9332008	10	1	LINC02196	
DMR5:7522001	5	7522001	1000	1	7.41E-06	-0.5242714	18	1.8	ADCY2	
DMR5:8058001	5	8058001	2000	1	5.09E-06	0.6063575	46	2.3	LOC102723339	
DMR5:13793001	5	13793001	1000	1	4.21E-06	0.7717868	9	0.9	DNAH5	Cytoskeleton
DMR5:14140001	5	14140001	1000	1	2.25E-06	-0.6246897	27	2.7	TRIO	Transcription
DMR5:14402001	5	14402001	4000	1	1.83E-06	-0.5195445	53	1.325	TRIO	Transcription
DMR5:15766001	5	15766001	1000	1	2.40E-06	0.7961497	11	1.1	FBXL7	
DMR5:16620001	5	16620001	2000	1	6.96E-06	1.0770764	17	0.85	RETREG1;RETREG1-AS1	
DMR5:17389001	5	17389001	2000	1	2.22E-11	-0.7374556	61	3.05	LINC02111	
DMR5:20776001	5	20776001	1000	1	5.82E-06	-0.6083409	15	1.5	LINC02241	
DMR5:21051001	5	21051001	1000	1	1.16E-06	-0.5179813	33	3.3	LOC105374678	
DMR5:21650001	5	21650001	2000	1	3.99E-08	-0.7544657	27	1.35	LOC105374685	
DMR5:25314001	5	25314001	1000	1	2.55E-06	-0.6987199	25	2.5		
DMR5:26235001	5	26235001	2000	1	1.30E-11	-0.6410874	33	1.65		
DMR5:28927001	5	28927001	1000	1	8.45E-06	0.8104662	59	5.9	LSP1P3	
DMR5:29090001	5	29090001	1000	1	5.95E-06	-0.7125872	14	1.4	LOC105374699	
DMR5:31226001	5	31226001	3000	1	5.29E-06	0.7083141	42	1.4	CDH6	Cytoskeleton
DMR5:31698001	5	31698001	1000	1	3.79E-09	-0.637476	32	3.2	PDZD2	Cytokine
DMR5:32552001	5	32552001	1000	1	1.30E-07	0.854696	16	1.6	SUB1	Transcription
DMR5:34085001	5	34085001	1000	1	6.15E-06	-0.8045716	14	1.4	C1QTNF3-AMACR;C1QTNF3	
DMR5:34438001	5	34438001	2000	1	5.67E-07	-0.6355517	30	1.5		
DMR5:34606001	5	34606001	1000	1	1.47E-06	-0.6462779	16	1.6		
DMR5:34799001	5	34799001	2000	1	1.72E-06	-0.6972618	28	1.4	RAI14	
DMR5:35713001	5	35713001	1000	1	2.13E-12	-1.8037978	9	0.9	SPEF2;LOC105374724	
DMR5:36138001	5	36138001	1000	1	2.72E-06	0.6777994	10	1	LMBRD2;MIR580	

DMR5:37293001	5	37293001	1000	1	3.10E-06	-0.6582756	24	2.4	NUP155	Transport
DMR5:39143001	5	39143001	1000	1	3.87E-08	1.0706419	3	0.3	FYB1	Cytoskeleton
DMR5:42860001	5	42860001	2000	1	2.60E-07	0.6959205	16	0.8		
DMR5:43377001	5	43377001	2000	1	3.11E-07	-0.6334055	41	2.05	CCL28	Growth Factors
DMR5:44779001	5	44779001	1000	1	1.34E-06	-1.0602149	12	1.2	MRPS30-DT	
DMR5:46433001	5	46433001	3000	2	6.20E-08	0.6189204	37	1.233333333		
DMR5:47526001	5	47526001	1000	1	2.78E-06	-1.1015763	23	2.3		
DMR5:48234001	5	48234001	1000	1	8.47E-06	-0.8828559	20	2		
DMR5:48791001	5	48791001	1000	1	2.72E-06	-1.1529135	17	1.7		
DMR5:48850001	5	48850001	1000	1	9.35E-10	-1.1070861	17	1.7		
DMR5:49385001	5	49385001	1000	1	2.61E-06	-1.0485601	20	2		
DMR5:49609001	5	49609001	10000	9	7.89E-11	-0.5598351	156	1.56		
DMR5:49621001	5	49621001	7000	6	1.93E-11	-0.5408733	117	1.671428571		
DMR5:49630001	5	49630001	4000	1	7.11E-07	-0.5666644	48	1.2		
DMR5:49642001	5	49642001	7000	4	3.17E-08	-0.5281371	114	1.628571429		
DMR5:49858001	5	49858001	1000	1	6.69E-06	0.8906798	17	1.7		
DMR5:49871001	5	49871001	1000	1	1.62E-07	1.0583676	20	2		
DMR5:50645001	5	50645001	1000	1	1.32E-37	-4.6924485	1	0.1		
DMR5:51635001	5	51635001	1000	1	4.90E-06	-0.9962163	4	0.4		
DMR5:53813001	5	53813001	1000	1	1.62E-06	0.8178702	7	0.7	LINC02105	
DMR5:55106001	5	55106001	1000	1	9.74E-06	-0.6984	4	0.4	GZMAP1;GZMA;CDC20B	Protease;Proteolysis
DMR5:55543001	5	55543001	1000	1	7.04E-06	-0.6829865	15	1.5	PLPP1;RNF138P1;AK4P2	Signaling
DMR5:56026001	5	56026001	1000	1	1.81E-07	-0.7097209	32	3.2		
DMR5:57899001	5	57899001	1000	1	2.48E-06	-0.652508	18	1.8	LINC02225	
DMR5:58483001	5	58483001	1000	1	5.10E-06	0.5555579	15	1.5	GAPT	
DMR5:60857001	5	60857001	1000	1	1.73E-06	-0.5584984	38	3.8	ERCC8	Transcription
DMR5:61045001	5	61045001	1000	1	2.61E-06	-1.070708	3	0.3	NDUFAF2	Transcription
DMR5:61696001	5	61696001	3000	1	7.06E-08	0.8432189	48	1.6	C5orf64;LOC101928651	
DMR5:66050001	5	66050001	1000	1	7.56E-07	-1.097891	27	2.7	ERBIN	Cytoskeleton
DMR5:68130001	5	68130001	1000	1	2.46E-15	-1.0669503	11	1.1		
DMR5:69548001	5	69548001	1000	1	5.28E-06	-0.7936316	18	1.8	OCLN;SNORD13B-1	Transcription
DMR5:70435001	5	70435001	1000	1	2.32E-06	-0.6957974	23	2.3	LOC107986356;GTF2H2B	
DMR5:70449001	5	70449001	1000	1	1.00E-07	-0.9054217	15	1.5	LOC107986356;GTF2H2B;NAIPP1	
DMR5:70954001	5	70954001	2000	1	6.91E-07	-0.5967678	31	1.55	SMN1	
DMR5:76786001	5	76786001	1000	1	2.52E-07	-0.5002547	36	3.6		
DMR5:78466001	5	78466001	2000	1	2.08E-06	0.4968098	14	0.7	SCAMP1	Transport
DMR5:79628001	5	79628001	1000	1	5.42E-06	0.8207955	4	0.4	TENT2	Metabolism
DMR5:81075001	5	81075001	1000	1	6.96E-06	0.6635591	10	1	RASGRF2	Transcription
DMR5:81949001	5	81949001	2000	1	4.18E-06	0.7893366	21	1.05		
DMR5:84248001	5	84248001	1000	1	5.06E-07	1.1420282	6	0.6	EDIL3	Metabolism
DMR5:86770001	5	86770001	1000	1	4.09E-09	-0.8607604	8	0.8		
DMR5:89050001	5	89050001	1000	1	7.11E-06	0.6070458	14	1.4	MEF2C-AS1	
DMR5:90886001	5	90886001	1000	1	2.67E-06	0.9043042	10	1	ADGRV1	Signaling
DMR5:91318001	5	91318001	1000	1	4.23E-06	-0.6789807	18	1.8	LUCAT1	
DMR5:92185001	5	92185001	1000	1	4.47E-07	-0.7325871	22	2.2		
DMR5:94045001	5	94045001	1000	1	2.01E-07	-0.6903807	3	0.3	FAM172A	
DMR5:94108001	5	94108001	1000	1	7.22E-06	-0.7238352	6	0.6	FAM172A;LOC105379087	
DMR5:95969001	5	95969001	1000	1	6.60E-06	0.553609	7	0.7	ELL2;LOC101929710;FABP5P5	Transcription
DMR5:98409001	5	98409001	3000	1	5.18E-06	0.7229104	49	1.633333333	MRPS35P2;MTCO2P24;MTCO1P24	
DMR5:104788001	5	104788001	1000	1	2.35E-06	-0.7103471	6	0.6	RNU6-334P	
DMR5:114703001	5	114703001	1000	1	7.55E-07	0.7298573	25	2.5	LOC101927078	
DMR5:120303001	5	120303001	1000	1	2.36E-06	-0.763984	9	0.9		
DMR5:120331001	5	120331001	1000	1	3.18E-07	-0.5967769	34	3.4	RNU6-718P	
DMR5:122940001	5	122940001	1000	1	2.36E-07	0.5277784	14	1.4	SNX24	Cytoskeleton
DMR5:123679001	5	123679001	1000	1	6.60E-07	0.7262022	16	1.6		
DMR5:126227001	5	126227001	1000	1	6.24E-06	0.4473199	13	1.3		
DMR5:126720001	5	126720001	2000	1	1.82E-11	-0.822166	32	1.6		
DMR5:128595001	5	128595001	1000	1	9.60E-07	0.8513043	8	0.8		
DMR5:128688001	5	128688001	1000	1	5.36E-07	-0.8578894	7	0.7	LOC105379168	
DMR5:133524001	5	133524001	2000	1	3.72E-06	0.704066	31	1.55	FSTL4	Protease; Proteolysis
DMR5:134480001	5	134480001	5000	1	6.99E-06	-0.4409575	139	2.78		
DMR5:134556001	5	134556001	1000	1	9.00E-09	-0.4612931	11	1.1	JADE2	Transcription
DMR5:134596001	5	134596001	1000	1	2.26E-06	-0.5507672	23	2.3	SAR1B	
DMR5:135265001	5	135265001	2000	1	5.76E-06	0.5728606	35	1.75	PITX1-AS1	
DMR5:138019001	5	138019001	1000	1	2.20E-06	-0.7832717	15	1.5	FAM13B	
DMR5:138108001	5	138108001	1000	1	1.79E-14	-0.824425	32	3.2	NME5;RNU6-460P	Signaling
DMR5:138134001	5	138134001	2000	1	2.18E-06	-0.7644419	62	3.1	NME5;BRD8	Signaling
DMR5:138550001	5	138550001	2000	1	1.23E-10	-0.6524084	47	2.35	ETF1;HSPA9;SNORD63B;SNORD63	Translation

DMR5:138562001	5	138562001	1000	1	1.75E-06	-0.7940666	23	2.3	HSPA9;SNORD63B;SNORD63;LOC105379193	
DMR5:139368001	5	139368001	1000	1	3.83E-07	-0.6091548	25	2.5	PAIP2;SLC23A1	Transport
DMR5:139451001	5	139451001	4000	1	1.06E-06	-0.4523882	18	0.45	RNU5B-4P;ECSCR	
DMR5:141873001	5	141873001	1000	1	6.85E-06	-0.5509889	32	3.2	PCDH1	Cytoskeleton
DMR5:142083001	5	142083001	1000	1	1.47E-06	-0.6499183	19	1.9	LOC105378204	
DMR5:142588001	5	142588001	1000	1	7.04E-06	-0.4662332	21	2.1	FGF1	Growth Factors
DMR5:149650001	5	149650001	1000	1	4.13E-45	-4.6730181	0	0		
DMR5:154878001	5	154878001	3000	1	2.80E-07	0.8351278	43	1.433333333	CNOT8;GEMIN5	Translation
DMR5:158608001	5	158608001	1000	1	9.84E-06	0.4891744	6	0.6		
DMR5:160252001	5	160252001	2000	1	7.37E-07	0.687352	45	2.25	CCNJL;LOC727947;LOC105377691	Signaling
DMR5:167427001	5	167427001	1000	1	1.22E-06	-0.561371	23	2.3	TENM2	
DMR5:170519001	5	170519001	1000	1	5.17E-06	0.8628384	9	0.9	KCNIP1	
DMR5:171856001	5	171856001	1000	1	7.93E-06	0.7909206	15	1.5	LOC107986476;FBXW11	
DMR5:172275001	5	172275001	1000	1	9.92E-06	0.779724	13	1.3	UBTD2;LOC100288254	Proteolysis
DMR5:172596001	5	172596001	2000	1	1.63E-07	-0.4655697	46	2.3	LOC105377729	
DMR5:176870001	5	176870001	3000	3	2.91E-09	-0.6258146	63	2.1	UNC5A;HK3	Receptor;Signaling
DMR5:178100001	5	178100001	1000	1	2.86E-06	-0.6773249	22	2.2	LOC105377754	
DMR5:178179001	5	178179001	1000	1	8.60E-06	-0.6498854	12	1.2	GMCL2	Proteolysis
DMR5:178203001	5	178203001	1000	1	1.36E-06	-0.5649924	58	5.8	HNRNPAB;PHYKPL	
DMR5:178709001	5	178709001	1000	1	5.53E-06	0.6650276	13	1.3	MSANTD5;ZNF354A	
DMR5:179061001	5	179061001	1000	1	3.81E-07	0.617565	35	3.5	ZNF354C	
DMR5:179411001	5	179411001	2000	1	2.13E-08	-0.7380081	67	3.35		
DMR5:179564001	5	179564001	1000	1	3.52E-07	-0.7729462	11	1.1	RUFY1;PRDX2P3	
DMR5:179629001	5	179629001	1000	1	7.72E-06	-0.6741495	31	3.1	HNRNPH1	Translation
DMR5:179725001	5	179725001	2000	1	8.29E-07	-0.6411553	52	2.6	CANX;MAML1	Transcription;Transcription
DMR6:906001	6	906001	1000	1	5.53E-18	-2.4777635	2	0.2		
DMR6:3529001	6	3529001	3000	1	2.05E-06	0.865059	44	1.466666667		
DMR6:3969001	6	3969001	2000	1	9.35E-06	0.7101413	29	1.45	GLRX3P2	
DMR6:4266001	6	4266001	1000	1	2.38E-08	0.948177	6	0.6		
DMR6:7074001	6	7074001	1000	1	7.93E-06	-0.8452056	13	1.3		
DMR6:7676001	6	7676001	1000	1	4.85E-07	-0.7595407	10	1	LOC105374906	
DMR6:7750001	6	7750001	1000	1	6.61E-06	0.8697464	10	1	BMP6	Growth Factors
DMR6:10541001	6	10541001	1000	1	1.07E-08	0.9195344	19	1.9	GCNT2	Golgi
DMR6:10751001	6	10751001	1000	1	1.71E-07	1.1345971	19	1.9	TMEM14B;RNA5SP203	
DMR6:11103001	6	11103001	2000	1	2.00E-06	0.765866	41	2.05	SMIM13;ERVFRD-1	Epigenetic
DMR6:11173001	6	11173001	1000	1	1.64E-06	-0.684396	7	0.7	NEDD9	
DMR6:11282001	6	11282001	1000	1	7.44E-07	0.6050631	11	1.1	NEDD9;LOC105374925	
DMR6:11492001	6	11492001	1000	1	1.08E-07	0.834156	10	1	LOC105374928	
DMR6:12761001	6	12761001	1000	1	2.35E-07	0.9453796	8	0.8	PHACTR1	Signaling
DMR6:14539001	6	14539001	1000	1	8.61E-06	0.9215752	12	1.2	LOC101928354	
DMR6:14921001	6	14921001	2000	1	1.39E-07	1.0621163	39	1.95	LOC105374945	
DMR6:15451001	6	15451001	2000	1	2.26E-06	0.5628422	25	1.25	JARID2	Epigenetic
DMR6:16749001	6	16749001	2000	1	4.82E-06	0.5980324	27	1.35	ATXN1	
DMR6:17532001	6	17532001	1000	1	3.33E-06	-0.5299207	22	2.2	CAP2;RPL7P26	Cytoskeleton
DMR6:18583001	6	18583001	2000	1	3.06E-07	1.0321517	14	0.7	MIR548A1HG	
DMR6:21091001	6	21091001	2000	1	9.30E-06	-0.6849333	30	1.5	CDKAL1	
DMR6:21377001	6	21377001	1000	1	7.85E-06	0.6554785	12	1.2		
DMR6:21892001	6	21892001	1000	1	8.45E-07	-0.5451289	15	1.5	CASC15	
DMR6:22032001	6	22032001	1000	1	5.62E-06	-0.5576867	11	1.1	CASC15	
DMR6:24210001	6	24210001	1000	1	5.77E-06	0.6325535	3	0.3	DCDC2	
DMR6:24339001	6	24339001	1000	1	5.88E-07	0.9158617	8	0.8	DCDC2	
DMR6:28755001	6	28755001	2000	1	9.60E-10	-1.0366755	33	1.65	TRK-TTT7-1;TRA-TGC6-1	
DMR6:29247001	6	29247001	1000	1	4.33E-06	0.5731288	11	1.1	LOC101929006	
DMR6:29717001	6	29717001	2000	1	1.99E-06	-1.0737659	91	4.55	ZDHHC20P1;HCG4P11;HLA-F;RPL23AP1;HLA-F-AS1	Immune
DMR6:30870001	6	30870001	1000	1	7.30E-07	-0.9938057	10	1	RN7SKP186;DDR1	Receptor
DMR6:31105001	6	31105001	1000	1	1.66E-06	-0.702999	24	2.4	C6orf15;PSORS1C1;CDSN	
DMR6:31116001	6	31116001	2000	1	8.20E-06	0.6904105	44	2.2	C6orf15;PSORS1C1;CDSN	
DMR6:31804001	6	31804001	1000	1	1.61E-08	-0.8031395	19	1.9	VARS1;LSM2;HSPA1L	Translation;Translation
DMR6:31832001	6	31832001	1000	1	1.55E-07	-0.6087266	25	2.5	HSPA1B;SNHG32;SNORD48;SNORD52	
DMR6:32308001	6	32308001	1000	1	1.02E-06	-0.6220729	24	2.4	TSBP1-AS1;TSBP1	
DMR6:33124001	6	33124001	2000	1	8.75E-06	-0.7660641	44	2.2	HLA-DPB2;LOC105375021;HLA-DPA3	
DMR6:33256001	6	33256001	2000	1	5.60E-06	-0.856972	19	0.95	LOC105375022;HCG25;VPS52	Transport
DMR6:33334001	6	33334001	2000	1	4.93E-06	-0.6531291	35	1.75	SMIM40;MYL12BP3	
DMR6:34070001	6	34070001	4000	1	9.71E-07	-0.4026982	46	1.15	GRM4	Signaling
DMR6:34662001	6	34662001	1000	1	2.68E-06	-0.6318511	23	2.3	ILRUN	

DMR6:37175001	6	37175001	1000	1	8.47E-06	0.7722313	13	1.3	PIM1	Signaling
DMR6:37420001	6	37420001	2000	1	4.05E-06	-0.626587	49	2.45		
DMR6:38166001	6	38166001	1000	1	3.46E-06	-0.539798	36	3.6	BTBD9	
DMR6:38264001	6	38264001	2000	1	6.18E-06	0.631414	24	1.2	BTBD9	
DMR6:40641001	6	40641001	1000	1	8.75E-06	1.0463951	10	1		
DMR6:44044001	6	44044001	2000	1	5.36E-06	-0.3819852	15	0.75	POLR1C;SCIRT	Transcription
DMR6:45051001	6	45051001	3000	1	6.88E-07	-0.9516637	24	0.8	SUPT3H	Transcription
DMR6:45121001	6	45121001	1000	1	3.03E-06	-0.840106	12	1.2	SUPT3H	Transcription
DMR6:53743001	6	53743001	1000	1	7.67E-06	-0.5904003	29	2.9		
DMR6:56974001	6	56974001	1000	1	2.29E-07	-0.8106341	5	0.5	BEND6;OSTCP6	
DMR6:57015001	6	57015001	1000	1	5.82E-06	-0.7372208	14	1.4	BEND6	
DMR6:57205001	6	57205001	2000	1	9.43E-06	0.4857397	13	0.65	RAB23	
DMR6:59256001	6	59256001	1000	1	8.36E-06	0.8057928	17	1.7		
DMR6:63079001	6	63079001	1000	1	2.76E-08	0.6698461	4	0.4		
DMR6:65849001	6	65849001	1000	1	6.61E-06	0.5962373	10	1		
DMR6:71880001	6	71880001	1000	1	5.62E-06	0.6353746	8	0.8	RIMS1	Transport
DMR6:73583001	6	73583001	2000	1	2.30E-06	-0.5253726	42	2.1	SLC17A5	Transport
DMR6:75655001	6	75655001	1000	1	1.21E-06	0.9199322	12	1.2	SENP6;RN7SKP163	Protease
DMR6:79341001	6	79341001	1000	1	1.82E-06	0.884524	4	0.4	LOC112267976	
DMR6:80050001	6	80050001	2000	1	5.13E-06	0.5359966	22	1.1	TTK	Signaling
DMR6:84965001	6	84965001	1000	1	1.09E-06	0.9368966	7	0.7	LOC105377881	
DMR6:87346001	6	87346001	1000	1	2.98E-06	0.7737394	7	0.7	SMIM8;C6orf163	
DMR6:88716001	6	88716001	1000	1	7.26E-08	-1.2601959	5	0.5	RNGTT	Translation
DMR6:101760001	6	101760001	1000	1	2.76E-14	-1.4692934	13	1.3	GRIK2	Receptor
DMR6:104361001	6	104361001	1000	1	5.09E-09	-0.6686165	28	2.8	LOC102724443	
DMR6:104990001	6	104990001	1000	1	5.02E-07	0.6079458	8	0.8	LIN28B	Metabolism
DMR6:106334001	6	106334001	2000	1	4.76E-06	-0.5926119	38	1.9	ATG5	Transport
DMR6:106687001	6	106687001	1000	1	3.92E-06	0.8634196	9	0.9	LINC02526	
DMR6:108273001	6	108273001	1000	1	2.64E-06	-0.4502858	24	2.4		
DMR6:110215001	6	110215001	2000	1	3.71E-08	1.0238288	24	1.2	CDC40	Translation
DMR6:110974001	6	110974001	1000	1	8.86E-07	-0.6084082	42	4.2	GTF3C6;RPF2	
DMR6:111309001	6	111309001	1000	1	7.32E-06	0.6691409	20	2	REV3L	Transcription
DMR6:111836001	6	111836001	2000	1	1.97E-06	0.6325777	27	1.35	FYN	
DMR6:111955001	6	111955001	3000	1	1.40E-06	0.6162772	35	1.166666667		
DMR6:112024001	6	112024001	1000	1	5.53E-06	0.6504052	8	0.8		
DMR6:116082001	6	116082001	1000	1	2.60E-06	0.8281328	3	0.3	FRK	
DMR6:117549001	6	117549001	1000	1	4.65E-06	0.9056137	13	1.3	DCBLD1	
DMR6:117821001	6	117821001	1000	1	2.10E-06	-0.6651752	25	2.5		
DMR6:119275001	6	119275001	1000	1	9.66E-06	-0.5066785	33	3.3	MAN1A1;RPL13AP15;LOC107986526	Golgi
DMR6:119279001	6	119279001	1000	1	4.23E-07	0.5371563	6	0.6	MAN1A1;RPL13AP15;LOC107986526	Golgi
DMR6:122055001	6	122055001	1000	1	4.64E-06	0.9546359	12	1.2	LOC105377979	
DMR6:125772001	6	125772001	1000	1	8.48E-06	-0.8693343	17	1.7	HEY2;TRE-CTC1-7;NCOA7	Transcription
DMR6:133105001	6	133105001	1000	1	4.44E-06	0.7434567	3	0.3	LINC00326	
DMR6:134471001	6	134471001	1000	1	1.71E-06	0.8466715	2	0.2	CT69;LINC01010	
DMR6:134482001	6	134482001	1000	1	4.57E-06	0.8215277	13	1.3	CT69;LINC01010	
DMR6:142910001	6	142910001	1000	1	3.60E-06	0.7292874	11	1.1	HIVEP2	
DMR6:143699001	6	143699001	1000	1	2.18E-06	0.7891229	11	1.1	PHACTR2	Signaling
DMR6:144819001	6	144819001	1000	1	5.20E-08	-0.9495033	9	0.9	UTRN	
DMR6:146314001	6	146314001	1000	1	2.26E-06	-0.851979	8	0.8	GRM1	Signaling
DMR6:147372001	6	147372001	1000	1	1.23E-08	-0.7287733	9	0.9	STXBP5	Transport
DMR6:148334001	6	148334001	1000	1	1.54E-10	-0.5734649	43	4.3	SASH1	
DMR6:148875001	6	148875001	4000	1	2.67E-08	-0.514432	133	3.325	UST	Transport
DMR6:149611001	6	149611001	1000	1	4.82E-06	-0.5477305	17	1.7	KATNA1	Cytoskeleton
DMR6:150715001	6	150715001	2000	1	2.11E-06	-0.5374587	49	2.45	PLEKHG1	
DMR6:154490001	6	154490001	4000	1	9.37E-06	0.7409971	38	0.95	CNKSR3	
DMR6:154724001	6	154724001	1000	1	5.67E-06	-0.5335803	36	3.6	SCAF8	Translation
DMR6:154914001	6	154914001	2000	1	7.74E-07	-0.5578125	49	2.45		
DMR6:157147001	6	157147001	1000	1	3.36E-10	-0.7356516	74	7.4	ARID1B	
DMR6:157255001	6	157255001	1000	1	5.66E-06	0.860535	11	1.1	LOC105378075	
DMR6:157310001	6	157310001	4000	1	5.25E-06	0.5519531	88	2.2	TMEM242;LDHAL6FP;LOC112267967	
DMR6:157416001	6	157416001	2000	1	8.26E-06	-0.6485256	40	2	ZDHHC14	
DMR6:157687001	6	157687001	2000	1	6.98E-10	-0.9311456	48	2.4	ZDHHC14	
DMR6:158020001	6	158020001	1000	1	8.08E-09	0.7693133	14	1.4	SYNJ2	Signaling
DMR6:158034001	6	158034001	3000	1	4.50E-06	0.8848106	26	0.866666667	SYNJ2	Signaling
DMR6:158169001	6	158169001	1000	1	1.19E-15	-1.3900025	19	1.9	SERAC1;GTF2H5	Transcription
DMR6:158443001	6	158443001	2000	1	4.08E-10	-0.8153594	28	1.4	TULP4;RN7SL173P	
DMR6:158552001	6	158552001	2000	1	7.59E-07	0.7810468	21	1.05	TMEM181	
DMR6:158690001	6	158690001	3000	1	3.06E-08	-0.6476832	74	2.466666667	SYTL3	

DMR6:159010001	6	159010001	3000	1	1.04E-06	0.9537377	34	1.133333333	RSPH3;TAGAP-AS1	Development
DMR6:160000001	6	160000001	1000	1	1.89E-06	-0.7659456	15	1.5	IGF2R;AIRN	Transport
DMR6:160100001	6	160100001	1000	1	1.45E-10	-0.8124152	5	0.5	IGF2R;CHP1P2	Transport
DMR6:160841001	6	160841001	5000	1	6.14E-06	0.573042	84	1.68	LOC107986665	
DMR6:160847001	6	160847001	5000	1	2.39E-09	0.5001345	77	1.54	LOC107986665	
DMR6:161279001	6	161279001	2000	1	4.95E-06	-0.6208596	29	1.45	AGPAT4	Metabolism
DMR6:161806001	6	161806001	2000	1	1.74E-06	0.5533055	33	1.65	PRKN	Proteolysis
DMR6:162124001	6	162124001	2000	1	1.03E-06	0.6723757	28	1.4	PRKN	Proteolysis
DMR6:162176001	6	162176001	1000	1	4.45E-06	1.2197988	7	0.7	PRKN	Proteolysis
DMR6:163155001	6	163155001	1000	1	1.85E-06	0.5280471	17	1.7	PACRG	
DMR6:163202001	6	163202001	1000	1	4.96E-06	0.5685911	13	1.3	PACRG;PACRG-AS3;LOC105378095	
DMR6:163647001	6	163647001	1000	1	3.78E-06	0.571868	16	1.6		
DMR6:164016001	6	164016001	1000	1	2.61E-06	0.676273	10	1	LOC105378102	
DMR6:166571001	6	166571001	1000	1	2.37E-06	0.6829901	27	2.7	RPS6KA2	Golgi
DMR6:167413001	6	167413001	1000	1	7.54E-06	0.7081031	17	1.7	LOC105378126	
DMR6:167569001	6	167569001	1000	1	1.12E-06	0.6802997	42	4.2	LOC105378130;LOC105378131	
DMR6:167590001	6	167590001	2000	1	8.00E-07	-0.3280842	12	0.6	LOC105378131	
DMR6:168026001	6	168026001	1000	1	4.20E-06	0.7699123	15	1.5	KIF25	Cytoskeleton
DMR6:168311001	6	168311001	1000	1	5.67E-06	-0.5049204	22	2.2	DACT2;LOC105378138	
DMR6:168823001	6	168823001	1000	1	5.40E-06	-0.6889514	33	3.3		
DMR6:169371001	6	169371001	2000	1	2.79E-08	-0.6159016	23	1.15	LINC02519	
DMR6:169621001	6	169621001	1000	1	3.29E-06	0.6188912	23	2.3	WDR27	
DMR6:169839001	6	169839001	4000	1	6.37E-07	-0.58285	102	2.55		
DMR6:170085001	6	170085001	3000	1	6.43E-06	-0.6691893	128	4.266666667		
DMR6:170495001	6	170495001	2000	2	4.05E-07	-0.6590228	54	2.7	LOC105378157	
DMR7:211001	7	211001	1000	1	3.63E-07	-0.6348414	41	4.1	FAM20C	
DMR7:296001	7	296001	3000	1	1.14E-07	-0.6110313	113	3.766666667	FOXL3;FOXL3-OT1	Transcription
DMR7:402001	7	402001	1000	1	2.30E-06	0.8070188	26	2.6		
DMR7:598001	7	598001	1000	1	2.26E-10	-0.6533024	23	2.3	PRKAR1B;PRKAR1B-AS1	Signaling
DMR7:615001	7	615001	1000	1	1.83E-07	-0.4196591	28	2.8	PRKAR1B;PRKAR1B-AS1;LOC105375119	Signaling
DMR7:785001	7	785001	1000	1	1.28E-06	0.8551599	37	3.7	DNAAF5	Transcription
DMR7:889001	7	889001	5000	1	6.15E-06	-0.5031117	262	5.24	GET4;ADAP1	
DMR7:989001	7	989001	2000	1	6.18E-07	-0.6931748	110	5.5	C7orf50;CYP2W1	Metabolism
DMR7:1929001	7	1929001	2000	2	3.99E-06	-0.5902876	100	5	MAD1L1	
DMR7:2902001	7	2902001	1000	1	8.81E-10	-0.728029	17	1.7	CARD11	
DMR7:4248001	7	4248001	1000	1	6.75E-06	0.5592938	15	1.5	SDK1	
DMR7:4419001	7	4419001	1000	1	3.70E-07	-0.7277455	8	0.8		
DMR7:4583001	7	4583001	1000	1	4.99E-07	0.830886	19	1.9		
DMR7:5564001	7	5564001	2000	1	4.70E-08	-0.6612371	65	3.25	LOC100288712	
DMR7:5611001	7	5611001	1000	1	2.67E-06	-0.577662	40	4	FSCN1;LOC107986762;RNF216	Cytoskeleton;Proteolysis
DMR7:6442001	7	6442001	3000	1	9.75E-07	-0.5820808	62	2.066666667	DAGLB	Metabolism
DMR7:6458001	7	6458001	1000	1	5.75E-06	-0.5372088	28	2.8	KDELR2	Transport
DMR7:6856001	7	6856001	1000	1	5.50E-06	-0.5916913	24	2.4	UNC93B2;OR7E136P	
DMR7:7577001	7	7577001	1000	1	8.17E-08	0.7654195	9	0.9	MIOS	
DMR7:12555001	7	12555001	1000	1	7.20E-06	0.5517322	20	2		
DMR7:15745001	7	15745001	1000	1	9.93E-07	0.9916838	10	1	LOC105375167	
DMR7:22909001	7	22909001	1000	1	6.62E-06	0.568065	8	0.8	FAM126A	
DMR7:24717001	7	24717001	1000	1	5.04E-07	0.7395718	21	2.1	GSDME	
DMR7:25554001	7	25554001	4000	1	9.98E-06	0.8410617	37	0.925		
DMR7:27367001	7	27367001	2000	1	1.57E-08	-0.575138	16	0.8		
DMR7:30798001	7	30798001	1000	1	3.66E-06	0.6617912	21	2.1	INMT-MINDY4;MINDY4	
DMR7:34149001	7	34149001	2000	1	2.08E-07	0.5932367	13	0.65	BMPER	Extracellular Matrix
DMR7:35807001	7	35807001	1000	1	1.14E-07	-0.7259951	28	2.8	SEPTIN7-DT;SEPTIN7	Cytoskeleton
DMR7:36515001	7	36515001	4000	1	8.25E-07	-0.5308659	40	1	AOAH	Metabolism
DMR7:37319001	7	37319001	1000	1	1.72E-06	0.4241159	10	1	ELMO1	Cytoskeleton
DMR7:37353001	7	37353001	1000	1	6.71E-06	0.5317499	32	3.2	ELMO1;RNU6-565P	Cytoskeleton
DMR7:39617001	7	39617001	1000	1	5.48E-09	-0.8470184	10	1	YAE1;LOC646999;LOC107986785;RALA	Signaling
DMR7:41967001	7	41967001	1000	1	9.56E-10	0.8514512	15	1.5	GLI3	Transcription
DMR7:43140001	7	43140001	2000	1	1.75E-07	0.7247125	37	1.85	HECW1;HECW1-IT1;MIR3943	Proteolysis
DMR7:43197001	7	43197001	2000	1	3.00E-08	-0.4685256	30	1.5	HECW1;RNU7-35P;RNU6-575P	Proteolysis
DMR7:44246001	7	44246001	1000	1	4.31E-07	0.919348	13	1.3	CAMK2B	Signaling
DMR7:44809001	7	44809001	2000	1	2.06E-07	-0.9692333	53	2.65	PPIA	Transcription
DMR7:44835001	7	44835001	3000	2	2.49E-07	-0.7050096	44	1.466666667	LOC105375260;H2AZ2	Epigenetic
DMR7:45771001	7	45771001	2000	1	8.95E-15	-0.9989322	31	1.55	SEPTIN7P2;GTF2IP13	

DMR7:51392001	7	51392001	1000	1	1.50E-09	0.9528299	12	1.2	CICP17;LOC101928675;LOC100133177;LOC107986795;LOC107986794;LOC102723533	
DMR7:55403001	7	55403001	1000	1	3.02E-06	-0.5333817	41	4.1	LANCL2	
DMR7:55741001	7	55741001	3000	1	8.44E-06	-0.5787649	147	4.9	SUMO2P3;CICP11;LOC107986799;LOC100419984;LOC101928755;LOC112267994;LOC101060341;LOC102723656	
DMR7:55772001	7	55772001	1000	1	5.10E-09	-0.5892904	34	3.4	PSPHP1	
DMR7:55866001	7	55866001	1000	1	2.07E-06	-0.5189087	37	3.7	SEPTIN14	Cytoskeleton
DMR7:56208001	7	56208001	1000	1	1.14E-06	-0.8282877	21	2.1		
DMR7:56367001	7	56367001	1000	1	3.45E-07	-0.7083391	50	5	SEPTIN14P24;CICP8;LOC731631;LOC100419985;LOC100533648;LOC100419986;LOC100533649;LOC107986800	
DMR7:56735001	7	56735001	1000	1	2.14E-06	-0.6822234	25	2.5	LOC728416	
DMR7:58057001	7	58057001	5000	1	2.47E-07	-0.4502049	199	3.98		
DMR7:58495001	7	58495001	1000	1	8.85E-07	0.7942899	17	1.7		
DMR7:60935001	7	60935001	4000	1	2.66E-06	-0.3589416	121	3.025		
DMR7:63245001	7	63245001	1000	1	1.89E-07	-0.7260039	26	2.6	SEPTIN7P4	
DMR7:64104001	7	64104001	6000	2	1.67E-07	0.8493662	76	1.266666667	GUSBP6;MTND4P2;MTND4LP2;MTND3P2;MTCO3P8;MTATP6P18;MTCO2P8;MTCO1P8;MTND2P4;MTND1P2	
DMR7:65251001	7	65251001	1000	1	1.59E-08	-0.5225928	43	4.3		
DMR7:65489001	7	65489001	2000	1	4.98E-07	-0.4236154	48	2.4	LOC101929322	
DMR7:65862001	7	65862001	1000	1	6.26E-06	-0.6281786	34	3.4	RNU6-973P;VKORC1L1	Metabolism
DMR7:66058001	7	66058001	3000	1	9.93E-06	-0.4438446	73	2.433333333	LOC644667	
DMR7:66498001	7	66498001	1000	1	2.79E-06	-0.5618092	14	1.4	GS1-124K5.4;LOC346329	
DMR7:68444001	7	68444001	1000	1	5.88E-06	0.7894388	3	0.3		
DMR7:72047001	7	72047001	2000	1	6.18E-06	0.8310149	24	1.2	CALN1	
DMR7:72218001	7	72218001	1000	1	2.16E-09	-1.0356469	20	2	CALN1	
DMR7:72290001	7	72290001	1000	1	8.38E-06	-0.8738361	1	0.1	CALN1	
DMR7:72315001	7	72315001	2000	1	8.89E-06	-0.7414009	29	1.45	CALN1	
DMR7:72458001	7	72458001	1000	1	4.75E-16	-2.8197892	2	0.2	CALN1	
DMR7:73227001	7	73227001	1000	1	9.78E-06	-0.5018414	25	2.5	NCF1B	
DMR7:73883001	7	73883001	2000	1	9.68E-07	-0.5966835	40	2		
DMR7:74129001	7	74129001	1000	1	4.28E-06	-0.6658583	17	1.7	LIMK1	
DMR7:74201001	7	74201001	1000	1	9.94E-06	-0.4435627	33	3.3	EIF4H;MIR590;LAT2	Translation
DMR7:74369001	7	74369001	3000	1	7.46E-06	-0.7147344	54	1.8	CLIP2	Transcription
DMR7:74574001	7	74574001	1000	1	1.58E-08	-0.6914872	23	2.3	GTF2IRD1	Transcription
DMR7:74885001	7	74885001	1000	1	8.93E-06	-0.6414692	18	1.8	STAG3L2;PMS2P5	Epigenetic;Transcription
DMR7:74935001	7	74935001	2000	2	3.71E-09	-0.9822569	20	1		
DMR7:75265001	7	75265001	2000	1	4.49E-07	-0.9555726	19	0.95	SPDYE14	
DMR7:76111001	7	76111001	1000	1	1.76E-06	-0.6107165	18	1.8	GTF2IP7;LOC645324	
DMR7:76265001	7	76265001	2000	1	7.98E-10	-1.2045744	46	2.3	SRRM3	
DMR7:76997001	7	76997001	3000	1	1.76E-06	-0.490573	52	1.733333333	DTX2P1;DTX2P1-UPK3BP1-PMS2P11;UPK3BP1	
DMR7:77640001	7	77640001	3000	1	5.58E-06	-0.5767105	43	1.433333333	PTPN12	
DMR7:77660001	7	77660001	1000	1	8.03E-06	-0.4813032	33	3.3	LOC105375363;APTR	
DMR7:81304001	7	81304001	1000	1	2.37E-07	0.7052895	7	0.7		
DMR7:81336001	7	81336001	1000	1	3.96E-06	-0.6877295	19	1.9		
DMR7:81387001	7	81387001	1000	1	3.59E-07	0.6439191	11	1.1		
DMR7:83457001	7	83457001	1000	1	7.57E-06	0.7404989	5	0.5	SEMA3E	Signaling
DMR7:84425001	7	84425001	1000	1	3.57E-06	-1.2516902	9	0.9	SEMA3A	Signaling
DMR7:92852001	7	92852001	1000	1	6.01E-06	0.5453769	8	0.8	CDK6-AS1	
DMR7:93316001	7	93316001	1000	1	7.62E-06	0.6851279	5	0.5	VPS50	
DMR7:97999001	7	97999001	2000	1	2.36E-06	-0.7560096	43	2.15	OCM2;RN7SL478P	Signaling
DMR7:100133001	7	100133001	2000	1	4.15E-09	-0.6983274	46	2.3	TAF6;CNPY4;MBLAC1;RPL7P60;LAMTOR4	Transcription
DMR7:100280001	7	100280001	4000	1	4.18E-06	-0.5723312	75	1.875	CASTOR3	
DMR7:101151001	7	101151001	1000	1	1.11E-06	-0.64429	20	2	AP1S1;MIR4653	Transport
DMR7:101874001	7	101874001	2000	1	8.66E-08	0.8551726	44	2.2	CUX1	Development
DMR7:102589001	7	102589001	1000	1	3.62E-07	0.8586013	9	0.9	RASA4	Signaling
DMR7:104181001	7	104181001	1000	1	2.83E-06	-0.800437	15	1.5	ORC5	Cell Cycle
DMR7:107675001	7	107675001	1000	1	3.29E-06	-0.7133845	14	1.4	SLC26A4	Transport
DMR7:107770001	7	107770001	2000	1	1.75E-07	-0.5913482	17	0.85	CBLL1;SLC26A3	Proteolysis;Transport
DMR7:107951001	7	107951001	1000	1	1.34E-06	0.8741751	17	1.7	LAMB1	Extracellular Matrix
DMR7:107980001	7	107980001	1000	1	7.10E-06	1.0101198	6	0.6	LAMB1	Extracellular Matrix
DMR7:114724001	7	114724001	1000	1	4.33E-06	0.9493746	6	0.6		
DMR7:115396001	7	115396001	1000	1	3.75E-06	-0.5757289	16	1.6		

DMR7:118703001	7	118703001	1000	1	2.28E-12	-0.7666172	15	1.5		
DMR7:119088001	7	119088001	1000	1	2.22E-07	-1.0465124	1	0.1		
DMR7:121740001	7	121740001	1000	1	1.44E-07	-1.1763638	5	0.5	RN7SKP277	
DMR7:126235001	7	126235001	1000	1	3.43E-07	-0.9146031	6	0.6	LOC105375488	
DMR7:127680001	7	127680001	1000	1	1.17E-06	0.5872704	29	2.9	SND1	
DMR7:129339001	7	129339001	2000	1	5.57E-08	-0.573273	44	2.2	AHCYL2;LOC105375500	Metabolism
DMR7:130374001	7	130374001	1000	1	5.45E-06	0.7605232	17	1.7	CPA5;LOC105375504;CPA1	Protease
DMR7:130449001	7	130449001	3000	1	8.64E-06	-0.6957482	26	0.866666667	CEP41;LOC105375505	
DMR7:130651001	7	130651001	1000	1	9.24E-07	-0.5252578	32	3.2	COPG2	Transport
DMR7:131653001	7	131653001	3000	1	1.10E-06	-0.5069784	45	1.5	EEF1B2P6;RPS14P10	
DMR7:133234001	7	133234001	1000	1	3.65E-06	-0.772889	15	1.5		
DMR7:135504001	7	135504001	1000	1	9.81E-07	-0.5937152	35	3.5	CNOT4	Proteolysis
DMR7:136164001	7	136164001	1000	1	3.46E-06	-0.8458492	14	1.4	LOC105375523	
DMR7:136972001	7	136972001	2000	1	1.00E-07	-0.528355	51	2.55	CHRM2;LOC349160	Signaling
DMR7:137760001	7	137760001	2000	1	6.09E-06	0.5172443	21	1.05	DGKI	Signaling
DMR7:140007001	7	140007001	1000	1	7.10E-06	0.5469778	17	1.7	TBXAS1	Metabolism
DMR7:140188001	7	140188001	1000	1	2.99E-06	-0.4940696	23	2.3	KDM7A-DT	
DMR7:140304001	7	140304001	2000	1	6.02E-06	-0.7634924	24	1.2	LOC105375534	
DMR7:140648001	7	140648001	2000	1	9.56E-06	-0.8448472	37	1.85	DENND2A;LOC105375535;RN7SL771P;<br>LOC107986718	
DMR7:144004001	7	144004001	1000	1	2.09E-08	0.9591823	15	1.5	OR6B1	Receptor
DMR7:144417001	7	144417001	1000	1	5.93E-06	0.5606994	26	2.6	NOBOX	Development
DMR7:144780001	7	144780001	1000	1	2.27E-07	-1.3670149	5	0.5	TPK1	Signaling
DMR7:145168001	7	145168001	2000	1	1.94E-06	-0.4446815	17	0.85	LOC105375551	
DMR7:145184001	7	145184001	1000	1	5.91E-07	0.7514227	14	1.4		
DMR7:148647001	7	148647001	2000	1	6.91E-09	1.3121523	30	1.5	LOC643438;LOC100301516	
DMR7:149526001	7	149526001	1000	1	2.32E-06	-0.720906	11	1.1		
DMR7:150359001	7	150359001	2000	1	1.55E-09	-0.5180086	10	0.5	LOC107986858;REPIN1-AS1;REPIN1	Transcription
DMR7:150999001	7	150999001	3000	1	2.47E-08	-0.6334141	67	2.233333333	NOS3	Metabolism
DMR7:151313001	7	151313001	1000	1	1.39E-07	-0.4849039	7	0.7		
DMR7:152783001	7	152783001	3000	1	7.47E-06	-0.6830835	42	1.4	ACTR3B	Cytoskeleton
DMR7:153282001	7	153282001	1000	1	3.51E-11	-0.8879289	19	1.9	LOC102723686	
DMR7:153410001	7	153410001	2000	1	4.32E-09	0.8393808	47	2.35	LINC01287	
DMR7:153471001	7	153471001	1000	1	5.50E-06	0.5744709	19	1.9		
DMR7:153950001	7	153950001	2000	1	4.15E-06	0.4809216	44	2.2	DPP6	Protease
DMR7:154058001	7	154058001	2000	1	7.24E-06	0.6780759	69	3.45	DPP6;LOC101929998;LOC107984014	Protease
DMR7:154615001	7	154615001	1000	1	4.18E-07	0.5717806	14	1.4	DPP6;LOC105375581	Protease
DMR7:154869001	7	154869001	2000	1	5.14E-07	-0.8484714	40	2	DPP6;LOC105375580	Protease
DMR7:155050001	7	155050001	1000	1	3.12E-06	-0.9109177	8	0.8		
DMR7:155207001	7	155207001	2000	1	3.01E-06	-0.6211307	17	0.85	LOC105375586	
DMR7:155925001	7	155925001	2000	1	5.88E-09	0.8229063	36	1.8		
DMR7:156920001	7	156920001	2000	1	8.33E-06	0.5869448	47	2.35	LOC102723795	
DMR7:157299001	7	157299001	2000	1	2.37E-07	-0.5461549	75	3.75		
DMR7:157481001	7	157481001	2000	1	4.70E-07	-0.5077342	29	1.45	LOC101927914	
DMR7:157592001	7	157592001	2000	1	6.77E-07	0.5551809	65	3.25	PTPRN2	Signaling
DMR7:157653001	7	157653001	2000	1	1.42E-10	-0.6388066	83	4.15	PTPRN2	Signaling
DMR7:157656001	7	157656001	2000	1	1.90E-06	-0.4560119	52	2.6	PTPRN2	Signaling
DMR7:157940001	7	157940001	3000	1	1.34E-06	-0.6481599	55	1.833333333	PTPRN2	Signaling
DMR7:158000001	7	158000001	1000	1	3.46E-06	-0.7586983	25	2.5	PTPRN2	Signaling
DMR7:158084001	7	158084001	3000	1	8.98E-06	0.6237183	108	3.6	PTPRN2	Signaling
DMR7:158245001	7	158245001	5000	1	4.54E-07	0.5079878	152	3.04	PTPRN2	Signaling
DMR7:158319001	7	158319001	1000	1	5.58E-06	-0.4565963	7	0.7	PTPRN2	Signaling
DMR7:158351001	7	158351001	2000	2	3.18E-06	-0.5346845	59	2.95	PTPRN2	Signaling
DMR7:158363001	7	158363001	3000	1	8.26E-06	0.5968575	97	3.233333333	PTPRN2	Signaling
DMR7:158727001	7	158727001	3000	1	7.82E-06	-0.5374359	64	2.133333333	RPL21P76;ESYT2	
DMR7:158893001	7	158893001	2000	2	3.86E-09	0.8855373	32	1.6	DYNC2I1	
DMR7:159004001	7	159004001	1000	1	6.37E-07	0.8080608	19	1.9	LINC00689	
DMR8:695001	8	695001	2000	1	1.35E-06	-0.4847167	53	2.65	ERICH1	
DMR8:923001	8	923001	1000	1	1.07E-06	0.8983565	18	1.8	DLGAP2	Cytoskeleton
DMR8:982001	8	982001	1000	1	2.50E-06	1.0668519	13	1.3	DLGAP2	Cytoskeleton
DMR8:990001	8	990001	5000	1	6.82E-06	-0.6067433	116	2.32	DLGAP2	Cytoskeleton
DMR8:1382001	8	1382001	2000	1	8.71E-06	0.6930113	34	1.7	DLGAP2;LOC105379585	Cytoskeleton
DMR8:2260001	8	2260001	1000	1	1.77E-06	0.8221615	20	2	LOC105377783	
DMR8:3558001	8	3558001	2000	1	1.49E-09	0.6784972	40	2	CSMD1	
DMR8:4375001	8	4375001	1000	1	8.81E-06	-0.6859318	9	0.9	CSMD1	
DMR8:4742001	8	4742001	3000	1	4.77E-08	-0.7122566	52	1.733333333	CSMD1	
DMR8:4951001	8	4951001	1000	1	1.31E-06	-0.6056301	4	0.4	CSMD1	
DMR8:5122001	8	5122001	1000	1	1.38E-07	0.6509619	64	6.4	LOC107986907	

DMR8:8705001	8	8705001	1000	1	4.20E-08	-0.7486574	21	2.1	CLDN23	Cell Junction
DMR8:9660001	8	9660001	1000	1	5.67E-06	0.9067821	10	1	TNKS	Signaling
DMR8:10355001	8	10355001	1000	1	3.71E-06	0.9042646	13	1.3	MSRA	Metabolism
DMR8:10426001	8	10426001	3000	1	4.15E-06	0.7481517	66	2.2	MSRA	Metabolism
DMR8:10758001	8	10758001	2000	1	5.74E-07	0.7056131	45	2.25	LOC102723313;PINX1	Metabolism
DMR8:11037001	8	11037001	1000	1	9.03E-06	-0.5255579	26	2.6	XKR6;MIR598	
DMR8:11175001	8	11175001	1000	1	1.59E-06	0.577703	18	1.8	XKR6;RPL17P29	
DMR8:11360001	8	11360001	2000	1	7.52E-06	0.7293608	37	1.85	TDH;FAM167A-AS1	Metabolism
DMR8:12560001	8	12560001	1000	1	5.21E-07	-0.7919264	43	4.3	LOC100506990;LOC729732;RPS3AP34	
DMR8:15333001	8	15333001	1000	1	1.56E-06	0.8431513	7	0.7		
DMR8:17239001	8	17239001	1000	1	3.28E-06	0.6034135	18	1.8	ZDHHC2;CNOT7;VPS37A	Translation
DMR8:17883001	8	17883001	1000	1	2.24E-10	-0.9317481	2	0.2	FGL1	Signaling
DMR8:18202001	8	18202001	1000	1	1.29E-08	0.8168289	24	2.4	NAT1;MTND4LP26	Metabolism
DMR8:18528001	8	18528001	1000	1	9.04E-06	0.6897581	21	2.1	PSD3	Transcription
DMR8:19222001	8	19222001	1000	1	1.13E-08	0.6812259	10	1	LOC100128993	
DMR8:20084001	8	20084001	1000	1	2.53E-07	0.7498466	13	1.3	LOC105379311	
DMR8:23330001	8	23330001	4000	1	1.13E-06	-0.4545566	73	1.825	LOXL2;LOC100507156	Metabolism
DMR8:26244001	8	26244001	2000	2	5.31E-18	-2.974132	14	0.7	LOC100129404	
DMR8:29061001	8	29061001	3000	1	3.05E-06	0.7521305	71	2.366666667	HMBOX1;KIF13B	Cytoskeleton
DMR8:30003001	8	30003001	2000	1	7.77E-06	-0.7970337	35	1.75		
DMR8:33319001	8	33319001	1000	1	2.05E-41	-5.1909151	0	0	FUT10	Golgi
DMR8:37493001	8	37493001	3000	1	7.91E-06	-0.4029523	68	2.266666667	LINC01605	
DMR8:38075001	8	38075001	1000	1	8.09E-10	-1.1563666	17	1.7		
DMR8:38151001	8	38151001	1000	1	9.14E-06	-0.5200993	21	2.1	STAR;LOC105379382	
DMR8:38322001	8	38322001	2000	1	8.23E-06	1.0020484	19	0.95	NSD3	
DMR8:38513001	8	38513001	1000	1	4.19E-08	-0.7068178	38	3.8	C8orf86	
DMR8:39010001	8	39010001	1000	1	5.06E-06	0.5883385	12	1.2	ADAM9;SNORD38D	Protease
DMR8:39131001	8	39131001	1000	1	9.91E-06	-0.5642219	21	2.1	ADAM32	Protease
DMR8:41608001	8	41608001	2000	1	1.72E-06	0.9581766	41	2.05	GPAT4	Metabolism
DMR8:43237001	8	43237001	5000	5	1.52E-25	-1.6077107	15	0.3	LOC101059977;AFG3L2P1	
DMR8:46469001	8	46469001	1000	1	7.68E-06	-0.8883298	24	2.4		
DMR8:47983001	8	47983001	3000	1	1.94E-06	-0.556199	60	2	MCM4;RNU6-519P;TCONS_00068220	Transcription
DMR8:50291001	8	50291001	1000	1	6.08E-06	0.8051969	8	0.8	SNTG1	
DMR8:50641001	8	50641001	1000	1	2.02E-06	0.6282847	8	0.8	SNTG1	
DMR8:53010001	8	53010001	1000	1	7.98E-06	-0.7561589	4	0.4		
DMR8:53888001	8	53888001	1000	1	9.94E-06	0.7739665	9	0.9	RGS20;RPS27AP13	Signaling
DMR8:54091001	8	54091001	2000	1	2.39E-06	-0.7205341	23	1.15	LYPLA1	Metabolism
DMR8:55299001	8	55299001	2000	1	7.24E-07	0.7390535	20	1	XKR4	
DMR8:56196001	8	56196001	2000	1	4.07E-06	0.6005002	34	1.7	PLAG1	Transcription
DMR8:57448001	8	57448001	1000	1	8.74E-07	-0.6033828	7	0.7		
DMR8:58011001	8	58011001	1000	1	1.40E-06	0.7700394	16	1.6	FAM110B	
DMR8:58518001	8	58518001	2000	1	1.51E-06	-0.7772659	17	0.85	LOC105375857	
DMR8:58697001	8	58697001	1000	1	4.79E-08	0.8861134	14	1.4		
DMR8:60465001	8	60465001	3000	1	6.67E-07	1.0923203	35	1.166666667		
DMR8:60565001	8	60565001	1000	1	2.91E-07	-0.6516426	13	1.3	RAB2A	
DMR8:62763001	8	62763001	1000	1	4.21E-06	-0.7337158	16	1.6	NKAIN3	
DMR8:62919001	8	62919001	1000	1	1.77E-07	-0.8514404	14	1.4	NKAIN3	
DMR8:63164001	8	63164001	1000	1	4.59E-06	-0.7135662	16	1.6	YTHDF3-DT;YTHDF3	
DMR8:63506001	8	63506001	1000	1	9.80E-06	0.663046	11	1.1		
DMR8:65326001	8	65326001	1000	1	3.73E-06	0.7234699	14	1.4		
DMR8:66694001	8	66694001	1000	1	6.79E-06	-0.7637361	23	2.3	C8orf44-SGK3	
DMR8:66700001	8	66700001	1000	1	7.54E-06	-0.609645	24	2.4	C8orf44-SGK3	
DMR8:69491001	8	69491001	2000	1	4.12E-07	-0.4891243	53	2.65	SULF1	Metabolism
DMR8:69672001	8	69672001	1000	1	3.00E-06	0.8182021	16	1.6	SLCO5A1	Transport
DMR8:69964001	8	69964001	3000	1	2.61E-07	-0.7577248	70	2.333333333		
DMR8:73350001	8	73350001	1000	1	1.74E-06	-0.4852951	31	3.1	RDH10-AS1	
DMR8:73817001	8	73817001	2000	1	7.32E-06	0.5810721	19	0.95	UBE2W	Proteolysis
DMR8:80281001	8	80281001	2000	1	2.53E-06	-0.5770747	45	2.25		
DMR8:80564001	8	80564001	1000	1	4.64E-06	-0.7442527	13	1.3	RPSAP47;RNU7-174P;OCIAD2P1	
DMR8:82914001	8	82914001	1000	1	2.04E-07	-0.918297	4	0.4	LOC101927141	
DMR8:85086001	8	85086001	1000	1	1.46E-06	-0.6289596	17	1.7	LOC105375933;LOC100422614	
DMR8:85595001	8	85595001	1000	1	2.21E-06	-0.5259029	33	3.3		
DMR8:91118001	8	91118001	1000	1	7.02E-07	-0.7155683	9	0.9	LRRC69	Cytoskeleton
DMR8:92151001	8	92151001	3000	1	1.68E-06	0.6242356	21	0.7	RPS26P10	
DMR8:93644001	8	93644001	1000	1	1.22E-14	-2.1327336	3	0.3	CIBAR1-DT;ZNF317P1	
DMR8:96121001	8	96121001	1000	1	9.09E-06	0.6814854	5	0.5	TUBBP7	
DMR8:100052001	8	100052001	2000	1	1.89E-07	-0.9352934	28	1.4	RGS22	
DMR8:100506001	8	100506001	1000	1	2.45E-09	0.6334663	13	1.3	LOC105375670;ANKRD46	

DMR8:100823001	8	100823001	1000	1	9.03E-06	0.8284529	5	0.5	RNU4-83P	
DMR8:102993001	8	102993001	2000	1	3.56E-06	-0.66169	30	1.5		
DMR8:109678001	8	109678001	1000	1	8.29E-06	-0.8377903	14	1.4	SYBU	Transcription
DMR8:111156001	8	111156001	1000	1	1.99E-09	-0.9329699	26	2.6	LINC01609	
DMR8:115000001	8	115000001	1000	1	2.80E-07	-0.7116658	19	1.9		
DMR8:116837001	8	116837001	2000	1	2.04E-06	-0.6455128	20	1	RAD21	
DMR8:117097001	8	117097001	1000	1	6.69E-11	-1.1199198	13	1.3	SLC30A8;LOC105375716;LOC107986969;LOC105375719	Transport
DMR8:118299001	8	118299001	1000	1	5.94E-07	0.7634082	6	0.6	SAMD12;LOC105375724	
DMR8:119378001	8	119378001	1000	1	6.57E-06	0.8893951	8	0.8		
DMR8:119716001	8	119716001	1000	1	6.03E-06	-0.6037312	24	2.4	RN7SKP153	
DMR8:124301001	8	124301001	2000	1	6.03E-37	-2.2249148	48	2.4	LOC112268031;TMEM65	
DMR8:126462001	8	126462001	1000	1	8.63E-06	0.8808327	10	1		
DMR8:129496001	8	129496001	2000	1	4.75E-09	-1.3085742	86	4.3	CCDC26	
DMR8:133737001	8	133737001	1000	1	3.58E-06	-0.3201729	21	2.1		
DMR8:134463001	8	134463001	4000	1	9.46E-06	0.6085371	219	5.475		
DMR8:134895001	8	134895001	1000	1	6.05E-08	0.8840011	10	1		
DMR8:136037001	8	136037001	2000	1	1.94E-12	-1.0808909	23	1.15		
DMR8:139633001	8	139633001	1000	1	2.38E-06	0.8267624	29	2.9	KCNK9	Transport
DMR8:139804001	8	139804001	1000	1	8.29E-09	-0.7293892	24	2.4	TRAPPC9	
DMR8:140112001	8	140112001	1000	1	2.12E-06	-0.3804788	22	2.2	TRAPPC9	
DMR8:140394001	8	140394001	1000	1	7.95E-07	0.9178136	15	1.5	TRAPPC9	
DMR8:140551001	8	140551001	1000	1	2.00E-06	-0.5113525	21	2.1	AGO2	Translation
DMR8:141630001	8	141630001	2000	1	2.91E-08	0.6036236	30	1.5		
DMR8:142082001	8	142082001	2000	1	1.34E-07	-0.4784593	29	1.45		
DMR8:142272001	8	142272001	1000	1	4.65E-07	-0.5134076	29	2.9	TSNARE1	Transcription
DMR8:142501001	8	142501001	2000	2	8.18E-07	-0.4190644	33	1.65	ADGRB1	Signaling
DMR8:142563001	8	142563001	2000	1	3.93E-08	0.5750741	87	4.35	MROH4P	
DMR8:142990001	8	142990001	1000	1	8.29E-06	-0.4990427	15	1.5	LY6E-DT;CDC42P3	
DMR8:143127001	8	143127001	1000	1	4.19E-06	-0.5446098	36	3.6		
DMR8:143165001	8	143165001	4000	2	1.83E-08	-0.5157004	99	2.475	LY6H	
DMR8:143372001	8	143372001	2000	1	2.70E-09	-0.8303568	88	4.4	RHPN1-AS1;RHPN1	Cytoskeleton
DMR8:144513001	8	144513001	2000	1	5.71E-06	-0.4951514	92	4.6	LOC101928953;GPT;MFSD3;RECQL4;LRRC14;LRRC24	Metabolism;Transport;Epigenetic;Receptor
DMR8:144619001	8	144619001	2000	1	1.26E-08	0.5451432	98	4.9	ARHGAP39	
DMR9:773001	9	773001	2000	1	2.26E-06	-0.8691716	29	1.45		
DMR9:869001	9	869001	1000	1	1.54E-07	0.7941371	13	1.3	DMRT1	Transcription
DMR9:3780001	9	3780001	1000	1	1.60E-08	-0.6850428	44	4.4		
DMR9:4777001	9	4777001	1000	1	3.88E-08	-0.7953428	14	1.4	ECM1P1;RPS5P6	
DMR9:5005001	9	5005001	1000	1	7.38E-07	-0.6825791	4	0.4	JAK2;INSL6	Hormone
DMR9:6488001	9	6488001	1000	1	8.94E-07	-0.5624505	16	1.6	UHRF2	Proteolysis
DMR9:6594001	9	6594001	1000	1	6.92E-07	-0.6619995	11	1.1	GLDC	Metabolism
DMR9:6745001	9	6745001	4000	1	1.09E-06	-0.5262093	90	2.25	KDM4C;PRELID3BP11;SNRPEP2	Epigenetic
DMR9:6778001	9	6778001	1000	1	9.94E-07	-0.8446054	12	1.2	KDM4C	Epigenetic
DMR9:8328001	9	8328001	2000	1	9.23E-06	0.6734078	27	1.35	PTPRD	Signaling
DMR9:11611001	9	11611001	1000	1	4.91E-07	0.8841053	19	1.9	LOC105375975	
DMR9:15609001	9	15609001	2000	1	7.52E-06	-0.635012	25	1.25	CCDC171	
DMR9:17408001	9	17408001	1000	1	5.08E-06	-0.6527653	15	1.5	CNTLN	
DMR9:18076001	9	18076001	2000	1	8.62E-06	0.5898919	16	0.8	ADAMTSL1	Protease
DMR9:20090001	9	20090001	1000	1	8.02E-07	0.7447527	10	1	SLC24A2	Transport
DMR9:23328001	9	23328001	2000	1	1.23E-07	-0.7194362	26	1.3		
DMR9:25309001	9	25309001	1000	1	1.22E-11	-0.9846901	15	1.5		
DMR9:30100001	9	30100001	1000	1	1.89E-06	-0.8265105	14	1.4		
DMR9:31028001	9	31028001	2000	1	4.24E-09	-0.7572707	35	1.75	LOC107987029	
DMR9:31620001	9	31620001	1000	1	1.76E-06	-1.0381345	15	1.5	LOC105376010	
DMR9:32360001	9	32360001	1000	1	3.66E-22	-3.5337093	2	0.2	LOC107987059	
DMR9:33194001	9	33194001	1000	1	1.43E-06	-0.6076862	32	3.2		
DMR9:34129001	9	34129001	2000	1	6.20E-06	-0.772951	20	1	DCAF12;IMPDH1P1	
DMR9:34155001	9	34155001	2000	1	1.25E-07	-0.9485144	38	1.9		
DMR9:35913001	9	35913001	2000	2	9.26E-08	-0.6606534	22	1.1	HRCT1;SPAAR	
DMR9:36218001	9	36218001	1000	1	6.31E-06	0.6630589	13	1.3	CLTA;GNE	Transport;Transcription
DMR9:36764001	9	36764001	1000	1	3.43E-07	-0.5393691	15	1.5		
DMR9:38076001	9	38076001	2000	1	2.75E-06	0.6259761	35	1.75	SHB;LOC105376039	
DMR9:38099001	9	38099001	1000	1	8.41E-08	0.9144393	5	0.5		
DMR9:38229001	9	38229001	1000	1	4.79E-07	0.6823907	18	1.8	LOC107987064	
DMR9:38425001	9	38425001	1000	1	1.32E-08	1.4062746	12	1.2	IGFBPL1;LOC105376041	
DMR9:39052001	9	39052001	1000	1	1.05E-07	-0.6524884	7	0.7		
DMR9:39633001	9	39633001	1000	1	1.36E-06	-0.6925634	29	2.9	FKBP4P2	
DMR9:39719001	9	39719001	1000	1	2.23E-08	-0.907635	24	2.4		

DMR9:39996001	9	39996001	1000	1	9.99E-06	-0.6664592	23	2.3	LOC105376050	
DMR9:40197001	9	40197001	2000	2	6.09E-09	-0.5802236	44	2.2	MEP1AP1;LOC102724431	
DMR9:40270001	9	40270001	2000	1	6.06E-07	-0.5763922	13	0.65	ANKRD20A2P	
DMR9:40785001	9	40785001	1000	1	2.45E-06	-0.9435811	3	0.3		
DMR9:40936001	9	40936001	2000	1	9.03E-06	0.687436	10	0.5	MIR1299	
DMR9:41231001	9	41231001	4000	1	3.48E-07	-0.7034783	38	0.95	MIR4477A;RNA5SP530	
DMR9:41496001	9	41496001	1000	1	2.18E-07	-0.7023405	10	1	LOC105376065	
DMR9:43318001	9	43318001	4000	1	6.66E-06	-0.4558083	147	3.675		
DMR9:60555001	9	60555001	89000	13	9.78E-08	-0.8356311	1150	1.292134831		
DMR9:60648001	9	60648001	2000	1	4.84E-06	-0.6629605	23	1.15		
DMR9:60663001	9	60663001	25000	4	5.39E-08	-0.9299371	306	1.224		
DMR9:60897001	9	60897001	1000	1	2.49E-07	-0.6637436	34	3.4	LOC105379434	
DMR9:62742001	9	62742001	2000	1	4.16E-06	-0.7688422	40	2		
DMR9:63817001	9	63817001	3000	1	7.51E-06	-0.4228806	73	2.433333333	LINC00537;RNA5SP284;DUX4L50;MIR4477B	
DMR9:64343001	9	64343001	2000	1	7.49E-09	-0.650034	46	2.3		
DMR9:65458001	9	65458001	1000	1	3.99E-07	-0.4887999	38	3.8	LOC105379446	
DMR9:65505001	9	65505001	2000	1	2.66E-06	-1.0547417	16	0.8		
DMR9:66176001	9	66176001	4000	1	2.15E-08	-0.5263182	70	1.75	MEP1AP3	
DMR9:67833001	9	67833001	3000	1	2.40E-06	-0.4550013	40	1.333333333	LOC101928608	
DMR9:69829001	9	69829001	1000	1	5.90E-06	0.9662452	11	1.1	C9orf135-DT;C9orf135	
DMR9:69916001	9	69916001	1000	1	2.39E-06	0.613819	6	0.6	C9orf135	
DMR9:70982001	9	70982001	1000	1	1.26E-06	0.5595262	7	0.7	TRPM3	Transport
DMR9:71496001	9	71496001	1000	1	2.91E-06	0.8207644	9	0.9		
DMR9:71618001	9	71618001	1000	1	6.88E-06	-0.6617496	11	1.1		
DMR9:71960001	9	71960001	2000	1	2.03E-08	-0.7721933	28	1.4	C9orf85	
DMR9:72492001	9	72492001	1000	1	5.14E-10	-0.6469954	23	2.3	LOC105376080	
DMR9:73054001	9	73054001	1000	1	1.18E-06	-0.8672987	19	1.9	CYP1D1P	
DMR9:76427001	9	76427001	2000	1	8.50E-06	-0.6372312	38	1.9	H3P32	
DMR9:78611001	9	78611001	1000	1	4.39E-06	0.7448797	14	1.4		
DMR9:78741001	9	78741001	4000	1	3.68E-06	0.6215535	64	1.6	MTCO1P50;MTND2P8	
DMR9:81543001	9	81543001	1000	1	9.50E-09	-0.7058031	25	2.5		
DMR9:81589001	9	81589001	2000	1	6.23E-06	0.9629849	39	1.95	TLE1;LOC105376106	Transcription
DMR9:81748001	9	81748001	2000	1	1.14E-09	-0.9631291	38	1.9	LOC101927502	
DMR9:83697001	9	83697001	1000	1	1.94E-07	-0.7551706	21	2.1	UBQLN1;UBQLN1-AS1	
DMR9:83926001	9	83926001	1000	1	4.34E-06	-0.6030935	10	1	KIF27;LOC105376335	Cytoskeleton
DMR9:85270001	9	85270001	1000	1	2.30E-06	-0.473968	20	2	LOC105376119	
DMR9:85785001	9	85785001	1000	1	6.28E-07	-0.8241247	16	1.6	LOC100419824;LOC102724057	
DMR9:85928001	9	85928001	1000	1	7.96E-06	-0.6894132	12	1.2	LOC100130049	
DMR9:86052001	9	86052001	2000	1	4.21E-06	-0.4572789	30	1.5	GOLM1	
DMR9:87632001	9	87632001	2000	1	8.91E-08	0.8003005	5	0.25	DAPK1	Signaling
DMR9:87937001	9	87937001	4000	1	2.43E-06	-0.6136249	111	2.775	LOC497256;LOC645937	
DMR9:89170001	9	89170001	1000	1	8.72E-06	0.9556024	12	1.2	SHC3	Cytoskeleton
DMR9:89283001	9	89283001	1000	1	4.39E-06	0.5692235	23	2.3	LOC105376136	
DMR9:89323001	9	89323001	1000	1	6.01E-06	0.591008	8	0.8	CKS2;SECISBP2	Cytoskeleton
DMR9:89370001	9	89370001	3000	2	3.35E-06	-0.5320137	40	1.333333333	SECISBP2;SEMA4D	Signaling
DMR9:90216001	9	90216001	1000	1	6.38E-07	0.6227665	20	2	OR7E31P	
DMR9:93439001	9	93439001	2000	1	3.62E-09	-0.9135197	28	1.4	LOC107987097;FAM120AOS	
DMR9:94313001	9	94313001	1000	1	4.41E-07	-1.1092861	7	0.7	ZNF169;LOC105376154;NUTM2F	Transcription
DMR9:94622001	9	94622001	1000	1	4.84E-12	0.919138	20	2	FBP1	Metabolism
DMR9:94634001	9	94634001	1000	1	3.93E-06	-0.5613091	22	2.2	FBP1	Metabolism
DMR9:95557001	9	95557001	1000	1	7.44E-06	-0.4660183	19	1.9	LOC105376157	
DMR9:96026001	9	96026001	2000	1	3.97E-08	-0.5632014	106	5.3	ERCC6L2;LINC00092;LOC105376159	
DMR9:96493001	9	96493001	2000	1	8.65E-06	-0.6029017	45	2.25	HABP4;CDC14B	Metabolism;Signaling
DMR9:96517001	9	96517001	1000	1	2.78E-07	-0.6446914	27	2.7	CDC14B	Signaling
DMR9:96910001	9	96910001	3000	1	8.11E-06	-0.5596863	107	3.566666667	PTMAP11;LOC107987024;MFSD14C	
DMR9:97014001	9	97014001	2000	1	9.69E-06	-0.478619	50	2.5	MFSD14C	
DMR9:98125001	9	98125001	1000	1	3.26E-06	-0.9505384	13	1.3	TRIM14;CORO2A	Proteolysis;Cytoskeleton
DMR9:99351001	9	99351001	1000	1	3.49E-06	0.833897	12	1.2	NAMA	
DMR9:99369001	9	99369001	1000	1	3.34E-06	0.6871728	16	1.6	NAMA	
DMR9:103985001	9	103985001	1000	1	9.65E-06	0.79565	7	0.7	LOC105376193	
DMR9:108997001	9	108997001	1000	1	1.55E-07	0.7332468	3	0.3	CTNNAL1;LOC105376216;RNA5-8SP3	
DMR9:109253001	9	109253001	1000	1	4.59E-06	0.6302652	16	1.6	EPB41L4B	
DMR9:109655001	9	109655001	1000	1	6.75E-07	-0.6917486	18	1.8	PALM2AKAP2	
DMR9:112108001	9	112108001	1000	1	6.07E-06	-0.8067152	6	0.6	SUSD1	Extracellular Matrix
DMR9:112642001	9	112642001	1000	1	7.73E-06	0.6929762	10	1	KIAA1958	
DMR9:113285001	9	113285001	1000	1	6.69E-07	-0.618543	19	1.9	CDC26;PRPF4	Translation
DMR9:113670001	9	113670001	3000	1	1.70E-06	0.7652948	52	1.733333333	LOC105376223	

DMR9:113728001	9	113728001	1000	1	4.07E-07	-0.8499456	12	1.2		
DMR9:113744001	9	113744001	1000	1	5.15E-06	0.9434348	15	1.5		
DMR9:114278001	9	114278001	1000	1	5.15E-06	-0.4668658	10	1	COL27A1	Extracellular Matrix
DMR9:114712001	9	114712001	1000	1	2.91E-06	-0.5603285	20	2		
DMR9:118664001	9	118664001	1000	1	1.11E-06	-1.0713262	8	0.8	LOC102724929	
DMR9:119445001	9	119445001	1000	1	3.24E-06	-0.783906	5	0.5	LOC105376250	
DMR9:121543001	9	121543001	2000	1	3.41E-06	-1.0577209	23	1.15	LOC107987016	
DMR9:122156001	9	122156001	1000	1	7.15E-06	0.6554131	7	0.7	NDUFA8;MORN5	Metabolism
DMR9:124728001	9	124728001	1000	1	4.25E-06	-0.6077853	19	1.9	NR6A1	
DMR9:125061001	9	125061001	2000	1	9.42E-06	-0.5376349	30	1.5	SCAI	
DMR9:125373001	9	125373001	1000	1	3.31E-06	-0.5442314	23	2.3	GAPVD1	Transcription
DMR9:125409001	9	125409001	1000	1	8.45E-11	-0.5443659	34	3.4	LOC101929014	
DMR9:125527001	9	125527001	3000	1	1.75E-07	-1.1206767	47	1.566666667	MAPKAP1	Cytoskeleton
DMR9:125615001	9	125615001	1000	1	2.60E-06	-1.02165	7	0.7	MAPKAP1	Cytoskeleton
DMR9:125740001	9	125740001	2000	1	1.73E-06	-0.5671895	45	2.25	LOC51145;PBX3	Development
DMR9:126810001	9	126810001	1000	1	4.84E-06	-0.5657069	24	2.4	ZBTB43	Cytoskeleton
DMR9:127577001	9	127577001	2000	1	5.99E-08	-0.7509087	38	1.9	NIBAN2	
DMR9:127660001	9	127660001	2000	1	4.42E-06	0.7294682	29	1.45	STXBP1	Transport
DMR9:127931001	9	127931001	2000	1	3.94E-07	-0.5700025	65	3.25	PIP5KL1;DPM2;FAM102A	Signaling
DMR9:128263001	9	128263001	3000	1	1.65E-06	-0.5230464	65	2.166666667	DNM1;GOLGA2;SWI5	Transport;Transcription
DMR9:128800001	9	128800001	2000	1	7.24E-06	-0.6255056	34	1.7	TBC1D13	Signaling
DMR9:128859001	9	128859001	1000	1	8.92E-07	-0.5558299	27	2.7	KYAT1	Metabolism
DMR9:129045001	9	129045001	1000	1	1.93E-07	-0.7741895	17	1.7	MIGA2	
DMR9:129297001	9	129297001	2000	1	5.33E-06	0.7295825	14	0.7	LOC107987133	
DMR9:129339001	9	129339001	2000	2	2.59E-06	-0.4600569	23	1.15	LINC01503	
DMR9:129735001	9	129735001	2000	1	8.03E-06	-0.6234204	51	2.55	PTGES	Transport
DMR9:129802001	9	129802001	1000	1	4.22E-06	-0.551289	56	5.6	TOR1B;TOR1A	Transcription
DMR9:130148001	9	130148001	2000	1	5.99E-06	-0.6210645	27	1.35	GPR107;GPRACR	Signaling
DMR9:132314001	9	132314001	3000	1	6.23E-07	0.6098932	45	1.5	SETX	
DMR9:133207001	9	133207001	1000	1	5.47E-06	-0.503461	26	2.6	OBP2B	Transport
DMR9:134097001	9	134097001	2000	1	5.07E-06	-0.5076227	42	2.1		
DMR9:134182001	9	134182001	2000	1	1.84E-08	-0.4365147	11	0.55		
DMR9:134369001	9	134369001	1000	1	1.08E-07	-0.6721292	17	1.7	RXRA;MIR4669	Transcription
DMR9:136451001	9	136451001	1000	1	9.40E-06	0.5615769	35	3.5	SEC16A	
DMR9:137050001	9	137050001	1000	1	7.18E-08	-0.5942289	29	2.9	NPDC1;ENTPD2;LOC105376327	Signaling
DMR9:137059001	9	137059001	1000	1	1.11E-07	-0.6296308	28	2.8	ENTPD2;LOC105376327;SAPCD2	Signaling
DMR9:137134001	9	137134001	1000	1	5.83E-07	-0.8896305	14	1.4	GRIN1	Receptor
DMR9:137394001	9	137394001	2000	1	1.82E-06	-0.5483109	89	4.45	EXD3	
DMR9:137450001	9	137450001	3000	1	1.03E-06	-0.6629973	95	3.166666667	ENTPD8;NSMF;MIR7114;PNPLA7	Signaling;Metabolism
DMR9:137472001	9	137472001	2000	1	5.85E-07	-0.8744819	28	1.4	PNPLA7	Metabolism
DMR9:137496001	9	137496001	1000	1	1.29E-09	-0.6915643	29	2.9	PNPLA7	Metabolism
DMR9:137716001	9	137716001	2000	1	8.33E-07	-0.72684	48	2.4	EHMT1	
DMR9:137721001	9	137721001	1000	1	6.45E-06	-0.47619	30	3	EHMT1	
DMR9:137841001	9	137841001	2000	1	4.47E-06	-0.7324628	140	7	EHMT1;MIR602	
DMR10:186001	10	186001	1000	1	3.55E-07	-0.6839421	26	2.6	ZMYND11	Transcription
DMR10:263001	10	263001	3000	2	1.31E-07	0.87977	32	1.066666667	ZMYND11;LOC107984191;DIP2C	Transcription
DMR10:380001	10	380001	1000	1	3.58E-06	0.5491859	28	2.8	DIP2C	
DMR10:416001	10	416001	1000	1	4.03E-06	0.600695	28	2.8	DIP2C	
DMR10:447001	10	447001	2000	1	6.03E-08	0.9690971	44	2.2	DIP2C	
DMR10:758001	10	758001	1000	1	3.09E-06	-0.6330596	14	1.4		
DMR10:947001	10	947001	3000	1	3.82E-06	0.4454451	81	2.7	LOC101927762;LOC107984285;LOC105376341	
DMR10:1153001	10	1153001	2000	1	3.35E-08	0.7008846	46	2.3	LOC105376344;LINC00200	
DMR10:1209001	10	1209001	3000	1	4.86E-07	0.6912811	62	2.066666667	ADARB2	Metabolism
DMR10:1991001	10	1991001	2000	1	7.38E-06	0.6122187	19	0.95	LOC105376346	
DMR10:2244001	10	2244001	2000	1	2.95E-07	0.6692222	36	1.8		
DMR10:3254001	10	3254001	1000	1	1.76E-06	-0.4534215	8	0.8	LINC02668;LOC105376353	
DMR10:3434001	10	3434001	1000	1	9.01E-07	0.6284912	15	1.5	LOC105376360;LINC02669	
DMR10:4645001	10	4645001	1000	1	2.81E-06	0.8620875	4	0.4	MANCR	
DMR10:7312001	10	7312001	1000	1	7.79E-06	0.7501144	18	1.8	SFMBT2	Epigenetic
DMR10:8051001	10	8051001	1000	1	1.66E-07	-0.9312288	78	7.8	LOC105376394;GATA3;GATA3-AS1;LOC107984204	Transcription
DMR10:8138001	10	8138001	1000	1	8.40E-07	-0.7442788	8	0.8		
DMR10:12463001	10	12463001	2000	1	8.01E-06	0.6785565	37	1.85	CAMK1D;RNU6ATAC39P;LOC107984209	Signaling
DMR10:13435001	10	13435001	1000	1	4.39E-06	-0.9703604	18	1.8	BEND7	
DMR10:13472001	10	13472001	3000	1	8.56E-11	0.6967543	160	5.333333333	BEND7	
DMR10:14776001	10	14776001	1000	1	2.64E-06	0.8920711	18	1.8	FAM107B	

DMR10:16133001	10	16133001	1000	1	3.71E-06	-0.7846005	21	2.1		
DMR10:21305001	10	21305001	1000	1	6.98E-06	-0.6424242	13	1.3		
DMR10:21381001	10	21381001	1000	1	6.60E-06	-0.7697797	10	1	LINC02643	
DMR10:23068001	10	23068001	1000	1	9.06E-06	-0.6811385	15	1.5	LOC107984215	
DMR10:24036001	10	24036001	1000	1	3.25E-06	0.6779534	15	1.5	KIAA1217	
DMR10:24208001	10	24208001	1000	1	1.05E-06	-0.8626083	28	2.8	KIAA1217	
DMR10:24785001	10	24785001	1000	1	4.78E-06	0.5224191	15	1.5	LOC105376456	
DMR10:24809001	10	24809001	1000	1	1.62E-09	-0.7602511	26	2.6		
DMR10:25719001	10	25719001	2000	1	2.37E-06	0.5252171	24	1.2	LINC00836	
DMR10:25751001	10	25751001	1000	1	2.47E-07	0.7327581	7	0.7		
DMR10:26292001	10	26292001	2000	1	1.82E-06	0.9245857	33	1.65	GAD2	Metabolism
DMR10:28336001	10	28336001	1000	1	7.13E-06	-0.7811018	11	1.1	LOC105376467;ZNF101P1	
DMR10:29695001	10	29695001	1000	1	1.96E-10	-0.7271233	22	2.2	SVIL;CKS1BP2	Cytoskeleton
DMR10:30323001	10	30323001	1000	1	9.50E-06	-0.5264341	30	3	MTPAP	Metabolism
DMR10:34324001	10	34324001	1000	1	9.00E-07	0.5461064	15	1.5	PARD3	
DMR10:34676001	10	34676001	1000	1	9.84E-06	0.5962823	15	1.5	PARD3;RPS12P16	
DMR10:36705001	10	36705001	1000	1	8.93E-06	-0.8538031	14	1.4		
DMR10:38483001	10	38483001	11000	1	4.32E-06	-0.3075888	255	2.318181818	LOC107984177	
DMR10:38941001	10	38941001	2000	1	5.38E-06	-0.4656142	141	7.05		
DMR10:41830001	10	41830001	2000	1	4.30E-06	-0.7382821	18	0.9		
DMR10:41839001	10	41839001	78000	1	8.39E-08	-0.496573	1551	1.988461538		
DMR10:42293001	10	42293001	14000	1	1.88E-06	-0.5693035	303	2.164285714		
DMR10:43381001	10	43381001	1000	1	7.47E-06	-0.701608	23	2.3	FXYD4;HNRNPF	Transport;Translation
DMR10:46715001	10	46715001	2000	1	6.61E-06	-0.7220555	30	1.5	LOC101927584	
DMR10:47752001	10	47752001	4000	1	9.77E-06	-0.7467178	101	2.525	ANXA8;LOC102724593;LOC102724488	Signaling
DMR10:49487001	10	49487001	1000	1	1.38E-07	0.9348577	9	0.9	ERCC6	
DMR10:49891001	10	49891001	1000	1	1.62E-07	-0.7613212	13	1.3	PARG	Metabolism
DMR10:53925001	10	53925001	1000	1	6.08E-07	0.5284286	23	2.3	PCDH15	Cytoskeleton
DMR10:63290001	10	63290001	2000	1	2.03E-06	-0.6335077	36	1.8	JMJD1C	Epigenetic
DMR10:65215001	10	65215001	1000	1	1.87E-06	-0.7814989	15	1.5		
DMR10:65474001	10	65474001	1000	1	6.29E-06	0.7633432	6	0.6	LOC105378337	
DMR10:68400001	10	68400001	1000	1	6.76E-06	-0.5110573	32	3.2	RUFY2	
DMR10:68429001	10	68429001	1000	1	1.39E-06	-0.5338108	26	2.6	DNA2;RPL26P29	
DMR10:68528001	10	68528001	2000	1	7.52E-06	-0.4180681	66	3.3	SLC25A16	Transport
DMR10:68616001	10	68616001	2000	1	6.93E-06	-0.5603843	52	2.6	TET1	
DMR10:68660001	10	68660001	2000	1	7.69E-06	-0.5690848	36	1.8	TET1	
DMR10:69307001	10	69307001	4000	1	2.37E-06	-0.55445	75	1.875	HK1;RPS15AP28	Signaling
DMR10:69380001	10	69380001	1000	1	6.92E-06	0.7154254	26	2.6	HK1	Signaling
DMR10:70607001	10	70607001	1000	1	9.80E-06	0.8541315	9	0.9	PRF1	
DMR10:72198001	10	72198001	1000	1	1.98E-08	-0.9208047	9	0.9	ASCC1;RPL15P14	
DMR10:72696001	10	72696001	1000	1	9.29E-10	-1.0000709	11	1.1	MCU	
DMR10:72980001	10	72980001	1000	1	8.42E-06	0.8771403	4	0.4		
DMR10:73204001	10	73204001	2000	1	3.75E-06	-0.6658238	17	0.85	FAM149B1;EIF4A2P2	
DMR10:73978001	10	73978001	1000	1	1.30E-11	-0.8183506	40	4		
DMR10:75394001	10	75394001	1000	1	4.29E-06	0.8680347	15	1.5	ZNF503;ZNF503-AS2	
DMR10:76034001	10	76034001	1000	1	8.35E-06	0.7464966	25	2.5	LRMDA	
DMR10:77669001	10	77669001	1000	1	6.28E-06	-0.6625454	13	1.3		
DMR10:84602001	10	84602001	2000	1	3.59E-06	-0.5031889	45	2.25		
DMR10:86653001	10	86653001	2000	1	8.89E-07	-0.4330556	31	1.55	OPN4;LOC105378409	Signaling
DMR10:87349001	10	87349001	2000	1	6.83E-06	-0.6415527	43	2.15	NUTM2A-AS1;LINC00863;NUTM2D	
DMR10:91072001	10	91072001	1000	1	1.68E-06	0.7820751	12	1.2	LINC00502	
DMR10:93553001	10	93553001	1000	1	1.55E-07	-0.7334508	12	1.2	LOC105378436	
DMR10:95553001	10	95553001	2000	1	3.73E-06	-0.5949176	22	1.1	SORBS1	
DMR10:95994001	10	95994001	2000	1	6.77E-07	0.7977443	25	1.25	ENTPD1-AS1;CC2D2B;RPL21P90	
DMR10:96718001	10	96718001	1000	1	4.90E-08	0.6847338	12	1.2	PIK3AP1	
DMR10:96887001	10	96887001	1000	1	9.91E-07	-0.7123957	19	1.9	LCOR	Transcription
DMR10:97348001	10	97348001	2000	1	2.54E-07	-0.6756823	52	2.6	LOC105378448;RRP12	
DMR10:97900001	10	97900001	2000	1	2.05E-06	0.634945	40	2	CRTAC1	
DMR10:99693001	10	99693001	1000	1	6.29E-06	0.5826878	13	1.3	ENTPD7;COX15;EBAG9P1	Signaling;Transcription
DMR10:99991001	10	99991001	2000	1	8.32E-06	-0.7094582	40	2	DNMBP	
DMR10:100649001	10	100649001	3000	1	1.50E-06	-0.5813533	51	1.7		
DMR10:101522001	10	101522001	1000	1	1.48E-07	-0.7886646	20	2	BTRC	Cytoskeleton
DMR10:101963001	10	101963001	1000	1	9.06E-06	-0.5229438	26	2.6	ARMH3	
DMR10:102141001	10	102141001	2000	1	3.17E-06	-0.574701	30	1.5	PPRC1;NOLC1	Transcription
DMR10:102771001	10	102771001	2000	1	6.50E-06	-0.728391	36	1.8	WBP1L	
DMR10:103274001	10	103274001	1000	1	6.71E-09	-0.8189635	29	2.9	INA	
DMR10:103753001	10	103753001	1000	1	5.62E-06	-0.9387626	6	0.6	SH3PXD2A;SH3PXD2A-AS1	

DMR10:104684001	10	104684001	1000	1	2.36E-09	-1.0948273	7	0.7	SORCS3	Transport
DMR10:105596001	10	105596001	1000	1	6.55E-06	-0.7712039	18	1.8		
DMR10:113159001	10	113159001	1000	1	7.69E-06	-0.5860083	15	1.5	TCF7L2	Transcription
DMR10:114138001	10	114138001	1000	1	8.85E-08	-0.7207626	9	0.9	CCDC186	
DMR10:114634001	10	114634001	1000	1	4.33E-09	-0.8638515	29	2.9	ABLIM1	Cytoskeleton
DMR10:116953001	10	116953001	1000	1	3.86E-06	-0.7817473	12	1.2	SHTN1	
DMR10:116963001	10	116963001	1000	1	3.03E-06	-0.5407799	28	2.8	SHTN1	
DMR10:117357001	10	117357001	1000	1	3.41E-06	-0.6010026	15	1.5	PDZD8	Cytoskeleton
DMR10:119797001	10	119797001	1000	1	7.82E-06	0.634446	12	1.2	INPP5F;LOC105378513	Signaling
DMR10:121374001	10	121374001	1000	1	4.62E-07	0.9903626	17	1.7		
DMR10:121584001	10	121584001	1000	1	1.33E-08	-0.649545	27	2.7	FGFR2	Receptor
DMR10:122090001	10	122090001	1000	1	3.71E-06	-0.7486844	13	1.3	TACC2	
DMR10:125088001	10	125088001	1000	1	1.75E-06	0.7188704	21	2.1	CTBP2	Transcription
DMR10:127042001	10	127042001	1000	1	3.85E-06	0.6588495	15	1.5	DOCK1	Transcription
DMR10:127138001	10	127138001	1000	1	5.94E-06	0.794003	13	1.3	DOCK1;INSYN2A	Transcription
DMR10:127164001	10	127164001	1000	1	1.83E-07	-0.9543287	12	1.2	DOCK1;INSYN2A	Transcription
DMR10:130496001	10	130496001	1000	1	3.03E-06	0.6337852	18	1.8		
DMR10:130747001	10	130747001	2000	1	5.64E-06	0.6086858	46	2.3		
DMR10:130790001	10	130790001	2000	1	1.79E-12	-0.476061	19	0.95		
DMR10:131156001	10	131156001	1000	1	7.77E-07	0.7700678	21	2.1	TCERG1L	Transcription
DMR10:131883001	10	131883001	1000	1	1.83E-06	-0.5491777	14	1.4	LOC105378565;LOC105378566;LOC100134362	
DMR10:132190001	10	132190001	2000	1	3.93E-07	0.8646009	78	3.9	JAKMIP3;LOC105378567;DPYSL4	Metabolism
DMR10:132358001	10	132358001	4000	1	1.07E-07	-0.7475017	126	3.15	LRRC27	
DMR10:132572001	10	132572001	3000	2	5.65E-08	-0.544323	83	2.766666667	INPP5A	Signaling
DMR10:132710001	10	132710001	1000	1	1.89E-06	-0.5136537	27	2.7	INPP5A	Signaling
DMR10:133054001	10	133054001	2000	1	3.25E-07	0.7579065	23	1.15		
DMR10:133337001	10	133337001	3000	2	2.09E-07	-0.5161183	35	1.166666667	CALY;BANF1P2;PRAP1	
DMR10:133349001	10	133349001	1000	1	4.88E-06	-0.4688228	23	2.3	BANF1P2;PRAP1;FUOM	
DMR10:133686001	10	133686001	1000	1	1.30E-07	-0.6225362	5	0.5	DUX4L22;DUX4L21;DUX4L20	
DMR11:768001	11	768001	2000	1	5.79E-06	-0.5446668	70	3.5	TALDO1;GATD1;LOC171391	Metabolism
DMR11:1094001	11	1094001	7000	1	1.00E-08	-0.4357597	214	3.057142857	MUC2	Extracellular Matrix
DMR11:1580001	11	1580001	3000	1	5.25E-06	0.6670407	83	2.766666667	DUSP8;KRTAP5-AS1;KRTAP5-1	Signaling
DMR11:1745001	11	1745001	2000	1	2.44E-06	0.5741444	55	2.75	IFITM10;CTSD	
DMR11:1923001	11	1923001	1000	1	8.66E-06	0.5008125	23	2.3	TNNT3	Cytoskeleton
DMR11:2196001	11	2196001	1000	1	4.47E-06	-0.3676453	18	1.8		
DMR11:2584001	11	2584001	1000	1	7.78E-06	-0.4047351	11	1.1	KCNQ1	Transport
DMR11:3791001	11	3791001	2000	1	1.91E-07	-0.6071611	51	2.55	NUP98;RNU7-50P;PGAP2	Transport;Golgi
DMR11:3916001	11	3916001	2000	1	2.72E-06	-0.8444591	25	1.25	STIM1	
DMR11:4110001	11	4110001	1000	1	1.12E-07	-0.935769	17	1.7	RRM1	Metabolism
DMR11:4545001	11	4545001	1000	1	3.70E-06	0.6904013	19	1.9	OR52M1	Receptor
DMR11:5925001	11	5925001	1000	1	1.21E-06	0.9270709	9	0.9	LOC112268071	
DMR11:7362001	11	7362001	1000	1	2.53E-06	-0.865388	6	0.6	SYT9	Transport
DMR11:8294001	11	8294001	1000	1	8.55E-06	0.7489225	9	0.9		
DMR11:8764001	11	8764001	1000	1	4.88E-06	-0.6443218	14	1.4	DENND2B;LOC105376539;LOC102724784	
DMR11:9039001	11	9039001	2000	1	7.14E-08	0.7657292	50	2.5	NRIP3-DT;SCUBE2	Extracellular Matrix
DMR11:9361001	11	9361001	3000	1	6.98E-07	0.8090377	50	1.666666667		
DMR11:9382001	11	9382001	1000	1	6.97E-06	-0.4987056	37	3.7	IPO7	Transport
DMR11:9391001	11	9391001	2000	1	2.32E-08	-0.9106395	49	2.45	IPO7	Transport
DMR11:9482001	11	9482001	1000	1	4.33E-07	-0.596453	31	3.1	ZNF143	Transcription
DMR11:10353001	11	10353001	1000	1	1.34E-06	-0.6171523	9	0.9	CAND1.11	
DMR11:12384001	11	12384001	1000	1	1.15E-06	0.811251	7	0.7	PARVA	Cytoskeleton
DMR11:14190001	11	14190001	1000	1	6.90E-06	-0.6258749	8	0.8	SPON1	Cytoskeleton
DMR11:14864001	11	14864001	1000	1	9.30E-07	0.7327984	7	0.7	PDE3B	Signaling
DMR11:18461001	11	18461001	2000	1	6.78E-06	-0.5967963	49	2.45	LDHC;LDHAL6A	Metabolism
DMR11:18678001	11	18678001	1000	1	3.79E-06	-0.7443252	22	2.2		
DMR11:20101001	11	20101001	1000	1	5.33E-06	0.7662073	20	2	NAV2	
DMR11:20755001	11	20755001	2000	1	1.29E-06	-0.6231489	41	2.05	NELL1	Signaling
DMR11:25601001	11	25601001	1000	1	7.67E-06	0.7093522	11	1.1		
DMR11:26912001	11	26912001	1000	1	4.23E-07	-0.8798494	5	0.5		
DMR11:30346001	11	30346001	2000	1	5.75E-06	-0.8632468	20	1	ARL14EP;LOC102723403	
DMR11:32174001	11	32174001	1000	1	2.17E-06	-0.5947596	17	1.7	THEM7P	
DMR11:34282001	11	34282001	2000	1	2.39E-09	-0.8910128	30	1.5	ABTB2	Cytoskeleton
DMR11:39370001	11	39370001	1000	1	3.00E-06	-0.6042041	30	3		
DMR11:39736001	11	39736001	1000	1	2.52E-06	0.6808861	18	1.8	LOC105376637	
DMR11:40860001	11	40860001	1000	1	8.24E-06	-0.6354622	10	1	LRRC4C	
DMR11:41442001	11	41442001	1000	1	6.48E-10	-1.0155583	16	1.6	LRRC4C	

DMR11:46595001	11	46595001	2000	1	4.39E-06	-0.5089084	72	3.6	AMBRA1;HARBI1	
DMR11:47933001	11	47933001	1000	1	8.03E-06	-0.7153099	20	2		
DMR11:47950001	11	47950001	1000	1	9.64E-07	-0.7518697	17	1.7		
DMR11:52128001	11	52128001	1000	1	2.27E-06	-0.5817254	11	1.1		
DMR11:53986001	11	53986001	2000	1	2.10E-07	-0.6197022	22	1.1		
DMR11:57757001	11	57757001	1000	1	8.40E-06	-1.0719011	8	0.8	TMX2-CTNND1;BTBD18;CTNND1	Cytoskeleton
DMR11:59530001	11	59530001	3000	1	3.74E-07	-0.5125481	75	2.5	OR4D9;OR4D7P;LOC102723575	Receptor
DMR11:59631001	11	59631001	1000	1	9.50E-06	-0.430179	29	2.9	LOC101927226;PATL1	Translation
DMR11:59793001	11	59793001	1000	1	5.10E-06	0.6873415	17	1.7	STX3	Transcription
DMR11:60065001	11	60065001	1000	1	1.05E-06	0.5788082	6	0.6	MS4A3	Transport
DMR11:61387001	11	61387001	1000	1	1.02E-06	-0.5706798	22	2.2	TMEM138;TMEM216	
DMR11:62362001	11	62362001	1000	1	4.12E-07	-1.0278308	3	0.3	ASRGL1;RCC2P6	Protease
DMR11:62392001	11	62392001	1000	1	7.08E-08	0.7383631	18	1.8	ASRGL1;LOC107984335	Protease
DMR11:62743001	11	62743001	4000	1	6.62E-07	-0.6674245	100	2.5	TTC9C;ZBTB3	Transcription;Transcription
DMR11:64010001	11	64010001	2000	1	1.53E-07	-0.5447573	19	0.95	MACROD1;LOC101927673	
DMR11:65215001	11	65215001	2000	1	1.23E-06	-0.5729738	53	2.65	CAPN1;SLC22A20P	Protease;Transport
DMR11:65483001	11	65483001	1000	1	9.72E-06	-0.6158578	37	3.7	LINC02736	
DMR11:66633001	11	66633001	3000	1	1.66E-06	-0.6451725	54	1.8	RBM14-RBM4;RBM14;RBM4	Translation
DMR11:67083001	11	67083001	2000	1	2.36E-07	-0.6623761	34	1.7		
DMR11:67306001	11	67306001	1000	1	1.57E-06	-0.5931752	27	2.7	ANKRD13D;SSH3	Signaling
DMR11:68451001	11	68451001	4000	1	1.11E-08	-0.687906	99	2.475	LRP5;PPP6R3	Receptor;Signaling
DMR11:68679001	11	68679001	3000	1	8.13E-06	-0.5887021	73	2.433333333	LOC107984343;GAL	
DMR11:70595001	11	70595001	1000	1	3.37E-06	0.7576052	31	3.1	SHANK2	
DMR11:71410001	11	71410001	1000	1	7.67E-06	0.9477624	11	1.1	ACTE1P	
DMR11:73251001	11	73251001	2000	1	6.04E-16	-2.3626752	26	1.3	P2RY2;OR8R1P;LOC105369382	Signaling
DMR11:73982001	11	73982001	1000	1	7.91E-06	-0.6005098	67	6.7	UCP2;LOC100037267	Transport
DMR11:74030001	11	74030001	1000	1	1.65E-06	0.6523502	13	1.3	C2CD3	
DMR11:75360001	11	75360001	2000	1	1.29E-07	-0.7809176	25	1.25	ARRB1	Cytoskeleton
DMR11:76885001	11	76885001	2000	1	6.84E-07	0.7518684	19	0.95	ACER3	
DMR11:78107001	11	78107001	1000	1	3.15E-06	-0.5368737	18	1.8	ALG8	Golgi
DMR11:79190001	11	79190001	2000	1	4.66E-08	-0.8309341	87	4.35	TENM4	
DMR11:79821001	11	79821001	1000	1	6.06E-06	0.7016573	11	1.1		
DMR11:81553001	11	81553001	1000	1	9.82E-08	0.9859911	8	0.8	MTCO3P25;MTND3P11;MTND4P36;MTND5P38;MTND6P25;MTCYBP25	
DMR11:85450001	11	85450001	2000	1	2.15E-06	0.8342614	10	0.5	DLG2;LOC100421303	Cytoskeleton
DMR11:90958001	11	90958001	1000	1	9.78E-06	0.7539862	9	0.9		
DMR11:92850001	11	92850001	1000	1	1.54E-06	0.9689058	9	0.9	FAT3	Cytoskeleton
DMR11:93650001	11	93650001	1000	1	4.94E-07	0.6469741	8	0.8		
DMR11:96445001	11	96445001	1000	1	4.93E-07	-0.825973	6	0.6	JRKL-AS1	
DMR11:96555001	11	96555001	1000	1	4.31E-07	-1.146636	17	1.7		
DMR11:99180001	11	99180001	1000	1	9.59E-06	0.6761952	17	1.7	CNTN5	
DMR11:99405001	11	99405001	1000	1	2.89E-06	-0.8138109	26	2.6	CNTN5	
DMR11:101046001	11	101046001	1000	1	4.21E-06	-0.7202048	5	0.5	PGR	
DMR11:102730001	11	102730001	2000	1	1.38E-06	-0.5054646	20	1	MMP8	Protease
DMR11:103402001	11	103402001	2000	1	2.26E-06	0.8337759	16	0.8	DYNC2H1;MTCO3P15;MTATP6P15;MTCO2P15;MTCO1P15;MTND2P26;MTND1P36	Cytoskeleton
DMR11:105748001	11	105748001	2000	1	6.94E-06	0.5536904	26	1.3	GRIA4	Receptor
DMR11:108132001	11	108132001	1000	1	1.03E-06	-0.6919443	13	1.3	ACAT1	Metabolism
DMR11:110060001	11	110060001	1000	1	8.11E-06	0.8199733	6	0.6	LOC107984384;LOC390250	
DMR11:113517001	11	113517001	1000	1	7.94E-07	1.0403823	7	0.7		
DMR11:113874001	11	113874001	1000	1	7.54E-08	-0.6547847	37	3.7	USP28	Protease
DMR11:114269001	11	114269001	1000	1	9.12E-06	0.762692	6	0.6	NNMT	Epigenetic
DMR11:117133001	11	117133001	1000	1	2.65E-08	-0.699004	24	2.4	LOC101060089;LOC653303	
DMR11:117320001	11	117320001	2000	1	7.67E-07	-0.6022191	29	1.45	BACE1;CEP164	Protease;Cytoskeleton
DMR11:117486001	11	117486001	3000	1	8.49E-06	-0.4932495	44	1.466666667	DSCAML1	Cytoskeleton
DMR11:118608001	11	118608001	3000	1	4.28E-06	-0.652523	232	7.733333333	ARCN1;PHLDB1	Transport
DMR11:120106001	11	120106001	2000	1	7.05E-06	0.5191131	26	1.3	LOC105378956;TRIM29	
DMR11:122017001	11	122017001	1000	1	5.59E-08	-1.7553254	7	0.7		
DMR11:122786001	11	122786001	1000	1	8.25E-07	-0.5690713	22	2.2	UBASH3B	
DMR11:124923001	11	124923001	2000	1	2.48E-06	1.0338639	32	1.6	LOC107984406;HEPACAM;HEPN1	Immune
DMR11:126237001	11	126237001	1000	1	4.32E-06	-0.6148765	24	2.4	FAM118B;RN7SL351P	
DMR11:128059001	11	128059001	1000	1	2.84E-06	0.7082516	8	0.8	LOC107984374	
DMR11:128548001	11	128548001	1000	1	4.36E-06	-0.5499246	32	3.2	ETS1	Transcription
DMR11:128868001	11	128868001	1000	1	2.15E-08	-1.4028138	14	1.4	KCNJ1;LOC107984409	Transport
DMR11:128969001	11	128969001	2000	1	8.68E-06	0.5764303	53	2.65	ARHGAP32	Signaling
DMR11:129034001	11	129034001	1000	1	5.91E-11	-0.8980627	6	0.6	ARHGAP32	Signaling
DMR11:129526001	11	129526001	1000	1	4.63E-06	0.6237291	17	1.7		

DMR11:131922001	11	131922001	2000	1	2.89E-06	0.5279458	28	1.4	NTM	Immune
DMR11:132205001	11	132205001	1000	1	9.21E-06	1.0091811	12	1.2	NTM	Immune
DMR11:133027001	11	133027001	1000	1	6.81E-06	-0.5287847	7	0.7	OPCML	Immune
DMR11:134092001	11	134092001	3000	1	8.77E-06	0.7818342	25	0.833333333	JAM3;LOC107984040	
DMR11:134656001	11	134656001	1000	1	7.97E-07	-0.5056956	35	3.5	LOC105369583	
DMR12:103001	12	103001	1000	1	2.82E-07	-0.6090807	26	2.6	IQSEC3;LOC105369593	Transcription
DMR12:511001	12	511001	3000	1	3.35E-08	-0.5517704	72	2.4	B4GALNT3	Golgi
DMR12:1483001	12	1483001	2000	1	8.33E-06	-0.586688	51	2.55	ERC1	Transport
DMR12:1573001	12	1573001	2000	1	6.02E-06	-0.4265704	33	1.65	WNT5B;FBXL14	Signaling
DMR12:1871001	12	1871001	1000	1	2.55E-06	0.5830932	25	2.5	CACNA2D4;LAMP1P1	Transport
DMR12:2668001	12	2668001	2000	1	6.02E-08	0.8672834	39	1.95	CACNA1C;RPS6P18;CACNA1C-AS2;CACNA1C-AS1	Transport
DMR12:2910001	12	2910001	2000	1	4.21E-06	-0.6408749	34	1.7	TULP3	
DMR12:4253001	12	4253001	1000	1	5.89E-06	0.7069973	18	1.8	CCND2-AS1	
DMR12:5279001	12	5279001	2000	1	3.19E-07	1.1357779	20	1	LOC105369617	
DMR12:6079001	12	6079001	1000	1	1.00E-05	-0.7252891	16	1.6	VWF	Extracellular Matrix
DMR12:6567001	12	6567001	1000	1	2.28E-06	0.7131791	27	2.7	NOP2;CHD4	
DMR12:6721001	12	6721001	2000	1	9.40E-06	-0.4402187	63	3.15	COPS7A	
DMR12:7761001	12	7761001	1000	1	1.40E-06	-0.5291458	33	3.3	CLEC4C;NANOGNB	Transport
DMR12:8039001	12	8039001	1000	1	2.74E-06	1.0435292	13	1.3	FOXJ2	
DMR12:9223001	12	9223001	1000	1	1.32E-06	0.8577781	12	1.2	KLRG1;A2MP1	Receptor
DMR12:9585001	12	9585001	1000	1	2.81E-06	-0.7189898	9	0.9	LOC408186;KLRB1	Receptor
DMR12:9775001	12	9775001	2000	1	9.44E-06	-0.6311313	26	1.3		
DMR12:10312001	12	10312001	1000	1	2.32E-10	-0.7292355	37	3.7	KLRD1	
DMR12:12262001	12	12262001	2000	1	2.67E-07	-0.6981414	40	2	LRP6	Binding Proteins
DMR12:12415001	12	12415001	1000	1	5.49E-06	-0.5420359	39	3.9	BORCS5	
DMR12:13881001	12	13881001	1000	1	4.76E-06	0.3693574	16	1.6	GRIN2B	Receptor
DMR12:15215001	12	15215001	1000	1	6.44E-06	0.5612042	13	1.3	RERG	Signaling
DMR12:16361001	12	16361001	1000	1	6.18E-07	0.9559386	10	1	MGST1	Transport
DMR12:16505001	12	16505001	1000	1	5.97E-06	0.7967009	7	0.7	GOT2P4	
DMR12:18038001	12	18038001	1000	1	8.44E-06	0.4732471	10	1		
DMR12:19595001	12	19595001	1000	1	9.64E-06	-0.6522131	13	1.3	LOC101928387	
DMR12:23872001	12	23872001	1000	1	2.90E-11	-0.8341535	22	2.2	SOX5	
DMR12:25628001	12	25628001	1000	1	2.24E-06	-0.8575681	11	1.1	LMNTD1	
DMR12:27313001	12	27313001	1000	1	3.90E-07	-0.7554936	21	2.1	STK38L	Signaling
DMR12:34359001	12	34359001	3000	1	3.31E-06	0.6708446	131	4.366666667		
DMR12:34833001	12	34833001	3000	2	1.06E-14	-0.7925659	41	1.366666667		
DMR12:37260001	12	37260001	4000	1	3.85E-06	-0.5502984	53	1.325		
DMR12:38854001	12	38854001	1000	1	3.41E-07	0.713629	9	0.9	CPNE8	
DMR12:43492001	12	43492001	1000	1	1.07E-07	-0.6048776	24	2.4	ADAMTS20	Protease
DMR12:44504001	12	44504001	1000	1	8.13E-09	0.9714515	6	0.6	NELL2	Signaling
DMR12:45926001	12	45926001	1000	1	8.44E-06	0.658713	12	1.2	SCAF11	
DMR12:46322001	12	46322001	4000	1	4.31E-06	0.5937114	27	0.675		
DMR12:47173001	12	47173001	1000	1	6.79E-07	0.7438156	17	1.7	PCED1B	
DMR12:49212001	12	49212001	1000	1	7.58E-06	-0.6107367	24	2.4		
DMR12:49467001	12	49467001	3000	1	1.79E-07	-0.684277	47	1.566666667	SPATS2	
DMR12:49875001	12	49875001	6000	1	1.08E-09	0.6721935	84	1.4	FAIM2	
DMR12:50905001	12	50905001	1000	1	6.11E-07	0.6709895	16	1.6		
DMR12:51294001	12	51294001	2000	1	1.31E-07	-0.7835225	41	2.05	BIN2	
DMR12:51612001	12	51612001	2000	1	1.21E-07	0.825831	13	0.65	SCN8A	Transport
DMR12:53003001	12	53003001	1000	1	8.38E-06	-0.4736029	53	5.3	EIF4B	Translation
DMR12:53384001	12	53384001	2000	1	5.06E-06	-0.5305607	70	3.5	SP1	Transcription
DMR12:53407001	12	53407001	2000	1	9.51E-09	-0.587436	63	3.15	SP1	Transcription
DMR12:54219001	12	54219001	1000	1	3.70E-06	-0.5002532	40	4		
DMR12:55810001	12	55810001	1000	1	4.08E-06	-0.775928	19	1.9	SARNP;ORMDL2;DNAJC14	Transcription
DMR12:55960001	12	55960001	3000	2	7.50E-08	-0.7061237	72	2.4	DGKA;PMEL;CDK2	Signaling;Signaling;Signaling
DMR12:56049001	12	56049001	9000	1	2.05E-06	-0.5216186	180	2	LOC105369780;RPS26	Translation
DMR12:56068001	12	56068001	1000	1	1.93E-06	-0.6502263	37	3.7		
DMR12:56303001	12	56303001	1000	1	1.08E-06	-0.6511131	21	2.1	CS;CNPY2;CNPY2-AS1	Transport
DMR12:56321001	12	56321001	1000	1	9.10E-07	-0.6662896	13	1.3	CNPY2;CNPY2-AS1;PAN2	Transcription
DMR12:56558001	12	56558001	2000	1	3.46E-06	-0.5283371	51	2.55	RBMS2	
DMR12:57264001	12	57264001	1000	1	2.25E-07	-1.0494783	14	1.4	R3HDM2	
DMR12:57884001	12	57884001	2000	1	3.18E-06	-0.5459179	32	1.6	LOC283387;LOC101927608	
DMR12:58345001	12	58345001	1000	1	2.96E-06	0.8539545	8	0.8	LOC105369787	
DMR12:62492001	12	62492001	1000	1	1.61E-06	-0.4615133	37	3.7	MON2	Transcription
DMR12:63169001	12	63169001	1000	1	4.42E-10	-1.0910098	6	0.6		
DMR12:63806001	12	63806001	2000	1	5.18E-06	0.4320347	20	1	RXYLT1;RXYLT1-AS1	Transport
DMR12:66057001	12	66057001	1000	1	2.63E-07	-0.7103541	9	0.9	RNA5SP362	

DMR12:66286001	12	66286001	1000	1	3.90E-06	0.5765285	11	1.1	PDCL3P7;RN7SKP166	
DMR12:67260001	12	67260001	1000	1	9.22E-06	-0.8326135	5	0.5	GGTA2P;CAND1	Proteolysis
DMR12:67629001	12	67629001	1000	1	2.60E-06	0.6317842	14	1.4		
DMR12:78000001	12	78000001	1000	1	2.34E-06	-0.6186269	36	3.6	NAV3	
DMR12:79958001	12	79958001	1000	1	2.31E-06	-0.5876265	26	2.6		
DMR12:80397001	12	80397001	1000	1	3.05E-06	-0.5936033	14	1.4		
DMR12:81703001	12	81703001	1000	1	7.44E-06	0.5895824	7	0.7	PPFIA2	
DMR12:82798001	12	82798001	1000	1	3.79E-07	-0.4808527	33	3.3	TMTC2	Golgi
DMR12:85723001	12	85723001	1000	1	8.60E-07	-1.0251178	4	0.4		
DMR12:87596001	12	87596001	1000	1	8.40E-07	0.8633064	6	0.6		
DMR12:88669001	12	88669001	1000	1	3.56E-06	0.8239727	4	0.4		
DMR12:88715001	12	88715001	1000	1	1.07E-07	-1.4687968	3	0.3		
DMR12:90345001	12	90345001	1000	1	1.48E-06	0.7579834	10	1		
DMR12:94013001	12	94013001	2000	1	3.93E-08	-0.5719249	32	1.6	LOC105369912;RN7SKP263	
DMR12:95072001	12	95072001	1000	1	3.80E-06	-0.6854815	51	5.1	NR2C1;FGD6	Transcription;Transcription
DMR12:95570001	12	95570001	2000	1	2.15E-07	-0.7998988	21	1.05		
DMR12:95643001	12	95643001	3000	1	2.63E-06	-0.5957417	28	0.933333333	PGAM1P5	
DMR12:96458001	12	96458001	2000	1	2.66E-06	-0.569557	35	1.75		
DMR12:96485001	12	96485001	2000	1	8.49E-06	-0.5843919	20	1	CFAP54	Development
DMR12:98455001	12	98455001	1000	1	1.86E-06	0.7007859	30	3	SLC9A7P1	
DMR12:100030001	12	100030001	1000	1	4.75E-06	-0.7069244	11	1.1	UHRF1BP1L	
DMR12:100147001	12	100147001	1000	1	2.12E-07	-0.6714059	21	2.1	UHRF1BP1L;DNM1P19;GOLGA2P5	
DMR12:100778001	12	100778001	1000	1	8.89E-06	-0.8218745	19	1.9	ANO4	
DMR12:101340001	12	101340001	1000	1	1.73E-06	-0.7542637	7	0.7	UTP20	
DMR12:103774001	12	103774001	1000	1	1.64E-06	-0.6968621	14	1.4	STAB2;NT5DC3	Transport;Signaling
DMR12:107215001	12	107215001	2000	1	5.43E-07	0.5342099	29	1.45		
DMR12:107736001	12	107736001	1000	1	6.31E-06	0.9155934	7	0.7	PRDM4;LOC101929162	Transcription
DMR12:110144001	12	110144001	2000	1	1.29E-07	-0.8810976	29	1.45	IFT81	
DMR12:110309001	12	110309001	1000	1	4.46E-09	-0.8427397	15	1.5	ATP2A2	Transport
DMR12:110363001	12	110363001	3000	1	5.09E-07	-0.6036797	53	1.766666667	RN7SL769P;ANAPC7	Proteolysis
DMR12:110413001	12	110413001	2000	1	9.91E-06	-0.5327186	46	2.3	ANAPC7	Proteolysis
DMR12:111605001	12	111605001	1000	1	3.92E-06	-0.5676865	28	2.8	ATXN2;ATXN2-AS	Metabolism
DMR12:111969001	12	111969001	1000	1	9.33E-07	-0.5653493	32	3.2	TMEM116	Signaling
DMR12:112044001	12	112044001	1000	1	1.30E-07	-0.6881433	21	2.1	NAA25;MIR3657	Metabolism
DMR12:112714001	12	112714001	1000	1	1.92E-08	0.8662617	13	1.3	RPH3A	
DMR12:113765001	12	113765001	1000	1	3.52E-06	0.5782426	11	1.1	LINC01234	
DMR12:114893001	12	114893001	1000	1	3.31E-06	-0.7419191	12	1.2		
DMR12:115245001	12	115245001	1000	1	4.34E-06	0.7967117	18	1.8		
DMR12:115991001	12	115991001	1000	1	1.84E-06	0.5950056	25	2.5	MED13L	
DMR12:116026001	12	116026001	1000	1	7.08E-06	0.6151881	5	0.5	MED13L	
DMR12:118096001	12	118096001	1000	1	4.30E-06	-0.5225127	34	3.4	VSIG10	
DMR12:119689001	12	119689001	1000	1	3.40E-07	-0.5860313	26	2.6	PRKAB1;CIT	Signaling;Signaling
DMR12:120203001	12	120203001	3000	1	9.88E-06	-0.5582659	67	2.233333333	GCN1;RPLP0;PXN-AS1;PXN	Cytoskeleton;Translation;Cytoskeleton
DMR12:120269001	12	120269001	1000	1	7.02E-06	-0.5995048	30	3	PXN	Cytoskeleton
DMR12:120553001	12	120553001	1000	1	4.91E-06	-0.5669134	24	2.4	RNF10	
DMR12:120600001	12	120600001	2000	1	8.31E-07	-0.676959	52	2.6	RPL11P5	
DMR12:121320001	12	121320001	2000	1	7.39E-06	-0.8347377	34	1.7	ANAPC5	
DMR12:121527001	12	121527001	1000	1	3.17E-06	-0.6120527	27	2.7	KDM2B	
DMR12:121747001	12	121747001	2000	1	2.06E-06	-0.7072062	55	2.75	TMEM120B	
DMR12:121891001	12	121891001	1000	1	3.03E-06	-0.5159996	32	3.2	HPD;PSMD9	Metabolism;Protease
DMR12:121920001	12	121920001	2000	1	2.02E-06	-0.5392701	39	1.95	PSMD9;CFAP251	Protease
DMR12:122093001	12	122093001	3000	2	8.05E-08	-0.5113486	34	1.133333333	MLXIP	Transcription
DMR12:122268001	12	122268001	1000	1	8.22E-10	-0.6075256	41	4.1	VPS33A;CLIP1	Transport;Transcription
DMR12:122355001	12	122355001	1000	1	7.99E-06	-0.5079336	34	3.4	CLIP1;RPL21P1	Transcription
DMR12:122467001	12	122467001	2000	1	3.49E-08	-0.6169717	76	3.8	ZCCHC8	Metabolism
DMR12:122640001	12	122640001	2000	1	1.79E-07	-0.6602572	46	2.3		
DMR12:122825001	12	122825001	1000	1	1.15E-06	-0.7660123	34	3.4	CCDC62;HIP1R	Cytoskeleton
DMR12:123276001	12	123276001	5000	1	6.23E-08	-0.5337873	122	2.44	CDK2AP1;RNA5SP375;SBNO1	Signaling
DMR12:123446001	12	123446001	1000	1	1.52E-07	-0.8410977	16	1.6	RILPL2;COPS5P2	
DMR12:123509001	12	123509001	5000	1	3.67E-08	-0.5225981	77	1.54	RILPL1	
DMR12:123679001	12	123679001	2000	1	2.62E-06	-0.7160593	46	2.3	TCTN2	
DMR12:124001001	12	124001001	1000	1	9.50E-06	0.8231163	8	0.8	ZNF664-RFLNA;ZNF664	Transcription
DMR12:124180001	12	124180001	1000	1	5.82E-07	0.7967788	17	1.7	ZNF664-RFLNA	
DMR12:126425001	12	126425001	1000	1	5.92E-07	1.0869352	15	1.5	LINC02825;LINC02350	
DMR12:128420001	12	128420001	1000	1	6.28E-06	0.5287048	9	0.9	TMEM132C	
DMR12:128645001	12	128645001	4000	1	6.30E-14	0.7092223	67	1.675	TMEM132C	
DMR12:129747001	12	129747001	2000	1	1.66E-06	-0.4479072	30	1.5	TMEM132D	

DMR12:129834001	12	129834001	1000	1	5.01E-07	0.7569652	15	1.5	TMEM132D	
DMR12:130055001	12	130055001	1000	1	4.29E-06	-0.4293595	8	0.8		
DMR12:130241001	12	130241001	2000	1	8.21E-08	0.600129	27	1.35		
DMR12:130926001	12	130926001	1000	1	9.82E-06	-0.6706132	19	1.9		
DMR12:131917001	12	131917001	3000	1	4.63E-06	-0.8390037	125	4.166666667	ULK1;LOC107984455;PUS1	Signaling;Metabolism
DMR12:132381001	12	132381001	3000	1	1.78E-06	0.6822597	48	1.6		
DMR12:132436001	12	132436001	1000	1	3.84E-06	0.6007864	39	3.9	LOC105370092;LOC112268102	
DMR12:132454001	12	132454001	1000	1	5.81E-07	-0.5338346	19	1.9		
DMR12:132539001	12	132539001	2000	1	4.08E-06	-0.5568518	49	2.45	FBRSL1	
DMR12:132868001	12	132868001	3000	1	6.49E-06	-0.4674133	105	3.5	CHFR	Proteolysis
DMR12:132938001	12	132938001	1000	1	2.29E-06	-0.6613898	47	4.7	ZNF605	
DMR12:132945001	12	132945001	1000	1	1.87E-06	0.6377299	16	1.6	ZNF605	
DMR13:19922001	13	19922001	2000	1	1.52E-06	-0.7011196	59	2.95		
DMR13:20229001	13	20229001	1000	1	5.81E-06	-0.6321279	13	1.3	GJB6	Cytoskeleton
DMR13:20391001	13	20391001	5000	2	2.73E-06	-0.6250957	187	3.74	CRYL1	Metabolism
DMR13:20958001	13	20958001	1000	1	7.69E-07	-0.7866779	19	1.9	LINC00367;HNRNPA1P30;PPIAP27;RPSAP54	
DMR13:21147001	13	21147001	1000	1	9.13E-06	0.6829321	12	1.2	SAP18;SKA3	Epigenetic
DMR13:21936001	13	21936001	1000	1	5.03E-06	-0.407741	6	0.6		
DMR13:22793001	13	22793001	2000	1	7.21E-07	0.8194719	31	1.55	RPL7AP73	
DMR13:22933001	13	22933001	2000	1	4.55E-06	-0.4719537	53	2.65		
DMR13:25159001	13	25159001	1000	1	2.37E-09	-1.6018671	6	0.6	AMER2	
DMR13:28799001	13	28799001	1000	1	1.70E-10	-0.9882593	14	1.4		
DMR13:29491001	13	29491001	2000	1	1.13E-06	-0.4320936	19	0.95	MTUS2;MTUS2-AS1	
DMR13:29687001	13	29687001	1000	1	1.47E-07	0.7837879	6	0.6	LOC105370139	
DMR13:30471001	13	30471001	1000	1	6.28E-06	-0.4715354	28	2.8	HMGB1	
DMR13:33385001	13	33385001	1000	1	5.02E-10	-1.0438404	13	1.3	STARD13	Signaling
DMR13:40363001	13	40363001	1000	1	5.45E-07	0.6865796	8	0.8	LINC00598	
DMR13:43806001	13	43806001	1000	1	1.55E-07	-0.7032378	27	2.7	LOC105370181	
DMR13:45419001	13	45419001	1000	1	1.13E-06	-0.748028	33	3.3	SLC25A30;SLC25A30-AS1	Transport
DMR13:46702001	13	46702001	2000	1	9.98E-06	0.7050303	21	1.05	LRCH1	
DMR13:55717001	13	55717001	1000	1	7.21E-06	-0.7137754	3	0.3		
DMR13:61729001	13	61729001	2000	1	9.80E-09	-0.7754609	27	1.35		
DMR13:65449001	13	65449001	1000	1	1.72E-25	-3.4124046	2	0.2		
DMR13:72853001	13	72853001	1000	1	3.72E-06	0.5821019	5	0.5	PIBF1	
DMR13:75223001	13	75223001	1000	1	6.28E-06	0.8556607	10	1		
DMR13:77933001	13	77933001	2000	1	5.46E-06	0.4643772	26	1.3	EDNRB	
DMR13:78648001	13	78648001	3000	1	2.50E-07	-0.8553283	24	0.8	OBI1	
DMR13:79278001	13	79278001	2000	1	7.02E-06	0.5388781	34	1.7		
DMR13:85951001	13	85951001	2000	1	2.90E-07	-0.8040115	31	1.55		
DMR13:86369001	13	86369001	1000	1	3.27E-06	0.6731622	15	1.5		
DMR13:88437001	13	88437001	2000	1	1.24E-07	-0.6977397	28	1.4		
DMR13:89055001	13	89055001	1000	1	7.16E-06	0.773367	12	1.2	LOC105370307	
DMR13:90349001	13	90349001	1000	1	3.03E-08	0.618348	27	2.7		
DMR13:91542001	13	91542001	1000	1	3.89E-07	-0.6195448	15	1.5	GPC5	
DMR13:95192001	13	95192001	2000	1	3.53E-06	0.9181853	32	1.6	ABCC4	Transport
DMR13:97260001	13	97260001	3000	1	8.79E-06	0.939899	38	1.266666667	MBNL2;LOC101927385	Translation
DMR13:98319001	13	98319001	1000	1	2.08E-06	-0.8804326	16	1.6	FARP1;LOC105370327	
DMR13:100611001	13	100611001	1000	1	4.63E-08	-0.7052482	18	1.8	TMTC4	
DMR13:103014001	13	103014001	1000	1	1.50E-06	0.7830597	8	0.8		
DMR13:103309001	13	103309001	1000	1	8.79E-06	0.6067159	8	0.8	LOC105370338	
DMR13:104339001	13	104339001	1000	1	3.79E-06	0.56435	10	1		
DMR13:106125001	13	106125001	1000	1	9.86E-06	0.7401703	17	1.7		
DMR13:108608001	13	108608001	1000	1	4.91E-08	0.7533719	14	1.4	MYO16	
DMR13:109085001	13	109085001	1000	1	1.08E-06	0.509774	16	1.6	MYO16	
DMR13:109152001	13	109152001	2000	1	8.18E-08	0.936357	38	1.9	MYO16;LOC105370356;MYO16-AS1	
DMR13:109300001	13	109300001	1000	1	1.25E-07	-0.6121194	16	1.6	LINC00370	
DMR13:109408001	13	109408001	2000	1	1.12E-06	0.7201018	27	1.35	LOC105370359;LINC00399	
DMR13:111507001	13	111507001	1000	1	9.12E-06	-0.6050672	17	1.7		
DMR13:113056001	13	113056001	3000	1	1.81E-06	-0.3960246	53	1.766666667	MCF2L	Transcription
DMR13:113984001	13	113984001	3000	1	6.54E-06	-0.6703305	39	1.3	C13orf46;RASA3	Signaling
DMR13:114042001	13	114042001	3000	2	1.56E-07	-0.9735965	161	5.366666667	RASA3	Signaling
DMR13:114047001	13	114047001	2000	1	7.20E-07	0.7189126	93	4.65	RASA3	Signaling
DMR13:114155001	13	114155001	2000	1	9.21E-06	-0.7123836	32	1.6		
DMR14:16029001	14	16029001	1000	1	5.03E-06	-0.42192	51	5.1	DUX4L48;PCMTD1P6;DUX4L49	
DMR14:16093001	14	16093001	2000	2	1.48E-19	-1.6192069	5	0.25		
DMR14:16096001	14	16096001	10000	3	6.29E-13	-0.6534065	143	1.43		
DMR14:18364001	14	18364001	1000	1	3.44E-08	-1.1327452	9	0.9		
DMR14:20834001	14	20834001	1000	1	8.33E-06	0.6431826	17	1.7		

DMR14:21296001	14	21296001	1000	1	5.79E-06	-0.5590422	25	2.5	RPGRIP1;MRPS17P6	Cytoskeleton
DMR14:21609001	14	21609001	1000	1	1.68E-07	-0.9787269	15	1.5	OR10G1P;UBE2NP1	
DMR14:22643001	14	22643001	1000	1	8.46E-07	-0.694067	13	1.3	OR6J1;LOC107984651	Receptor
DMR14:23347001	14	23347001	5000	1	2.61E-07	0.6512523	83	1.66	SLC22A17;EFS	Transport
DMR14:23705001	14	23705001	1000	1	7.80E-06	-0.8225365	18	1.8	LOC105370408;LOC105370410;LOC105370409	
DMR14:25680001	14	25680001	1000	1	2.45E-06	0.6735167	18	1.8		
DMR14:30776001	14	30776001	1000	1	4.99E-08	0.886449	13	1.3		
DMR14:31037001	14	31037001	1000	1	8.50E-06	-0.5381154	9	0.9	AP4S1	Transport
DMR14:31137001	14	31137001	1000	1	1.47E-06	-0.7123672	17	1.7	HECTD1;RNU6-541P	Proteolysis
DMR14:32908001	14	32908001	1000	1	5.51E-06	0.7514793	16	1.6		
DMR14:34205001	14	34205001	1000	1	5.68E-08	-0.7118703	25	2.5	LOC102724945	
DMR14:35132001	14	35132001	1000	1	1.21E-06	-0.528941	28	2.8	PPP2R3C;PRORP	Signaling
DMR14:35291001	14	35291001	1000	1	1.69E-06	-0.5995679	46	4.6	PSMA6	Protease
DMR14:36182001	14	36182001	1000	1	5.64E-07	-0.779257	16	1.6	PTCSC3	
DMR14:39418001	14	39418001	1000	1	3.15E-07	-0.5043599	25	2.5	FBXO33	
DMR14:45059001	14	45059001	2000	1	1.98E-06	-0.8182144	23	1.15	TOGARAM1	Cytoskeleton
DMR14:48201001	14	48201001	2000	1	8.33E-06	0.3905376	27	1.35	LOC101927483	
DMR14:49772001	14	49772001	2000	1	6.20E-06	-0.6725066	30	1.5	KLHDC2;NEMF	
DMR14:52701001	14	52701001	2000	1	4.34E-06	-0.7328508	30	1.5	ERO1A;PSMC6	Metabolism;Protease
DMR14:53181001	14	53181001	1000	1	1.11E-07	-0.4883426	18	1.8	LOC105370502	
DMR14:55043001	14	55043001	1000	1	2.72E-08	0.7476546	18	1.8	SOCS4;MAPK1IP1L	Signaling
DMR14:55489001	14	55489001	1000	1	1.98E-09	-1.3042244	9	0.9		
DMR14:58317001	14	58317001	1000	1	2.95E-09	-0.6681927	35	3.5	ARID4A	Transcription
DMR14:60143001	14	60143001	1000	1	7.88E-06	1.1451971	5	0.5	PCNX4;DHRS7	Metabolism
DMR14:61134001	14	61134001	2000	1	9.36E-06	0.5212454	17	0.85		
DMR14:62258001	14	62258001	1000	1	7.98E-06	0.8070403	9	0.9		
DMR14:63630001	14	63630001	1000	1	1.41E-07	-0.8252646	21	2.1	WDR89;RNU6-1162P	
DMR14:63688001	14	63688001	1000	1	7.27E-06	-0.6104256	26	2.6	SGPP1	Signaling
DMR14:64370001	14	64370001	1000	1	3.48E-06	-0.5804016	22	2.2		
DMR14:65997001	14	65997001	1000	1	6.29E-06	-0.6566834	16	1.6		
DMR14:67814001	14	67814001	1000	1	4.76E-06	-0.937592	12	1.2	ZFYVE26;RAD51B	Transcription
DMR14:68572001	14	68572001	1000	1	9.09E-06	0.9699516	13	1.3	RAD51B	Transcription
DMR14:68673001	14	68673001	1000	1	3.69E-07	1.1988048	7	0.7	RAD51B	Transcription
DMR14:71195001	14	71195001	2000	1	9.71E-06	0.6627149	20	1	LOC107984695;LOC105370706	
DMR14:72988001	14	72988001	1000	1	6.51E-08	-0.952245	17	1.7	ZFYVE1	
DMR14:73579001	14	73579001	1000	1	7.29E-07	-0.5376142	25	2.5	ACOT2;NT5CP1;TRK-TTT14-1	Metabolism
DMR14:76266001	14	76266001	1000	1	4.70E-07	-0.5641709	14	1.4	LOC105370575	
DMR14:77019001	14	77019001	1000	1	5.42E-06	-0.6627421	30	3	IRF2BPL;LOC107984638	Transcription
DMR14:77220001	14	77220001	2000	2	6.58E-09	-0.8419995	66	3.3	TMEM63C	
DMR14:77269001	14	77269001	1000	1	5.20E-06	-0.56151	14	1.4	TMEM63C;NGB;MIR1260A;POMT2	Transport;Transport
DMR14:77783001	14	77783001	2000	1	2.60E-06	-0.6162953	45	2.25		
DMR14:79849001	14	79849001	1000	1	7.24E-06	1.0427971	10	1	NRXN3	
DMR14:81457001	14	81457001	3000	1	3.85E-07	-0.7411412	48	1.6	LINC02308	
DMR14:81604001	14	81604001	1000	1	4.25E-06	-0.9791407	9	0.9	LINC01467	
DMR14:88246001	14	88246001	1000	1	3.52E-11	-0.7814058	14	1.4	KCNK10	Transport
DMR14:89360001	14	89360001	3000	1	5.68E-06	-0.7525623	16	0.533333333	FOXN3;LOC101928817	
DMR14:89524001	14	89524001	1000	1	1.95E-06	-0.7929962	17	1.7	FOXN3	
DMR14:90528001	14	90528001	2000	1	5.27E-07	0.4948633	44	2.2	TTC7B	
DMR14:90983001	14	90983001	1000	1	1.14E-06	-0.5754993	18	1.8	RPS6KA5	Golgi
DMR14:91293001	14	91293001	4000	2	5.47E-07	0.6806442	93	2.325	CCDC88C	Transport
DMR14:92193001	14	92193001	2000	1	4.91E-06	0.7610246	31	1.55		
DMR14:92717001	14	92717001	1000	1	1.07E-06	0.6649555	19	1.9	LGMN	Protease
DMR14:94642001	14	94642001	3000	1	6.10E-07	0.7925487	56	1.866666667	ADIPOR1P2;SERPINA13P	Protease; Proteolysis
DMR14:99510001	14	99510001	1000	1	1.78E-07	-0.6725748	36	3.6	CCNK;CCDC85C	Signaling
DMR14:100378001	14	100378001	3000	1	2.25E-09	-0.7542914	70	2.333333333	WARS1;WDR25	Translation;Cytoskeleton
DMR14:101419001	14	101419001	1000	1	7.78E-08	-1.6772323	5	0.5		
DMR14:101678001	14	101678001	1000	1	1.95E-06	0.7365693	10	1	LINC02320;RPL26P4	
DMR14:102256001	14	102256001	2000	1	3.40E-06	-0.6760097	47	2.35	MOK	Signaling
DMR14:102364001	14	102364001	2000	1	1.86E-08	0.5896902	17	0.85	CINP;TECPR2	
DMR14:102378001	14	102378001	2000	1	1.70E-09	0.8376121	26	1.3	TECPR2	
DMR14:102441001	14	102441001	1000	1	7.10E-07	-0.7039902	17	1.7	TECPR2	
DMR14:102581001	14	102581001	2000	1	2.72E-06	-0.6421828	51	2.55	RN7SL546P;RCOR1	
DMR14:103272001	14	103272001	2000	1	6.65E-06	-0.6129802	130	6.5	RAP2CP1;RPL17P4	
DMR14:104242001	14	104242001	1000	1	1.24E-06	-0.6744813	17	1.7	LINC02691	
DMR14:104486001	14	104486001	1000	1	8.16E-07	0.7563422	22	2.2		
DMR14:104673001	14	104673001	3000	1	3.05E-06	-0.561316	44	1.466666667	LINC02280;MIR4710	
DMR14:104855001	14	104855001	1000	1	3.48E-06	-0.5641176	15	1.5	CEP170B;MIR12121	Cytoskeleton

DMR14:105346001	14	105346001	1000	1	7.55E-06	-0.6062567	43	4.3	PACS2	
DMR14:105513001	14	105513001	1000	1	2.65E-06	-0.7689414	22	2.2		
DMR14:105562001	14	105562001	3000	2	1.41E-10	-0.5502818	44	1.466666667	LOC105370698	
DMR14:106592001	14	106592001	1000	1	3.15E-06	0.880407	26	2.6	IGH;IGHV8-51-1;IGHVII-51-2;IGHV3-52;IGHV3-53;IGHVII-53-1;IGHV3-54	Immune
DMR15:22214001	15	22214001	4000	1	2.90E-07	-0.9064347	86	2.15	LOC101928039;MIR1268A	
DMR15:22449001	15	22449001	3000	1	6.75E-06	-0.4988911	104	3.466666667	LOC100631266;GOLGA6L22	Cytoskeleton
DMR15:22598001	15	22598001	2000	1	1.62E-06	-0.7049651	67	3.35	HERC2P2;LOC101927846;RN7SL495P	
DMR15:22783001	15	22783001	1000	1	3.62E-06	-0.7303367	27	2.7	LOC283683;LOC729900;NIPA1	
DMR15:23064001	15	23064001	3000	1	1.75E-08	-0.5092752	89	2.966666667		
DMR15:23175001	15	23175001	3000	1	3.07E-06	-0.6328348	117	3.9	GOLGA8DP;RN7SL106P;ABCB10P1	Transport
DMR15:23372001	15	23372001	2000	1	5.86E-08	-0.5787476	78	3.9	GOLGA8S;LOC105370726;RN7SL536P	Transport
DMR15:24902001	15	24902001	4000	1	5.89E-07	-0.6093289	66	1.65	SNHG14;SNRPN;RPL5P1;RPS27P2	Translation
DMR15:27728001	15	27728001	2000	1	2.24E-06	0.7836291	20	1	OCA2	Transport
DMR15:27951001	15	27951001	1000	1	3.86E-08	0.8396938	19	1.9	OCA2	Transport
DMR15:28194001	15	28194001	1000	1	4.23E-08	-0.5602913	34	3.4	HERC2	Transcription
DMR15:28265001	15	28265001	1000	1	4.11E-06	0.6592655	35	3.5	HERC2	Transcription
DMR15:28559001	15	28559001	3000	2	2.96E-06	-0.4601314	70	2.333333333	LOC100132202;LOC105369220	
DMR15:28642001	15	28642001	1000	1	5.76E-06	0.7181719	26	2.6		
DMR15:28693001	15	28693001	2000	1	2.25E-07	-0.7664344	60	3	HERC2P9;GOLGA8M;LOC107984746;RN7SL719P	Transport
DMR15:28725001	15	28725001	3000	1	2.34E-08	-0.713307	92	3.066666667	GOLGA8M;WHAMMP2	Transport
DMR15:30426001	15	30426001	2000	2	1.08E-08	-0.7245442	78	3.9	LOC101927788;LOC105376704	
DMR15:30538001	15	30538001	2000	1	4.39E-13	-0.950567	80	4		
DMR15:30896001	15	30896001	3000	1	4.08E-06	-0.6039447	79	2.633333333	FAN1	
DMR15:31391001	15	31391001	1000	1	5.87E-07	-0.9894785	44	4.4	KLF13;LOC105370939	Transcription
DMR15:31728001	15	31728001	1000	1	7.57E-08	0.8625699	8	0.8	OTUD7A	Protease
DMR15:32154001	15	32154001	2000	1	1.81E-06	-0.6479877	34	1.7	CHRNA7;LOC105370754;LOC102724078	Ion Channel
DMR15:32365001	15	32365001	2000	1	1.54E-06	-0.5396051	40	2	LOC105376709;LOC112268159	
DMR15:32580001	15	32580001	1000	1	5.49E-06	-0.5396891	50	5	LINC02256;LOC101928042	
DMR15:34103001	15	34103001	1000	1	7.70E-08	0.7873549	17	1.7	EMC7;PGBD4	Epigenetic
DMR15:34949001	15	34949001	2000	1	4.62E-10	-0.8635179	23	1.15	AQR;RPL36AP8	
DMR15:34957001	15	34957001	1000	1	1.08E-06	-0.5482586	29	2.9	AQR	
DMR15:35355001	15	35355001	2000	1	6.76E-06	0.8198725	20	1	DPH6	
DMR15:35408001	15	35408001	1000	1	1.81E-07	0.6450503	5	0.5	DPH6;HNRNPA1P45	
DMR15:35726001	15	35726001	1000	1	8.36E-07	0.7411687	7	0.7	DPH6-DT	
DMR15:37660001	15	37660001	1000	1	6.17E-07	-0.5481708	37	3.7		
DMR15:39228001	15	39228001	1000	1	7.47E-08	-0.7714513	18	1.8		
DMR15:39524001	15	39524001	1000	1	5.24E-06	0.603216	10	1	LOC105370784	
DMR15:39668001	15	39668001	1000	1	8.77E-06	0.9154177	10	1	FSIP1	
DMR15:39690001	15	39690001	1000	1	5.33E-06	0.7808686	6	0.6	FSIP1	
DMR15:40050001	15	40050001	2000	1	3.43E-06	-0.5489583	25	1.25	SRP14-DT	
DMR15:40422001	15	40422001	1000	1	4.45E-08	-0.6221615	48	4.8	IVD	Metabolism
DMR15:40602001	15	40602001	1000	1	4.77E-06	-0.4353522	38	3.8	TRS-GCT4-2;KNL1	
DMR15:40664001	15	40664001	2000	1	2.54E-07	-1.058767	20	1	KNL1	
DMR15:40875001	15	40875001	1000	1	2.12E-14	-0.7230475	25	2.5	RHOV	Signaling
DMR15:41426001	15	41426001	3000	1	3.01E-07	-0.5814537	63	2.1	RTF1	
DMR15:41461001	15	41461001	1000	1	4.52E-06	-0.570388	26	2.6	RTF1	
DMR15:42111001	15	42111001	1000	1	4.03E-06	-0.6373565	13	1.3		
DMR15:44944001	15	44944001	1000	1	8.01E-07	-1.0981655	6	0.6		
DMR15:47771001	15	47771001	1000	1	1.90E-06	0.6534672	16	1.6	SEMA6D	Signaling
DMR15:47833001	15	47833001	1000	1	6.62E-06	0.8143942	6	0.6	LINC01491	
DMR15:49277001	15	49277001	1000	1	1.25E-06	-0.5438488	56	5.6	GALK2	Metabolism
DMR15:50444001	15	50444001	2000	1	4.85E-07	-0.8590224	30	1.5	USP8	Protease
DMR15:50638001	15	50638001	1000	1	2.02E-06	-0.6332885	31	3.1	TRPM7	Transport
DMR15:50785001	15	50785001	1000	1	4.02E-06	-0.618127	13	1.3		
DMR15:50849001	15	50849001	2000	1	3.68E-07	-1.0596971	15	0.75		
DMR15:51972001	15	51972001	1000	1	4.38E-09	-0.9840259	46	4.6	LEO1	Transcription
DMR15:54548001	15	54548001	1000	1	1.20E-06	-0.7401045	15	1.5	UNC13C	
DMR15:54926001	15	54926001	1000	1	6.51E-09	-0.6990691	9	0.9		
DMR15:59739001	15	59739001	1000	1	6.38E-06	0.8003351	19	1.9		
DMR15:59875001	15	59875001	1000	1	3.39E-06	-0.7717571	3	0.3	DDX18P2	
DMR15:59900001	15	59900001	1000	1	3.14E-06	-0.7131165	17	1.7		
DMR15:61999001	15	61999001	2000	1	5.86E-06	-0.5416253	22	1.1	VPS13C	Transport
DMR15:63123001	15	63123001	1000	1	1.30E-06	0.7593443	24	2.4	LOC107984798;LACTB	
DMR15:63158001	15	63158001	1000	1	5.12E-06	-0.7431538	30	3	RPS27L	Translation
DMR15:64801001	15	64801001	1000	1	1.53E-08	-0.7508842	19	1.9		

DMR15:64853001	15	64853001	1000	1	7.85E-06	-0.6362068	24	2.4	PLEKHO2	
DMR15:67423001	15	67423001	1000	1	2.78E-06	-0.6008803	11	1.1	IQCH;IQCH-AS1	
DMR15:68691001	15	68691001	1000	1	4.17E-06	-0.5787362	49	4.9	CORO2B	Cytoskeleton
DMR15:71075001	15	71075001	1000	1	9.45E-07	-1.1108196	7	0.7		
DMR15:71395001	15	71395001	2000	1	4.18E-07	0.9155346	34	1.7	THSD4	Protease
DMR15:74357001	15	74357001	4000	1	3.44E-06	-0.7733716	45	1.125	CYP11A1;PPIAP46	
DMR15:74366001	15	74366001	1000	1	3.63E-07	-0.6239048	59	5.9	CYP11A1;PPIAP46	
DMR15:74713001	15	74713001	1000	1	1.48E-13	-1.5260114	11	1.1	CYP1A1	Metabolism
DMR15:75120001	15	75120001	1000	1	5.68E-07	-0.5306837	42	4.2	LOC100128721	
DMR15:75530001	15	75530001	1000	1	3.81E-13	-1.3722467	10	1	PTPN9	Signaling
DMR15:75603001	15	75603001	1000	1	9.41E-07	-0.6452532	28	2.8	SNUPN	Transport
DMR15:77297001	15	77297001	2000	2	2.68E-08	-0.7010164	44	2.2	PEAK1	Signaling
DMR15:77618001	15	77618001	1000	1	4.32E-10	-0.7909022	34	3.4	LOC105370906;LINGO1	Receptor
DMR15:77982001	15	77982001	1000	1	2.04E-06	-0.6186413	19	1.9	ADAMTS7P3	
DMR15:78574001	15	78574001	1000	1	3.75E-07	-0.6374227	24	2.4	CHRNA5	Ion Channel
DMR15:79437001	15	79437001	3000	1	3.60E-06	-0.9554725	17	0.566666667	MINAR1	Receptor
DMR15:79950001	15	79950001	1000	1	4.40E-06	-0.5420532	12	1.2	FDPSP9;BCL2A1	
DMR15:80004001	15	80004001	2000	1	2.95E-24	-3.2998953	8	0.4		
DMR15:80078001	15	80078001	1000	1	8.72E-06	0.8953224	5	0.5	ZFAND6	
DMR15:80509001	15	80509001	1000	1	5.81E-06	0.5775784	11	1.1	ARNT2	Transcription
DMR15:81811001	15	81811001	1000	1	7.92E-07	0.806763	11	1.1		
DMR15:82094001	15	82094001	1000	1	7.42E-07	0.6492118	15	1.5	LINC01583;LOC105370924	
DMR15:82578001	15	82578001	3000	1	1.78E-06	-0.5580629	58	1.933333333	CPEB1	Translation
DMR15:83012001	15	83012001	1000	1	7.51E-06	0.5868478	23	2.3	C15orf40;BTBD1	Proteolysis
DMR15:83073001	15	83073001	2000	1	2.26E-08	-0.8501306	8	0.4	BTBD1;MIR4515	Proteolysis
DMR15:83230001	15	83230001	1000	1	1.05E-08	-1.4513556	34	3.4		
DMR15:84092001	15	84092001	1000	1	8.68E-12	-0.8676633	16	1.6	EFL1P1	
DMR15:86250001	15	86250001	1000	1	8.53E-06	-0.7969958	8	0.8	AGBL1	Protease
DMR15:86825001	15	86825001	1000	1	8.60E-07	-0.8974539	2	0.2	AGBL1	Protease
DMR15:88875001	15	88875001	1000	1	3.47E-06	1.1420778	17	1.7	ACAN;HAPLN3	Extracellular Matrix
DMR15:89322001	15	89322001	1000	1	2.34E-06	0.6829433	14	1.4	FANCI;POLG;MIR6766	Transcription
DMR15:89628001	15	89628001	4000	1	3.93E-06	0.8186873	120	3	TICRR;KIF7	Cytoskeleton
DMR15:90138001	15	90138001	3000	1	2.00E-06	0.7691835	54	1.8	IDH2-DT	
DMR15:90611001	15	90611001	2000	1	2.00E-07	-0.6671324	26	1.3	CRTC3;CRTC3-AS1	Transcription
DMR15:90721001	15	90721001	3000	1	1.99E-06	-0.6857414	41	1.366666667	CRTC3-AS1;BLM	Epigenetic
DMR15:93323001	15	93323001	2000	1	5.64E-07	0.7424426	16	0.8	LOC105370982	
DMR15:93999001	15	93999001	1000	1	1.87E-07	0.5142082	12	1.2	LINC01581	
DMR15:94905001	15	94905001	2000	1	4.08E-06	0.817383	16	0.8		
DMR15:97238001	15	97238001	1000	1	5.75E-06	0.7348028	14	1.4	LINC02253	
DMR15:98404001	15	98404001	1000	1	2.65E-06	0.7715165	24	2.4	LINC02351	
DMR15:99104001	15	99104001	1000	1	8.99E-07	-0.6268268	57	5.7	SYNM-AS1;SYNM	
DMR15:99491001	15	99491001	1000	1	2.61E-06	0.8291181	13	1.3	LOC107984790;LOC105371019	
DMR15:99891001	15	99891001	1000	1	6.91E-07	0.5414647	10	1	LOC400464	
DMR15:101198001	15	101198001	1000	1	9.15E-06	0.6640317	29	2.9	CHSY1	Golgi
DMR15:101869001	15	101869001	1000	1	5.46E-06	0.7435515	5	0.5	LOC105376730;OR4F28P	
DMR16:612001	16	612001	3000	1	5.08E-06	0.5494666	127	4.233333333	RAB40C	
DMR16:1345001	16	1345001	1000	1	6.57E-07	-0.7372447	27	2.7	BAIAP3;TSR3;GNPTG	Signaling
DMR16:1367001	16	1367001	3000	1	9.73E-06	-0.59905	118	3.933333333	GNPTG;UNKL;TJP1P1	Signaling
DMR16:1735001	16	1735001	7000	1	6.77E-07	0.5449789	563	8.042857143	MAPK8IP3;MIR3177	Cytoskeleton
DMR16:1842001	16	1842001	3000	1	5.83E-06	-0.6752879	69	2.3	FAHD1;MEIOB	Metabolism
DMR16:2215001	16	2215001	2000	1	2.04E-06	-0.6291692	110	5.5	MLST8;BRICD5;PGP;E4F1	Signaling;Transcription
DMR16:3367001	16	3367001	5000	1	1.35E-06	0.738659	89	1.78	OR2C1;LOC105371059;MTCO2P28;MTCO1P28;MTND2P34;MTND1P8;MTRNR2L4	Receptor
DMR16:4009001	16	4009001	1000	1	3.94E-07	0.701629	17	1.7	ADCY9	
DMR16:4196001	16	4196001	2000	1	4.27E-06	-0.7154692	33	1.65	SRL	Transport
DMR16:4674001	16	4674001	2000	1	3.15E-07	-0.5769495	50	2.5	MGRN1;MIR6769A	Proteolysis
DMR16:5286001	16	5286001	4000	1	8.43E-06	0.8433873	44	1.1	RBFOX1;SNRPCP20	Translation
DMR16:5335001	16	5335001	1000	1	1.10E-06	0.8274596	12	1.2	RBFOX1	Translation
DMR16:5901001	16	5901001	2000	1	5.02E-07	0.6806903	32	1.6	RBFOX1	Translation
DMR16:7027001	16	7027001	2000	1	3.23E-07	-0.8759821	18	0.9	RBFOX1	Translation
DMR16:8983001	16	8983001	3000	1	7.41E-07	-0.4720753	88	2.933333333		
DMR16:9143001	16	9143001	1000	1	9.30E-06	-1.0824321	7	0.7		
DMR16:9332001	16	9332001	1000	1	7.42E-07	1.0640966	18	1.8	LOC101927026	
DMR16:10444001	16	10444001	1000	1	2.96E-08	0.6399008	11	1.1	ATF7IP2;LOC107984877	Transcription
DMR16:11717001	16	11717001	1000	1	2.83E-06	-0.5870478	23	2.3	TXNDC11	Metabolism
DMR16:13313001	16	13313001	1000	1	2.54E-07	-0.5688518	20	2	SHISA9;LOC107984137	
DMR16:13840001	16	13840001	1000	1	1.13E-07	0.768917	9	0.9		

DMR16:14200001	16	14200001	1000	1	6.95E-06	0.6628062	20	2	MRTFB;TVP23CP2	Transcription
DMR16:15690001	16	15690001	1000	1	1.71E-09	-0.8728953	11	1.1	NDE1	
DMR16:16267001	16	16267001	2000	1	6.63E-06	0.6419811	28	1.4	NOMO3	
DMR16:16623001	16	16623001	1000	1	7.13E-08	-0.7540706	17	1.7		
DMR16:16721001	16	16721001	1000	1	1.93E-06	-0.6787396	19	1.9		
DMR16:17558001	16	17558001	1000	1	7.15E-06	0.6389481	15	1.5		
DMR16:18654001	16	18654001	1000	1	5.22E-06	-0.6064425	12	1.2		
DMR16:19012001	16	19012001	1000	1	2.87E-07	-0.8249612	11	1.1	TMC7;RNU6-1340P	
DMR16:19413001	16	19413001	1000	1	5.15E-06	-0.9270122	9	0.9	TMC5	
DMR16:19707001	16	19707001	1000	1	4.03E-08	-0.5452831	24	2.4	VPS35L;KNOP1	
DMR16:20721001	16	20721001	1000	1	1.28E-06	0.6239365	8	0.8	LOC100887080;THUMPD1	
DMR16:21168001	16	21168001	1000	1	5.72E-12	-1.4563379	8	0.8	DNAH3;LDAF1	Cytoskeleton
DMR16:21831001	16	21831001	1000	1	5.73E-06	-0.55925	39	3.9	LOC112268174;NPIPB4	
DMR16:22519001	16	22519001	2000	1	5.18E-06	-0.6600544	57	2.85	NPIPB5;LOC105371131	
DMR16:23453001	16	23453001	1000	1	5.46E-06	-0.680073	67	6.7	COG7;GGA2	
DMR16:23761001	16	23761001	1000	1	4.91E-06	-0.8603506	18	1.8	CHP2	
DMR16:24376001	16	24376001	1000	1	4.79E-07	-0.7055963	8	0.8		
DMR16:24664001	16	24664001	1000	1	1.74E-07	-0.9012883	13	1.3	TNRC6A;LINC01567	Metabolism
DMR16:24868001	16	24868001	1000	1	2.14E-08	-0.5902142	21	2.1	SLC5A11	Transport
DMR16:25003001	16	25003001	1000	1	4.66E-06	-0.9505197	10	1	ARHGAP17	
DMR16:26194001	16	26194001	1000	1	8.96E-06	0.8442602	6	0.6		
DMR16:28089001	16	28089001	1000	1	8.40E-06	-0.5179314	3	0.3	XPO6	
DMR16:28470001	16	28470001	1000	1	2.86E-07	-0.4886337	29	2.9	NPIPB7;CLN3	Transport
DMR16:28549001	16	28549001	2000	1	7.96E-06	-0.5399004	54	2.7	SGF29	
DMR16:28672001	16	28672001	1000	1	2.20E-07	-0.7151577	16	1.6		
DMR16:28758001	16	28758001	4000	1	2.94E-06	-0.4542888	94	2.35	NPIPB9;LOC105379464;PAWRP2	
DMR16:29445001	16	29445001	2000	1	4.41E-12	-0.8894175	23	1.15	SMG1P6;BOLA2-SMG1P6;LOC606724;BOLA2;SLX1B;SLX1B-SULT1A4	
DMR16:29522001	16	29522001	3000	1	9.31E-06	-0.5941022	52	1.733333333		
DMR16:29713001	16	29713001	1000	1	4.83E-22	-2.5806383	4	0.4		
DMR16:29881001	16	29881001	2000	1	5.56E-06	-0.6978015	31	1.55	SEZ6L2	
DMR16:30136001	16	30136001	1000	1	4.98E-06	-0.5850919	28	2.8		
DMR16:30238001	16	30238001	2000	1	3.68E-07	-0.5773517	61	3.05	LOC101929894;NPIPB13	
DMR16:31057001	16	31057001	2000	1	3.70E-08	-0.8314939	44	2.2	LOC101928762;ZNF668	Transcription
DMR16:33058001	16	33058001	2000	1	4.39E-07	-0.7233639	117	5.85	LOC107984809	
DMR16:34451001	16	34451001	6000	1	1.02E-07	-0.5449922	237	3.95		
DMR16:34510001	16	34510001	2000	1	2.01E-06	-0.6267302	12	0.6		
DMR16:34802001	16	34802001	2000	1	3.36E-06	-0.5195174	251	12.55		
DMR16:34891001	16	34891001	5000	1	8.68E-06	-0.4843965	171	3.42		
DMR16:38265001	16	38265001	4000	4	8.26E-10	-0.5258897	68	1.7		
DMR16:38275001	16	38275001	6000	2	1.74E-12	-0.5895495	85	1.416666667		
DMR16:46856001	16	46856001	2000	1	1.52E-06	-0.8703499	35	1.75		
DMR16:46988001	16	46988001	1000	1	1.34E-07	-0.6901877	21	2.1		
DMR16:53927001	16	53927001	1000	1	5.82E-07	0.683032	7	0.7	FTO	Metabolism
DMR16:54883001	16	54883001	1000	1	4.04E-07	-0.8288508	6	0.6	LOC105371275	
DMR16:57070001	16	57070001	2000	1	8.38E-06	-0.4976409	11	0.55	NLRC5	Cytoskeleton
DMR16:57887001	16	57887001	2000	1	1.76E-06	0.6799488	36	1.8	CNGB1	Ion Channel
DMR16:60843001	16	60843001	1000	1	8.84E-09	-1.091394	5	0.5		
DMR16:63897001	16	63897001	1000	1	4.64E-06	0.5861368	6	0.6		
DMR16:64875001	16	64875001	1000	1	1.98E-06	0.7585032	11	1.1		
DMR16:66504001	16	66504001	2000	1	4.84E-07	-0.7521243	45	2.25	BEAN1;TK2	Signaling
DMR16:66789001	16	66789001	2000	1	3.50E-08	-0.6980976	34	1.7	TERB1	
DMR16:67338001	16	67338001	1000	1	3.82E-06	-1.0827862	3	0.3	LRRC36	
DMR16:67491001	16	67491001	2000	1	1.08E-06	-0.9247598	29	1.45	ATP6V0D1;ATP6V0D1-DT;AGRP	Metabolism;Signaling
DMR16:67500001	16	67500001	2000	1	4.60E-07	-0.9597891	30	1.5	ATP6V0D1-DT	
DMR16:68212001	16	68212001	1000	1	2.99E-06	-0.7355857	7	0.7	NFATC3;RPS12P27	Transcription
DMR16:68671001	16	68671001	2000	1	1.82E-07	-0.7358709	30	1.5	CDH3	Cytoskeleton
DMR16:68818001	16	68818001	1000	1	1.76E-06	-0.5147666	35	3.5	CDH1;FTLP14	Cytoskeleton
DMR16:69143001	16	69143001	1000	1	7.19E-06	0.5715039	23	2.3	UTP4	
DMR16:69359001	16	69359001	1000	1	3.35E-06	-0.6940417	20	2	TMED6;TERF2	Transport
DMR16:69427001	16	69427001	1000	1	1.04E-06	-0.9001128	18	1.8	CYB5B	Metabolism
DMR16:69912001	16	69912001	3000	2	1.51E-10	-0.7439521	46	1.533333333	WWP2	Proteolysis
DMR16:70327001	16	70327001	1000	1	3.78E-06	-0.707058	18	1.8	DDX19B;DDX19A-DT	
DMR16:70923001	16	70923001	1000	1	1.21E-08	-0.6805045	17	1.7	HYDIN	
DMR16:71773001	16	71773001	2000	1	1.25E-08	-0.8047827	32	1.6	AP1G1;LOC105371337	Transport
DMR16:71841001	16	71841001	1000	1	7.67E-06	-0.4993845	38	3.8	LOC100420489;ATXN1L	
DMR16:71999001	16	71999001	1000	1	5.33E-06	-0.693731	18	1.8	PKD1L3;RPL39P31;ATP5F1AP3;DHODH	Transport;Metabolism

DMR16:73872001	16	73872001	2000	1	1.00E-06	-0.8435147	23	1.15	ZFHX3	Transcription
DMR16:75596001	16	75596001	1000	1	1.04E-07	-0.7439302	20	2	ADAT1	Translation
DMR16:78635001	16	78635001	1000	1	5.54E-06	0.5298308	21	2.1	WWOX	Metabolism
DMR16:79386001	16	79386001	1000	1	5.73E-06	0.787854	10	1	MAF	Transcription
DMR16:82711001	16	82711001	2000	1	3.77E-06	0.8673394	27	1.35	CDH13	Cytoskeleton
DMR16:83118001	16	83118001	2000	1	3.76E-06	0.5640698	35	1.75	CDH13	Cytoskeleton
DMR16:83907001	16	83907001	1000	1	4.20E-07	0.8914439	21	2.1	MLYCD	
DMR16:84997001	16	84997001	1000	1	1.15E-06	-0.5321896	33	3.3	ZDHHC7	
DMR16:86370001	16	86370001	1000	1	6.36E-07	0.6253344	26	2.6		
DMR16:86932001	16	86932001	1000	1	6.00E-06	-0.4233458	14	1.4	LOC105371393	
DMR16:87034001	16	87034001	3000	1	7.95E-06	0.8345403	57	1.9	LOC105371393	
DMR16:87136001	16	87136001	1000	1	5.44E-06	-0.5541929	15	1.5		
DMR16:87152001	16	87152001	1000	1	3.07E-07	0.5184775	27	2.7		
DMR16:87629001	16	87629001	1000	1	8.08E-06	0.7087552	25	2.5	JPH3	
DMR16:88085001	16	88085001	1000	1	8.53E-06	-0.673064	25	2.5	BANP	
DMR16:88470001	16	88470001	1000	1	4.74E-07	-0.4991618	42	4.2	ZFPM1;MIR5189	Transcription
DMR16:88547001	16	88547001	1000	1	3.38E-07	-0.9329004	31	3.1	ZFPM1;LOC107984905	Transcription
DMR16:88831001	16	88831001	1000	1	6.55E-07	-0.5868393	85	8.5	GALNS;LOC107987238	Metabolism
DMR16:89070001	16	89070001	1000	1	8.28E-07	-0.7351201	40	4	LOC105371411;LOC105371412	
DMR16:89157001	16	89157001	2000	1	2.04E-08	0.8634858	55	2.75	ACSF3;LINC00304;LINC02138	Metabolism
DMR16:89192001	16	89192001	3000	1	2.89E-06	-1.6381348	184	6.133333333	CDH15;SLC22A31;ZNF778	Cytoskeleton;Transport;Transcription
DMR16:89229001	16	89229001	1000	1	1.13E-06	0.6370975	12	1.2	ZNF778	Transcription
DMR16:89304001	16	89304001	4000	2	1.60E-07	-0.5940812	220	5.5	ANKRD11;LOC105371414	
DMR16:89417001	16	89417001	1000	1	1.18E-06	0.8698404	13	1.3	ANKRD11	
DMR16:89568001	16	89568001	4000	1	3.17E-06	-0.5775098	159	3.975	RPL13;SNORD68;CPNE7	Translation
DMR16:90111001	16	90111001	1000	1	7.28E-06	-0.6863451	9	0.9	LOC105376786;FAM157C	
DMR17:113001	17	113001	1000	1	7.06E-06	-0.473938	9	0.9	LOC101929823;LOC101929828	
DMR17:541001	17	541001	1000	1	7.51E-09	0.6423036	33	3.3	VPS53	Transport
DMR17:575001	17	575001	9000	1	4.88E-07	0.4688583	163	1.811111111	VPS53	Transport
DMR17:678001	17	678001	1000	1	3.27E-06	-0.4915843	34	3.4	VPS53	Transport
DMR17:963001	17	963001	2000	1	8.09E-06	-0.637906	46	2.3	NXN	Metabolism
DMR17:1194001	17	1194001	5000	1	3.02E-06	-0.4966464	145	2.9	ABR;LOC105371479	Signaling
DMR17:1275001	17	1275001	1000	1	1.36E-06	-0.5427996	14	1.4	BHLHA9;TRARG1	Transcription
DMR17:1292001	17	1292001	2000	1	4.93E-10	-0.5845206	51	2.55	TRARG1	
DMR17:1357001	17	1357001	2000	1	5.32E-06	-0.5578463	72	3.6	YWHAE	Cytoskeleton
DMR17:1599001	17	1599001	5000	1	3.10E-06	0.6163454	129	2.58	SLC43A2;RN7SL105P	
DMR17:1924001	17	1924001	1000	1	4.43E-07	-0.5720705	24	2.4	RTN4RL1	Receptor
DMR17:2133001	17	2133001	2000	1	3.22E-06	-0.9057236	29	1.45	SMG6;MCUR1P1	Metabolism
DMR17:2230001	17	2230001	1000	1	8.93E-06	-0.6608527	11	1.1	SMG6;LOC101927839	Metabolism
DMR17:3728001	17	3728001	1000	1	9.87E-07	-0.7240017	17	1.7	ITGAE;HASPIN	Extracellular Matrix
DMR17:4029001	17	4029001	1000	1	3.12E-07	-1.1640653	4	0.4	ZZEF1	
DMR17:4824001	17	4824001	9000	2	3.68E-07	-0.687987	217	2.411111111	PLD2;MINK1	Metabolism;Signaling
DMR17:4855001	17	4855001	1000	1	6.07E-06	-0.5658243	28	2.8	MINK1;ATP6V0CP1	Signaling
DMR17:7174001	17	7174001	3000	2	1.26E-07	-0.5624529	61	2.033333333	ASGR1	Transport
DMR17:8217001	17	8217001	1000	1	3.89E-06	-0.5733933	37	3.7	AURKB;LINC00324;TRW-CCA1-1;TRG-TCC3-1;TRD-GTC2-11;TRP-CGG1-3;CTC1;TRT-AGT1-2;TRS-AGA2-6;TRI-AAT4-1	Signaling
DMR17:9429001	17	9429001	3000	1	1.33E-06	-1.0032046	38	1.266666667	STX8	Transcription
DMR17:9630001	17	9630001	2000	1	6.87E-06	-0.4993221	51	2.55	CFAP52	
DMR17:10153001	17	10153001	1000	1	5.52E-06	-0.7529044	12	1.2	GAS7	Cytoskeleton
DMR17:10619001	17	10619001	1000	1	6.23E-06	-0.4517079	36	3.6	MYHAS;MYH3	
DMR17:10759001	17	10759001	1000	1	8.44E-09	-0.686819	33	3.3	TMEM220-AS1	
DMR17:10846001	17	10846001	2000	1	3.80E-06	-0.5612097	21	1.05	PIRT;RNU6-1065P	
DMR17:11937001	17	11937001	1000	1	4.85E-06	0.594339	18	1.8	DNAH9	Cytoskeleton
DMR17:16469001	17	16469001	2000	1	7.38E-07	0.7853438	28	1.4	SNHG29;LRRC75A	
DMR17:17747001	17	17747001	2000	1	5.00E-06	0.7298751	27	1.35	RAI1	Transcription
DMR17:18030001	17	18030001	1000	1	6.21E-06	-0.6141983	24	2.4	ATPAF2;GID4	Transcription
DMR17:18690001	17	18690001	1000	1	3.59E-06	-0.639921	8	0.8	ZNF286B;FOXO3B;TRIM16L	
DMR17:18790001	17	18790001	1000	1	5.43E-06	-0.6372705	16	1.6	TVP23B	
DMR17:19148001	17	19148001	1000	1	6.09E-06	-0.4798411	42	4.2	GRAPL;GRAPL-AS1	Cytoskeleton
DMR17:19183001	17	19183001	2000	1	1.22E-12	-0.8743943	37	1.85	SNORD3A;SNORD3C	
DMR17:19528001	17	19528001	1000	1	1.75E-06	-0.5477435	23	2.3	SLC47A1	Transport
DMR17:20015001	17	20015001	3000	1	4.41E-07	-0.5972522	107	3.566666667	SPECC1	
DMR17:20915001	17	20915001	1000	1	7.73E-06	-0.7622975	15	1.5	CCDC144NL-AS1	
DMR17:21968001	17	21968001	24000	2	6.04E-07	-0.4523794	370	1.541666667		
DMR17:22091001	17	22091001	1000	1	5.90E-06	-0.4812258	19	1.9		

DMR17:22528001	17	22528001	4000	1	5.04E-06	0.3643482	76	1.9	MTND6P35;MTCYBP13;MTRNR2L1;MTND1P15;MTND2P13;MTCO1P13;NMTRS-TGA3-1;MTCO2P13;MTATP6P3;MTCO3P13;MTND4LP8;MTND6P34	
DMR17:25735001	17	25735001	1000	1	1.94E-07	-0.858364	6	0.6		
DMR17:26572001	17	26572001	2000	1	6.64E-07	0.7216134	25	1.25		
DMR17:26773001	17	26773001	4000	1	3.19E-07	-0.4768216	351	8.775		
DMR17:27924001	17	27924001	2000	1	2.89E-06	-0.8929372	38	1.9	LINC01992	
DMR17:28730001	17	28730001	2000	1	2.39E-06	-0.4775851	58	2.9	RPL23A;SNORD42B;SNORD4A;SNORD42A;SNORD4B;TLCD1;NEK8	Translation;Signaling
DMR17:30228001	17	30228001	2000	1	4.20E-06	1.0562173	30	1.5	SLC6A4;LOC107984991;LOC105371720	Transport
DMR17:31545001	17	31545001	1000	1	6.19E-07	-0.6002978	31	3.1	RAB11FIP4;TRT-CGT4-1	
DMR17:31866001	17	31866001	1000	1	1.85E-06	-0.5598215	27	2.7	COPRS;UTP6	Metabolism
DMR17:31925001	17	31925001	2000	1	3.94E-06	-0.4995322	49	2.45		
DMR17:32032001	17	32032001	2000	1	7.19E-06	-0.7842063	24	1.2	LRRC37B;SH3GL1P1	Receptor
DMR17:33928001	17	33928001	2000	1	6.30E-07	0.8677504	25	1.25	ASIC2;LOC107985038	Transport
DMR17:34372001	17	34372001	2000	1	3.16E-08	0.8763582	16	0.8	CCL1	Growth Factors
DMR17:37459001	17	37459001	2000	1	1.30E-06	-0.8868144	22	1.1	TADA2A;LOC100419621	Transcription
DMR17:38262001	17	38262001	3000	1	1.61E-07	-0.5671546	71	2.366666667	NPEPPSP1;LOC105371760	
DMR17:38813001	17	38813001	2000	1	3.88E-06	-0.6251864	56	2.8	CWC25	
DMR17:39276001	17	39276001	1000	1	2.92E-08	-0.6363241	19	1.9	FBXL20	
DMR17:39421001	17	39421001	2000	1	1.69E-09	-0.7266384	50	2.5	MED1	Transcription
DMR17:39437001	17	39437001	2000	1	7.77E-06	-0.5168175	45	2.25	MED1	Transcription
DMR17:39585001	17	39585001	1000	1	4.81E-06	-0.5840436	23	2.3	LOC105371771	
DMR17:40046001	17	40046001	1000	1	5.42E-08	-0.6219634	47	4.7	MED24	Transcription
DMR17:40184001	17	40184001	4000	1	4.92E-06	-0.4965266	82	2.05	RAPGEFL1;MIR6867	Transcription
DMR17:41703001	17	41703001	2000	1	4.38E-07	-0.6112228	44	2.2	GAST	Hormone
DMR17:42123001	17	42123001	1000	1	3.35E-06	-0.4969693	45	4.5	KAT2A;HSPB9;RAB5C	
DMR17:42173001	17	42173001	1000	1	5.79E-06	-0.6650794	14	1.4	KCNH4	Transport
DMR17:42616001	17	42616001	2000	1	7.73E-06	-0.5669061	38	1.9	RETREG3;TUBG1	Cytoskeleton
DMR17:42738001	17	42738001	3000	1	3.07E-07	-0.6941574	55	1.833333333	EZH1	Epigenetic
DMR17:43842001	17	43842001	2000	1	4.20E-08	-0.6351435	80	4	MPP3;LOC107984979;CD300LG;LOC107985077	Cytoskeleton;Immune
DMR17:44079001	17	44079001	2000	1	9.61E-07	0.9534184	39	1.95	G6PC3;HDAC5	Signaling
DMR17:44512001	17	44512001	2000	1	3.15E-09	-0.5645893	68	3.4	GPATCH8	
DMR17:44984001	17	44984001	2000	1	2.43E-09	-0.8014134	99	4.95	LOC112268183;LOC107987243	
DMR17:46319001	17	46319001	2000	1	5.03E-06	-0.7784243	23	1.15	ARL17B;LRRC37A;RN7SL656P	Receptor
DMR17:47863001	17	47863001	2000	1	1.57E-06	-0.6591498	34	1.7	SP6	Transcription
DMR17:48157001	17	48157001	3000	1	7.14E-06	-0.5792749	66	2.2	SKAP1;MIR1203	Cytoskeleton
DMR17:49333001	17	49333001	1000	1	4.25E-06	-0.556171	22	2.2	ZNF652	Transcription
DMR17:49960001	17	49960001	2000	2	6.86E-07	-0.6112813	52	2.6	RNU6-1313P;DLX4	Development
DMR17:50086001	17	50086001	1000	1	5.63E-15	-0.9883369	15	1.5	ITGA3;PDK2	Extracellular Matrix;Signaling
DMR17:50775001	17	50775001	1000	1	5.89E-11	-0.7969115	23	2.3	ANKRD40CL;MIR8059	
DMR17:52179001	17	52179001	1000	1	3.90E-10	-0.8242539	17	1.7		
DMR17:55501001	17	55501001	1000	1	2.02E-11	-1.3085068	21	2.1		
DMR17:55627001	17	55627001	1000	1	7.78E-06	0.863732	13	1.3	LOC101927389	
DMR17:55643001	17	55643001	2000	1	2.37E-06	0.6896392	18	0.9	LOC101927389	
DMR17:57697001	17	57697001	1000	1	4.17E-06	-0.4384789	15	1.5		
DMR17:58592001	17	58592001	1000	1	4.29E-08	-0.6254502	28	2.8	TEX14	
DMR17:58697001	17	58697001	2000	1	2.93E-08	-0.7288188	40	2	TEX14;RAD51C	Transcription
DMR17:58836001	17	58836001	2000	1	1.21E-07	-0.7955828	42	2.1	PPM1E;RNU6-518P	Signaling
DMR17:59209001	17	59209001	1000	1	6.14E-06	-0.7614737	35	3.5	PRR11;SMG8	
DMR17:59246001	17	59246001	1000	1	7.81E-08	-0.7556745	20	2	GDPD1	Metabolism
DMR17:60621001	17	60621001	1000	1	5.37E-09	-0.6682374	28	2.8	PPM1D	
DMR17:61313001	17	61313001	1000	1	3.92E-08	0.9362022	23	2.3	BCAS3	
DMR17:62235001	17	62235001	2000	1	7.58E-06	-0.4186111	77	3.85	LOC100996361	
DMR17:62451001	17	62451001	1000	1	8.86E-07	-0.6249391	24	2.4	METTL2A;LOC112268200	Epigenetic
DMR17:62549001	17	62549001	1000	1	1.01E-06	-0.5869207	21	2.1	TLK2	Signaling
DMR17:63207001	17	63207001	1000	1	5.98E-06	-0.6283573	15	1.5	TANC2	
DMR17:64300001	17	64300001	3000	1	1.81E-08	-0.8697701	61	2.033333333	RPL31P57	
DMR17:64556001	17	64556001	1000	1	6.62E-06	0.6979723	11	1.1	SMURF2	Proteolysis
DMR17:65359001	17	65359001	2000	1	7.98E-06	0.8013968	18	0.9		
DMR17:65431001	17	65431001	1000	1	1.27E-06	0.6746894	16	1.6		
DMR17:65903001	17	65903001	1000	1	2.46E-07	0.8042097	16	1.6	CEP112;LOC105371867	
DMR17:68008001	17	68008001	1000	1	2.15E-06	-0.6129225	29	2.9		
DMR17:69274001	17	69274001	1000	1	6.05E-06	-0.8005959	8	0.8	ABCA5	Transport

DMR17:69449001	17	69449001	1000	1	2.20E-06	-0.6227236	27	2.7	MAP2K6	Signaling
DMR17:73147001	17	73147001	2000	1	2.83E-07	-0.6426642	50	2.5		
DMR17:73357001	17	73357001	1000	1	7.01E-06	0.6916319	25	2.5	SDK2	
DMR17:74991001	17	74991001	1000	1	1.20E-06	-0.5437654	33	3.3	CDR2L	
DMR17:75044001	17	75044001	1000	1	2.38E-07	-0.6499252	34	3.4	KCTD2;TRR-CCT2-1;TRR-TCG3-1;ATP5PD;RN7SL573P	Metabolism
DMR17:75310001	17	75310001	2000	1	3.56E-06	-0.5916727	34	1.7	GRB2	
DMR17:75841001	17	75841001	1000	1	6.42E-06	-0.5320606	29	2.9	UNC13D;WBP2	
DMR17:75966001	17	75966001	3000	1	6.95E-07	-0.4991988	85	2.833333333	ACOX1	Metabolism
DMR17:76704001	17	76704001	1000	1	6.11E-06	-0.7255993	32	3.2	MXRA7;RNY4P36;JMJD6	Golgi
DMR17:77168001	17	77168001	3000	1	1.32E-06	0.7167537	37	1.233333333	SEC14L1	
DMR17:77174001	17	77174001	2000	1	2.34E-08	0.9098936	33	1.65	SEC14L1	
DMR17:77448001	17	77448001	1000	1	3.10E-06	-0.5803546	22	2.2	SEPTIN9;LOC112268198;LOC105371903	Cytoskeleton
DMR17:77930001	17	77930001	1000	1	5.28E-08	-0.8157858	17	1.7	RNU1-80P;LOC105371909	
DMR17:78459001	17	78459001	2000	1	7.97E-06	0.6833206	67	3.35	DNAH17	Cytoskeleton
DMR17:79462001	17	79462001	2000	1	5.34E-08	-0.6022006	64	3.2	RBFOX3	Translation
DMR17:79699001	17	79699001	1000	1	4.18E-06	-0.5378748	21	2.1	MIR4739;LINC02078	
DMR17:80511001	17	80511001	1000	1	1.59E-07	-0.4318812	13	1.3		
DMR17:80574001	17	80574001	1000	1	7.93E-07	-0.5366309	33	3.3	RPTOR;LOC105371922	
DMR17:80748001	17	80748001	2000	2	2.91E-09	-0.5634943	84	4.2	RPTOR	
DMR17:81424001	17	81424001	3000	1	1.31E-09	-0.5295235	29	0.966666667	BAHCC1	Transcription
DMR17:81587001	17	81587001	3000	1	2.83E-06	-0.5404155	76	2.533333333	NPLOC4	
DMR17:81611001	17	81611001	1000	1	4.61E-08	-0.645703	27	2.7	NPLOC4	
DMR17:81742001	17	81742001	1000	1	8.15E-06	-0.5022729	9	0.9	LOC105371929	
DMR17:81988001	17	81988001	1000	1	4.41E-06	-0.5777154	25	2.5	ASPSCR1	
DMR17:82521001	17	82521001	1000	1	4.47E-06	-0.5819983	42	4.2	FOXK2	
DMR17:83172001	17	83172001	2000	1	4.66E-06	-0.5503647	73	3.65	LOC101929650	
DMR18:146001	18	146001	2000	1	7.51E-06	-0.5203521	54	2.7	LOC105371951	
DMR18:1720001	18	1720001	2000	1	2.41E-06	-1.1738825	22	1.1		
DMR18:2189001	18	2189001	1000	1	6.80E-06	-0.6998838	13	1.3		
DMR18:3674001	18	3674001	1000	1	1.36E-06	-0.5278526	10	1	DLGAP1;LOC107985137	Cytoskeleton
DMR18:4798001	18	4798001	1000	1	3.06E-06	0.5682879	8	0.8		
DMR18:7241001	18	7241001	2000	1	5.78E-07	-0.7367132	32	1.6	LRRC30;LOC105371973	Cytoskeleton
DMR18:7522001	18	7522001	1000	1	1.06E-09	-0.6178372	44	4.4		
DMR18:8611001	18	8611001	2000	1	2.12E-07	1.0417117	30	1.5	RAB12	
DMR18:9767001	18	9767001	1000	1	2.83E-07	0.843816	10	1	RAB31	
DMR18:10149001	18	10149001	1000	1	1.44E-06	0.6338314	15	1.5		
DMR18:10506001	18	10506001	1000	1	6.63E-06	0.7804361	53	5.3		
DMR18:10682001	18	10682001	1000	1	1.43E-06	0.9915159	18	1.8	PIEZO2	
DMR18:11654001	18	11654001	1000	1	2.35E-07	0.6856763	23	2.3	MIR7153	
DMR18:12612001	18	12612001	1000	1	7.92E-06	0.5574022	9	0.9	SPIRE1	Cytoskeleton
DMR18:12624001	18	12624001	1000	1	2.95E-06	-0.7114766	20	2	SPIRE1	Cytoskeleton
DMR18:14640001	18	14640001	2000	1	4.75E-06	-0.416908	40	2	LOC105372004;SNX19P3;GTF2IP8	
DMR18:20561001	18	20561001	2000	2	3.79E-10	-0.5953821	29	1.45		
DMR18:20921001	18	20921001	2000	1	6.17E-07	0.6585293	30	1.5		
DMR18:20933001	18	20933001	1000	1	5.67E-06	0.65989	21	2.1		
DMR18:22307001	18	22307001	1000	1	7.14E-09	0.9298139	8	0.8	LOC101927548	
DMR18:25312001	18	25312001	1000	1	4.61E-06	-0.5848284	27	2.7	ZNF521	Transcription
DMR18:28472001	18	28472001	1000	1	1.88E-08	0.8484998	11	1.1		
DMR18:30490001	18	30490001	3000	1	1.49E-29	-3.0065184	8	0.266666667		
DMR18:32376001	18	32376001	1000	1	3.89E-06	-0.8649771	12	1.2	GAREM1	
DMR18:35116001	18	35116001	2000	1	9.96E-07	0.7487236	23	1.15	MAPRE2	Cytoskeleton
DMR18:37654001	18	37654001	1000	1	1.90E-06	-0.8933564	8	0.8	MIR4318	
DMR18:43144001	18	43144001	1000	1	2.66E-06	0.7522161	10	1		
DMR18:44652001	18	44652001	1000	1	4.62E-06	0.823461	9	0.9	LOC105372089	
DMR18:46349001	18	46349001	2000	1	6.31E-08	0.7738177	30	1.5	RNF165	Proteolysis
DMR18:47850001	18	47850001	1000	1	8.77E-23	-2.5359017	3	0.3	SMAD2;MTCO2P2	Transcription
DMR18:48601001	18	48601001	1000	1	3.10E-06	1.0039766	12	1.2	CTIF	Metabolism
DMR18:49759001	18	49759001	4000	1	1.72E-06	-0.6220821	102	2.55		
DMR18:49958001	18	49958001	1000	1	3.44E-07	0.7765349	11	1.1	MYO5B	Cytoskeleton
DMR18:52370001	18	52370001	1000	1	9.10E-09	-1.2096767	7	0.7	DCC	
DMR18:56470001	18	56470001	1000	1	2.53E-06	-0.4684875	4	0.4		
DMR18:57326001	18	57326001	2000	1	1.03E-06	-0.627242	52	2.6		
DMR18:60605001	18	60605001	2000	1	4.62E-06	0.7236615	13	0.65		
DMR18:61970001	18	61970001	1000	1	1.05E-65	-5.7314429	0	0	LOC105372158	
DMR18:62435001	18	62435001	1000	1	2.17E-07	1.1289915	12	1.2	ACTBP9	
DMR18:63209001	18	63209001	1000	1	5.37E-06	0.5041076	13	1.3	BCL2	

DMR18:63722001	18	63722001	1000	1	4.31E-07	0.879428	8	0.8	SERPINB11	Protease; Proteolysis
DMR18:63937001	18	63937001	1000	1	2.36E-06	0.4894029	19	1.9	SERPINB10	Protease; Proteolysis
DMR18:67075001	18	67075001	1000	1	4.82E-07	-0.7465334	5	0.5	MIR5011	
DMR18:71931001	18	71931001	1000	1	8.87E-06	-0.5256539	5	0.5		
DMR18:72358001	18	72358001	1000	1	2.82E-06	0.7193982	5	0.5		
DMR18:75165001	18	75165001	1000	1	1.21E-06	1.1513774	3	0.3	LOC105376874	
DMR18:76120001	18	76120001	1000	1	2.52E-06	-0.7472806	10	1	LOC339298	
DMR18:76401001	18	76401001	1000	1	1.35E-08	-0.5607552	34	3.4	ZNF516	Transcription
DMR18:76423001	18	76423001	2000	1	5.11E-06	0.4067854	70	3.5	ZNF516	Transcription
DMR18:76432001	18	76432001	1000	1	2.63E-06	0.5848653	22	2.2	ZNF516	Transcription
DMR18:76925001	18	76925001	3000	1	9.47E-07	0.4893093	50	1.666666667	ZNF236	Transcription
DMR18:77373001	18	77373001	1000	1	4.34E-07	-0.7494154	22	2.2		
DMR18:77992001	18	77992001	2000	1	1.96E-08	0.6860294	47	2.35	LINC01029;LOC105372218	
DMR18:78172001	18	78172001	1000	1	1.63E-07	0.7410762	14	1.4		
DMR18:78702001	18	78702001	5000	1	9.72E-07	-0.444002	120	2.4		
DMR18:78851001	18	78851001	1000	1	4.66E-07	0.5727155	16	1.6		
DMR18:79038001	18	79038001	3000	1	1.39E-12	0.7520149	51	1.7	LOC105372225	
DMR18:79339001	18	79339001	1000	1	4.64E-06	0.5847893	22	2.2	ATP9B;LOC107985149	Transport
DMR18:79524001	18	79524001	2000	1	6.05E-06	-0.5772977	124	6.2	NFATC1;LOC102723506	Transcription
DMR18:79623001	18	79623001	1000	1	2.24E-08	0.7051726	18	1.8	LOC105372228	
DMR18:79892001	18	79892001	4000	1	5.58E-07	0.504038	57	1.425	KCNG2;SLC66A2	Transport
DMR18:79952001	18	79952001	1000	1	3.51E-06	-0.8581175	98	9.8	SLC66A2	
DMR19:103001	19	103001	1000	1	5.05E-06	0.7535465	14	1.4	OR4G3P;OR4G1P;OR4F17	Receptor
DMR19:183001	19	183001	1000	1	1.43E-08	-0.9271632	19	1.9	SEPTIN14P19;CICP19	
DMR19:269001	19	269001	2000	1	1.48E-06	0.6155663	28	1.4	VN2R11P	
DMR19:428001	19	428001	6000	1	2.22E-06	0.7668201	313	5.216666667	SHC2	Cytoskeleton
DMR19:857001	19	857001	1000	1	2.04E-08	-0.6071836	35	3.5	PRTN3;ELANE;CFD;MED16	Protease
DMR19:871001	19	871001	1000	1	1.11E-09	-0.9644363	45	4.5	CFD;MED16	Protease
DMR19:1137001	19	1137001	2000	1	6.66E-06	-0.6370321	100	5	SBNO2	
DMR19:1193001	19	1193001	5000	1	5.32E-06	-0.5183393	206	4.12	HMGB2P1;STK11	
DMR19:1532001	19	1532001	7000	2	8.65E-07	-0.547405	218	3.114285714	LOC107985337;PLK5	Signaling
DMR19:1570001	19	1570001	1000	1	7.06E-06	-0.4830692	15	1.5	MEX3D;RN7SL477P;MBD3	Metabolism
DMR19:1899001	19	1899001	2000	1	9.91E-07	-0.4824232	66	3.3	SCAMP4;ADAT3	Transport;Metabolism
DMR19:2024001	19	2024001	3000	1	1.70E-06	-0.4890286	60	2	BTBD2;LOC107985278	Proteolysis
DMR19:2104001	19	2104001	1000	1	7.82E-06	-0.3798831	30	3	MOB3A;IZUMO4;AP3D1	Signaling;Transport
DMR19:2200001	19	2200001	2000	1	2.12E-06	-0.5461934	104	5.2	DOT1L	Epigenetic
DMR19:2604001	19	2604001	1000	1	9.25E-07	-0.5323897	28	2.8	GNG7;RN7SL121P	Signaling
DMR19:2802001	19	2802001	2000	1	9.14E-08	-0.5218096	80	4	THOP1	Protease
DMR19:3063001	19	3063001	2000	1	3.29E-06	-0.6318946	44	2.2	TLE5;LOC105372242	Transcription
DMR19:3528001	19	3528001	3000	1	4.56E-06	-0.4956112	95	3.166666667	FZR1;MFSD12;C19orf71	Proteolysis
DMR19:4653001	19	4653001	1000	1	2.79E-07	-0.5395317	32	3.2	TNFAIP8L1;MYDGF	Growth Factors
DMR19:4783001	19	4783001	2000	1	2.07E-07	-0.9198093	52	2.6	FEM1A	
DMR19:4898001	19	4898001	4000	1	3.07E-06	-0.4881431	107	2.675	ARRDC5;UHRF1	Proteolysis
DMR19:5019001	19	5019001	2000	1	2.65E-06	-0.4146911	48	2.4	KDM4B	Epigenetic
DMR19:5715001	19	5715001	1000	1	3.20E-06	-0.6113556	28	2.8	LONP1;CATSPERD	Protease
DMR19:5787001	19	5787001	1000	1	1.54E-08	-0.5327437	56	5.6	CATSPERD;PRR22;DUS3L	Translation
DMR19:6448001	19	6448001	1000	1	5.09E-08	-0.7577381	18	1.8	SLC25A23	Transport
DMR19:6477001	19	6477001	2000	1	1.93E-06	-0.9434586	81	4.05	CRB3;DENND1C	
DMR19:6497001	19	6497001	1000	1	2.16E-06	-0.7545024	23	2.3	TUBB4A	Cytoskeleton
DMR19:6532001	19	6532001	2000	1	2.70E-06	-0.4693562	42	2.1	TNFSF9	
DMR19:7053001	19	7053001	1000	1	2.60E-17	-1.8784816	4	0.4	MBD3L2;MBD3L3	
DMR19:7197001	19	7197001	1000	1	1.54E-06	-0.8207822	38	3.8	INSR	Receptor
DMR19:8032001	19	8032001	2000	1	2.70E-06	-0.7275464	32	1.6		
DMR19:8057001	19	8057001	2000	1	2.80E-06	-0.9269528	32	1.6	CCL25;FBN3	Growth Factors;Extracellular Matrix
DMR19:8099001	19	8099001	1000	1	1.39E-09	-0.6992111	26	2.6	FBN3	Extracellular Matrix
DMR19:8161001	19	8161001	2000	1	5.73E-06	-0.612304	48	2.4		
DMR19:8361001	19	8361001	1000	1	3.10E-06	-0.5576764	25	2.5	ANGPTL4	Signaling
DMR19:8419001	19	8419001	3000	1	1.19E-07	-0.5600499	80	2.666666667	MARCHF2	Proteolysis
DMR19:8915001	19	8915001	3000	1	6.22E-06	0.8766411	27	0.9	MUC16	
DMR19:9407001	19	9407001	2000	1	1.21E-06	0.8189852	44	2.2	LOC112268250;ZNF266	Transcription
DMR19:10480001	19	10480001	2000	1	2.74E-06	-0.5514047	82	4.1	KEAP1	Cytoskeleton
DMR19:10618001	19	10618001	2000	1	2.95E-06	-0.5041841	64	3.2	SLC44A2	Transport
DMR19:10709001	19	10709001	1000	1	6.18E-06	-0.4410358	28	2.8	QTRT1;DNM2;MIR638	Translation;Transport
DMR19:10807001	19	10807001	1000	1	5.79E-06	-0.5723688	29	2.9	DNM2;MIR199A1	Transport
DMR19:10937001	19	10937001	1000	1	1.04E-07	-0.7195088	22	2.2	YIPF2;TIMM29	
DMR19:10940001	19	10940001	2000	1	2.69E-06	-0.8493338	40	2	TIMM29	
DMR19:11255001	19	11255001	2000	1	1.74E-06	-0.5258821	48	2.4	DOCK6	Transcription

DMR19:11272001	19	11272001	1000	1	8.57E-08	-0.6880779	25	2.5	DOCK6	Transcription
DMR19:11427001	19	11427001	1000	1	6.94E-09	-0.63576	36	3.6	RGL3;ODAD3;PRKCSH	Transcription;Signaling
DMR19:11560001	19	11560001	2000	1	7.66E-06	-0.6889455	43	2.15	CNN1;ELOF1	Cytoskeleton
DMR19:12601001	19	12601001	2000	1	4.98E-06	-0.9090136	45	2.25	ZNF490;VN2R16P;ZNF791	Transcription
DMR19:12780001	19	12780001	2000	1	2.01E-10	-0.6519843	84	4.2	HOOK2;MIR5684;JUNB	Transport;Transcription
DMR19:12869001	19	12869001	3000	1	1.94E-06	-0.5633231	87	2.9	MAST1;DNASE2	Signaling;Transcription
DMR19:12987001	19	12987001	4000	1	4.13E-06	-0.4388419	87	2.175	NFIX	Transcription
DMR19:13234001	19	13234001	1000	1	5.80E-06	-0.6752452	38	3.8	CACNA1A	Transport
DMR19:13910001	19	13910001	2000	1	7.92E-06	-0.5379399	51	2.55	BRME1;CC2D1A	
DMR19:13936001	19	13936001	2000	2	4.02E-06	-0.5369383	69	3.45	CC2D1A;PODNL1	
DMR19:14041001	19	14041001	5000	1	9.00E-06	-0.771333	112	2.24	RLN3;IL27RA;PALM3	Receptor
DMR19:14269001	19	14269001	2000	1	7.44E-06	-0.5846545	39	1.95		
DMR19:14313001	19	14313001	2000	1	3.47E-06	-0.358844	43	2.15	LINC01841	
DMR19:14376001	19	14376001	2000	1	1.05E-16	-1.03228	55	2.75	ADGRE5	Signaling
DMR19:14385001	19	14385001	3000	1	6.33E-06	-0.6106195	73	2.433333333	ADGRE5	Signaling
DMR19:15895001	19	15895001	2000	1	4.42E-08	0.6541477	15	0.75	CYP4F2;LOC89844;LOC100421620	Metabolism
DMR19:16181001	19	16181001	2000	1	8.57E-06	-0.6630486	58	2.9	CIB3;FAM32A	
DMR19:16412001	19	16412001	2000	1	9.62E-09	0.8991661	39	1.95	EPS15L1;RPS2P51	Transport
DMR19:16565001	19	16565001	1000	1	1.79E-06	-0.5606019	25	2.5	SLC35E1;LOC105372295;MED26	Transport;Transcription
DMR19:16828001	19	16828001	1000	1	2.58E-06	-0.4373899	37	3.7	SIN3B	Epigenetic
DMR19:17221001	19	17221001	2000	1	2.34E-07	-0.7405684	41	2.05	MYO9B;USE1;OCEL1;NR2F6	Transcription;Transcription
DMR19:17261001	19	17261001	2000	1	7.22E-06	-0.5213908	52	2.6	USHBP1;BABAM1	
DMR19:17372001	19	17372001	1000	1	2.52E-08	-0.7192815	28	2.8	PLVAP;LOC105372298	
DMR19:17669001	19	17669001	2000	1	1.26E-06	-0.6067661	39	1.95	UNC13A	
DMR19:17785001	19	17785001	1000	1	3.58E-08	-0.6928168	25	2.5	FCHO1;B3GNT3	Cytoskeleton;Golgi
DMR19:18524001	19	18524001	2000	1	2.60E-07	-0.7561527	55	2.75	ELL;FKBP8	Transcription;Transcription
DMR19:19422001	19	19422001	1000	1	2.36E-06	-0.6178075	19	1.9	GATAD2A	Transcription
DMR19:19947001	19	19947001	4000	1	3.50E-06	0.5100318	38	0.95	BNIP3P12	
DMR19:21076001	19	21076001	1000	1	2.62E-06	-0.7895708	14	1.4	ZNF714	Transcription
DMR19:21331001	19	21331001	1000	1	2.10E-08	-0.6813968	32	3.2	ZNF708	Transcription
DMR19:21829001	19	21829001	1000	1	4.45E-06	-0.7175814	12	1.2	ZNF43;BNIP3P27	Transcription
DMR19:23180001	19	23180001	3000	1	1.55E-07	0.8528692	46	1.533333333		
DMR19:23200001	19	23200001	4000	1	6.98E-06	-0.6915116	106	2.65		
DMR19:23265001	19	23265001	1000	1	2.70E-06	-0.7195073	21	2.1	IPO5P1	
DMR19:23274001	19	23274001	2000	1	2.56E-07	-0.5929792	57	2.85	IPO5P1	
DMR19:23346001	19	23346001	2000	1	3.86E-06	0.730996	23	1.15	ZNF91	Transcription
DMR19:23447001	19	23447001	1000	1	3.92E-06	-0.6880267	22	2.2	VN1R92P	
DMR19:24801001	19	24801001	2000	1	5.43E-06	1.0168145	33	1.65		
DMR19:26281001	19	26281001	1000	1	7.63E-07	-0.8431255	22	2.2		
DMR19:26656001	19	26656001	1000	1	8.45E-09	-1.2120773	18	1.8		
DMR19:29128001	19	29128001	2000	1	4.24E-07	0.5942717	44	2.2		
DMR19:30083001	19	30083001	1000	1	1.79E-06	0.8791339	27	2.7	LOC107985271	
DMR19:31567001	19	31567001	2000	1	6.97E-06	0.8621069	27	1.35		
DMR19:31774001	19	31774001	1000	1	5.48E-07	0.7684319	12	1.2		
DMR19:32025001	19	32025001	2000	1	4.87E-07	0.6003722	34	1.7	LINC01837;LINC01533	
DMR19:33650001	19	33650001	1000	1	2.28E-06	-0.853627	8	0.8	CHST8	Transport
DMR19:34187001	19	34187001	1000	1	2.02E-06	-1.0828513	12	1.2	LSM14A	Metabolism
DMR19:34232001	19	34232001	1000	1	7.63E-08	-0.9320247	18	1.8	LSM14A	Metabolism
DMR19:34819001	19	34819001	3000	1	3.32E-06	0.5806083	25	0.833333333	LINC01801;LOC100419842	
DMR19:35455001	19	35455001	1000	1	4.89E-07	-0.7721082	24	2.4	FFAR2	
DMR19:36666001	19	36666001	2000	1	2.98E-06	-0.6530248	112	5.6	ZNF461;ZNF567	Transcription
DMR19:37621001	19	37621001	1000	1	3.14E-06	-0.7899975	12	1.2	ZNF540;ZFP30	
DMR19:37629001	19	37629001	2000	1	5.80E-06	-0.4325192	61	3.05	ZFP30	
DMR19:38456001	19	38456001	1000	1	4.22E-06	-0.6020612	28	2.8	RYR1	Ion Channel
DMR19:38916001	19	38916001	1000	1	1.83E-06	-0.4561622	32	3.2	NFKBIB;CCER2;SARS2	Translation
DMR19:39968001	19	39968001	1000	1	6.30E-06	0.6015056	19	1.9	PSMC4	Protease
DMR19:40450001	19	40450001	1000	1	1.27E-07	-0.6144186	27	2.7	SERTAD3;SERTAD3-AS1;BLVRB	Metabolism
DMR19:40544001	19	40544001	3000	1	1.95E-06	-0.8043749	45	1.5	SPTBN4	
DMR19:40837001	19	40837001	1000	1	1.47E-07	-0.773386	25	2.5	CYP2A6	Metabolism
DMR19:41786001	19	41786001	1000	1	8.03E-06	0.7456547	30	3	CEACAM3	
DMR19:41896001	19	41896001	3000	1	8.99E-06	-0.5614372	69	2.3	ARHGEF1;ERFL	Transcription;Transcription
DMR19:41963001	19	41963001	3000	1	1.86E-06	-0.5960569	79	2.633333333	RABAC1;ATP1A3	Transport;Transport
DMR19:44407001	19	44407001	2000	1	1.08E-06	0.7382802	29	1.45	ZNF285	
DMR19:44915001	19	44915001	2000	1	2.24E-06	-0.569196	60	3	APOE;APOC1;APOC1P1	Binding Proteins
DMR19:45398001	19	45398001	4000	1	3.65E-07	-0.6059407	151	3.775	PPP1R13L;POLR1G;ERCC1	Transcription;Transcription
DMR19:45580001	19	45580001	1000	1	1.29E-06	-0.6121966	29	2.9	OPA3;GPR4	Signaling
DMR19:45807001	19	45807001	3000	1	2.53E-06	-0.5873335	77	2.566666667	RSPH6A;SYMPK	Cytoskeleton
DMR19:46361001	19	46361001	3000	1	4.97E-06	-0.6226715	67	2.233333333	PPP5C	Signaling

DMR19:46751001	19	46751001	3000	1	5.23E-06	-0.6202931	62	2.066666667	STRN4;FKRP	
DMR19:46870001	19	46870001	1000	1	1.23E-07	-0.6757079	30	3	ARHGAP35	Signaling
DMR19:47114001	19	47114001	2000	1	2.36E-06	-0.6007953	80	4	ZC3H4	Transcription
DMR19:47368001	19	47368001	1000	1	8.68E-06	-0.6363795	17	1.7	DHX34	Transcription
DMR19:47391001	19	47391001	2000	1	6.95E-06	0.5288943	43	2.15	DHX34	Transcription
DMR19:47837001	19	47837001	2000	1	9.04E-09	0.6564325	24	1.2	CRX	Development
DMR19:48011001	19	48011001	1000	1	1.32E-09	0.7044403	10	1	ELSPBP1	
DMR19:48327001	19	48327001	2000	1	9.66E-06	-0.8280928	35	1.75	ODAD1;EMP3;TMEM143	Cytoskeleton
DMR19:49201001	19	49201001	1000	1	1.03E-07	0.6477024	21	2.1	TRPM4	Transport
DMR19:49507001	19	49507001	1000	1	2.57E-07	-0.621283	33	3.3	RPS11;SNORD35B;MIR150;COX6CP7;FCGRT	Translation;Immune
DMR19:49701001	19	49701001	2000	1	3.58E-06	-0.8337416	63	3.15	CPT1C	Metabolism
DMR19:50846001	19	50846001	2000	1	5.13E-06	-0.8777498	27	1.35	LOC105372441;KLK3	Protease
DMR19:51347001	19	51347001	1000	1	3.39E-08	0.7284294	20	2	VSIG10L;ETFB	Metabolism
DMR19:51447001	19	51447001	2000	1	2.74E-06	0.6697316	39	1.95	SIGLEC8	Immune
DMR19:52372001	19	52372001	2000	1	4.95E-06	-0.5434543	48	2.4	ZNF610;ZNF880	
DMR19:52627001	19	52627001	1000	1	1.27E-06	0.6517495	10	1	ZNF701;ZNF83	Transcription
DMR19:52960001	19	52960001	1000	1	3.90E-06	0.7466301	27	2.7	ZNF816-ZNF321P;ZNF816;ZNF702P	
DMR19:53299001	19	53299001	2000	1	7.01E-06	0.7509365	42	2.1	BIRC8;FAM90A28P;LOC107987270	Protease; Proteolysis
DMR19:54045001	19	54045001	2000	1	2.74E-12	0.8654101	18	0.9	VSTM1	Immune
DMR19:54195001	19	54195001	1000	1	4.45E-06	-0.7647978	9	0.9	MBOAT7;TSEN34;RPS9	Metabolism;Translation;Translation
DMR19:55002001	19	55002001	1000	1	1.95E-06	-0.7389854	19	1.9	NLRP2;GP6-AS1	
DMR19:55190001	19	55190001	1000	1	1.56E-06	-0.8581913	17	1.7	SYT5;PTPRH	Transport;Signaling
DMR19:55447001	19	55447001	3000	1	1.17E-07	-0.5704546	64	2.133333333	SHISA7;ISOC2	Metabolism
DMR19:55860001	19	55860001	4000	1	3.13E-07	0.8236799	79	1.975	NLRP4	
DMR19:56256001	19	56256001	3000	1	4.62E-07	0.7173518	57	1.9	ZSCAN5A;ZSCAN5DP	Transcription
DMR19:56698001	19	56698001	1000	1	3.77E-07	-0.8286666	16	1.6	LOC105372473;LOC105372472	
DMR19:56720001	19	56720001	1000	1	5.54E-07	-0.6595886	9	0.9	LOC105372473;LOC105372472	
DMR19:57927001	19	57927001	2000	1	3.89E-06	-0.6723596	31	1.55	ZNF418	
DMR19:58033001	19	58033001	1000	1	7.08E-06	-0.6609188	67	6.7	VN2R19P;ZSCAN1	Transcription
DMR19:58299001	19	58299001	1000	1	2.10E-07	-0.4860354	39	3.9	ZNF8;ZNF8-ERVK3-1;ERVK3-1	
DMR19:58376001	19	58376001	1000	1	1.90E-06	-0.6293885	22	2.2	ZNF497-AS1;ZNF837;LOC105372484;MIR4754	
DMR19:58521001	19	58521001	1000	1	1.16E-07	-0.6275853	33	3.3	SLC27A5;LOC105372485;ZBTB45;LOC105372486	Transport;Transcription
DMR20:452001	20	452001	2000	1	2.26E-11	-0.7208139	60	3	TBC1D20	Signaling
DMR20:595001	20	595001	1000	1	1.66E-06	-0.7047371	23	2.3	TCF15	Transcription
DMR20:897001	20	897001	1000	1	1.95E-07	0.5903861	14	1.4	ANGPT4	Signaling
DMR20:1990001	20	1990001	1000	1	5.18E-06	0.5552599	20	2	PDYN-AS1;PDYN	Signaling
DMR20:2822001	20	2822001	2000	1	3.19E-09	-1.17425	95	4.75	C20orf141;TMEM239	
DMR20:3809001	20	3809001	1000	1	2.85E-09	-1.0024248	23	2.3	CDC25B;LINC01730	Signaling
DMR20:3841001	20	3841001	1000	1	9.42E-07	-0.6751771	23	2.3	MAVS	
DMR20:3853001	20	3853001	2000	1	2.39E-06	-0.5722809	67	3.35	MAVS	
DMR20:3877001	20	3877001	1000	1	7.55E-11	-0.6383114	33	3.3	MAVS	
DMR20:4573001	20	4573001	1000	1	6.82E-09	-0.7909294	12	1.2	LOC105372510	
DMR20:5923001	20	5923001	1000	1	2.05E-06	0.7711375	14	1.4	CHGB;KANK1P1	
DMR20:10989001	20	10989001	1000	1	1.11E-06	0.9202166	1	0.1		
DMR20:13680001	20	13680001	1000	1	5.87E-06	-0.5292371	17	1.7		
DMR20:13888001	20	13888001	1000	1	2.52E-07	-0.8096154	12	1.2	SEL1L2	
DMR20:20072001	20	20072001	1000	1	5.23E-09	-1.1480329	12	1.2	CFAP61	Development
DMR20:22038001	20	22038001	1000	1	2.54E-08	0.7697262	11	1.1		
DMR20:22198001	20	22198001	1000	1	1.30E-06	0.8847625	6	0.6		
DMR20:23980001	20	23980001	1000	1	9.95E-07	-0.4755045	18	1.8	GGTLC1;POM121L3P	Metabolism
DMR20:25308001	20	25308001	1000	1	4.29E-07	0.9123908	24	2.4	PYGB;ABHD12	Golgi;Protease
DMR20:28494001	20	28494001	5000	4	9.85E-10	-0.6063222	61	1.22		
DMR20:31207001	20	31207001	8000	1	8.94E-06	-0.3789195	183	2.2875		
DMR20:31224001	20	31224001	7000	1	4.80E-06	-0.3735031	166	2.371428571		
DMR20:31492001	20	31492001	1000	1	9.34E-06	-0.4855222	25	2.5	REM1;LINC00028;TRS-AGA7-1	
DMR20:31613001	20	31613001	3000	1	3.33E-07	-0.6201792	86	2.866666667	LOC105372588;ID1;MIR3193	Transcription
DMR20:32812001	20	32812001	3000	1	1.84E-07	-0.6275801	66	2.2	DNMT3B;MAPRE1	Epigenetic;Cytoskeleton
DMR20:33472001	20	33472001	1000	1	2.19E-06	-0.6686958	26	2.6		
DMR20:33733001	20	33733001	1000	1	2.20E-06	-0.7616159	39	3.9	ZNF341	Transcription
DMR20:33764001	20	33764001	1000	1	1.20E-07	-0.7066078	19	1.9	ZNF341	Transcription
DMR20:34134001	20	34134001	2000	1	3.91E-06	0.7963977	27	1.35		
DMR20:34268001	20	34268001	2000	1	2.81E-07	-0.9985759	97	4.85	ASIP;AHCY	Signaling;Metabolism
DMR20:34764001	20	34764001	1000	1	3.69E-10	-0.7550918	24	2.4	NCOA6	Epigenetic
DMR20:35325001	20	35325001	2000	1	4.39E-06	0.6325941	19	0.95	UQCC1	Transcription

DMR20:35762001	20	35762001	3000	1	1.17E-06	-0.8609362	53	1.766666667	RPF2P1;PHF20	
DMR20:36382001	20	36382001	1000	1	3.80E-06	-0.8803442	9	0.9	DLGAP4	Cytoskeleton
DMR20:36953001	20	36953001	2000	1	1.04E-06	-0.6895484	33	1.65	SAMHD1	Metabolism
DMR20:37023001	20	37023001	1000	1	5.00E-06	-0.7119271	11	1.1	RBL1;RPS3AP3	Epigenetic
DMR20:37038001	20	37038001	1000	1	7.89E-06	-0.660864	15	1.5	RBL1	Epigenetic
DMR20:38124001	20	38124001	1000	1	4.44E-10	-1.5511781	7	0.7	TGM2	Transport
DMR20:38596001	20	38596001	1000	1	4.25E-07	0.5979904	22	2.2	ADIG;ARHGAP40	Signaling
DMR20:38694001	20	38694001	1000	1	3.68E-06	0.6168628	9	0.9		
DMR20:38974001	20	38974001	1000	1	1.14E-06	0.6511032	8	0.8	DHX35;NPM1P19	Transcription
DMR20:39265001	20	39265001	1000	1	6.78E-07	0.6164066	5	0.5		
DMR20:40174001	20	40174001	1000	1	5.49E-06	0.6391239	10	1		
DMR20:41368001	20	41368001	1000	1	6.78E-06	-0.4760148	31	3.1	LPIN3;EMILIN3	
DMR20:42936001	20	42936001	1000	1	3.44E-06	0.7692659	9	0.9	PTPRT	Signaling
DMR20:43490001	20	43490001	1000	1	4.53E-09	-0.8299449	8	0.8		
DMR20:44132001	20	44132001	1000	1	7.94E-06	-0.7042956	13	1.3	JPH2	
DMR20:44323001	20	44323001	2000	1	7.34E-06	-0.5572713	25	1.25		
DMR20:45829001	20	45829001	1000	1	1.28E-06	-0.7481507	21	2.1	TNNC2;SNX21	Cytoskeleton;Cytoskeleton
DMR20:46788001	20	46788001	2000	1	6.60E-07	-0.4274371	42	2.1		
DMR20:47381001	20	47381001	1000	1	5.61E-06	-0.5368327	21	2.1	LINC01754	
DMR20:47394001	20	47394001	1000	1	5.90E-06	-0.7846462	13	1.3	LINC01754	
DMR20:48393001	20	48393001	2000	1	6.42E-07	0.6446047	35	1.75	LOC105372643	
DMR20:49065001	20	49065001	1000	1	4.57E-06	-0.6909616	10	1	CSE1L	Transport
DMR20:49195001	20	49195001	2000	1	8.13E-07	-0.6550294	71	3.55	STAU1	
DMR20:50415001	20	50415001	2000	1	4.03E-06	-0.7176424	44	2.2		
DMR20:50702001	20	50702001	1000	1	4.06E-06	-0.650262	15	1.5	RIPOR3	Signaling
DMR20:50773001	20	50773001	1000	1	5.70E-06	-0.6096376	22	2.2		
DMR20:51031001	20	51031001	2000	2	1.45E-08	0.7151284	25	1.25	KCNG1	Transport
DMR20:54148001	20	54148001	1000	1	5.45E-06	-0.6192031	12	1.2	CYP24A1	Metabolism
DMR20:57008001	20	57008001	1000	1	2.77E-06	-0.8133471	11	1.1		
DMR20:57760001	20	57760001	1000	1	1.16E-06	0.9012279	7	0.7		
DMR20:58427001	20	58427001	2000	1	8.67E-10	0.6364695	26	1.3	VAPB	Transport
DMR20:58465001	20	58465001	1000	1	3.19E-06	0.9674036	19	1.9	APCDD1L	
DMR20:59767001	20	59767001	1000	1	7.25E-07	-0.4506779	5	0.5	PHACTR3	Signaling
DMR20:61013001	20	61013001	1000	1	2.12E-07	-0.9325025	6	0.6		
DMR20:61051001	20	61051001	1000	1	2.45E-08	1.0145786	24	2.4		
DMR20:61980001	20	61980001	3000	2	4.11E-06	-0.4159319	99	3.3	TAF4	Transcription
DMR20:62054001	20	62054001	1000	1	2.46E-06	-0.4842127	23	2.3	TAF4;MIR3195	Transcription
DMR20:62229001	20	62229001	2000	2	7.96E-07	-0.4597548	20	1	HRH3;LOC105369209;OSBPL2	Signaling
DMR20:62397001	20	62397001	2000	1	9.73E-09	-0.5598752	52	2.6	RPS21;CABLES2;LOC105372709	Translation
DMR20:62852001	20	62852001	2000	1	8.89E-06	0.632803	43	2.15	TCFL5;DPH3P1;LOC105372717	Transcription
DMR20:62871001	20	62871001	1000	1	1.27E-06	-0.6457188	27	2.7	TCFL5;LOC105372717;ARF4P2;DIDO1	Transcription;Transcription
DMR20:63757001	20	63757001	3000	1	8.14E-07	-0.4567613	71	2.366666667	ZBTB46	Cytoskeleton
DMR20:63824001	20	63824001	1000	1	9.77E-06	-0.4933125	23	2.3	ZBTB46;ZBTB46-AS1	Cytoskeleton
DMR20:63835001	20	63835001	2000	2	2.18E-09	-0.7821647	62	3.1	ZBTB46;C20orf181	Cytoskeleton
DMR21:5105001	21	5105001	1000	1	9.89E-06	-0.683893	27	2.7	LOC101928576	
DMR21:6551001	21	6551001	2000	2	3.09E-07	-0.5594302	65	3.25	LOC102724701;CRYAA2	
DMR21:6565001	21	6565001	2000	1	1.19E-06	-0.4614899	51	2.55	LOC102724701;CRYAA2	
DMR21:8559001	21	8559001	2000	1	1.01E-07	-0.9173448	38	1.9		
DMR21:9173001	21	9173001	2000	1	1.46E-08	-1.1424432	7	0.35		
DMR21:9250001	21	9250001	3000	1	1.75E-07	-0.7869952	13	0.433333333		
DMR21:9350001	21	9350001	3000	1	4.69E-06	-0.4094536	13	0.433333333	LOC101930100;LOC101927345	
DMR21:9373001	21	9373001	1000	1	8.50E-06	-0.4933162	7	0.7	LOC101930100;LOC101927345;SNX18P12	
DMR21:10271001	21	10271001	1000	1	8.20E-06	-0.5533402	3	0.3		
DMR21:10273001	21	10273001	1000	1	1.58E-08	-0.8161806	2	0.2		
DMR21:10324001	21	10324001	1000	1	9.76E-08	-0.8270127	3	0.3	EIF3FP1	
DMR21:10368001	21	10368001	3000	1	1.39E-08	0.6778265	35	1.166666667	VN1R7P	
DMR21:12616001	21	12616001	1000	1	5.87E-07	0.9420424	19	1.9		
DMR21:15934001	21	15934001	1000	1	1.33E-06	-0.9773987	13	1.3	LOC112268281	
DMR21:17124001	21	17124001	3000	1	1.03E-06	0.5281643	28	0.933333333		
DMR21:18829001	21	18829001	1000	1	5.57E-07	-0.7084572	20	2		
DMR21:18857001	21	18857001	1000	1	6.07E-07	0.6070446	23	2.3	PPIAP22	
DMR21:21951001	21	21951001	1000	1	7.70E-09	0.8255061	10	1		
DMR21:22451001	21	22451001	1000	1	8.09E-07	-0.4210631	21	2.1		
DMR21:25969001	21	25969001	1000	1	4.93E-06	-0.6685365	14	1.4	APP	Protease; Proteolysis
DMR21:29874001	21	29874001	1000	1	3.30E-07	0.7373454	19	1.9	GRIK1	Receptor
DMR21:30209001	21	30209001	1000	1	1.23E-06	0.7113132	10	1	LINC00307;CLDN8	Cell Junction
DMR21:31333001	21	31333001	2000	1	7.66E-08	0.6780783	34	1.7	TIAM1	
DMR21:31485001	21	31485001	1000	1	5.74E-06	0.8568802	15	1.5	TIAM1	

DMR21:33322001	21	33322001	2000	1	8.74E-07	-1.2394732	20	1	IFNAR1	Receptor
DMR21:33580001	21	33580001	1000	1	4.56E-07	-0.6393761	22	2.2	SON;LOC107985481;DONSON;CRYZL1	Translation;Metabolism
DMR21:35334001	21	35334001	1000	1	6.59E-07	1.277363	12	1.2		
DMR21:35991001	21	35991001	4000	1	7.99E-08	-0.4547893	53	1.325	LOC101928269	
DMR21:38482001	21	38482001	1000	1	8.82E-06	0.73617	16	1.6	ERG	Transcription
DMR21:39368001	21	39368001	2000	1	5.66E-06	-0.6938535	45	2.25	LOC105372804;RNF6P1	
DMR21:40138001	21	40138001	1000	1	1.43E-06	-0.4254342	10	1	DSCAM	Cytoskeleton
DMR21:40992001	21	40992001	2000	1	7.92E-06	-0.4674514	11	0.55		
DMR21:41753001	21	41753001	3000	1	9.65E-06	0.8036997	75	2.5	RIPK4;MIR6814	Signaling
DMR21:41954001	21	41954001	1000	1	1.26E-06	-0.4972032	63	6.3	C2CD2;SNORA91	
DMR21:42027001	21	42027001	2000	1	1.02E-09	-0.5534896	37	1.85	ZNF295-AS1;LOC107985502	
DMR21:42183001	21	42183001	2000	1	3.47E-06	-0.4782337	16	0.8		
DMR21:42358001	21	42358001	1000	1	3.01E-09	0.7869195	20	2	TFF2;LOC105372815;TFF1	Signaling
DMR21:42758001	21	42758001	3000	1	7.79E-06	-0.4324802	71	2.366666667	PDE9A	Signaling
DMR21:42768001	21	42768001	2000	1	3.74E-06	0.5568059	42	2.1	PDE9A;LOC107985504;LINC01668	Signaling
DMR21:42882001	21	42882001	2000	1	5.08E-06	-0.6832038	29	1.45	WDR4;LOC105372817;NDUFV3	Translation;Metabolism
DMR21:43336001	21	43336001	2000	1	1.23E-07	-0.4274323	8	0.4	LINC00322	
DMR21:43467001	21	43467001	2000	1	5.67E-07	-0.5307198	37	1.85	HSF2BP;LINC00313;LOC102723380	
DMR21:43604001	21	43604001	2000	1	1.45E-07	-0.4259725	22	1.1	HSF2BP;MIR6070	
DMR21:43915001	21	43915001	1000	1	4.86E-07	-0.7368251	15	1.5	AGPAT3;RNU6-859P	Metabolism
DMR21:43958001	21	43958001	2000	1	1.88E-07	-0.5456434	54	2.7	AGPAT3	Metabolism
DMR21:44156001	21	44156001	1000	1	3.22E-08	-0.9315856	26	2.6	LINC01678	
DMR21:44569001	21	44569001	1000	1	7.78E-06	0.8742267	27	2.7	TSPEAR;KRTAP10-4;KRTAP10-5	Signaling
DMR21:44576001	21	44576001	1000	1	3.52E-06	0.7722378	16	1.6	TSPEAR;KRTAP10-4;KRTAP10-5	Signaling
DMR21:44983001	21	44983001	2000	1	1.35E-06	-0.5898387	66	3.3	FAM207A;LINC00163;LINC00165	
DMR21:45042001	21	45042001	1000	1	1.97E-06	-0.4268298	14	1.4		
DMR21:45210001	21	45210001	1000	1	4.64E-06	0.91595	25	2.5	ADARB1	Metabolism
DMR21:45633001	21	45633001	4000	1	6.66E-07	-0.5167457	197	4.925	PCBP3	Metabolism
DMR21:45831001	21	45831001	2000	1	2.42E-08	0.6426951	38	1.9	PCBP3;PCBP3-AS1	Metabolism
DMR21:45923001	21	45923001	4000	1	4.60E-06	0.6278714	173	4.325	PCBP3	Metabolism
DMR21:46249001	21	46249001	1000	1	4.43E-06	0.6721024	17	1.7	MCM3AP-AS1;MCM3AP	Cytoskeleton
DMR22:10698001	22	10698001	2000	1	8.37E-07	-0.4700112	141	7.05		
DMR22:11021001	22	11021001	3000	2	1.91E-08	0.6183331	30	1		
DMR22:11211001	22	11211001	4000	4	2.29E-19	-1.6125197	22	0.55		
DMR22:11906001	22	11906001	1000	1	3.56E-06	-0.8399363	7	0.7	LOC102723769	
DMR22:11929001	22	11929001	3000	1	2.96E-06	-0.7687481	18	0.6	LOC102723769	
DMR22:16342001	22	16342001	22000	1	7.68E-06	-0.3117465	523	2.377272727		
DMR22:16383001	22	16383001	2000	1	1.86E-06	-0.9059869	9	0.45	ABCD1P4	
DMR22:17806001	22	17806001	2000	1	2.89E-06	0.8711778	37	1.85	MICAL3	
DMR22:18141001	22	18141001	3000	1	1.25E-06	0.7936658	59	1.966666667	TUBA8;USP18	Cytoskeleton;Protease
DMR22:18237001	22	18237001	2000	1	6.52E-12	-1.3305396	7	0.35	LOC105379518	
DMR22:18350001	22	18350001	1000	1	8.07E-06	-0.7803758	19	1.9	GGTLC5P;FAM247D	
DMR22:18387001	22	18387001	1000	1	9.17E-07	-0.9969443	8	0.8	FAM230J	
DMR22:18599001	22	18599001	2000	1	1.67E-06	-0.7520384	47	2.35	RIMBP3;RN7SKP131	
DMR22:18891001	22	18891001	2000	1	2.04E-13	-0.9215368	6	0.3	FAM230F	
DMR22:19378001	22	19378001	2000	1	1.12E-06	-0.4670586	43	2.15	HIRA	Epigenetic
DMR22:20122001	22	20122001	2000	1	5.32E-06	-0.5590644	50	2.5	TRMT2A;MIR6816;RANBP1;SNORA77B;ZDHHC8	Epigenetic;Cytoskeleton
DMR22:20687001	22	20687001	2000	1	7.60E-08	-0.6678582	91	4.55	POM121L4P;BCRP5;LOC107985584	
DMR22:20900001	22	20900001	2000	1	2.58E-06	-0.5481025	44	2.2	SNAP29	Transcription
DMR22:21254001	22	21254001	1000	1	3.62E-08	-1.0694496	4	0.4	GGT2	Protease
DMR22:21280001	22	21280001	3000	2	2.42E-06	-0.5848826	155	5.166666667	GGT2;E2F6P3;POM121L8P;BCRP6	Protease
DMR22:21433001	22	21433001	2000	1	2.25E-06	-1.0318608	34	1.7	HIC2	Transcription
DMR22:21505001	22	21505001	5000	1	6.03E-07	-0.7015612	126	2.52	PI4KAP2	Signaling
DMR22:21537001	22	21537001	4000	1	9.52E-07	-0.6841665	99	2.475	RN7SKP221;RIMBP3C;UBE2L3	Proteolysis
DMR22:21562001	22	21562001	4000	1	1.60E-06	-0.4700179	103	2.575	UBE2L3	Proteolysis
DMR22:21654001	22	21654001	2000	1	1.44E-07	-0.7823935	43	2.15	SDF2L1;LOC107985532;MIR301B;MIR130B	Transport
DMR22:22558001	22	22558001	1000	1	2.96E-06	-0.5236117	28	2.8	IGL;PRAME;LL22NC03-63E9.3	
DMR22:22652001	22	22652001	1000	1	3.41E-08	-0.6782894	21	2.1	IGL;POM121L1P;GGTLC2;LOC129026;IGLV3-29	Metabolism
DMR22:24423001	22	24423001	2000	1	3.11E-07	-0.6775069	200	10	SPECC1L-ADORA2A;SPECC1L;ADORA2A;ADORA2A-AS1	Signaling
DMR22:24659001	22	24659001	3000	2	6.05E-07	-0.6203413	138	4.6	BCRP3;POM121L10P	
DMR22:25394001	22	25394001	1000	1	9.94E-06	-0.7150092	17	1.7	LRP5L	Receptor
DMR22:26676001	22	26676001	1000	1	5.70E-07	0.7774474	10	1	MIAT;MIATNB	

DMR22:29406001	22	29406001	1000	1	3.07E-13	-0.9175468	16	1.6	RFPL1;LOC102723305	
DMR22:29827001	22	29827001	1000	1	5.38E-06	-0.7385106	11	1.1	ASCC2	
DMR22:30824001	22	30824001	1000	1	6.57E-06	0.6259362	21	2.1	OSBP2	
DMR22:31034001	22	31034001	1000	1	4.05E-09	-0.7211969	30	3		
DMR22:31067001	22	31067001	1000	1	1.03E-06	-0.5687292	22	2.2	RN7SL633P;SMTN	
DMR22:31379001	22	31379001	2000	1	3.37E-07	-0.6334181	30	1.5	RNU6-338P	
DMR22:31687001	22	31687001	1000	1	5.51E-07	-0.5518931	32	3.2	PRR14L	
DMR22:31971001	22	31971001	2000	1	5.89E-07	-0.5190363	71	3.55	LINC02558;RN7SL305P	
DMR22:33428001	22	33428001	1000	1	1.87E-06	-0.6665146	24	2.4	LARGE1;MIR4764	Golgi
DMR22:33651001	22	33651001	2000	1	8.96E-07	-0.6456901	38	1.9	LARGE1	Golgi
DMR22:34578001	22	34578001	1000	1	5.22E-16	-2.200554	7	0.7	LOC441996	
DMR22:35049001	22	35049001	2000	1	1.77E-08	1.0197603	17	0.85		
DMR22:35342001	22	35342001	2000	1	2.21E-07	-0.4608263	16	0.8	TOM1;MIR3909;MIR6069	
DMR22:36715001	22	36715001	1000	1	6.15E-06	0.6611023	11	1.1	LOC105373021	
DMR22:38048001	22	38048001	1000	1	7.22E-06	-0.5646952	15	1.5	POLR2F;PICK1	Transcription;Transport
DMR22:38217001	22	38217001	1000	1	3.39E-11	-0.6980156	33	3.3	MAFF;TMEM184B;SNORA92	Transcription;Transport
DMR22:38550001	22	38550001	2000	1	5.21E-06	-0.624169	40	2	DMC1	Transcription
DMR22:38979001	22	38979001	2000	1	4.93E-06	-0.8423098	29	1.45	LOC105373033;LOC107985562;APOBEC3B	Translation
DMR22:39396001	22	39396001	2000	2	8.30E-08	-0.6217596	40	2	TAB1	Signaling
DMR22:39982001	22	39982001	2000	1	7.12E-07	-0.6562117	28	1.4	GRAP2	
DMR22:40744001	22	40744001	4000	1	3.64E-08	-0.7237752	162	4.05		
DMR22:41291001	22	41291001	3000	1	9.18E-07	-0.4771604	92	3.066666667	RANGAP1;ZC3H7B	Signaling;Metabolism
DMR22:41587001	22	41587001	1000	1	8.34E-07	-0.6258174	27	2.7	PMM1	Metabolism
DMR22:41647001	22	41647001	1000	1	8.80E-06	-0.6000818	23	2.3	XRCC6	Epigenetic
DMR22:41785001	22	41785001	1000	1	9.33E-07	-0.526826	36	3.6	MEI1	
DMR22:42638001	22	42638001	1000	1	6.08E-06	-0.5835199	24	2.4	CYB5R3;ATP5MGL	Metabolism;Metabolism
DMR22:42706001	22	42706001	1000	1	9.87E-07	-0.6341207	26	2.6	A4GALT	Transport
DMR22:42755001	22	42755001	1000	1	7.85E-06	-0.5624019	28	2.8		
DMR22:42844001	22	42844001	1000	1	7.32E-07	-1.0206466	8	0.8	ARFGAP3;LOC692246	Signaling
DMR22:43268001	22	43268001	6000	1	1.41E-06	0.626503	145	2.416666667	SCUBE1;SCUBE1-AS1	Extracellular Matrix
DMR22:44114001	22	44114001	1000	1	2.19E-07	1.2313994	4	0.4	PARVB	Cytoskeleton
DMR22:44119001	22	44119001	2000	1	8.05E-06	0.7456038	46	2.3	PARVB	Cytoskeleton
DMR22:44744001	22	44744001	2000	1	7.34E-08	0.9855938	34	1.7	PRR5;PRR5-ARHGAP8;ARHGAP8	Signaling
DMR22:45289001	22	45289001	3000	1	6.11E-06	-0.4260226	70	2.333333333	UPK3A	
DMR22:45312001	22	45312001	1000	1	5.79E-06	0.7087235	21	2.1	FAM118A	
DMR22:45979001	22	45979001	3000	1	3.39E-06	-0.4357635	86	2.866666667	WNT7B	Signaling
DMR22:46614001	22	46614001	4000	1	5.31E-06	0.4611549	155	3.875	GRAMD4	
DMR22:47002001	22	47002001	3000	1	5.55E-06	0.4409283	42	1.4	TBC1D22A	Signaling
DMR22:47379001	22	47379001	1000	1	1.28E-06	-0.6801975	44	4.4	LOC339685	
DMR22:47431001	22	47431001	2000	1	1.93E-08	-0.6952606	41	2.05		
DMR22:47540001	22	47540001	1000	1	6.51E-08	-0.703233	30	3		
DMR22:47674001	22	47674001	2000	1	1.12E-08	0.8163066	42	2.1	LOC284930	
DMR22:48040001	22	48040001	1000	1	6.76E-06	-0.7010006	17	1.7		
DMR22:48082001	22	48082001	1000	1	1.26E-08	1.0622646	20	2		
DMR22:48580001	22	48580001	5000	1	7.00E-08	-0.4246376	113	2.26	TAFA5	Growth Factors
DMR22:48985001	22	48985001	1000	1	1.02E-06	-0.4911911	14	1.4		
DMR22:50043001	22	50043001	1000	1	7.44E-14	0.7489404	40	4	TTLL8	
DMR22:50337001	22	50337001	4000	1	3.89E-08	-0.4310684	43	1.075	DENND6B;PPP6R2	Signaling
DMR22:50680001	22	50680001	4000	1	1.40E-06	0.992547	77	1.925	SHANK3;RNU6-409P	
DMR22:50732001	22	50732001	2000	1	8.90E-06	0.7219813	60	3	SHANK3;LOC105373100;ACR	Protease
DMRX:321001	X	321001	3000	1	1.59E-06	-0.6958399	80	2.666666667	GTPBP6;LINC00685;PPP2R3B	Signaling
DMRX:427001	X	427001	1000	1	3.57E-06	0.5479587	67	6.7	LOC102724521	
DMRX:430001	X	430001	2000	1	6.04E-06	0.7248748	59	2.95	LOC102724521	
DMRX:530001	X	530001	2000	1	9.10E-07	0.6770226	37	1.85	FABP5P13	
DMRX:570001	X	570001	1000	1	1.08E-09	-1.0936253	21	2.1		
DMRX:737001	X	737001	2000	1	1.53E-07	0.747453	16	0.8		
DMRX:818001	X	818001	2000	1	8.23E-17	-2.7741201	24	1.2		
DMRX:873001	X	873001	1000	1	2.59E-06	-0.847625	17	1.7		
DMRX:893001	X	893001	1000	1	5.24E-07	0.8244316	2	0.2		
DMRX:970001	X	970001	5000	1	1.58E-07	0.9222438	70	1.4		
DMRX:1146001	X	1146001	2000	1	2.19E-09	0.8411247	53	2.65		
DMRX:1176001	X	1176001	2000	2	4.45E-09	-1.091435	40	2	LOC652608	
DMRX:1219001	X	1219001	3000	1	4.51E-06	0.5531005	38	1.266666667	CRLF2	Receptor
DMRX:1342001	X	1342001	3000	1	5.34E-06	-0.4570954	101	3.366666667	IL3RA;LOC101928032;LOC101928055	Receptor
DMRX:1658001	X	1658001	1000	1	3.63E-06	-0.6283216	34	3.4	LOC107985706	
DMRX:1728001	X	1728001	1000	1	2.11E-08	-0.8751734	17	1.7	LOC107985637;LOC105373105	
DMRX:1801001	X	1801001	2000	1	2.76E-08	0.7887804	12	0.6		

DMRX:3434001	X	3434001	2000	1	9.12E-06	0.6824972	29	1.45		
DMRX:3655001	X	3655001	2000	1	6.44E-08	0.7299303	43	2.15	PRKX;PRKX-AS1	Signaling
DMRX:3757001	X	3757001	1000	1	9.46E-07	-0.7009539	30	3		
DMRX:4227001	X	4227001	1000	1	4.76E-10	-1.1790147	2	0.2		
DMRX:6704001	X	6704001	1000	1	2.77E-06	0.5445679	15	1.5		
DMRX:8743001	X	8743001	1000	1	6.85E-06	-0.7897293	17	1.7		
DMRX:10002001	X	10002001	1000	1	6.43E-07	-0.6612094	25	2.5		
DMRX:12560001	X	12560001	1000	1	7.46E-06	-0.6641782	17	1.7	FRMPD4	
DMRX:15234001	X	15234001	1000	1	5.91E-06	-0.7661161	18	1.8	LOC102724092;ASB9	
DMRX:15351001	X	15351001	1000	1	5.01E-06	-0.6353096	28	2.8	PIR-FIGF;VEGFD	Growth Factors
DMRX:29236001	X	29236001	1000	1	9.85E-07	-0.9209087	15	1.5	IL1RAPL1	Receptor
DMRX:30083001	X	30083001	1000	1	1.36E-06	-0.6763604	19	1.9		
DMRX:40072001	X	40072001	2000	1	2.62E-08	0.9481686	60	3	BCOR	
DMRX:40246001	X	40246001	1000	1	9.19E-06	-0.8129376	18	1.8		
DMRX:40302001	X	40302001	1000	1	2.49E-06	-0.6118564	21	2.1		
DMRX:40768001	X	40768001	1000	1	5.81E-06	-0.7428453	23	2.3	DPRXP6;CLDN7P1	
DMRX:41244001	X	41244001	1000	1	5.83E-06	-0.7989872	27	2.7	USP9X	Protease
DMRX:41438001	X	41438001	1000	1	5.36E-06	-0.9784536	17	1.7	NYX	Receptor
DMRX:44505001	X	44505001	1000	1	5.60E-06	-0.6310909	27	2.7		
DMRX:44565001	X	44565001	1000	1	7.15E-06	-0.5864745	36	3.6		
DMRX:44849001	X	44849001	1000	1	2.94E-06	-0.5382529	44	4.4	DUSP21	Signaling
DMRX:46835001	X	46835001	1000	1	5.40E-06	-0.7094135	43	4.3	RP2	Signaling
DMRX:47356001	X	47356001	3000	1	1.59E-07	-0.6525123	85	2.833333333		
DMRX:47476001	X	47476001	2000	1	3.89E-07	-0.8452736	38	1.9	ZNF41;LINC01560	
DMRX:48743001	X	48743001	1000	1	8.11E-08	-0.7782657	18	1.8		
DMRX:49013001	X	49013001	3000	1	2.38E-07	-0.7902843	81	2.7		
DMRX:49091001	X	49091001	1000	1	5.67E-08	-0.5513909	50	5	WDR45;RNU4-52P	
DMRX:49128001	X	49128001	2000	1	1.18E-06	-0.6472843	59	2.95	GPKOW;LOC105373195	Translation
DMRX:50429001	X	50429001	1000	1	2.93E-06	-0.7170258	30	3	DGKK	Signaling
DMRX:54094001	X	54094001	1000	1	4.17E-08	-0.9304009	19	1.9	FAM120C	
DMRX:54864001	X	54864001	1000	1	9.44E-06	-0.6113953	18	1.8		
DMRX:62462001	X	62462001	43000	1	1.53E-07	-0.7273101	856	1.990697674		
DMRX:65862001	X	65862001	1000	1	4.76E-06	0.9232612	9	0.9		
DMRX:70096001	X	70096001	1000	1	9.07E-06	0.7817942	10	1		
DMRX:72223001	X	72223001	2000	1	1.49E-07	-0.9093806	35	1.75	PIN4;ERCC6L	
DMRX:72843001	X	72843001	1000	1	1.43E-06	-0.5806617	44	4.4	DMRTC1B	Transcription
DMRX:74519001	X	74519001	1000	1	5.11E-09	-0.9877016	19	1.9	SLC16A2	Transport
DMRX:75713001	X	75713001	1000	1	7.94E-06	1.2035291	15	1.5	LOC107985664	
DMRX:78072001	X	78072001	1000	1	1.17E-07	-0.8879742	31	3.1		
DMRX:85254001	X	85254001	1000	1	6.90E-07	-0.6138941	49	4.9	ZNF711	Transcription
DMRX:92132001	X	92132001	1000	1	6.55E-09	-0.8701277	24	2.4	PCDH11X	Cytoskeleton
DMRX:94483001	X	94483001	1000	1	8.68E-06	-0.9353177	2	0.2		
DMRX:95638001	X	95638001	1000	1	3.46E-06	-0.6549152	14	1.4	HNRNPDLP1	
DMRX:98848001	X	98848001	1000	1	6.85E-09	-1.9861431	10	1		
DMRX:105388001	X	105388001	1000	1	2.60E-09	-1.0740168	6	0.6	IL1RAPL2	Receptor
DMRX:116462001	X	116462001	2000	1	3.24E-06	1.0360922	38	1.9	SLC6A14;CT83	Transport
DMRX:120631001	X	120631001	1000	1	1.57E-06	-0.5629623	54	5.4	MCTS1;C1GALT1C1	Translation;Transport
DMRX:123051001	X	123051001	2000	1	8.38E-08	-0.726618	34	1.7		
DMRX:123770001	X	123770001	1000	1	3.43E-06	-0.5667033	22	2.2	MEMO1P4;RNU7-69P	
DMRX:124852001	X	124852001	2000	1	2.05E-06	0.5781379	15	0.75	TENM1	
DMRX:133344001	X	133344001	1000	1	6.20E-07	-0.6213235	16	1.6	GPC4	
DMRX:141265001	X	141265001	1000	1	8.33E-25	-1.1643439	14	1.4	RBMX2P2	
DMRX:142058001	X	142058001	2000	1	1.45E-06	0.9927449	48	2.4		
DMRX:142813001	X	142813001	1000	1	5.74E-08	-0.8505832	31	3.1		
DMRX:143514001	X	143514001	2000	1	2.09E-06	0.9914428	20	1	SPANXN3	
DMRX:146248001	X	146248001	1000	1	5.49E-06	-0.6824841	28	2.8		
DMRX:146733001	X	146733001	1000	1	2.41E-06	-0.5475911	14	1.4		
DMRX:147883001	X	147883001	1000	1	6.01E-06	-0.7603669	30	3	LOC105373349	
DMRX:151912001	X	151912001	8000	1	6.91E-06	-0.8614657	292	3.65	MAGEA4-AS1;MAGEA4	Cytoskeleton
DMRX:152444001	X	152444001	1000	1	5.47E-06	-0.7452022	21	2.1	GABRA3	Ion Channel
DMRX:153512001	X	153512001	1000	1	4.26E-08	-0.6682045	13	1.3	BGN;ATP2B3	Transport
DMRX:153978001	X	153978001	2000	1	8.79E-06	-0.6095233	51	2.55	HCFC1;HCFC1-AS1;TMEM187;MIR3202-1;MIR3202-2	
DMRY:4895001	Y	4895001	1000	1	3.94E-08	-0.7301292	57	5.7		
DMRY:5815001	Y	5815001	1000	1	7.17E-08	0.875155	23	2.3		
DMRY:6264001	Y	6264001	2000	1	8.07E-07	-0.5582446	97	4.85	FAM197Y9;TSPY11P	
DMRY:6922001	Y	6922001	1000	1	3.25E-09	1.3020067	34	3.4	TBL1Y	
DMRY:7325001	Y	7325001	1000	1	7.04E-07	0.8723978	20	2	PRKY;RN7SKP282	

DMRY:9336001	Y	9336001	3000	1	2.24E-08	-0.6132637	142	4.733333333	RBMY1GP;TTTY20;TSPY4	Epigenetic
DMRY:9356001	Y	9356001	3000	1	5.17E-07	-0.4671773	159	5.3	FAM197Y8;TSPY8	Epigenetic
DMRY:9377001	Y	9377001	2000	1	2.16E-06	-0.4291162	109	5.45	FAM197Y7;TSPY7P	
DMRY:9482001	Y	9482001	2000	1	1.61E-08	-0.824409	20	1	FAM197Y4;TSPY9P	Epigenetic
DMRY:9485001	Y	9485001	3000	1	1.67E-06	-0.459701	160	5.333333333	FAM197Y4;TSPY9P	Epigenetic
DMRY:10744001	Y	10744001	109000	2	3.05E-06	-0.4870666	1070	0.981651376		
DMRY:10855001	Y	10855001	42000	1	2.65E-06	-0.4247186	383	0.911904762		
DMRY:10906001	Y	10906001	2000	1	1.05E-06	-0.8097815	16	0.8		
DMRY:10967001	Y	10967001	74000	4	2.58E-06	-0.4569229	572	0.772972973		
DMRY:11287001	Y	11287001	18000	1	3.01E-06	-0.4185209	186	1.033333333	DUX4L16;DUX4L17	
DMRY:11321001	Y	11321001	2000	1	9.81E-06	-0.3143592	121	6.05	DUX4L17;DUX4L18;DUX4L19	
DMRY:11697001	Y	11697001	10000	1	5.50E-06	-0.370837	231	2.31		
DMRY:11708001	Y	11708001	6000	1	6.22E-06	-0.3949809	146	2.433333333		
DMRY:14036001	Y	14036001	1000	1	2.59E-06	-0.7217488	36	3.6		
DMRY:15237001	Y	15237001	1000	1	2.07E-07	-0.6466208	23	2.3	LOC107987355	
DMRY:15882001	Y	15882001	2000	2	1.67E-08	-0.9554528	29	1.45		
DMRY:15896001	Y	15896001	2000	1	4.89E-10	-0.9594604	31	1.55		
DMRY:18335001	Y	18335001	2000	1	3.05E-07	-0.5340662	55	2.75	FAM224A;RNA5SP522	
DMRY:19552001	Y	19552001	1000	1	1.70E-06	-0.7117952	4	0.4	LOC105377223	
DMRY:23061001	Y	23061001	1000	1	4.70E-07	-0.6933198	29	2.9	ZNF736P3Y;TRIM60P9Y	
DMRY:24695001	Y	24695001	1000	1	9.15E-07	-0.7504781	27	2.7	TRIM60P11Y	
DMRY:26638001	Y	26638001	36000	2	6.18E-06	-0.4117581	643	1.786111111	PARP4P1	
DMRY:56860001	Y	56860001	3000	1	3.52E-06	-0.7407607	19	0.633333333	CTBP2P1	
DMRY:56875001	Y	56875001	2000	1	2.94E-07	-0.511483	7	0.35		

Supplemental Table S2. DMR table for mild (distal) hypospadias versus control p<1e-05. DMR name, chromosome number, start nucleotide site, length (bp), p-value, maximum log fold change (LFC), CpG number and density, gene annotation, and gene category.

Supplemental Table S3
DMR Table Moderate versus Control p<1e-05

DMR Name	Chr	Start	Length	# Sig Win	minP	maxLFC	CpG #	CpG Density	Gene Annotation	Gene Category
DMR1:1102001	1	1102001	1000	1	3.49E-06	-0.8495741	10	1	C1orf159	
DMR1:9603001	1	9603001	1000	1	1.59E-06	-0.7442713	28	2.8	TMEM201	
DMR1:10784001	1	10784001	3000	1	8.50E-06	0.5615164	67	2.233333333	CASZ1	Transcription
DMR1:85552001	1	85552001	1000	1	7.23E-07	1.00363	5	0.5	DDAH1	Metabolism
DMR1:122462001	1	122462001	1000	1	3.28E-06	-0.6001338	20	2		
DMR1:146808001	1	146808001	1000	1	2.59E-06	0.7310347	10	1	HYDIN2	
DMR1:161725001	1	161725001	1000	1	2.32E-06	-0.7098047	30	3	FCRLB;RN7SL466P	Immune
DMR1:189562001	1	189562001	1000	1	5.75E-06	-1.4217269	0	0	LOC105371657	
DMR2:23831001	2	23831001	1000	1	4.06E-07	0.7200568	4	0.4	ATAD2B	Epigenetic
DMR2:68449001	2	68449001	3000	1	1.61E-08	-1.8757408	10	0.333333333	WDR4P2;FBXO48	
DMR2:168975001	2	168975001	1000	1	1.68E-10	-2.0579939	0	0	ABCB11	Transport
DMR2:169465001	2	169465001	1000	1	3.31E-10	-2.4345864	3	0.3		
DMR2:174755001	2	174755001	1000	1	8.98E-07	-0.9512696	19	1.9	CHRNA1	Ion Channel
DMR2:196603001	2	196603001	1000	1	3.61E-07	-0.7438519	11	1.1	HECW2	Proteolysis
DMR2:205380001	2	205380001	1000	1	1.02E-10	-1.9437439	2	0.2	PARD3B	
DMR3:16744001	3	16744001	1000	1	9.57E-06	0.5353258	14	1.4		
DMR3:18826001	3	18826001	2000	1	2.60E-09	-2.0740348	18	0.9		
DMR3:62461001	3	62461001	1000	1	4.93E-06	0.8012924	10	1	CADPS	Transport
DMR3:81032001	3	81032001	1000	1	1.94E-06	-0.7899964	24	2.4	LINC02027	
DMR3:91548001	3	91548001	3000	1	3.80E-06	-0.5467575	43	1.433333333		
DMR3:92384001	3	92384001	1000	1	7.49E-06	-0.7117281	19	1.9		
DMR3:134395001	3	134395001	2000	1	1.28E-06	0.7773984	17	0.85	HMGN1P9	
DMR4:49709001	4	49709001	3000	3	6.47E-07	-0.7733204	11	0.366666667		
DMR4:51107001	4	51107001	1000	1	8.09E-09	-0.8414533	7	0.7		
DMR4:150489001	4	150489001	1000	1	3.88E-07	-1.6082281	3	0.3	LRBA	
DMR4:168155001	4	168155001	1000	1	4.80E-07	-1.3156751	4	0.4	ANXA10	Signaling
DMR5:48135001	5	48135001	1000	1	3.56E-06	-1.0348463	19	1.9		
DMR5:48849001	5	48849001	2000	1	6.77E-06	-0.8780894	35	1.75		
DMR5:50645001	5	50645001	1000	1	6.52E-06	-1.7119825	1	0.1		
DMR5:68130001	5	68130001	1000	1	9.50E-06	-0.6701974	11	1.1		
DMR5:128731001	5	128731001	1000	1	3.49E-06	0.788048	10	1	LOC105379168	
DMR5:149650001	5	149650001	1000	1	3.85E-11	-2.1965393	0	0		
DMR5:158218001	5	158218001	1000	1	1.66E-06	0.6812284	9	0.9		
DMR6:3135001	6	3135001	2000	1	7.27E-06	0.7066636	31	1.55	BPHL	Metabolism
DMR6:89896001	6	89896001	1000	1	2.49E-06	0.5451446	7	0.7	LOC644269;GJA10	Cytoskeleton
DMR6:101760001	6	101760001	1000	1	1.75E-07	-1.1390759	13	1.3	GRIK2	Receptor
DMR6:158169001	6	158169001	1000	1	5.91E-07	-1.0947898	19	1.9	SERAC1;GTF2H5	Transcription
DMR6:166749001	6	166749001	1000	1	5.12E-06	0.8114996	16	1.6	RPS6KA2	Golgi
DMR6:166760001	6	166760001	1000	1	9.21E-06	0.7714006	18	1.8	RPS6KA2	Golgi
DMR7:29749001	7	29749001	1000	1	1.94E-07	0.7751734	10	1	DPY19L2P3	
DMR7:63039001	7	63039001	1000	1	7.36E-06	0.8600539	12	1.2		
DMR7:72458001	7	72458001	1000	1	1.61E-08	-2.0033356	2	0.2	CALN1	
DMR7:120055001	7	120055001	1000	1	9.12E-06	1.0761624	10	1		
DMR7:135528001	7	135528001	1000	1	1.84E-06	0.8974702	10	1		
DMR8:33319001	8	33319001	1000	1	3.73E-07	-2.2196083	0	0	FUT10	Golgi
DMR8:43237001	8	43237001	5000	5	1.50E-10	-1.1304151	15	0.3	LOC101059977;AFG3L2P1	
DMR8:93644001	8	93644001	1000	1	1.16E-06	-1.5983027	3	0.3	CIBAR1-DT;ZNF317P1	
DMR8:124302001	8	124302001	1000	1	1.24E-12	-1.3067648	5	0.5	LOC112268031;TMEM65	
DMR9:32360001	9	32360001	1000	1	5.49E-09	-2.2977938	2	0.2	LOC107987059	
DMR9:33916001	9	33916001	1000	1	6.88E-06	0.6238816	18	1.8	UBE2R2;UBAP2	
DMR9:90216001	9	90216001	1000	1	4.06E-07	0.7437479	20	2	OR7E31P	
DMR9:110690001	9	110690001	1000	1	1.99E-08	-1.8185854	2	0.2	MUSK	Receptor
DMR9:132998001	9	132998001	1000	1	4.77E-06	-0.7221563	20	2	GFI1B	Transcription
DMR10:133686001	10	133686001	4000	1	7.64E-06	-0.5156055	25	0.625	DUX4L22;DUX4L21;DUX4L20	
DMR11:73251001	11	73251001	2000	1	2.80E-06	-1.6006911	26	1.3	P2RY2;OR8R1P;LOC105369382	Signaling
DMR12:2638001	12	2638001	1000	1	3.23E-06	0.5304332	17	1.7	CACNA1C	Transport
DMR12:5279001	12	5279001	2000	1	6.89E-08	1.3430982	20	1	LOC105369617	
DMR12:30125001	12	30125001	1000	1	1.59E-06	0.66	8	0.8		
DMR12:57879001	12	57879001	1000	1	1.82E-06	0.4747671	4	0.4	LOC283387	
DMR12:127958001	12	127958001	1000	1	2.35E-06	-0.7358498	18	1.8	LINC00508;LINC00507	
DMR13:23866001	13	23866001	2000	1	5.41E-06	0.7612855	29	1.45	MIPEP	Protease
DMR13:109424001	13	109424001	1000	1	1.29E-06	-0.7920961	9	0.9		

DMR14:16093001	14	16093001	2000	1	9.41E-09	-0.951005	5	0.25		
DMR14:55489001	14	55489001	1000	1	4.39E-07	-1.4464609	9	0.9		
DMR15:49099001	15	49099001	1000	1	2.47E-06	0.7568769	4	0.4		
DMR15:74713001	15	74713001	1000	1	4.17E-06	-0.8978868	11	1.1	CYP1A1	Metabolism
DMR15:80004001	15	80004001	2000	1	1.30E-06	-1.9533234	8	0.4		
DMR15:80378001	15	80378001	1000	1	5.60E-06	0.6639793	6	0.6		
DMR16:10644001	16	10644001	1000	1	2.16E-06	0.7913631	12	1.2	TEKT5	Cytoskeleton
DMR16:29713001	16	29713001	1000	1	1.75E-08	-1.6358437	4	0.4		
DMR16:69912001	16	69912001	2000	1	4.74E-06	-0.5338395	33	1.65	WWP2	Proteolysis
DMR16:87076001	16	87076001	1000	1	4.93E-06	0.615423	25	2.5		
DMR17:1812001	17	1812001	1000	1	8.20E-06	-0.6768329	14	1.4	SMYD4	
DMR17:4586001	17	4586001	1000	1	3.06E-06	0.577587	14	1.4	SMTNL2	
DMR17:50086001	17	50086001	1000	1	3.76E-06	-0.7234957	15	1.5	ITGA3;PDK2	Extracellular Matrix;Signaling
DMR17:52335001	17	52335001	1000	1	6.97E-06	-0.8195384	6	0.6		
DMR18:30490001	18	30490001	1000	1	5.75E-07	-1.5763301	2	0.2		
DMR18:47850001	18	47850001	1000	1	1.75E-09	-1.5902211	3	0.3	SMAD2;MTCO2P2	Transcription
DMR18:61970001	18	61970001	1000	1	6.53E-11	-2.1057268	0	0	LOC105372158	
DMR18:76432001	18	76432001	1000	1	5.88E-06	0.5467351	22	2.2	ZNF516	Transcription
DMR19:11268001	19	11268001	1000	1	8.62E-07	-0.8472115	25	2.5	DOCK6	Transcription
DMR19:14377001	19	14377001	1000	1	9.86E-06	-0.6188354	20	2	ADGRE5	Signaling
DMR19:25087001	19	25087001	2000	1	4.33E-06	-0.9655574	29	1.45		
DMR19:54045001	19	54045001	2000	1	1.90E-07	0.7044139	18	0.9	VSTM1	Immune
DMR20:37431001	20	37431001	1000	1	6.71E-06	0.6091376	13	1.3		
DMR20:38124001	20	38124001	1000	1	9.29E-06	-1.0133876	7	0.7	TGM2	Transport
DMR20:46854001	20	46854001	1000	1	7.26E-06	0.78876	17	1.7	RN7SKP33;RPL13P14	
DMR20:63280001	20	63280001	1000	1	8.64E-06	-0.7533013	24	2.4	ARFGAP1;MIR4326	Signaling
DMR21:44902001	21	44902001	2000	1	1.91E-06	0.6731946	68	3.4	ITGB2;LOC107987303	Extracellular Matrix
DMR22:11211001	22	11211001	4000	3	4.47E-07	-0.9981185	22	0.55		
DMR22:18204001	22	18204001	2000	1	3.29E-09	-2.2868193	6	0.3	FAM230D;LOC105372858	
DMR22:18238001	22	18238001	1000	1	6.11E-07	-0.9924672	5	0.5	LOC105379518	
DMR22:18891001	22	18891001	2000	1	1.38E-07	-0.7392631	6	0.3	FAM230F	
DMRX:818001	X	818001	2000	1	4.58E-06	-1.815608	24	1.2		
DMRX:123051001	X	123051001	2000	1	1.96E-06	-0.6793812	34	1.7		

Supplemental Table S3. DMR table for moderate (mid shaft) hypospadias versus control p<1e-05. DMR name, chromosome number, start nucleotide site, length (bp), p-value, maximum log fold change (LFC), CpG number and density, gene annotation, and gene category.

Supplemental Table S4
DMR Table Severe versus Control p<1e-05

DMR Name	Chr	Start	Length	# Sig Win	minP	maxLFC	CpG #	CpG Density	Gene Annotation	Gene Category
DMR2:68451001	2	68451001	1000	1	3.22E-06	-1.6616287	0	0	WDR4P2;FBXO48	
DMR2:168975001	2	168975001	1000	1	6.71E-06	-1.6645728	0	0	ABCB11	Transport
DMR2:169465001	2	169465001	1000	1	7.88E-06	-1.6841439	3	0.3		
DMR3:18826001	3	18826001	1000	1	2.26E-06	-1.8754874	8	0.8		
DMR3:136934001	3	136934001	1000	1	8.62E-06	0.716987	12	1.2	NCK1	Cytoskeleton
DMR4:18254001	4	18254001	1000	1	1.34E-06	0.7569075	7	0.7		
DMR4:150489001	4	150489001	1000	1	3.08E-09	-1.8882677	3	0.3	LRBA	
DMR5:1290001	5	1290001	1000	1	1.96E-06	0.7041965	87	8.7	TERT	Transcription
DMR5:149650001	5	149650001	1000	1	5.25E-09	-2.1269875	0	0		
DMR8:12560001	8	12560001	1000	1	5.89E-06	-0.8017599	43	4.3	LOC100506990;LOC729732;RPS3AP34	
DMR8:33319001	8	33319001	1000	1	2.46E-06	-1.9972711	0	0	FUT10	Golgi
DMR8:124302001	8	124302001	1000	1	1.19E-06	-0.972987	5	0.5	LOC112268031;TMEM65	
DMR10:124524001	10	124524001	1000	1	2.42E-06	-0.8765964	12	1.2	LHPP	Signaling
DMR14:80296001	14	80296001	1000	1	8.11E-06	-0.7359918	13	1.3	DIO2-AS1	
DMR14:103133001	14	103133001	1000	1	1.82E-06	0.7786238	37	3.7	TNFAIP2;NDUFB4P11	Transport
DMR15:23706001	15	23706001	1000	1	3.30E-07	-0.820992	21	2.1		
DMR16:36853001	16	36853001	1000	1	9.56E-06	2.3538889	21	2.1		
DMR16:74711001	16	74711001	1000	1	2.23E-06	-0.8924371	13	1.3	FA2H	Metabolism
DMR18:61970001	18	61970001	1000	1	6.63E-11	-2.0872447	0	0	LOC105372158	
DMR19:103001	19	103001	1000	1	9.91E-06	1.0800642	14	1.4	OR4G3P;OR4G1P;OR4F17	Receptor
DMR22:38943001	22	38943001	1000	1	1.30E-08	-1.1264873	18	1.8		
DMRX:75178001	X	75178001	1000	1	4.72E-07	0.7717193	42	4.2	UPRT	Signaling

Supplemental Table S4. DMR table for severe (proximal) hypospadias versus control p<1e-05. DMR name, chromosome number, start nucleotide site, length (bp), p-value, maximum log fold change (LFC), CpG number and density, gene annotation, and gene category.

Supplemental Table S5
DMR Table All versus Control p<1e-05

DMR Name	Chr	Start	Length	# Sig Win	minP	maxLFC	CpG #	CpG Density	Gene Annotation	Gene Category
DMR1:820001	1	820001	4000	1	9.33E-07	0.7390545	37	0.925	FAM87B;LINC00115;LINC01128	
DMR1:1102001	1	1102001	1000	1	8.21E-06	-0.6449267	10	1	C1orf159	
DMR1:1720001	1	1720001	2000	1	2.16E-07	-0.5102853	49	2.45	CDK11A;SLC35E2A	Signaling;Transport
DMR1:6651001	1	6651001	1000	1	5.47E-06	0.7012049	20	2	DNAJC11	Transcription
DMR1:7163001	1	7163001	1000	1	3.58E-06	0.6339129	13	1.3	CAMTA1	Transcription
DMR1:9122001	1	9122001	2000	1	2.34E-07	-1.2701007	22	1.1	GPR157	Signaling
DMR1:9476001	1	9476001	4000	1	9.17E-06	0.6586825	58	1.45		
DMR1:11833001	1	11833001	2000	1	7.73E-06	0.6093002	45	2.25	CLCN6;NPPA-AS1	Transport
DMR1:13951001	1	13951001	1000	1	1.38E-07	1.3468866	5	0.5	KAZN	
DMR1:17412001	1	17412001	1000	1	1.91E-06	0.9617695	17	1.7	RCC2;RCC2-AS1	
DMR1:20512001	1	20512001	2000	1	3.98E-06	-0.7202658	33	1.65	MUL1	
DMR1:20712001	1	20712001	1000	1	1.51E-06	-1.2733896	11	1.1	KIF17;SH2D5	Cytoskeleton;Cytoskeleton
DMR1:32865001	1	32865001	2000	1	2.00E-06	-0.5504369	24	1.2	S100PBP;FNDC5;LOC105378631	
DMR1:37953001	1	37953001	1000	1	4.59E-06	-0.9086886	22	2.2	INPP5B;LOC105378651;SF3A3	Signaling;Translation
DMR1:41328001	1	41328001	1000	1	3.27E-07	0.8186293	11	1.1		
DMR1:43851001	1	43851001	1000	1	6.69E-06	0.6421616	24	2.4	ST3GAL3;SHMT1P1	Transport
DMR1:48547001	1	48547001	1000	1	4.31E-06	0.6277564	8	0.8	AGBL4	Protease
DMR1:52797001	1	52797001	1000	1	2.67E-07	-0.7757449	17	1.7	ZYG11B;RNU6-969P	
DMR1:62081001	1	62081001	1000	1	4.19E-06	-0.8940264	9	0.9	PATJ;RN7SL180P;MIR3116-1;MIR3116-2	
DMR1:63422001	1	63422001	1000	1	7.19E-07	-0.839703	7	0.7	ALG6	Golgi
DMR1:65530001	1	65530001	2000	1	3.70E-08	0.7611034	15	0.75	LEPR	Receptor
DMR1:67163001	1	67163001	1000	1	2.99E-07	-0.7040023	6	0.6	IL23R	Receptor
DMR1:68347001	1	68347001	1000	1	7.62E-06	-0.6915358	16	1.6		
DMR1:69916001	1	69916001	1000	1	5.32E-11	-1.7467411	6	0.6	LRRC7;PIN1P1	Cytoskeleton;Transcription
DMR1:70480001	1	70480001	2000	1	8.61E-06	-0.6460464	32	1.6		
DMR1:75280001	1	75280001	1000	1	1.06E-14	-2.5279533	2	0.2	SLC44A5	Transport
DMR1:77367001	1	77367001	1000	1	1.77E-08	-1.4968824	4	0.4	AK5	Signaling
DMR1:85552001	1	85552001	1000	1	9.47E-06	0.8342677	5	0.5	DDAH1	Metabolism
DMR1:86797001	1	86797001	1000	1	9.16E-09	-1.1800087	7	0.7		
DMR1:90685001	1	90685001	2000	1	2.02E-06	-0.5374309	42	2.1		
DMR1:91277001	1	91277001	1000	1	3.55E-08	-1.2749975	3	0.3	HFM1	Transcription
DMR1:98215001	1	98215001	1000	1	4.59E-06	-0.4237448	18	1.8	LINC01776	
DMR1:105707001	1	105707001	1000	1	1.57E-08	-0.7639112	9	0.9	SEPTIN2P1	
DMR1:106483001	1	106483001	1000	1	1.05E-06	0.7498762	6	0.6	LOC105378887	
DMR1:111502001	1	111502001	1000	1	3.74E-06	-1.3071551	2	0.2	TMIGD3;ADORA3	Immune;Signaling
DMR1:111995001	1	111995001	1000	1	1.75E-06	0.6195852	1	0.1	KCND3;LINC01750;LOC105378906	Transport
DMR1:114205001	1	114205001	1000	1	2.11E-07	-0.6090088	10	1	LOC107985443	
DMR1:116437001	1	116437001	1000	1	5.48E-06	-1.4448727	6	0.6	LINC01762	
DMR1:122462001	1	122462001	1000	1	4.90E-08	-0.5070493	20	2		
DMR1:144845001	1	144845001	3000	1	1.72E-07	-0.5079355	53	1.7666667	LOC105371217	
DMR1:146808001	1	146808001	2000	1	3.97E-09	0.7628939	21	1.05	HYDIN2	
DMR1:149825001	1	149825001	1000	1	4.44E-06	-0.547504	22	2.2	H4C14	
DMR1:150247001	1	150247001	1000	1	6.22E-07	-0.5952453	24	2.4	RNU2-17P;CA14	
DMR1:150266001	1	150266001	1000	1	1.35E-06	0.6972268	15	1.5	CA14;SNORD13C;APH1A;C1orf54	Protease
DMR1:151321001	1	151321001	1000	1	1.56E-06	-0.6732653	13	1.3	PI4KB	Signaling
DMR1:154189001	1	154189001	1000	1	9.48E-06	-0.4789246	20	2	TPM3;MIR190B;C1orf189	Cytoskeleton
DMR1:154754001	1	154754001	1000	1	8.52E-06	0.5137685	18	1.8	KCNN3	Transport
DMR1:159975001	1	159975001	2000	1	8.02E-06	-0.8357366	6	0.3	SLAMF9;LINC01133;FCRL6P1	Immune
DMR1:161725001	1	161725001	1000	1	1.37E-08	-0.6668572	30	3	FCRLB;RN7SL466P	Immune
DMR1:162727001	1	162727001	1000	1	1.15E-07	-1.4358803	2	0.2	DDR2	Receptor
DMR1:166878001	1	166878001	2000	1	9.43E-08	0.6490021	30	1.5	TADA1;DUTP6	
DMR1:182338001	1	182338001	1000	1	3.42E-06	0.9956123	14	1.4	RPL18P2;EIF1P3	
DMR1:189562001	1	189562001	1000	1	7.45E-10	-1.9500117	0	0	LOC105371657	
DMR1:201684001	1	201684001	1000	1	5.08E-06	-0.5368579	19	1.9	NAV1;IPO9-AS1	
DMR1:208288001	1	208288001	1000	1	2.09E-06	0.6343097	8	0.8	LOC105372889	
DMR1:221372001	1	221372001	1000	1	1.78E-06	1.0190668	13	1.3	LOC105372932	
DMR1:222461001	1	222461001	1000	1	5.20E-10	-0.7481067	8	0.8	TRT-TGT2-1;CICP13;LOC728417	
DMR1:226013001	1	226013001	1000	1	1.03E-06	0.7237603	10	1		
DMR1:229443001	1	229443001	1000	1	3.15E-06	-0.7171424	11	1.1	ACTA1;NUP133	Cytoskeleton
DMR1:232368001	1	232368001	1000	1	9.17E-06	-0.8452797	0	0	LOC105373196;LOC105373198	
DMR1:246413001	1	246413001	1000	1	2.84E-06	0.8874041	12	1.2	SMYD3	Epigenetic

DMR1:246636001	1	246636001	4000	1	2.21E-06	0.5970099	88	2.2	CNST;LOC100887078;LOC107985100	
DMR1:246922001	1	246922001	1000	1	1.53E-06	-0.6638901	24	2.4	AHCTF1	Cytoskeleton
DMR2:926001	2	926001	2000	1	2.25E-06	0.7658544	49	2.45	LOC105373481	
DMR2:1164001	2	1164001	2000	1	1.86E-07	0.9704003	34	1.7	SNTG2	
DMR2:2692001	2	2692001	3000	1	1.31E-07	0.7602293	61	2.0333333		
DMR2:3312001	2	3312001	1000	1	1.00E-06	0.845631	18	1.8	EIPR1	
DMR2:4864001	2	4864001	1000	1	1.44E-06	0.8393313	15	1.5		
DMR2:11090001	2	11090001	1000	1	3.63E-07	-1.1141967	3	0.3	FLJ33534	
DMR2:12583001	2	12583001	1000	1	9.70E-06	0.694085	10	1	MIR3681HG	
DMR2:16143001	2	16143001	1000	1	3.71E-09	-1.284539	10	1		
DMR2:18755001	2	18755001	1000	1	3.78E-08	-2.5416608	0	0	LOC105373456	
DMR2:23831001	2	23831001	1000	1	5.29E-07	0.6261945	4	0.4	ATAD2B	Epigenetic
DMR2:25004001	2	25004001	2000	1	8.61E-06	-0.6894584	48	2.4	DNAJC27-AS1	
DMR2:26418001	2	26418001	1000	1	2.61E-09	-1.4827965	8	0.8	DRC1	
DMR2:27750001	2	27750001	1000	1	7.00E-06	-0.5569047	15	1.5		
DMR2:29907001	2	29907001	1000	1	2.42E-06	0.566878	10	1	ALK	Receptor
DMR2:32110001	2	32110001	2000	1	7.61E-07	-0.8452708	25	1.25	SPAST	Cytoskeleton
DMR2:33990001	2	33990001	2000	1	4.94E-06	-0.8583939	21	1.05	LINC01317	
DMR2:68449001	2	68449001	3000	3	8.43E-13	-2.8620153	10	0.3333333	WDR4P2;FBXO48	
DMR2:76430001	2	76430001	1000	1	7.20E-07	0.6682492	11	1.1		
DMR2:78762001	2	78762001	2000	1	9.72E-07	0.7961683	27	1.35		
DMR2:84291001	2	84291001	1000	1	2.07E-07	0.8794042	8	0.8	LOC107985905;FUNDC2P2	
DMR2:84445001	2	84445001	1000	1	9.90E-06	0.4795191	12	1.2	SUCLG1	Metabolism
DMR2:89264001	2	89264001	2000	1	2.18E-10	-2.3521462	7	0.35	IGK;IGKV1-32;IGKV1-33;IGKV3-34	Immune
DMR2:92270001	2	92270001	1000	1	3.04E-07	-0.6238143	14	1.4		
DMR2:96217001	2	96217001	2000	1	8.21E-06	0.6679678	37	1.85	STARD7;STARD7-AS1;SNORA112	
DMR2:96804001	2	96804001	1000	1	4.38E-07	-0.568276	25	2.5	CNNM4;MIR3127;CNNM3-DT;CNNM3	
DMR2:101449001	2	101449001	1000	1	4.53E-08	0.5996853	7	0.7	RFX8	Transcription
DMR2:102502001	2	102502001	1000	1	3.92E-06	0.7012213	19	1.9	SLC9A4	Transport
DMR2:114275001	2	114275001	2000	1	4.22E-07	-0.6336604	6	0.3		
DMR2:116828001	2	116828001	2000	1	6.38E-06	-0.8827497	30	1.5	LOC100533709	
DMR2:121107001	2	121107001	1000	1	6.62E-07	-1.1661723	15	1.5		
DMR2:121236001	2	121236001	1000	1	2.51E-07	0.7040426	25	2.5	TFCP2L1	Transcription
DMR2:127248001	2	127248001	1000	1	1.13E-06	0.6233198	18	1.8	WBP11P2;ERCC3	Epigenetic
DMR2:127569001	2	127569001	1000	1	3.74E-06	-0.8701337	13	1.3	MYO7B;LOC105373609	Cytoskeleton
DMR2:136345001	2	136345001	1000	1	2.39E-08	-1.4422665	8	0.8		
DMR2:150024001	2	150024001	1000	1	2.11E-11	-2.627824	0	0		
DMR2:151376001	2	151376001	1000	1	2.91E-08	-0.7734676	9	0.9	LOC101929319;TNFAIP6;MIR4773-2;MIR4773-1;RN7SL124P	
DMR2:152707001	2	152707001	1000	1	3.21E-06	0.6640097	14	1.4	PRPF40A;ARL6IP6	
DMR2:155263001	2	155263001	1000	1	4.56E-06	0.4934873	20	2	MTCO1P45;LOC105373698;MTND2P20;ATP5F1AP2	
DMR2:168975001	2	168975001	1000	1	2.83E-10	-2.691055	0	0	ABCB11	Transport
DMR2:169465001	2	169465001	1000	1	1.31E-14	-2.8406413	3	0.3		
DMR2:171239001	2	171239001	1000	1	4.23E-06	-0.7012784	12	1.2	TLK1;LOC107983995	Signaling
DMR2:172856001	2	172856001	2000	1	3.47E-06	-0.5482072	19	0.95	RAPGEF4	Transcription
DMR2:173784001	2	173784001	1000	1	7.89E-08	0.8737926	12	1.2		
DMR2:178607001	2	178607001	1000	1	9.45E-06	0.6707831	13	1.3	TTN-AS1;TTN	
DMR2:181288001	2	181288001	1000	1	1.16E-10	-2.4679959	4	0.4	LINC01934	
DMR2:185959001	2	185959001	1000	1	3.35E-06	-0.8364748	4	0.4	LOC107985783;RPL21P32	
DMR2:196603001	2	196603001	1000	1	1.19E-07	-0.6703793	11	1.1	HECW2	Proteolysis
DMR2:197027001	2	197027001	1000	1	7.80E-06	0.666746	12	1.2	ANKRD44;RPL4P7	Cytoskeleton
DMR2:205380001	2	205380001	2000	1	6.63E-12	-2.4753666	9	0.45	PARD3B	
DMR2:216010001	2	216010001	1000	1	1.66E-07	-0.5393645	33	3.3	MREG	
DMR2:217215001	2	217215001	1000	1	9.00E-06	-0.6217013	4	0.4	LOC101928278;LOC105373872	
DMR2:221381001	2	221381001	1000	1	1.40E-08	-1.083634	20	2		
DMR2:233788001	2	233788001	1000	1	2.26E-06	-0.6707692	23	2.3	MROH2A	
DMR2:238236001	2	238236001	1000	1	8.47E-06	0.7027402	16	1.6	LINC02610;TARDBPP3;HES6;PER2	Transcription;Transcription
DMR2:239386001	2	239386001	1000	1	1.73E-06	0.5588886	28	2.8	HDAC4	
DMR3:2705001	3	2705001	1000	1	2.21E-07	-0.898149	13	1.3	CNTN4	Cytoskeleton
DMR3:5716001	3	5716001	1000	1	4.55E-07	0.9270811	8	0.8		
DMR3:5960001	3	5960001	1000	1	5.61E-06	-0.586564	16	1.6	LOC102723596	
DMR3:13702001	3	13702001	8000	1	5.90E-07	0.6176207	70	0.875	LINC00620	
DMR3:18826001	3	18826001	2000	1	3.86E-12	-2.7682894	18	0.9		
DMR3:24254001	3	24254001	1000	1	2.01E-06	-0.6400569	10	1	THRB	Transcription
DMR3:26160001	3	26160001	5000	1	1.76E-06	0.4600473	57	1.14	LOC105377002	

DMR3:31740001	3	31740001	1000	1	4.08E-06	-0.8732868	6	0.6	OSBPL10	
DMR3:31965001	3	31965001	1000	1	9.33E-08	-1.0792757	11	1.1	OSBPL10	
DMR3:32114001	3	32114001	2000	1	8.48E-07	0.7703629	33	1.65	GPD1L	Metabolism
DMR3:40713001	3	40713001	2000	1	7.27E-06	0.7056903	21	1.05	LOC105377043	
DMR3:48486001	3	48486001	1000	1	3.48E-06	-0.7015057	10	1	SHISA5	Cytoskeleton
DMR3:49967001	3	49967001	2000	1	3.68E-06	0.60676	39	1.95	RBM6	
DMR3:56255001	3	56255001	1000	1	9.31E-06	0.7166154	9	0.9	ERC2	Transport
DMR3:56967001	3	56967001	1000	1	1.98E-07	-1.0931043	8	0.8	ARHGEF3;ARHGEF3-AS1	Transcription
DMR3:57554001	3	57554001	1000	1	7.66E-06	-0.4978339	18	1.8	DNAH12;RNU6-483P;PDE12	Translation
DMR3:58374001	3	58374001	1000	1	3.66E-06	-0.5001869	13	1.3	PXK	
DMR3:58386001	3	58386001	1000	1	4.32E-07	-0.9212266	6	0.6	PXK	
DMR3:72682001	3	72682001	1000	1	9.91E-07	0.9340719	7	0.7	LOC105377161;RNA5SP136	
DMR3:84188001	3	84188001	3000	1	2.62E-06	0.6505961	26	0.8666667		
DMR3:85769001	3	85769001	1000	1	2.11E-07	-0.9772116	10	1	CADM2	
DMR3:91543001	3	91543001	11000	2	1.64E-11	-0.5840603	169	1.5363636		
DMR3:91617001	3	91617001	4000	1	1.92E-06	-0.8319439	67	1.675		
DMR3:92383001	3	92383001	2000	1	4.75E-07	-0.6380201	34	1.7		
DMR3:93470001	3	93470001	1000	1	6.27E-09	-0.7813684	8	0.8		
DMR3:93705001	3	93705001	9000	7	1.76E-08	-0.5111597	153	1.7		
DMR3:95784001	3	95784001	1000	1	5.33E-14	-2.5608631	18	1.8		
DMR3:107391001	3	107391001	1000	1	1.94E-06	0.8259271	6	0.6	LOC101929579	
DMR3:113666001	3	113666001	1000	1	1.30E-06	-0.586106	23	2.3	USF3	
DMR3:121646001	3	121646001	1000	1	2.44E-07	-1.3766823	0	0	HCLS1;RN7SL172P;RNU4-62P	Cytoskeleton
DMR3:125027001	3	125027001	1000	1	1.96E-06	0.7433766	9	0.9	HEG1	
DMR3:136405001	3	136405001	1000	1	8.42E-06	-0.7240622	11	1.1	STAG1	Epigenetic
DMR3:142790001	3	142790001	1000	1	4.50E-06	0.6457768	10	1	TRPC1	Transport
DMR3:161287001	3	161287001	1000	1	4.70E-06	-0.90477	5	0.5	LOC105374187;LOC112268450	
DMR3:167183001	3	167183001	1000	1	2.49E-06	0.9290198	15	1.5	ZBBX	
DMR3:170377001	3	170377001	1000	1	8.62E-06	-0.4900879	17	1.7	SKIL	
DMR3:171357001	3	171357001	3000	1	3.78E-06	0.887585	33	1.1	TNIK	Signaling
DMR3:174564001	3	174564001	1000	1	2.03E-06	0.6870357	6	0.6	NAALADL2	Protease
DMR3:179387001	3	179387001	1000	1	6.14E-06	-0.5189522	19	1.9	MFN1;GNB4	Signaling
DMR3:184210001	3	184210001	1000	1	4.48E-06	-0.5117184	16	1.6		
DMR3:193888001	3	193888001	2000	1	7.35E-06	-0.709754	27	1.35	LOC105374287;LOC105374286	
DMR3:194840001	3	194840001	1000	1	7.61E-06	0.5744531	18	1.8	LOC107986174;LOC107986173	
DMR3:196161001	3	196161001	1000	1	6.80E-06	-0.6468694	15	1.5	LINC00885	
DMR3:197033001	3	197033001	3000	1	3.78E-06	0.7888561	25	0.8333333	MELTF;DLG1	Transport;Cytoskeleton
DMR4:5316001	4	5316001	1000	1	7.36E-17	-2.3671255	1	0.1	STK32B	Signaling
DMR4:12971001	4	12971001	1000	1	1.52E-07	-0.8807225	5	0.5	LOC105374493	
DMR4:14923001	4	14923001	1000	1	5.69E-12	-2.6108727	0	0	CPEB2-DT	
DMR4:15670001	4	15670001	1000	1	1.45E-06	-0.8958433	8	0.8	FBXL5;FAM200B	
DMR4:19226001	4	19226001	1000	1	7.56E-08	-1.1995387	16	1.6	LINC02438	
DMR4:23844001	4	23844001	1000	1	3.91E-10	-1.8364146	2	0.2	PPARGC1A	Transcription
DMR4:29898001	4	29898001	1000	1	3.00E-06	0.7352125	6	0.6		
DMR4:30499001	4	30499001	1000	1	7.23E-06	0.9397294	4	0.4		
DMR4:40554001	4	40554001	1000	1	5.25E-06	-0.5053218	20	2	RBM47	Metabolism
DMR4:49566001	4	49566001	1000	1	5.17E-06	-0.864795	4	0.4	SNX18P24	
DMR4:49601001	4	49601001	1000	1	1.40E-06	-0.477403	1	0.1		
DMR4:49709001	4	49709001	4000	3	2.90E-07	-0.7810933	23	0.575		
DMR4:51107001	4	51107001	1000	1	5.25E-08	-0.8247157	7	0.7		
DMR4:58293001	4	58293001	3000	1	4.80E-06	0.4604368	29	0.9666667		
DMR4:71685001	4	71685001	1000	1	9.60E-09	-1.7558827	0	0		
DMR4:73412001	4	73412001	1000	1	5.88E-06	0.7298991	13	1.3	ALB	Transport
DMR4:73543001	4	73543001	1000	1	6.89E-06	-1.0336829	17	1.7	LOC107986287	
DMR4:74560001	4	74560001	1000	1	2.73E-07	-1.8944002	1	0.1	LOC107986229	
DMR4:75627001	4	75627001	3000	1	2.52E-07	-0.6289895	64	2.1333333	CDKL2	Signaling
DMR4:78535001	4	78535001	1000	1	3.33E-06	0.6284776	11	1.1	FRAS1	
DMR4:80799001	4	80799001	1000	1	1.87E-10	-2.767917	0	0	CFAP299	Development
DMR4:81594001	4	81594001	1000	1	2.74E-06	-0.5552168	4	0.4		
DMR4:82877001	4	82877001	1000	1	7.38E-06	-0.7088425	18	1.8	SEC31A	Transport
DMR4:92388001	4	92388001	1000	1	3.09E-06	1.0036129	11	1.1	GRID2	Receptor
DMR4:99014001	4	99014001	3000	1	4.86E-06	0.7857449	44	1.4666667	METAP1;ABT1P1	Protease
DMR4:100645001	4	100645001	1000	1	1.37E-06	-0.9538675	2	0.2		
DMR4:108066001	4	108066001	1000	1	7.77E-07	0.7740955	15	1.5	LEF1	Transcription
DMR4:109753001	4	109753001	1000	1	1.99E-14	-2.1052638	0	0	CFI	Protease
DMR4:110233001	4	110233001	1000	1	5.98E-06	0.4730832	11	1.1		

DMR4:112573001	4	112573001	1000	1	6.25E-07	-0.8367149	6	0.6	ZGRF1;H3P14	
DMR4:114640001	4	114640001	1000	1	1.95E-06	-0.5867455	26	2.6	UGT8	
DMR4:130010001	4	130010001	2000	1	4.24E-11	-1.1450611	21	1.05		
DMR4:136374001	4	136374001	1000	1	7.97E-08	-0.7671404	15	1.5		
DMR4:140287001	4	140287001	2000	1	2.32E-06	0.7609392	9	0.45	SCOC;SCOC-AS1	
DMR4:142132001	4	142132001	3000	1	6.43E-06	0.594078	21	0.7	INPP4B	
DMR4:143271001	4	143271001	3000	1	2.51E-06	0.6411542	29	0.9666667		
DMR4:149557001	4	149557001	2000	1	4.07E-07	0.7019471	13	0.65	IQCM	
DMR4:150489001	4	150489001	1000	1	1.63E-12	-2.2844874	3	0.3	LRBA	
DMR4:154371001	4	154371001	1000	1	3.18E-06	0.7182121	9	0.9	DCHS2;LOC100419960	Cytoskeleton
DMR4:155463001	4	155463001	1000	1	7.02E-06	0.6120865	9	0.9	MTND6P17;MTND5P9;MTND4P8;MTND4LP29;MTND3P3;MTCO3P9;MTATP6P9;MTCO2P9;MTCO1P9;MTND2P33;NMTRQ-TTG15-1;MTND1P22	
DMR4:157275001	4	157275001	1000	1	1.91E-07	1.0087253	8	0.8	GRIA2	Receptor
DMR4:157625001	4	157625001	1000	1	2.77E-06	-0.9046871	3	0.3		
DMR4:168155001	4	168155001	2000	2	2.99E-11	-1.4734666	12	0.6	ANXA10	Signaling
DMR4:179725001	4	179725001	1000	1	2.35E-06	0.386223	14	1.4		
DMR4:182792001	4	182792001	1000	1	6.41E-08	0.7131351	23	2.3	TENM3	
DMR4:186202001	4	186202001	1000	1	8.30E-06	0.6249061	17	1.7	CYP4V2	Metabolism
DMR4:188829001	4	188829001	1000	1	9.04E-06	0.6421695	20	2		
DMR4:189048001	4	189048001	1000	1	3.32E-06	-0.5899505	26	2.6	LOC105377611;LOC105377612	
DMR5:344001	5	344001	3000	1	6.39E-07	-0.663702	117	3.9	PDCD6-AHRR;AHRR	Transcription
DMR5:2226001	5	2226001	1000	1	2.21E-17	-2.4447451	2	0.2		
DMR5:3508001	5	3508001	1000	1	8.91E-07	-0.7283063	6	0.6	LINC01019;LINC01017	
DMR5:14518001	5	14518001	1000	1	2.04E-06	0.6874811	15	1.5	TRIO	Transcription
DMR5:28619001	5	28619001	1000	1	8.13E-16	-2.5945209	3	0.3	RNU6-909P	
DMR5:34606001	5	34606001	1000	1	1.12E-06	-0.554518	16	1.6		
DMR5:45373001	5	45373001	2000	1	4.02E-06	-1.0327841	25	1.25	HCN1	Transport
DMR5:48850001	5	48850001	1000	1	5.64E-07	-0.8364243	17	1.7		
DMR5:49609001	5	49609001	10000	8	1.13E-08	-0.4273423	156	1.56		
DMR5:49621001	5	49621001	7000	5	8.88E-07	-0.4421726	117	1.6714286		
DMR5:49641001	5	49641001	9000	4	1.71E-07	-0.4231424	136	1.5111111		
DMR5:50645001	5	50645001	1000	1	1.57E-09	-2.4284714	1	0.1		
DMR5:56025001	5	56025001	2000	1	9.74E-06	-0.5295295	36	1.8		
DMR5:68130001	5	68130001	1000	1	2.33E-11	-0.8313573	11	1.1		
DMR5:69402001	5	69402001	1000	1	3.29E-07	-0.8178446	23	2.3	RAD17	
DMR5:70435001	5	70435001	1000	1	6.59E-06	-0.5590011	23	2.3	LOC107986356;GTF2H2B	
DMR5:70954001	5	70954001	2000	1	2.83E-07	-0.5167123	31	1.55	SMN1	
DMR5:74548001	5	74548001	1000	1	8.53E-06	0.5588735	12	1.2		
DMR5:78278001	5	78278001	2000	1	8.44E-06	1.2938871	34	1.7	AP3B1	Transport
DMR5:84248001	5	84248001	1000	1	8.81E-06	0.9015556	6	0.6	EDIL3	Metabolism
DMR5:94995001	5	94995001	1000	1	3.74E-06	-1.3062676	7	0.7	MCTP1	
DMR5:100493001	5	100493001	1000	1	8.09E-12	-1.9606609	3	0.3		
DMR5:102827001	5	102827001	1000	1	3.44E-08	-0.965169	17	1.7	PAM	Metabolism
DMR5:106075001	5	106075001	1000	1	8.66E-15	-2.5872609	0	0		
DMR5:113056001	5	113056001	1000	1	1.57E-06	0.7271778	10	1	MCC	
DMR5:120438001	5	120438001	1000	1	9.89E-06	-0.5727955	13	1.3		
DMR5:126720001	5	126720001	2000	1	1.73E-08	-0.6912109	32	1.6		
DMR5:128688001	5	128688001	1000	1	3.87E-06	-0.6557852	7	0.7	LOC105379168	
DMR5:133625001	5	133625001	1000	1	8.64E-07	-1.2609574	0	0	FSTL4	Protease; Proteolysis
DMR5:135588001	5	135588001	1000	1	4.91E-06	0.5384471	15	1.5	CXCL14;SLC25A48	Growth Factors;Transport
DMR5:138019001	5	138019001	1000	1	2.55E-06	-0.7034619	15	1.5	FAM13B	
DMR5:138108001	5	138108001	1000	1	2.25E-06	-0.537443	32	3.2	NME5;RNU6-460P	Signaling
DMR5:138562001	5	138562001	1000	1	7.78E-06	-0.6499723	23	2.3	HSPA9;SNORD63B;SNORD63;LOC105379193	
DMR5:144355001	5	144355001	1000	1	3.19E-06	0.7908621	6	0.6	KCTD16	Cytoskeleton
DMR5:149650001	5	149650001	2000	2	5.76E-19	-2.9454014	2	0.1		
DMR5:154878001	5	154878001	3000	1	7.33E-06	0.7840745	43	1.4333333	CNOT8;GEMIN5	Translation
DMR5:172275001	5	172275001	1000	1	8.72E-06	0.6969135	13	1.3	UBTD2;LOC100288254	Proteolysis
DMR5:174531001	5	174531001	1000	1	5.03E-06	0.6801863	10	1	LINC01411;LOC105377739	
DMR5:175548001	5	175548001	1000	1	3.26E-06	-0.7543954	7	0.7		
DMR5:176743001	5	176743001	1000	1	5.27E-07	-0.9892913	60	6	LINC01574	
DMR5:179061001	5	179061001	1000	1	2.18E-06	0.5812889	35	3.5	ZNF354C	
DMR6:361001	6	361001	7000	1	3.47E-06	0.5844737	144	2.0571429	DUSP22	Signaling
DMR6:906001	6	906001	1000	1	3.00E-13	-1.9912835	2	0.2		
DMR6:4266001	6	4266001	1000	1	2.61E-07	0.867628	6	0.6		

DMR6:6088001	6	6088001	1000	1	3.75E-06	0.6113316	7	0.7		
DMR6:7024001	6	7024001	1000	1	1.11E-07	-0.9227698	8	0.8		
DMR6:7750001	6	7750001	1000	1	5.46E-06	0.7711126	10	1	BMP6	Growth Factors
DMR6:10751001	6	10751001	1000	1	2.13E-07	1.0136724	19	1.9	TMEM14B;RNA5SP203	
DMR6:11103001	6	11103001	2000	1	4.24E-07	0.770324	41	2.05	SMIM13;ERVFRD-1	Epigenetic
DMR6:12761001	6	12761001	1000	1	3.36E-06	0.8609334	8	0.8	PHACTR1	Signaling
DMR6:18583001	6	18583001	2000	1	1.73E-06	0.8410887	14	0.7	MIR548A1HG	
DMR6:28118001	6	28118001	2000	1	2.95E-08	-0.8533927	12	0.6	ZNF602P;ZSCAN16-AS1;ZSCAN16	Transcription
DMR6:28755001	6	28755001	1000	1	1.01E-06	-0.7965747	6	0.6	TRK-TTT7-1;TRA-TGC6-1	
DMR6:30869001	6	30869001	2000	1	2.31E-06	-0.8195059	25	1.25	RN7SKP186;DDR1	Receptor
DMR6:31532001	6	31532001	1000	1	4.63E-06	0.8126663	15	1.5	RPL15P4;MCCD1;ATP6V1G2-DDX39B;DDX39B;SNORD117;SNORD84;DDX39B-AS1	
DMR6:71880001	6	71880001	1000	1	1.07E-06	0.6075037	8	0.8	RIMS1	Transport
DMR6:75655001	6	75655001	1000	1	5.86E-06	0.8071917	12	1.2	SENP6;RN7SKP163	Protease
DMR6:77590001	6	77590001	2000	2	2.34E-16	-2.2046667	4	0.2		
DMR6:88716001	6	88716001	1000	1	2.20E-08	-1.1156101	5	0.5	RNGTT	Translation
DMR6:101760001	6	101760001	1000	1	1.29E-11	-1.2036632	13	1.3	GRIK2	Receptor
DMR6:105767001	6	105767001	2000	1	9.88E-07	0.6248582	10	0.5	LOC105377923	
DMR6:116082001	6	116082001	1000	1	2.70E-06	0.7797771	3	0.3	FRK	
DMR6:120406001	6	120406001	1000	1	7.48E-07	1.0336374	11	1.1		
DMR6:123266001	6	123266001	1000	1	1.89E-10	-1.734609	4	0.4	TRDN	
DMR6:135563001	6	135563001	1000	1	7.49E-06	0.6192336	8	0.8	LINC00271	
DMR6:142753001	6	142753001	1000	1	4.45E-06	0.7263915	19	1.9	HIVEP2	
DMR6:142910001	6	142910001	1000	1	3.23E-06	0.656697	11	1.1	HIVEP2	
DMR6:148334001	6	148334001	1000	1	5.82E-08	-0.4632855	43	4.3	SASH1	
DMR6:149557001	6	149557001	1000	1	7.51E-06	-0.7125897	9	0.9	GINM1	
DMR6:158169001	6	158169001	1000	1	8.27E-11	-1.1431703	19	1.9	SERAC1;GTF2H5	Transcription
DMR6:158443001	6	158443001	3000	1	5.16E-07	-0.6346653	36	1.2	TULP4;RN7SL173P	
DMR6:159010001	6	159010001	2000	1	2.45E-07	0.9196286	23	1.15	RSPH3;TAGAP-AS1	Development
DMR6:160100001	6	160100001	1000	1	7.58E-09	-0.6510164	5	0.5	IGF2R;CHP1P2	Transport
DMR6:164016001	6	164016001	1000	1	4.62E-06	0.6010827	10	1	LOC105378102	
DMR6:166760001	6	166760001	1000	1	1.62E-06	0.7088645	18	1.8	RPS6KA2	Golgi
DMR6:168012001	6	168012001	2000	1	1.67E-07	1.1281038	21	1.05	KIF25	Cytoskeleton
DMR6:169637001	6	169637001	1000	1	3.30E-06	0.4814209	23	2.3	WDR27	
DMR7:854001	7	854001	5000	1	6.49E-06	0.7051095	168	3.36	SUN1	Cytoskeleton
DMR7:6192001	7	6192001	1000	1	2.00E-06	-0.6436597	14	1.4	CYTH3	Transcription
DMR7:11180001	7	11180001	1000	1	2.95E-11	-2.1700038	2	0.2		
DMR7:11771001	7	11771001	1000	1	6.81E-06	1.017153	8	0.8	THSD7A	Cytoskeleton
DMR7:29748001	7	29748001	2000	1	2.82E-06	0.6250416	13	0.65	DPY19L2P3	
DMR7:45771001	7	45771001	2000	1	2.54E-06	-0.6645411	31	1.55	SEPTIN7P2;GTF2IP13	
DMR7:51392001	7	51392001	1000	1	4.04E-08	0.8591439	12	1.2	CICP17;LOC101928675;LOC100133177;LOC107986795;LOC107986794;LOC102723533	
DMR7:53538001	7	53538001	1000	1	6.90E-07	0.7299675	8	0.8		
DMR7:63039001	7	63039001	1000	1	2.82E-06	0.7395757	12	1.2		
DMR7:70402001	7	70402001	1000	1	2.06E-07	1.0268656	11	1.1	AUTS2	
DMR7:72458001	7	72458001	1000	1	4.28E-16	-2.2792347	2	0.2	CALN1	
DMR7:73589001	7	73589001	1000	1	3.90E-26	-2.2840516	7	0.7	MLXIPL	Transcription
DMR7:76265001	7	76265001	2000	1	3.59E-08	-0.9515498	46	2.3	SRRM3	
DMR7:76860001	7	76860001	1000	1	2.79E-06	0.806085	11	1.1		
DMR7:92851001	7	92851001	2000	1	4.64E-06	0.5201339	15	0.75	CDK6-AS1	
DMR7:93719001	7	93719001	1000	1	1.55E-06	-0.8446881	5	0.5	MIR4652	
DMR7:94193001	7	94193001	1000	1	8.81E-06	-1.064401	3	0.3		
DMR7:118703001	7	118703001	2000	1	7.38E-06	-0.491667	29	1.45		
DMR7:119088001	7	119088001	1000	1	8.05E-07	-0.8000713	1	0.1		
DMR7:120055001	7	120055001	1000	1	2.25E-06	0.9686748	10	1		
DMR7:121740001	7	121740001	1000	1	1.96E-06	-1.0165192	5	0.5	RN7SKP277	
DMR7:124047001	7	124047001	1000	1	4.17E-09	-0.8842033	6	0.6		
DMR7:124505001	7	124505001	1000	1	1.77E-07	-1.1792086	5	0.5		
DMR7:133234001	7	133234001	1000	1	2.50E-06	-0.6824997	15	1.5		
DMR7:135239001	7	135239001	1000	1	1.35E-06	1.022884	11	1.1	STRA8	
DMR7:153282001	7	153282001	1000	1	7.00E-09	-0.6631625	19	1.9	LOC102723686	
DMR7:153471001	7	153471001	1000	1	6.47E-06	0.5210372	19	1.9		
DMR7:154858001	7	154858001	1000	1	9.38E-06	0.6702812	25	2.5	DPP6;LOC105375580	Protease
DMR7:154902001	7	154902001	1000	1	2.97E-07	1.0946529	12	1.2	DPP6	Protease

DMR7:155050001	7	155050001	1000	1	5.84E-09	-0.9905806	8	0.8		
DMR7:156599001	7	156599001	4000	1	6.65E-06	0.8224615	56	1.4	LINC01006	
DMR7:158085001	7	158085001	2000	1	8.03E-06	0.5881423	79	3.95	PTPRN2	Signaling
DMR7:158245001	7	158245001	5000	1	3.34E-06	0.438529	152	3.04	PTPRN2	Signaling
DMR7:159004001	7	159004001	1000	1	1.01E-06	0.7054979	19	1.9	LINC00689	
DMR8:923001	8	923001	3000	1	6.17E-06	0.7868389	57	1.9	DLGAP2	Cytoskeleton
DMR8:982001	8	982001	1000	1	2.15E-07	0.9643435	13	1.3	DLGAP2	Cytoskeleton
DMR8:5122001	8	5122001	1000	1	1.03E-06	0.538273	64	6.4	LOC107986907	
DMR8:6114001	8	6114001	1000	1	3.01E-13	-2.4138534	0	0		
DMR8:10355001	8	10355001	1000	1	2.24E-06	0.9032793	13	1.3	MSRA	Metabolism
DMR8:12560001	8	12560001	1000	1	1.99E-07	-0.7135558	43	4.3	LOC100506990;LOC729732;RPS3AP34	
DMR8:12927001	8	12927001	3000	1	5.36E-06	0.8314336	31	1.0333333		
DMR8:13217001	8	13217001	3000	2	1.04E-09	-1.4940507	30	1	DLC1	Signaling
DMR8:16189001	8	16189001	1000	1	4.91E-10	-1.8163265	4	0.4	MSR1	Protease
DMR8:17239001	8	17239001	1000	1	1.41E-06	0.5904471	18	1.8	ZDHHC2;CNOT7;VPS37A	Translation
DMR8:17882001	8	17882001	2000	1	2.29E-07	-1.1275905	11	0.55	FGL1	Signaling
DMR8:19222001	8	19222001	1000	1	5.90E-07	0.5638323	10	1	LOC100128993	
DMR8:20084001	8	20084001	1000	1	1.82E-06	0.683099	13	1.3	LOC105379311	
DMR8:24796001	8	24796001	1000	1	9.69E-06	0.6256172	5	0.5		
DMR8:26244001	8	26244001	2000	2	6.83E-09	-1.9734336	14	0.7	LOC100129404	
DMR8:28099001	8	28099001	2000	1	9.65E-06	0.6466198	20	1	ELP3	Epigenetic
DMR8:33319001	8	33319001	1000	1	2.00E-11	-2.9368354	0	0	FUT10	Golgi
DMR8:38073001	8	38073001	3000	2	6.54E-11	-2.5390354	22	0.7333333		
DMR8:38322001	8	38322001	1000	1	7.03E-07	0.9459028	7	0.7	NSD3	
DMR8:41607001	8	41607001	3000	1	3.10E-07	0.8894614	48	1.6	GPAT4	Metabolism
DMR8:43237001	8	43237001	5000	5	1.46E-09	-1.1171127	15	0.3	LOC101059977;AFG3L2P1	
DMR8:60465001	8	60465001	3000	1	4.78E-06	0.9525877	35	1.1666667		
DMR8:65326001	8	65326001	1000	1	1.98E-06	0.6705898	14	1.4		
DMR8:80427001	8	80427001	2000	1	7.36E-07	-0.9962764	24	1.2		
DMR8:85384001	8	85384001	1000	1	8.48E-06	-0.8771785	4	0.4	CA1	
DMR8:93644001	8	93644001	1000	1	4.58E-10	-1.6674255	3	0.3	CIBAR1-DT;ZNF317P1	
DMR8:100052001	8	100052001	1000	1	6.66E-08	-0.7672867	18	1.8	RGS22	
DMR8:100506001	8	100506001	1000	1	4.39E-07	0.5828283	13	1.3	LOC105375670;ANKRD46	
DMR8:104338001	8	104338001	1000	1	3.69E-06	0.7757366	6	0.6	DCSTAMP	
DMR8:111156001	8	111156001	2000	1	6.07E-07	-0.7001264	32	1.6	LINC01609	
DMR8:117097001	8	117097001	1000	1	1.67E-08	-0.9425049	13	1.3	SLC30A8;LOC105375716;LOC107986969;LOC105375719	Transport
DMR8:124302001	8	124302001	1000	1	1.24E-17	-1.6046953	5	0.5	LOC112268031;TMEM65	
DMR8:124945001	8	124945001	2000	1	5.23E-07	0.9280962	12	0.6	LOC105375742;LINC00964	
DMR8:128512001	8	128512001	1000	1	3.01E-06	0.659362	7	0.7	LINC00824	
DMR8:129495001	8	129495001	3000	1	8.82E-07	-0.9312666	98	3.2666667	CCDC26	
DMR8:134895001	8	134895001	2000	1	2.23E-06	0.7586151	20	1		
DMR8:141629001	8	141629001	3000	1	7.36E-08	0.5512813	55	1.8333333		
DMR8:144619001	8	144619001	2000	1	4.32E-06	0.4617195	98	4.9	ARHGAP39	
DMR9:869001	9	869001	1000	1	3.74E-07	0.747618	13	1.3	DMRT1	Transcription
DMR9:2996001	9	2996001	1000	1	9.10E-06	0.820686	7	0.7	CARM1P1	
DMR9:3780001	9	3780001	1000	1	2.06E-06	-0.5206321	44	4.4		
DMR9:4777001	9	4777001	2000	1	3.64E-06	-0.5842681	21	1.05	ECM1P1;RPS5P6	
DMR9:6405001	9	6405001	1000	1	5.14E-06	-0.935074	7	0.7	UHRF2	Proteolysis
DMR9:6423001	9	6423001	1000	1	9.52E-07	-0.9002324	11	1.1	UHRF2	Proteolysis
DMR9:13199001	9	13199001	2000	1	8.59E-06	1.0458544	13	0.65	MPDZ	
DMR9:16907001	9	16907001	1000	1	1.50E-06	-1.0826159	11	1.1		
DMR9:17477001	9	17477001	1000	1	9.29E-06	0.9388158	6	0.6	CNTLN	
DMR9:18076001	9	18076001	2000	1	6.26E-06	0.5698225	16	0.8	ADAMTSL1	Protease
DMR9:25309001	9	25309001	1000	1	6.65E-06	-0.6268498	15	1.5		
DMR9:32360001	9	32360001	1000	1	7.44E-17	-2.7271562	2	0.2	LOC107987059	
DMR9:34155001	9	34155001	1000	1	4.82E-06	-0.7047945	11	1.1		
DMR9:35781001	9	35781001	1000	1	5.05E-06	-0.6715821	6	0.6	NPR2	Signaling
DMR9:38425001	9	38425001	1000	1	3.58E-06	1.1957113	12	1.2	IGFBPL1;LOC105376041	
DMR9:41010001	9	41010001	1000	1	2.73E-07	-1.2414573	4	0.4	FRG1HP;PGM5P2	
DMR9:41607001	9	41607001	1000	1	1.06E-06	-0.7096952	9	0.9		
DMR9:42029001	9	42029001	1000	1	9.33E-06	-0.7615736	10	1	CNTNAP3B	
DMR9:60555001	9	60555001	36000	4	8.26E-07	-0.6458579	463	1.2861111		
DMR9:60592001	9	60592001	52000	5	1.57E-06	-0.6811829	674	1.2961538		
DMR9:60652001	9	60652001	37000	2	1.29E-06	-0.7316325	462	1.2486486		
DMR9:69916001	9	69916001	1000	1	4.64E-06	0.5600422	6	0.6	C9orf135	

DMR9:69988001	9	69988001	1000	1	4.95E-06	-0.6597132	3	0.3		
DMR9:70982001	9	70982001	1000	1	5.13E-06	0.5133003	7	0.7	TRPM3	Transport
DMR9:72375001	9	72375001	1000	1	1.58E-06	-0.8943321	14	1.4	ZFAND5	
DMR9:74629001	9	74629001	1000	1	3.18E-06	0.8241841	7	0.7	RORB;LOC105376088	Transcription
DMR9:76921001	9	76921001	1000	1	9.98E-06	0.7953465	15	1.5		
DMR9:85780001	9	85780001	1000	1	5.57E-06	-0.9133583	8	0.8	LOC100419824;LOC102724057	
DMR9:88343001	9	88343001	1000	1	3.21E-06	-1.0777228	8	0.8		
DMR9:88697001	9	88697001	1000	1	4.19E-06	0.4597635	32	3.2		
DMR9:90216001	9	90216001	1000	1	2.03E-07	0.6440907	20	2	OR7E31P	
DMR9:94312001	9	94312001	2000	1	3.30E-07	-0.944888	25	1.25	ZNF169;LOC105376154;NUTM2F	Transcription
DMR9:94622001	9	94622001	1000	1	1.62E-07	0.7602111	20	2	FBP1	Metabolism
DMR9:97255001	9	97255001	1000	1	1.02E-06	-1.4828352	3	0.3	SUGT1P4;SUGT1P4-STRA6LP-CCDC180;SUGT1P4-STRA6LP	
DMR9:110690001	9	110690001	1000	1	9.80E-17	-2.4279623	2	0.2	MUSK	Receptor
DMR9:127577001	9	127577001	2000	1	9.79E-06	-0.5595623	38	1.9	NIBAN2	
DMR10:447001	10	447001	2000	1	2.72E-06	0.8085425	44	2.2	DIP2C	
DMR10:948001	10	948001	2000	1	1.38E-06	0.418496	58	2.9	LOC101927762;LOC107984285;LOC105376341	
DMR10:1210001	10	1210001	2000	1	2.31E-06	0.6432228	43	2.15	ADARB2	Metabolism
DMR10:3321001	10	3321001	1000	1	7.37E-06	0.6887195	18	1.8	LOC105376356;LOC105376360	
DMR10:3434001	10	3434001	1000	1	6.69E-06	0.5739506	15	1.5	LOC105376360;LINC02669	
DMR10:3962001	10	3962001	1000	1	2.74E-07	0.8785737	12	1.2		
DMR10:4637001	10	4637001	1000	1	4.29E-06	0.6498932	5	0.5		
DMR10:12694001	10	12694001	2000	1	2.19E-11	-1.6517434	23	1.15	CAMK1D	Signaling
DMR10:14832001	10	14832001	1000	1	6.18E-06	0.6098818	9	0.9	CDNF;HSPA14;LOC100421372	
DMR10:19238001	10	19238001	1000	1	1.72E-10	-1.4470155	2	0.2	MALRD1	
DMR10:19315001	10	19315001	1000	1	1.17E-12	-2.3455262	0	0	MALRD1	
DMR10:21985001	10	21985001	2000	1	3.45E-06	-0.677422	32	1.6	DNAJC1	Transcription
DMR10:24809001	10	24809001	1000	1	9.80E-06	-0.5695374	26	2.6		
DMR10:26292001	10	26292001	3000	1	7.52E-06	0.8010627	45	1.5	GAD2	Metabolism
DMR10:28336001	10	28336001	1000	1	1.59E-06	-0.7490934	11	1.1	LOC105376467;ZNF101P1	
DMR10:30503001	10	30503001	1000	1	4.60E-06	0.4875973	12	1.2		
DMR10:49487001	10	49487001	1000	1	1.42E-06	0.8028985	9	0.9	ERCC6	
DMR10:51126001	10	51126001	1000	1	9.47E-06	-0.8935285	1	0.1	PRKG1	Signaling
DMR10:53925001	10	53925001	1000	1	2.61E-06	0.4584647	23	2.3	PCDH15	Cytoskeleton
DMR10:62614001	10	62614001	1000	1	1.28E-06	0.8441718	11	1.1	LOC105378327	
DMR10:62809001	10	62809001	1000	1	3.67E-06	-0.8295538	9	0.9	ADO;EGR2	Transcription
DMR10:83792001	10	83792001	1000	1	9.54E-06	0.6767813	7	0.7		
DMR10:83915001	10	83915001	1000	1	3.71E-06	0.7932807	7	0.7	LOC107984181	
DMR10:84623001	10	84623001	1000	1	9.97E-07	1.1091721	6	0.6		
DMR10:95553001	10	95553001	2000	1	1.57E-06	-0.6080138	22	1.1	SORBS1	
DMR10:103220001	10	103220001	1000	1	3.69E-06	-1.0694072	1	0.1	ST13P13	
DMR10:104696001	10	104696001	1000	1	7.99E-12	-1.1863932	10	1	SORCS3	Transport
DMR10:104910001	10	104910001	1000	1	1.06E-06	0.8404749	12	1.2	SORCS3	Transport
DMR10:113529001	10	113529001	1000	1	5.27E-06	0.6727865	20	2		
DMR10:114138001	10	114138001	1000	1	4.90E-07	-0.5614322	9	0.9	CCDC186	
DMR10:126565001	10	126565001	3000	1	4.72E-07	-1.224053	34	1.1333333	C10orf90	
DMR10:133686001	10	133686001	1000	1	4.13E-06	-0.5459126	5	0.5	DUX4L22;DUX4L21;DUX4L20	
DMR11:4110001	11	4110001	1000	1	7.92E-07	-0.7855959	17	1.7	RRM1	Metabolism
DMR11:12774001	11	12774001	2000	1	1.61E-06	0.8295512	24	1.2	TEAD1	Transcription
DMR11:23433001	11	23433001	1000	1	8.23E-06	0.844336	6	0.6		
DMR11:25601001	11	25601001	1000	1	7.53E-06	0.6451793	11	1.1		
DMR11:26057001	11	26057001	2000	1	6.21E-14	-1.7924123	5	0.25	LOC100533706	
DMR11:36507001	11	36507001	1000	1	3.00E-10	-1.5075529	24	2.4	TRAF6;RAG1	Cytoskeleton;Transcription
DMR11:44596001	11	44596001	1000	1	8.65E-09	1.259168	16	1.6	CD82	
DMR11:47362001	11	47362001	1000	1	5.50E-06	-0.8630892	8	0.8	MYBPC3;SPI1	Transcription
DMR11:49312001	11	49312001	1000	1	6.72E-06	0.8531839	5	0.5	LOC729960	
DMR11:57757001	11	57757001	1000	1	7.01E-07	-1.011087	8	0.8	TMX2-CTNND1;BTBD18;CTNND1	Cytoskeleton
DMR11:60065001	11	60065001	1000	1	3.91E-06	0.5688318	6	0.6	MS4A3	Transport
DMR11:65072001	11	65072001	1000	1	5.85E-06	-1.07531	4	0.4	CDCA5	
DMR11:73251001	11	73251001	2000	1	1.88E-12	-1.8543741	26	1.3	P2RY2;OR8R1P;LOC105369382	Signaling
DMR11:74402001	11	74402001	1000	1	1.09E-06	-1.4265416	3	0.3	PGM2L1;MIR548AL	Metabolism
DMR11:76660001	11	76660001	2000	1	5.76E-06	0.6153172	62	3.1	LRRC32;LOC107984360	Receptor
DMR11:90097001	11	90097001	1000	1	6.52E-09	-1.6116456	2	0.2	UBTFL1	Transcription
DMR11:93649001	11	93649001	2000	1	3.44E-07	0.6025837	13	0.65		
DMR11:96445001	11	96445001	1000	1	5.03E-06	-0.6368614	6	0.6	JRKL-AS1	

DMR11:96555001	11	96555001	1000	1	5.01E-07	-1.0159922	17	1.7		
DMR11:96562001	11	96562001	1000	1	7.80E-09	-2.2415248	5	0.5		
DMR11:105748001	11	105748001	2000	1	4.65E-06	0.5227146	26	1.3	GRIA4	Receptor
DMR11:108934001	11	108934001	1000	1	7.95E-06	0.723681	8	0.8	DDX10	
DMR11:113517001	11	113517001	1000	1	1.49E-07	0.9909071	7	0.7		
DMR11:122017001	11	122017001	1000	1	4.31E-06	-1.2943671	7	0.7		
DMR11:128868001	11	128868001	1000	1	1.82E-07	-1.1125512	14	1.4	KCNJ1;LOC107984409	Transport
DMR11:132295001	11	132295001	2000	1	4.88E-06	0.5423371	19	0.95	NTM;NTM-IT	Immune
DMR11:133973001	11	133973001	1000	1	2.84E-07	0.6884753	21	2.1		
DMR12:5279001	12	5279001	3000	1	7.90E-09	1.2027946	29	0.9666667	LOC105369617	
DMR12:6133001	12	6133001	1000	1	7.17E-06	0.6077476	21	2.1	VWF	Extracellular Matrix
DMR12:6909001	12	6909001	1000	1	7.54E-06	-1.1072252	5	0.5	LRRC23;ENO2	Cytoskeleton;Metabolism
DMR12:8596001	12	8596001	2000	1	5.89E-06	0.9104524	24	1.2	AICDA	Translation
DMR12:13881001	12	13881001	1000	1	8.37E-06	0.3377119	16	1.6	GRIN2B	Receptor
DMR12:16361001	12	16361001	1000	1	3.77E-08	0.9007094	10	1	MGST1	Transport
DMR12:25580001	12	25580001	1000	1	5.33E-06	1.1355355	5	0.5	LMNTD1	
DMR12:29643001	12	29643001	1000	1	6.33E-10	-1.2978384	5	0.5	TMTC1	
DMR12:29653001	12	29653001	2000	1	3.57E-06	0.8952261	12	0.6	TMTC1;RPL21P99	
DMR12:30125001	12	30125001	1000	1	1.18E-06	0.5884705	8	0.8		
DMR12:34834001	12	34834001	2000	2	9.95E-09	-0.5649059	27	1.35		
DMR12:45649001	12	45649001	2000	1	4.08E-06	0.6078413	11	0.55		
DMR12:47173001	12	47173001	1000	1	1.37E-07	0.703797	17	1.7	PCED1B	
DMR12:49878001	12	49878001	3000	1	6.00E-08	0.5690106	27	0.9	FAIM2	
DMR12:57264001	12	57264001	1000	1	8.68E-07	-0.8071896	14	1.4	R3HDM2	
DMR12:57951001	12	57951001	1000	1	8.84E-06	0.6899503	9	0.9	ATP23	Protease
DMR12:60799001	12	60799001	1000	1	6.99E-07	1.1354309	4	0.4		
DMR12:63169001	12	63169001	1000	1	1.04E-06	-0.7900134	6	0.6		
DMR12:66057001	12	66057001	1000	1	5.60E-07	-0.5567645	9	0.9	RNA5SP362	
DMR12:79534001	12	79534001	1000	1	2.19E-06	0.735188	6	0.6		
DMR12:80397001	12	80397001	1000	1	8.36E-06	-0.5114554	14	1.4		
DMR12:81903001	12	81903001	1000	1	5.29E-06	0.5531228	7	0.7		
DMR12:88715001	12	88715001	1000	1	8.84E-07	-1.174192	3	0.3		
DMR12:91141001	12	91141001	1000	1	6.11E-06	0.6839774	8	0.8	DCN	
DMR12:94217001	12	94217001	1000	1	7.69E-06	0.6567774	4	0.4	PLXNC1	
DMR12:95570001	12	95570001	2000	1	7.88E-06	-0.5812294	21	1.05		
DMR12:100022001	12	100022001	2000	1	8.71E-06	0.5226194	18	0.9	UHRF1BP1L	
DMR12:110309001	12	110309001	2000	1	5.02E-06	-0.6034693	24	1.2	ATP2A2	Transport
DMR12:110709001	12	110709001	1000	1	6.39E-06	-0.5955101	13	1.3	HVCN1;PPP1CC	Signaling
DMR12:111055001	12	111055001	2000	1	2.27E-06	0.9550212	40	2	CUX2	Development
DMR12:120928001	12	120928001	1000	1	7.16E-18	-1.9378314	1	0.1		
DMR12:122632001	12	122632001	1000	1	1.78E-06	-0.6264137	17	1.7	KNTC1	
DMR12:127267001	12	127267001	1000	1	4.90E-06	0.4417167	16	1.6	LINC02376	
DMR12:128084001	12	128084001	2000	1	1.82E-10	-2.6404128	19	0.95	LOC105370071;LINC02369	
DMR12:128646001	12	128646001	3000	1	1.81E-06	0.5404576	47	1.5666667	TMEM132C	
DMR12:132165001	12	132165001	1000	1	1.11E-06	-1.1321833	6	0.6	LOC105370090	
DMR13:22868001	13	22868001	1000	1	3.69E-07	-2.0060674	4	0.4	LINC00621	
DMR13:23865001	13	23865001	3000	1	7.11E-06	0.6801954	38	1.2666667	MIPEP	Protease
DMR13:23994001	13	23994001	1000	1	2.71E-07	1.0838227	7	0.7	SPATA13	Transcription
DMR13:25159001	13	25159001	1000	1	1.20E-07	-1.2922874	6	0.6	AMER2	
DMR13:27935001	13	27935001	1000	1	3.55E-10	-1.4605432	10	1	PDX1;ATP5F1EP2	Metabolism
DMR13:27999001	13	27999001	1000	1	2.02E-07	-1.1277581	8	0.8	LOC105370132;RN7SL272P;FLT3	Receptor
DMR13:28797001	13	28797001	4000	1	5.60E-08	-0.734434	45	1.125		
DMR13:40214001	13	40214001	1000	1	5.24E-12	-1.9006639	2	0.2	LINC00548;RPL17P51	
DMR13:56314001	13	56314001	1000	1	8.34E-06	-1.2220887	4	0.4	LOC105370214	
DMR13:56377001	13	56377001	1000	1	1.55E-06	-0.902688	3	0.3	LOC105370214	
DMR13:56814001	13	56814001	1000	1	1.53E-06	-1.1002427	3	0.3		
DMR13:62688001	13	62688001	1000	1	1.20E-14	-2.5396086	1	0.1	LINC00448	
DMR13:64920001	13	64920001	2000	1	4.97E-06	0.6942486	9	0.45		
DMR13:65449001	13	65449001	1000	1	1.74E-08	-1.9886542	2	0.2		
DMR13:72372001	13	72372001	1000	1	1.62E-06	-0.8325619	3	0.3		
DMR13:85395001	13	85395001	1000	1	2.67E-08	-1.3894423	9	0.9	LINC00351	
DMR13:92031001	13	92031001	1000	1	2.70E-06	-0.5507507	6	0.6	GPC5	
DMR13:100611001	13	100611001	1000	1	5.14E-07	-0.6040021	18	1.8	TMTC4	
DMR13:103014001	13	103014001	1000	1	4.26E-06	0.7346816	8	0.8		
DMR13:109152001	13	109152001	1000	1	6.85E-07	0.8367615	15	1.5	MYO16;LOC105370356	
DMR14:16092001	14	16092001	14000	4	2.28E-09	-1.3965111	156	1.1142857		
DMR14:34349001	14	34349001	1000	1	9.36E-10	-1.8773184	6	0.6		

DMR14:41539001	14	41539001	1000	1	2.51E-06	-1.3404147	4	0.4		
DMR14:55489001	14	55489001	1000	1	5.33E-12	-1.3845586	9	0.9		
DMR14:58334001	14	58334001	2000	1	5.77E-06	0.7157455	17	0.85	ARID4A	Transcription
DMR14:60142001	14	60142001	2000	1	2.18E-06	1.0752103	19	0.95	PCNX4;DHRS7	Metabolism
DMR14:62258001	14	62258001	1000	1	9.96E-06	0.7375659	9	0.9		
DMR14:72335001	14	72335001	1000	1	8.15E-06	0.9309069	12	1.2	RGS6	
DMR14:76872001	14	76872001	1000	1	5.55E-06	-0.7992878	13	1.3	LRRC74A	
DMR14:77269001	14	77269001	1000	1	2.11E-06	-0.5089888	14	1.4	TMEM63C;NGB;MIR1260A;POMT2	Transport;Transport
DMR14:79995001	14	79995001	1000	1	4.17E-06	0.7384093	7	0.7	LOC105370590	
DMR14:81457001	14	81457001	3000	1	6.98E-07	-0.6541365	48	1.6	LINC02308	
DMR14:84854001	14	84854001	1000	1	2.28E-07	-1.029333	5	0.5		
DMR14:85848001	14	85848001	1000	1	3.96E-06	-1.0161415	16	1.6		
DMR14:86443001	14	86443001	1000	1	8.49E-12	-1.7458236	16	1.6		
DMR14:88246001	14	88246001	1000	1	5.87E-06	-0.5381495	14	1.4	KCNK10	Transport
DMR14:89870001	14	89870001	1000	1	2.93E-06	-0.7453362	7	0.7	EFCAB11	Signaling
DMR14:101419001	14	101419001	1000	1	7.54E-07	-1.3314628	5	0.5		
DMR14:102441001	14	102441001	1000	1	8.11E-06	-0.5396603	17	1.7	TECPR2	
DMR15:23304001	15	23304001	5000	1	8.95E-06	0.6927498	79	1.58	LOC102723534;LOC102723564	
DMR15:27948001	15	27948001	4000	1	3.76E-07	0.788016	60	1.5	OCA2	Transport
DMR15:30071001	15	30071001	1000	1	1.81E-08	-1.4222537	2	0.2		
DMR15:30538001	15	30538001	2000	1	6.28E-06	-0.6043956	80	4		
DMR15:30929001	15	30929001	1000	1	5.48E-07	-1.2338663	11	1.1	FAN1;MTMR10	Signaling
DMR15:39228001	15	39228001	1000	1	3.26E-07	-0.6404296	18	1.8		
DMR15:39734001	15	39734001	1000	1	3.20E-08	-1.031994	4	0.4	FSIP1	
DMR15:40664001	15	40664001	1000	1	1.03E-06	-0.9187085	11	1.1	KNL1	
DMR15:40875001	15	40875001	1000	1	3.64E-07	-0.510186	25	2.5	RHOV	Signaling
DMR15:47833001	15	47833001	1000	1	2.34E-06	0.7225074	6	0.6	LINC01491	
DMR15:58321001	15	58321001	1000	1	9.36E-06	0.758304	7	0.7		
DMR15:64352001	15	64352001	1000	1	5.44E-06	-0.6981554	20	2	CSNK1G1	Signaling
DMR15:71075001	15	71075001	1000	1	7.18E-06	-0.8961214	7	0.7		
DMR15:74713001	15	74713001	1000	1	9.13E-08	-1.0505057	11	1.1	CYP1A1	Metabolism
DMR15:75530001	15	75530001	1000	1	8.54E-06	-0.8751461	10	1	PTPN9	Signaling
DMR15:77297001	15	77297001	2000	2	6.97E-07	-0.5637258	44	2.2	PEAK1	Signaling
DMR15:77618001	15	77618001	1000	1	1.03E-07	-0.591112	34	3.4	LOC105370906;LINGO1	Receptor
DMR15:80004001	15	80004001	2000	1	1.86E-11	-2.3566063	8	0.4		
DMR15:83073001	15	83073001	2000	1	2.03E-08	-0.6913751	8	0.4	BTBD1;MIR4515	Proteolysis
DMR15:83230001	15	83230001	1000	1	5.47E-08	-1.1826017	34	3.4		
DMR15:90721001	15	90721001	3000	1	2.60E-06	-0.5759944	41	1.3666667	CRTC3-AS1;BLM	Epigenetic
DMR15:98404001	15	98404001	1000	1	2.60E-06	0.716932	24	2.4	LINC02351	
DMR16:1871001	16	1871001	2000	1	2.08E-06	-0.9179294	122	6.1	MEIOB;LINC00254	
DMR16:2524001	16	2524001	3000	1	2.54E-06	0.8371793	65	2.1666667	ATP6V0C;AMDHD2;CEMP1;MIR3178	Metabolism;Metabolism
DMR16:4196001	16	4196001	2000	1	6.59E-06	-0.5834519	33	1.65	SRL	Transport
DMR16:5286001	16	5286001	4000	1	6.25E-06	0.7797137	44	1.1	RBFOX1;SNRPCP20	Translation
DMR16:6494001	16	6494001	2000	1	4.74E-06	0.7855977	35	1.75	RBFOX1	Translation
DMR16:6611001	16	6611001	1000	1	4.30E-06	0.6721077	17	1.7	RBFOX1	Translation
DMR16:9332001	16	9332001	1000	1	1.81E-07	0.9316043	18	1.8	LOC101927026	
DMR16:10444001	16	10444001	1000	1	2.86E-06	0.5462708	11	1.1	ATF7IP2;LOC107984877	Transcription
DMR16:10643001	16	10643001	2000	1	1.97E-07	0.7458121	34	1.7	TEKT5	Cytoskeleton
DMR16:15690001	16	15690001	1000	1	4.27E-06	-0.6592619	11	1.1	NDE1	
DMR16:24664001	16	24664001	1000	1	5.62E-07	-0.7068603	13	1.3	TNRC6A;LINC01567	Metabolism
DMR16:27294001	16	27294001	1000	1	4.37E-08	-1.0493805	10	1	NSMCE1-DT	
DMR16:28672001	16	28672001	1000	1	2.87E-06	-0.5388285	16	1.6		
DMR16:29445001	16	29445001	2000	1	1.93E-06	-0.5816586	23	1.15	SMG1P6;BOLA2-SMG1P6;LOC606724;BOLA2;SLX1B;SLX1B-SULT1A4	
DMR16:29712001	16	29712001	2000	1	4.77E-14	-1.8425289	15	0.75		
DMR16:38275001	16	38275001	6000	1	4.80E-08	-0.3951393	85	1.4166667		
DMR16:54883001	16	54883001	1000	1	5.90E-06	-0.6398119	6	0.6	LOC105371275	
DMR16:62900001	16	62900001	1000	1	2.03E-06	0.8533275	10	1		
DMR16:64166001	16	64166001	1000	1	3.71E-06	-1.444532	4	0.4		
DMR16:64189001	16	64189001	1000	1	3.92E-06	-0.7754222	2	0.2		
DMR16:69427001	16	69427001	1000	1	2.95E-06	-0.7097227	18	1.8	CYB5B	Metabolism
DMR16:69912001	16	69912001	3000	1	2.61E-10	-0.618918	46	1.5333333	WWP2	Proteolysis
DMR16:79386001	16	79386001	1000	1	5.83E-07	0.8161976	10	1	MAF	Transcription
DMR16:83118001	16	83118001	2000	1	5.93E-07	0.5623628	35	1.75	CDH13	Cytoskeleton
DMR16:86370001	16	86370001	1000	1	4.55E-06	0.5354299	26	2.6		
DMR16:87076001	16	87076001	1000	1	6.29E-06	0.4992938	25	2.5		

DMR17:10152001	17	10152001	3000	1	6.90E-06	-0.6342505	35	1.1666667	GAS7	Cytoskeleton
DMR17:11439001	17	11439001	1000	1	9.69E-06	0.7693839	12	1.2	SHISA6	
DMR17:31925001	17	31925001	2000	1	9.73E-06	-0.4265214	49	2.45		
DMR17:34372001	17	34372001	2000	1	5.58E-06	0.7077999	16	0.8	CCL1	Growth Factors
DMR17:36554001	17	36554001	1000	1	3.98E-06	-0.6951404	18	1.8	GGNBP2	
DMR17:39421001	17	39421001	2000	1	3.23E-07	-0.5488635	50	2.5	MED1	Transcription
DMR17:50085001	17	50085001	2000	1	3.13E-12	-0.8492054	26	1.3	ITGA3;PDK2	Extracellular Matrix;Signaling
DMR17:55501001	17	55501001	1000	1	2.40E-07	-0.8926105	21	2.1		
DMR17:55627001	17	55627001	1000	1	1.41E-07	0.8630179	13	1.3	LOC101927389	
DMR17:60621001	17	60621001	3000	1	9.09E-06	-0.5044804	52	1.7333333	PPM1D	
DMR17:61313001	17	61313001	1000	1	6.06E-07	0.802821	23	2.3	BCAS3	
DMR17:64302001	17	64302001	1000	1	2.36E-06	-0.6613352	13	1.3	RPL31P57	
DMR17:64368001	17	64368001	1000	1	6.07E-07	-0.8714042	11	1.1	PECAM1	Immune
DMR17:65446001	17	65446001	2000	1	1.72E-06	0.8111563	16	0.8	LINC02563	
DMR17:66015001	17	66015001	1000	1	7.87E-06	0.8722637	4	0.4	CEP112	
DMR17:69274001	17	69274001	1000	1	8.45E-06	-0.6486027	8	0.8	ABCA5	Transport
DMR17:71607001	17	71607001	3000	2	1.54E-18	-1.7418386	12	0.4		
DMR17:72078001	17	72078001	2000	1	4.11E-07	1.1690157	11	0.55	SOX9-AS1;LINC02097	
DMR17:77168001	17	77168001	2000	1	4.17E-06	0.6671004	28	1.4	SEC14L1	
DMR18:3529001	18	3529001	1000	1	3.73E-06	1.0002229	8	0.8	DLGAP1	Cytoskeleton
DMR18:4235001	18	4235001	1000	1	3.19E-07	-0.9927208	9	0.9	DLGAP1	Cytoskeleton
DMR18:5462001	18	5462001	1000	1	1.91E-06	1.0559087	8	0.8	EPB41L3	
DMR18:11654001	18	11654001	1000	1	5.53E-07	0.6082601	23	2.3	MIR7153	
DMR18:20561001	18	20561001	2000	1	7.88E-06	-0.4414894	29	1.45		
DMR18:22307001	18	22307001	1000	1	4.75E-07	0.8099126	8	0.8	LOC101927548	
DMR18:28472001	18	28472001	1000	1	1.15E-06	0.7350527	11	1.1		
DMR18:29511001	18	29511001	1000	1	1.01E-11	-2.4715489	0	0		
DMR18:29594001	18	29594001	4000	2	6.57E-20	-3.3371555	19	0.475		
DMR18:30490001	18	30490001	2000	1	3.05E-14	-2.095592	3	0.15		
DMR18:40502001	18	40502001	1000	1	2.89E-06	0.9006952	4	0.4		
DMR18:44360001	18	44360001	2000	2	1.15E-23	-3.0881526	5	0.25	LINC01478	
DMR18:45420001	18	45420001	1000	1	9.27E-06	0.6304174	12	1.2	SLC14A2;SLC14A2-AS1	Transport
DMR18:46112001	18	46112001	1000	1	3.54E-07	-1.08575	6	0.6	ATP5F1A;HAUS1;RNU6-1278P	Metabolism
DMR18:47850001	18	47850001	1000	1	3.68E-18	-1.9487217	3	0.3	SMAD2;MTCO2P2	Transcription
DMR18:52370001	18	52370001	1000	1	3.70E-06	-0.8652406	7	0.7	DCC	
DMR18:61970001	18	61970001	2000	2	1.04E-13	-2.9645577	0	0	LOC105372158	
DMR18:63209001	18	63209001	1000	1	7.92E-06	0.4569304	13	1.3	BCL2	
DMR18:76432001	18	76432001	1000	1	9.27E-07	0.5258093	22	2.2	ZNF516	Transcription
DMR18:78172001	18	78172001	1000	1	2.01E-06	0.6171124	14	1.4		
DMR18:79038001	18	79038001	3000	1	5.13E-07	0.5790086	51	1.7	LOC105372225	
DMR18:79066001	18	79066001	2000	1	5.00E-06	0.4269676	6	0.3	LOC105372225;ATP9B	Transport
DMR19:103001	19	103001	3000	1	6.21E-07	0.7978508	32	1.0666667	OR4G3P;OR4G1P;OR4F17	Receptor
DMR19:107001	19	107001	1000	1	3.47E-06	-1.5578813	2	0.2	OR4G1P;OR4F17	Receptor
DMR19:183001	19	183001	1000	1	4.34E-08	-0.7505166	19	1.9	SEPTIN14P19;CICP19	
DMR19:7053001	19	7053001	2000	2	1.51E-09	-1.4810199	11	0.55	MBD3L2;MBD3L3	
DMR19:8099001	19	8099001	1000	1	6.88E-06	-0.4870898	26	2.6	FBN3	Extracellular Matrix
DMR19:8915001	19	8915001	2000	1	7.16E-06	0.7996119	16	0.8	MUC16	
DMR19:9636001	19	9636001	4000	1	8.73E-06	0.7525903	26	0.65	ZNF561-AS1;ZNF562	Transcription
DMR19:11563001	19	11563001	1000	1	4.15E-06	0.6810751	14	1.4	ELOF1	
DMR19:14215001	19	14215001	3000	1	1.38E-10	-2.6994603	64	2.1333333	ADGRL1;RN7SL231P	Signaling
DMR19:14377001	19	14377001	1000	1	2.83E-08	-0.7187939	20	2	ADGRE5	Signaling
DMR19:16412001	19	16412001	2000	1	6.74E-08	0.7645227	39	1.95	EPS15L1;RPS2P51	Transport
DMR19:20370001	19	20370001	2000	1	6.37E-06	0.6827292	31	1.55		
DMR19:20878001	19	20878001	3000	1	5.79E-07	1.038626	27	0.9	LOC100418986;LOC100418987;LOC100418988;LOC100418989	
DMR19:23346001	19	23346001	2000	1	1.36E-06	0.6536909	23	1.15	ZNF91	Transcription
DMR19:26656001	19	26656001	1000	1	7.32E-07	-0.906276	18	1.8		
DMR19:29128001	19	29128001	2000	1	8.40E-06	0.483369	44	2.2		
DMR19:29980001	19	29980001	1000	1	5.92E-06	-0.6814184	15	1.5	URI1	Epigenetic
DMR19:40035001	19	40035001	1000	1	6.87E-06	0.5784605	19	1.9	LOC390933;ZNF780B	
DMR19:40546001	19	40546001	1000	1	5.96E-06	-0.6657574	10	1	SPTBN4	
DMR19:44017001	19	44017001	1000	1	1.09E-06	-0.7718277	20	2	ZNF230;ZNF222-DT;ZNF222	
DMR19:44445001	19	44445001	1000	1	9.71E-06	0.5502865	8	0.8	ZNF229	Transcription
DMR19:44645001	19	44645001	1000	1	2.75E-08	-1.1821316	8	0.8	IGSF23;CEACAM16-AS1;PVR;MIR4531	
DMR19:44800001	19	44800001	1000	1	6.82E-06	0.7101604	13	1.3	CBLC;BCAM	Metabolism;Immune
DMR19:47368001	19	47368001	1000	1	4.36E-06	-0.5576097	17	1.7	DHX34	Transcription

DMR19:48011001	19	48011001	1000	1	6.65E-06	0.5526003	10	1	ELSPBP1	
DMR19:51346001	19	51346001	3000	1	4.32E-06	0.6005004	59	1.9666667	VSIG10L;ETFB	Metabolism
DMR19:52627001	19	52627001	1000	1	1.00E-06	0.6135181	10	1	ZNF701;ZNF83	Transcription
DMR19:54045001	19	54045001	2000	1	1.43E-10	0.7866869	18	0.9	VSTM1	Immune
DMR19:57110001	19	57110001	1000	1	8.66E-08	-1.099351	4	0.4	LOC100419839;USP29	Protease
DMR19:57927001	19	57927001	2000	1	8.69E-06	-0.5839092	31	1.55	ZNF418	
DMR20:897001	20	897001	2000	1	6.47E-08	0.5545497	27	1.35	ANGPT4	Signaling
DMR20:5923001	20	5923001	1000	1	1.62E-06	0.7269924	14	1.4	CHGB;KANK1P1	
DMR20:11075001	20	11075001	1000	1	2.38E-06	-0.7096228	2	0.2		
DMR20:18104001	20	18104001	1000	1	7.12E-06	0.8980963	7	0.7	RNU7-137P	
DMR20:22038001	20	22038001	1000	1	4.10E-06	0.6699085	11	1.1		
DMR20:33472001	20	33472001	2000	1	7.71E-07	-0.5947513	32	1.6		
DMR20:34268001	20	34268001	2000	1	4.83E-06	-0.7460839	97	4.85	ASIP;AHCY	Signaling;Metabolism
DMR20:38123001	20	38123001	2000	1	1.34E-10	-1.2575443	11	0.55	TGM2	Transport
DMR20:43490001	20	43490001	2000	1	5.39E-06	-0.5828598	28	1.4		
DMR20:44134001	20	44134001	1000	1	2.04E-09	-2.7965221	0	0	JPH2	
DMR20:46854001	20	46854001	1000	1	6.30E-06	0.6804702	17	1.7	RN7SKP33;RPL13P14	
DMR20:47192001	20	47192001	1000	1	5.52E-06	-0.5275352	18	1.8	EYA2	
DMR20:51031001	20	51031001	2000	1	4.39E-07	0.627032	25	1.25	KCNG1	Transport
DMR20:53868001	20	53868001	2000	1	7.01E-07	-1.314101	13	0.65	SUMO1P1	
DMR20:54148001	20	54148001	1000	1	2.09E-06	-0.5786935	12	1.2	CYP24A1	Metabolism
DMR20:60529001	20	60529001	1000	1	2.55E-06	0.9736279	12	1.2		
DMR20:61008001	20	61008001	2000	1	1.83E-06	0.5663553	28	1.4		
DMR20:61013001	20	61013001	1000	1	3.59E-07	-0.7340015	6	0.6		
DMR20:61051001	20	61051001	1000	1	6.39E-06	0.7948975	24	2.4		
DMR21:6002001	21	6002001	1000	1	9.29E-08	-0.9152921	11	1.1	LOC105379490	
DMR21:10271001	21	10271001	1000	1	9.22E-07	-0.4842888	3	0.3		
DMR21:10273001	21	10273001	1000	1	4.24E-07	-0.6500955	2	0.2		
DMR21:10324001	21	10324001	2000	1	9.92E-08	-0.7112719	8	0.4	EIF3FP1	
DMR21:12615001	21	12615001	3000	1	8.21E-06	0.8032755	52	1.7333333		
DMR21:20347001	21	20347001	1000	1	8.72E-06	0.752077	12	1.2	RNU6-772P;RN7SKP147	
DMR21:33322001	21	33322001	2000	1	1.78E-07	-1.1883335	20	1	IFNAR1	Receptor
DMR21:41942001	21	41942001	1000	1	5.02E-06	0.7618477	19	1.9	C2CD2;SNORA91	
DMR21:42768001	21	42768001	3000	1	8.52E-06	0.4878213	78	2.6	PDE9A;LOC107985504;LINC01668	Signaling
DMR21:44902001	21	44902001	1000	1	5.72E-06	0.6258465	32	3.2	ITGB2;LOC107987303	Extracellular Matrix
DMR21:45831001	21	45831001	2000	1	8.33E-06	0.5300478	38	1.9	PCBP3;PCBP3-AS1	Metabolism
DMR22:11211001	22	11211001	4000	3	3.16E-07	-1.0454424	22	0.55		
DMR22:11835001	22	11835001	1000	1	2.21E-06	-0.7543955	7	0.7	LOC107984037;LOC107984030	
DMR22:15808001	22	15808001	1000	1	3.53E-06	-0.8634727	4	0.4	DUXAP8;BMS1P22	
DMR22:16781001	22	16781001	1000	1	4.29E-06	0.7584044	15	1.5	XKR3	
DMR22:17807001	22	17807001	1000	1	3.68E-06	0.7535097	15	1.5	MICAL3	
DMR22:18203001	22	18203001	3000	1	1.36E-12	-3.0796614	9	0.3	FAM230D;LOC105372858	
DMR22:18236001	22	18236001	3000	2	2.26E-15	-1.6544537	10	0.3333333	LOC105379518	
DMR22:18387001	22	18387001	2000	2	4.13E-09	-2.6563469	9	0.45	FAM230J	
DMR22:18729001	22	18729001	2000	1	6.41E-12	-1.9187964	9	0.45	PPP1R26P4;FAM230E	
DMR22:18891001	22	18891001	2000	1	2.32E-13	-0.7840924	6	0.3	FAM230F	
DMR22:21505001	22	21505001	2000	1	3.33E-06	-0.5760586	44	2.2	PI4KAP2	Signaling
DMR22:21654001	22	21654001	2000	1	1.24E-06	-0.6483935	43	2.15	SDF2L1;LOC107985532;MIR301B;MIR130B	Transport
DMR22:24605001	22	24605001	1000	1	5.08E-10	-2.61146	0	0	GGT1	Protease
DMR22:25485001	22	25485001	2000	1	3.41E-06	0.829752	21	1.05		
DMR22:29406001	22	29406001	1000	1	1.63E-07	-0.64742	16	1.6	RFPL1;LOC102723305	
DMR22:31358001	22	31358001	2000	1	9.61E-06	-0.7870172	31	1.55	LINC01521	
DMR22:33651001	22	33651001	2000	1	5.62E-06	-0.5289239	38	1.9	LARGE1	Golgi
DMR22:34578001	22	34578001	1000	1	1.32E-06	-1.359634	7	0.7	LOC441996	
DMR22:34776001	22	34776001	1000	1	3.96E-06	0.6111113	9	0.9	LINC02885;LOC105373014	
DMR22:35050001	22	35050001	1000	1	3.55E-06	0.8677909	9	0.9		
DMR22:38048001	22	38048001	1000	1	9.29E-06	-0.4889567	15	1.5	POLR2F;PICK1	Transcription;Transport
DMR22:38339001	22	38339001	2000	1	2.60E-09	-1.6780878	28	1.4	TPTEP2-CSNK1E;TPTEP2	
DMR22:42844001	22	42844001	1000	1	2.74E-06	-0.8039297	8	0.8	ARFGAP3;LOC692246	Signaling
DMR22:44114001	22	44114001	2000	1	1.22E-06	1.1224162	9	0.45	PARVB	Cytoskeleton
DMR22:47003001	22	47003001	2000	1	5.24E-06	0.4047063	27	1.35	TBC1D22A	Signaling
DMR22:47674001	22	47674001	3000	1	1.16E-07	0.7298614	46	1.5333333	LOC284930	
DMR22:48082001	22	48082001	1000	1	4.40E-06	0.8635188	20	2		
DMRX:570001	X	570001	1000	1	3.61E-07	-0.8110547	21	2.1		
DMRX:810001	X	810001	3000	1	5.18E-06	0.5290293	27	0.9		

DMRX:818001	X	818001	2000	1	8.22E-10	-2.03051	24	1.2		
DMRX:1175001	X	1175001	4000	2	1.70E-07	-0.9462529	67	1.675	LOC652608	
DMRX:1946001	X	1946001	1000	1	8.45E-07	-0.8043587	9	0.9		
DMRX:3655001	X	3655001	2000	1	4.51E-08	0.6938879	43	2.15	PRKX;PRKX-AS1	Signaling
DMRX:4227001	X	4227001	1000	1	5.05E-06	-0.8511135	2	0.2		
DMRX:10220001	X	10220001	2000	1	8.33E-06	0.9394146	40	2	CLCN4	Transport
DMRX:16410001	X	16410001	1000	1	5.55E-08	-0.8245284	6	0.6		
DMRX:30194001	X	30194001	1000	1	9.12E-06	0.8316862	9	0.9		
DMRX:40072001	X	40072001	2000	1	2.44E-07	0.8554575	60	3	BCOR	
DMRX:48470001	X	48470001	1000	1	8.32E-07	0.8720056	23	2.3	SLC38A5;FTSJ1	Transport;Epigenetic
DMRX:63870001	X	63870001	2000	1	1.98E-06	0.7961569	10	0.5		
DMRX:65862001	X	65862001	1000	1	7.31E-06	0.819661	9	0.9		
DMRX:67389001	X	67389001	1000	1	7.98E-06	0.8677819	7	0.7		
DMRX:74519001	X	74519001	1000	1	3.50E-06	-0.7013391	19	1.9	SLC16A2	Transport
DMRX:103484001	X	103484001	1000	1	4.74E-06	-0.6895992	15	1.5	LL0XNC01-250H12.3	
DMRX:105388001	X	105388001	1000	1	5.18E-06	-0.8287879	6	0.6	IL1RAPL2	Receptor
DMRX:116462001	X	116462001	2000	1	8.68E-06	1.0020571	38	1.9	SLC6A14;CT83	Transport
DMRX:123051001	X	123051001	2000	2	6.26E-08	-0.7375461	34	1.7		
DMRX:141265001	X	141265001	1000	1	1.90E-08	-0.7406354	14	1.4	RBMX2P2	
DMRX:142059001	X	142059001	1000	1	2.54E-06	0.822806	24	2.4		
DMRX:145743001	X	145743001	1000	1	9.50E-06	0.8605469	14	1.4		
DMRX:150237001	X	150237001	2000	1	1.41E-06	0.9621459	17	0.85	MIR2114;XRCC6P2	
DMRX:152444001	X	152444001	1000	1	6.58E-07	-0.6663755	21	2.1	GABRA3	Ion Channel
DMRY:6922001	Y	6922001	1000	1	1.66E-08	1.1690525	34	3.4	TBL1Y	
DMRY:10906001	Y	10906001	5000	1	3.64E-06	-0.6625109	41	0.82		
DMRY:21156001	Y	21156001	1000	1	1.17E-08	-0.8729594	1	0.1	LOC105377225	
DMRY:24695001	Y	24695001	1000	1	2.06E-06	-0.6122415	27	2.7	TRIM60P11Y	

Supplemental Table S5. DMR table for combined mild, moderate, and severe hypospadias versus control p<1e-05. DMR name, chromosome number, start nucleotide site, length (bp), p-value, maximum log fold change (LFC), CpG number and density, gene annotation, and gene category.

Supplemental Table S6
DMR Associated Genes and Names

Gene Symbol	Gene Name
ACER3	Alkaline Ceramidase 3
ADAM17	ADAM Metallopeptidase Domain 17
ADORA2A	Adenosine A2a Receptor
ALK	ALK Receptor Tyrosine Kinase
APP	Amyloid Beta Precursor Protein
ARRB1	Arrestin Beta 1
ATG5	Autophagy Related 5
BCL11A	B-cell lymphoma/leukemia 11A
BCL2	B-cell lymphoma 2
BMP6	Bone morphogenetic protein 6
CDH1	Cadherin 1
CDH13	Cadherin 13
CDH3	Cadherin 3
CDK2	Cyclin Dependent Kinase 2
CDSN	Corneodesmosin
CNR2	Cannabinoid Receptor 2
DBI	Decibel Isotropic
DHODH	Dihydroorotate Dehydrogenase
ELANE	Elastase, Neutrophil Expressed
EPB41	Erythrocyte Membrane Protein Band 4.1
EPCAM	Epithelial Cell Adhesion Molecul
ERBB4	Erb-B2 Receptor Tyrosine Kinase 4
ETS1	ETS Proto-Oncogene 1
EZH1	Enhancer Of Zeste 1
EZH2	Enhancer Of Zeste 2
FGF1	Fibroblast Growth Factor 1
GATA3	GATA Binding Protein 3
GJB6	Gap Junction Protein Beta 6
GRB2	Growth Factor Receptor Bound Protein 2
HMGB1	High Mobility Group Box 1
HTR2B	5-Hydroxytryptamine Receptor 2B
IFNAR1	Interferon Alpha And Beta Receptor Subunit 1
IL1A	Interleukin 1 Alpha
INSR	Insulin Receptor
ITGA3	Integrin Subunit Alpha 3
ITGA9	Integrin Subunit Alpha 9
JUNB	JunB Proto-Oncogene, AP-1 Transcription Factor Subunit

LEF1	Lymphoid Enhancer Binding Factor 1
LEPR	Leptin Receptor
MAPKAPK2	MAPK Activated Protein Kinase 2
MECP2	Methyl-CpG Binding Protein 2
MED1	Mediator Complex Subunit 1
MIR150	MicroRNA 150
MIR199A1	MicroRNA 199a-1
MYT1L	Myelin Transcription Factor 1 Like
NFATC1	Nuclear Factor Of Activated T Cells 1
NR3C2	Nuclear Receptor Subfamily 3 Group C Member 2
P2RY2	Purinergic Receptor P2Y2
PIKFYVE	Phosphoinositide Kinase, FYVE-Type Zinc Finger Containing
PLD2	Phospholipase D2
PPP1R13L	Protein Phosphatase 1 Regulatory Subunit 13 Like
PXN	Paxillin
RIPK4	Receptor Interacting Serine/Threonine Kinase 4
RXRA	Retinoid X Receptor Alpha
SEMA3A	Semaphorin 3A
SH3PXD2A-AS1	SH3PXD2A Antisense RNA 1
SLC12A8	Solute Carrier Family 12 Member 8
SLC6A4	Solute Carrier Family 6 Member 4
SMAD2	SMAD Family Member 2
SP6	Sp6 Transcription Factor
STIM1	Stromal Interaction Molecule 1
THRB	Thyroid Hormone Receptor Beta
TIAM1	TIAM Rac1 Associated GEF 1
TNFAIP6	TNF Alpha Induced Protein 6
TP63	Tumor Protein P6
TRPC1	Transient Receptor Potential Cation Channel Subfamily C Member 1
TRPM7	Transient Receptor Potential Cation Channel Subfamily M Member 7
TRPM8	Transient Receptor Potential Cation Channel Subfamily M Member 8
VEGFD	Vascular Endothelial Growth Factor D
